# Chromosome-level genome assembly of the hard-shelled mussel *Mytilus coruscus*, a widely distributed species from the temperate areas of East Asia

**DOI:** 10.1093/gigascience/giab024

**Published:** 2021-04-23

**Authors:** Jin-Long Yang, Dan-Dan Feng, Jie Liu, Jia-Kang Xu, Ke Chen, Yi-Feng Li, You-Ting Zhu, Xiao Liang, Ying Lu

**Affiliations:** International Research Center for Marine Biosciences, Ministry of Science and Technology, Shanghai Ocean University, 999 Huchenghuan Road, Shanghai 201306, China; Key Laboratory of Exploration and Utilization of Aquatic Genetic Resources, Ministry of Education, Shanghai Ocean University, 999 Huchenghuan Road, Shanghai 201306, China; Southern Marine Science and Engineering Guangdong Laboratory, Guangzhou 511458, China; International Research Center for Marine Biosciences, Ministry of Science and Technology, Shanghai Ocean University, 999 Huchenghuan Road, Shanghai 201306, China; Key Laboratory of Exploration and Utilization of Aquatic Genetic Resources, Ministry of Education, Shanghai Ocean University, 999 Huchenghuan Road, Shanghai 201306, China; International Research Center for Marine Biosciences, Ministry of Science and Technology, Shanghai Ocean University, 999 Huchenghuan Road, Shanghai 201306, China; Key Laboratory of Exploration and Utilization of Aquatic Genetic Resources, Ministry of Education, Shanghai Ocean University, 999 Huchenghuan Road, Shanghai 201306, China; International Research Center for Marine Biosciences, Ministry of Science and Technology, Shanghai Ocean University, 999 Huchenghuan Road, Shanghai 201306, China; Key Laboratory of Exploration and Utilization of Aquatic Genetic Resources, Ministry of Education, Shanghai Ocean University, 999 Huchenghuan Road, Shanghai 201306, China; International Research Center for Marine Biosciences, Ministry of Science and Technology, Shanghai Ocean University, 999 Huchenghuan Road, Shanghai 201306, China; Key Laboratory of Exploration and Utilization of Aquatic Genetic Resources, Ministry of Education, Shanghai Ocean University, 999 Huchenghuan Road, Shanghai 201306, China; International Research Center for Marine Biosciences, Ministry of Science and Technology, Shanghai Ocean University, 999 Huchenghuan Road, Shanghai 201306, China; Key Laboratory of Exploration and Utilization of Aquatic Genetic Resources, Ministry of Education, Shanghai Ocean University, 999 Huchenghuan Road, Shanghai 201306, China; International Research Center for Marine Biosciences, Ministry of Science and Technology, Shanghai Ocean University, 999 Huchenghuan Road, Shanghai 201306, China; Key Laboratory of Exploration and Utilization of Aquatic Genetic Resources, Ministry of Education, Shanghai Ocean University, 999 Huchenghuan Road, Shanghai 201306, China; International Research Center for Marine Biosciences, Ministry of Science and Technology, Shanghai Ocean University, 999 Huchenghuan Road, Shanghai 201306, China; Key Laboratory of Exploration and Utilization of Aquatic Genetic Resources, Ministry of Education, Shanghai Ocean University, 999 Huchenghuan Road, Shanghai 201306, China; International Research Center for Marine Biosciences, Ministry of Science and Technology, Shanghai Ocean University, 999 Huchenghuan Road, Shanghai 201306, China; Key Laboratory of Exploration and Utilization of Aquatic Genetic Resources, Ministry of Education, Shanghai Ocean University, 999 Huchenghuan Road, Shanghai 201306, China

**Keywords:** Mytilus coruscus, genome sequencing, Hi-C, chromosome, metamorphosis

## Abstract

**Background:**

The hard-shelled mussel (*Mytilus coruscus*) is widely distributed in the temperate seas of East Asia and is an important commercial bivalve in China. Chromosome-level genome information of this species will contribute not only to the development of hard-shelled mussel genetic breeding but also to studies on larval ecology, climate change biology, marine biology, aquaculture, biofouling, and antifouling.

**Findings:**

We applied a combination of Illumina sequencing, Oxford Nanopore Technologies sequencing, and high-throughput chromosome conformation capture technologies to construct a chromosome-level genome of the hard-shelled mussel, with a total length of 1.57 Gb and a median contig length of 1.49 Mb. Approximately 90.9% of the assemblies were anchored to 14 linkage groups. We assayed the genome completeness using BUSCO. In the metazoan dataset, the present assemblies have 89.4% complete, 1.9% incomplete, and 8.7% missing BUSCOs. Gene modeling enabled the annotation of 37,478 protein-coding genes and 26,917 non-coding RNA loci. Phylogenetic analysis showed that *M. coruscus* is the sister taxon to the clade including *Modiolus philippinarum* and *Bathymodiolus platifrons*. Conserved chromosome synteny was observed between hard-shelled mussel and king scallop, suggesting that this is shared ancestrally. Transcriptomic profiling indicated that the pathways of catecholamine biosynthesis and adrenergic signaling in cardiomyocytes might be involved in metamorphosis.

**Conclusions:**

The chromosome-level assembly of the hard-shelled mussel genome will provide novel insights into mussel genome evolution and serve as a fundamental platform for studies regarding the planktonic-sessile transition, genetic diversity, and genomic breeding of this bivalve.

## Context

Marine mussels, which belong to the phylum Mollusca, settle on most immersed surfaces of substrata and play a crucial role in marine ecosystems. As healthy and sustainable food items, these mussels are beneficial for humans owing to their high economic value for fishery and aquaculture, constituting >8% of mollusc aquaculture production [1]. Simultaneously, mussels are also known as typical macrofouling organisms that result in detrimental economic and ecological consequences for the maritime and aquaculture industries [2–4]. Mussels have been used as model organisms for adaptation to climate change, biomonitoring, integrative ecomechanics, biomaterials, larval ecology, settlement and metamorphosis, adhesion, bacteria-host interaction, and biofouling and antifouling studies [5–12]. Although they are significant for biology, ecology, and the economy, whole-genome information of marine mussels is limited [13, 14] and this lack of knowledge postpones our understanding the molecular basis of adaption, evolution, breeding, genetic manipulation, bacteria-host interaction, and settlement mechanisms.

Like many other marine invertebrates, marine mussels also possess a free-swimming larval phase. After this stage, these minute larvae will settle on the substrata and finish metamorphosis transition, accompanied by dramatic remodeling of their anatomy [4, 15]. Multiple physicochemical stimuli play critical roles in the process of larval settlement and metamorphosis [15–17]. Thus, understanding of the larvae-juvenile transition process is still a keystone question in marine biology, larval ecology, aquaculture, biofouling, and antifouling [4, 15, 18, 19]. The finding that chemical cues from bacterial biofilms trigger settlement and metamorphosis is widespread among metazoans [15, 16, 18].

**Figure 1:**
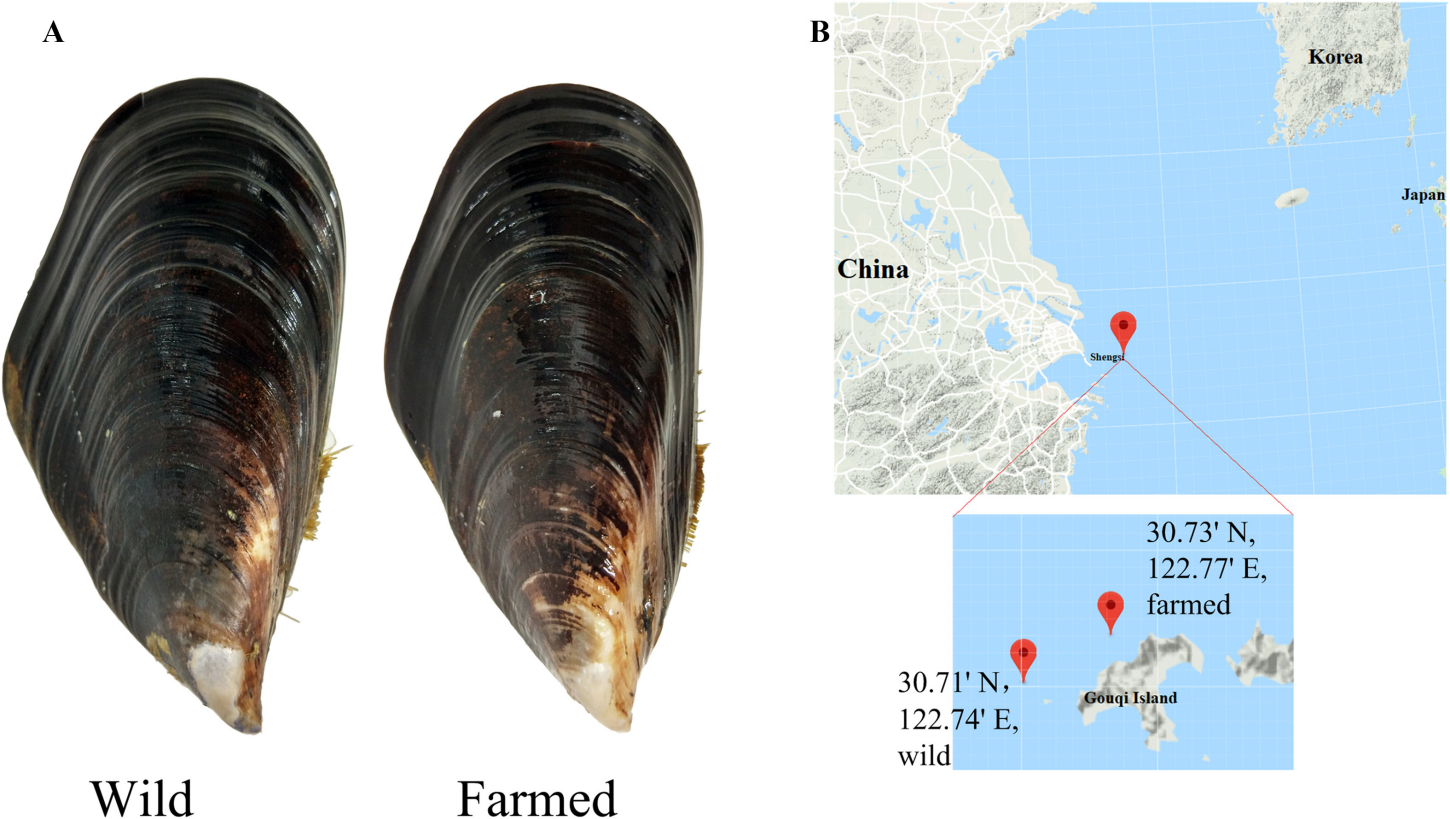
Sequenced individuals and sampling sites. **A**. Pictures of the sequenced individuals collected in Shengsi. A wild *M. coruscus* adult was used for genome sequencing. Both wild and farmed populations were used for resequencing. **B**. The geographic locations of the sampling sites.

The hard-shelled mussel (*Mytilus coruscus* Gould 1861, NCBI:txid42192, Fig. 1) mainly inhabits temperate areas along the coastal waters of China, Japan, Korea, and the Far East of Russia, covering from the East China Sea to Sea of Japan [20]. In China, the hard-shelled mussel is an important commercial bivalve, as well as a typical macrofouling organism. As a sessile marine bivalve, the hard-shelled mussel needs to adapt to the hostile and complex environments of intertidal regions. Most studies have focused on the planktonic-sessile transition mechanism of receptor and biofilm regulation, host-bacteria interaction, aquaculture, and biofouling and antifouling in this species [3–5, 12, 21–23]. To date, no genome of any member of the genus *Mytilus* has been assembled at the chromosome level, although a draft genome of *M. coruscus* [24] and an improved genome of *Mytilus galloprovincialis* [13, 25] have been reported. The lack of whole-genome information has hindered the development of hard-shelled mussel genetic breeding, larval ecology, climate change biology, marine biology, aquaculture, biofouling, and antifouling studies.

In this study, we report a chromosome-level assembly of the hard-shelled mussel genome obtained by combining Illumina sequencing, Oxford Nanopore Technologies (ONT) sequencing, and high-throughput chromosome conformation capture (Hi-C) technologies. We validated the genome assemblies by chromosome synteny analysis, comparing them with the published chromosome-level genomes of the most studied molluscs. Larvae at 5 early developmental stages were subjected to RNA sequencing (RNA-seq) analysis for the profiling of gene expression during metamorphosis. Accessible chromosome-level genome datasets [26, 27] will facilitate comparative genomics studies on chromosome rearrangements across different species.

## Methods

### Sample information and collection

Wild individuals for genome sequencing were collected from the coast of Shengsi, Zhejiang province, which is the central coast of the Chinese mainland, and one of the original and main breeding areas of the hard-shelled mussel in China. Farmed and wild adults were also collected from the coast of Shengsi (122 46.2 E 30 43.8 N and 122 44.4 E, 30 42.6 N, respectively) (Fig.   1). A female wild adult with a mature ovary was dissected, and the adductor muscle was collected to isolate high molecular weight genomic DNA for the sequencing of the reference genome. The DNA extracted from the farmed and wild populations (10 individuals per population) was pooled for genome resequencing. Adductor muscle, mantle, gill, digestive gland, hemocyte, labial palp, female gonad, male gonad, foot, and gut tissues were dissected from fresh samples for transcriptome sequencing to assist with the prediction of protein-coding genes.

### Isolation of genomic DNA and RNA

Genomic DNA was extracted from fresh adductor muscle tissue using the sodium dodecyl sulfate extraction method [28] and then used for sequencing on an ONT PromethION platform (Oxford Nanopore, Oxford, UK). Using the TIANamp Marine Animals DNA kit (Tiangen,Beijing, China), DNA for whole-genome resequencing was extracted from the muscles of 5 female and 5 male individuals from each population. Using the RNAiso Plus kit (TaKaRa, Shiga, Japan), total RNA was extracted from 10 different tissues of 5 female and 5 male individuals from each population to obtain a large gene expression dataset. Fresh muscle cells were crosslinked with formaldehyde, and digestion, marking of DNA ends, and blunt-end ligation were performed as described in a previous study [29]. The purified DNA was used for Hi-C.

### Genome sequencing with different technologies

**Figure 2:**
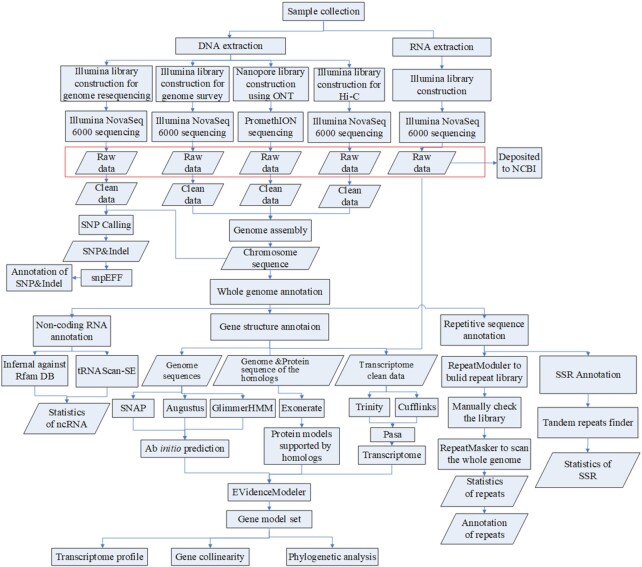
Workflow of genome sequencing and annotation. The rectangles indicate the steps of data treatment and the diamonds indicate output or input data. ncRNA: non-coding RNA.

A combined sequencing strategy was applied to obtain the hard-shelled mussel genome (Fig. 2). Qualified DNA was filtered using a BluePippin^TM^ System to extract large fragments. The large-fragment DNA was used to construct a library using the ONT Template prep kit and the NEB Next FFPE DNA Repair Mix kit (New England Biolabs, Ipswich, MA, USA). A high-quality library with a mean length of 20 kb was sequenced on the ONT PromethION platform with the corresponding R9 cell and ONT sequencing reagent kit. A total of 246.8 Gb of data (∼159× coverage) were generated (Table 1).

**Table 1:** Statistics of whole-genome sequencing using Illumina and ONT

Type	Method	Library size (bp)	Reads No.	Clean data (Gb)	Length (bp)	Coverage (×)
Genome	Illumina	300–350	1,235,384,620	160.6	150	104
	ONT	20,000	11,108,773	246.8	30,945 (N50)	159
	Hi-C		832,911,978	249.6	150	161
Transcriptome	Illumina	300–350	787,692,308	102.4	150	

Sequencing of Hi-C and genome survey libraries was performed on an Illumina sequencing platform. Briefly, the extracted DNA was fragmented to a size of 300–350 bp using an E210 Focused Ultrasonicator (Covaris,Woburn, MA, USA). The construction of paired-end (PE) libraries encompassed the successive steps of end repair, poly(A) addition, barcode indexing, purification, and PCR amplification. The libraries were sequenced with the Illumina NovaSeq 6000 platform (Illumina, San Diego, CA, USA) to generate 150-bp PE reads. Sequencing of the Hi-C libraries generated a total of 249.6 Gb of data (∼161× coverage), and sequencing of the genome survey libraries generated a total of 160.6 Gb of data (∼104× coverage).

The qualified RNA extracted from the same tissues of 10 individuals was equally mixed for RNA-seq. The sample was enriched in messenger RNA by extracting poly(A) transcripts from total RNA using oligo-d(T) magnetic beads. Sequencing libraries were prepared using the NEBNext® Ultra™ RNA Library Prep Kit for Illumina® (New England Biolabs, Ipswich, MA, USA) following the manufacturer's recommendations. A total of 10 libraries were sequenced on the Illumina NovaSeq 6000 platform in a 150-bp PE mode.

The raw reads from the Illumina sequencing platform were cleaned using FastQC45 and HTQC46 by the following steps: (i) filtered reads with adapter sequence; (ii) filtered PE reads with 1 read having >10% N bases; (iii) filtered PE reads with any end having >50% inferior quality (≤5) bases.

### Genome survey and contig assembly

**Figure 3:**
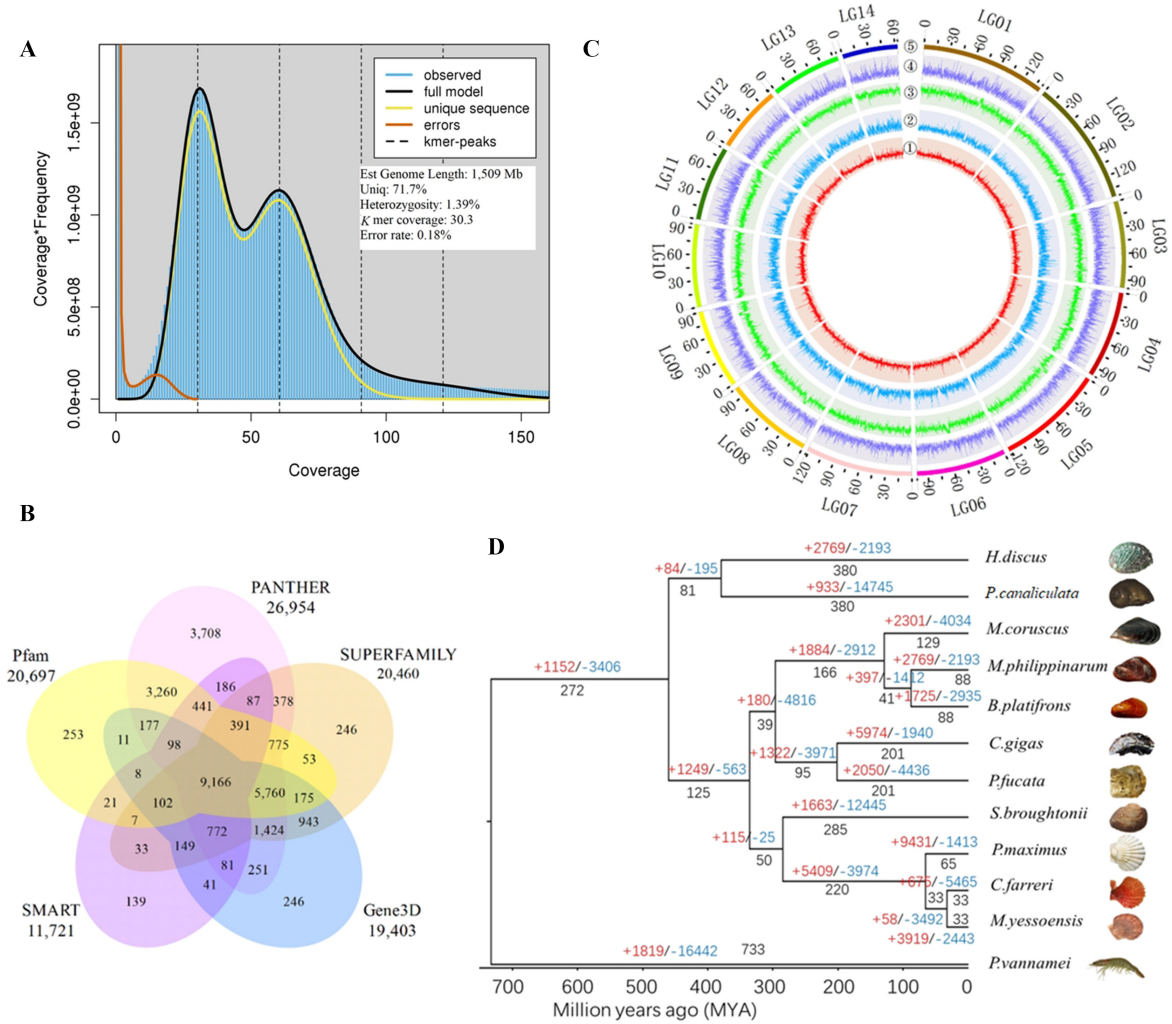
Annotation and evolution. **A**. GenomeScope plot of the 51-mer content within the hard-shelled mussel genome. Estimates of genome size and read data are shown. **B**. Venn diagram indicating the number of genes that were annotated in ≥1 database. **C**. Genomic landscape of *M. coruscus*. The chromosomes are labeled as LG01–LG14. From the outer to the inner circle: 5, marker distribution across 14 chromosomes at a megabase scale; 4, gene density across the whole genome; 3, SNP density; 2 and 1, number of repetitive sequences and GC content across the genome, respectively. 1–5 are drawn in non-overlapping 0.1-Mb sliding windows. The length of chromosomes is defined by the scale (Mb) on the outer circles. **D**. Phylogenetic tree based on protein sequences from 12 metazoan genomes, namely, those of *Chlamys farreri* (PRJNA185465), *Pinctada fucata martensii* (GCA_002216045.1), *Modiolus philippinarum* (GCA_002080025.1), *Crassostrea gigas* (GCF_000297895.1), *Mytilus coruscus, Bathymodiolus platifrons* (GCA_002080005.1), *Mizuhopecten yessoensis* (GCA_002113885.2), *Penaeus vannamei* (ASM378908v1), *Pecten maximus* (GCA_902652985.1), *Scapharca* (*Anadara*) *broughtonii* (PRJNA521075), *Pomacea canaliculata* (PRJNA427478), and *Haliotis discus hannai* (PRJNA317403).

The size of the hard-shelled mussel genome was estimated using the *k*-mer–based method implemented in Jellyfish (version 2.3.0) with values of 51-mers [30] and GenomeScope (10,000× cut-off) [31]. The *k*-mers refer to all the *k*-mer frequency distributions from a read obtained through Illumina DNA sequencing. The homozygous peak of the assembly was at 57× coverage and the heterozygous peak was at 28× coverage (Fig. 3A). The assessment of genome size by *k*-mer counting suggested a complete genome size of ∼1.51 Gb (Fig. 3A), which is close to the final assembly (1.57 Gb) and cytogenetic estimates [32]. Sequence alignment between the previous assembly (1.90 Gb) [24] and the one in this study revealed considerable heterozygous redundancies in the former. This kind of overestimation of genome size usually occurs in fragmented assemblies, like the recently published *M. galloprovincialis* genome [25].

Genome assembly from long-read data was carried out following 3 methods. First, long reads were *de novo* assembled using the Canu (Canu, RRID:SCR_015880) v1.5 software with default parameters [33]; next, error correction was performed with Racon v1.3.1 [34]. Then, further polishing with Illumina short-read data was conducted using Pilon (Pilon, RRID:SCR_014731) v1.22 [35]. The final assembly was ∼1.57 Gb in size, consisting of 6,449 contigs with an overall median length (N50) of 1.49 Mb, while the previously published draft genome only had an N50 of 0.66 Mb [24]. The present genome had a heterozygous rate of 1.39% (also calculated by GenomeScope) and a mean GC content of ∼32%.

### Anchoring of the contigs to pseudo-moleculars with Hi-C data

To complete the assembly of the hard-shelled mussel genome, Hi-C technology was carried out to generate information on the interactions among contigs. DNA from fresh adductor muscle tissue was used to prepare a Hi-C library. This was then sequenced on the Illumina NovaSeq 6000 platform, producing 249.6 Gb of reads (Table 1). These reads were aligned to the assembled contigs using BWA (BWA, RRID:SCR_010910) aligner v0.7.10-r789 [36]. Lachesis v2e27abb was applied to anchor the contigs onto the linkage groups using the agglomerative hierarchical clustering method [37]. Finally, 2,029 contigs representing 90.9% of the total assemblies were successfully anchored to 14 chromosomes (Table 2); this number was consistent with the outputs of the karyotype [38]. The unclosed gaps only occupy 0.014% of the assembly (201,500 bp), which is filled with Ns (Table 2). The N50 of the anchored contigs was >1.7 Mb, ∼1.14 times that of the initial assemblies from the ONT long reads.

**Table 2:** Results of contig anchoring on pseudochromosomes using Hi-C data

LG	Length (bp)	Gene No.	Contig N50 length (bp)	Contig No.	No. of gaps (bp)
LG01	141,585,364	3,535	2,274,693	122	12,100
LG02	144,576,766	3,347	3,700,000	88	8,700
LG03	99,268,963	2,454	1,068,300	196	19,500
LG04	99,542,347	2,554	894,135	225	22,400
LG05	122,084,758	3,159	2,900,000	96	9,500
LG06	102,382,230	2,442	2,078,006	106	10,500
LG07	122,148,919	2,720	3,437,001	91	9,000
LG08	101,363,610	2,456	2,665,365	138	13,700
LG09	90,511,107	2,243	1,458,983	124	12,300
LG10	94,491,177	2,295	1,062,238	172	17,100
LG11	85,619,405	1,927	619,639	249	24,800
LG12	76,129,233	1,754	767,559	180	17,900
LG13	79,962,191	1,837	2,050,444	117	11,600
LG14	63,392,598	1,391	1,000,000	125	12,400
Total	1,423,058,668	34,114	1,700,000	2,029	201,500

Gaps are preset at 100 Ns.

### Genome annotation

A *de novo* repeat annotation of the hard-shelled mussel genome was carried out using RepeatModeler (RepeatModeler, RRID:SCR_015027) version 1.0.11 [39] and RepeatMasker (RepeatMasker, RRID:SCR_012954) version 4.0.7 [40]. RepeatModeler was used to construct the repeat library, which was then examined using 2 other programs, RECON and RepeatScout (RepeatScout, RRID:SCR_014653). The yielded consensus sequences were manually checked by aligning to the genes from the GenBank database (nt and nr; released in October 2019) to avoid sequences of the high-copy genes being masked in following process with RepeatMasker. The final repeat library consisted of 2,264 consensus sequences with the respective classification information, which was used to run RepeatMasker against the genome assemblies. The repetitive sequences constituted a length of 735.6 Mb, representing 47.4% of the total genome length (Supplementary Table S1). Simple sequence repeats (SSRs) were identified using Tandem Repeats Finder V 4.04. Only monomers, dimers, trimers, tetramers, pentamers, and hexamers with ≥4 repeat units were considered. The total length of the 5,324 identified SSRs was ∼138.0 kb.

Conserved non-coding RNAs were predicted using the Rfam 11.0 databases. Putative microRNAs and ribosomal RNAs were predicted using Infernal (Infernal, RRID:SCR_011809) version 1.1.2 [41], and transfer RNAs (tRNAs) were predicted with tRNAscan-SE (tRNAscan-SE, RRID:SCR_010835) v2.0.3. A total of 9,186 microRNAs, 342 ribosomal RNAs, and 1,881 tRNAs were detected (Supplementary Table S2).

Protein-coding genes were predicted using a combined strategy of *ab initio* prediction, homology-based prediction, and transcriptome-based prediction (Fig. 2). The *ab initio* prediction was conducted using Augustus (Augustus: Gene Prediction, RRID:SCR_008417) version 3.1 [38], GlimmerHMM (GlimmerHMM, RRID:SCR_002654) version 1.2 [39], and SNAP (version 2006–07-28) software [42]. For homology-based prediction, protein sequences of 2 closely related mollusk species (*Modiolus philippinarum* and *Bathymodiolus platifrons*), downloaded from GenBank, were aligned to the genome assemblies using Exonerate (version 2.2.0) [43]. In parallel, transcriptomic data from 10 tissues (GenBank SRA accession ID: PRJNA578350) were assembled *de novo* using Trinity (Trinity, RRID:SCR_013048) version 2.4.0 [44] and Cufflinks (Cufflinks, RRID:SCR_014597) version 2.2.1 [45]. The outputs of both assemblers were integrated using PASA, version 2.3.3 [46]. After merging of all of these predictions using EVidenceModeler (v1.1.0) [46], a total of 37,478 final gene models were generated (Table 3), a number lower than that of the previously published 42,684 gene models in the draft genome [24]. Functional annotations displayed that 35,471 protein-coding genes (94.6% of the 37,478 gene models) align to ≥1 of the InterPro (version 5.22–61.0) [47], GO [48], KEGG [49], Swissprot [50], and NCBI non-redundant protein (NR) functional databases (Table 4; Fig. 3B). This information is illustrated in a genome landscape map (Fig. 3C). Using a bidirectional BLASTp between the 2 assemblies, we observed that a considerable proportion of heterozygous redundancies (>20%) were probably included into the previous draft assemblies (Supplementary Table S3), which might be owing to the widespread hemizygosity and massive gene presence/absence variation [25, 51] or assembling errors.

**Table 3:** General statistics of the predicted protein-coding genes

Gene set		No.	Mean length (bp)		Mean exons per gene	Mean length (bp)	
			Transcripts	CDSs		Exons	Introns
*De novo*	SNAP	52,359	15,377	488	4.8	101	3,894
	GlimmerHMM	196,665	7,017	525	3.3	157	2,776
	Augustus	67,930	8,512	1,036	4.1	250	2,380
Homolog	*B. platifrons*	34,836	10,631	784	3.6	217	3,778
	*M. philippinarum*	27,088	7,174	643	2.8	227	3,568
RNAseq		53,578	16,183	966	6.0	275	2,900
Final EVM models		37,478	14,735	1,290	5.9	217	2,727

CDS: coding sequence.

**Table 4:** General statistics of gene functional annotation

Type	No. (%)
Total	37,478 (100)
Annotated	
InterPro	32,821 (87.6)
GO	18,497 (49.4)
KEGG	7,625 (20.3)
Swissprot	16,868 (45.0)
NR	31,489 (84.0)
Total	35,471 (94.6)
Unannotated	2,007 (5.4)

### Phylogenetic analysis

Gene clusters were identified among 12 selected genomes, namely, those of *Chlamys farreri* (PRJNA185465), *Pinctada fucata martensii* (GCA_002216045.1), *M. philippinarum* (GCA_002080025.1), *Crassostrea gigas* (GCF_000297895.1), *B. platifrons* (GCA_002080005.1), *Mizuhopecten yessoensis*(GCA_002113 885.2), *Penaeus vannamei* (ASM378908v1), *Pecten maximus* (GCA_902652985.1), *Scapharca* (*Anadara*) *broughtonii* (PRJNA521075), *Pomacea canaliculata* (PRJNA427478), *Haliotis discus hannai* (PRJNA317403), and *M. coruscus*, using OrthoMCL (OrthoMCL DB: Ortholog Groups of Protein Sequences, RRID:SCR_007839) version 1.4 with a BLASTp cut-off value of 10^−5^ and an inflation value of 1.5 [52]. A total of 448 single-copy genes identified by OrthoDB were aligned and concatenated. The amino acid sequences were first aligned using MUSCLE (MUSCLE, RRID:SCR_011812) [53] and then further concatenated to create 1 supergene sequence for each species and form a data matrix. The phylogenetic relationships among different supergenes were then assessed using a maximum-likelihood model in RAxML (RAxML, RRID:SCR_006086) version 8 [54] with the optimal substitution model of PROTGAMMAJTT. The robustness of the maximum-likelihood tree was assessed using the bootstrap method (100 pseudo-replicates). Furthermore, single-copy orthologs and 1 reference divergence time on the root node obtained from the TimeTree database [55] were used to calibrate the divergence dates of other nodes on this phylogenetic tree using the MCMC_TREE_ tool in the PAML (PAML, RRID:SCR_014932) package [56]. Visualization of phylogenetic relationships with FigTree (version 1.4.3) [57] suggested that *M. coruscus* is the sister taxon to the clade containing *M. philippinarum* and *B. platifrons*, with a divergence time of ∼129 million years ago (Fig. 3D).

### Whole-genome resequencing of farmed and wild individuals

**Figure 4:**
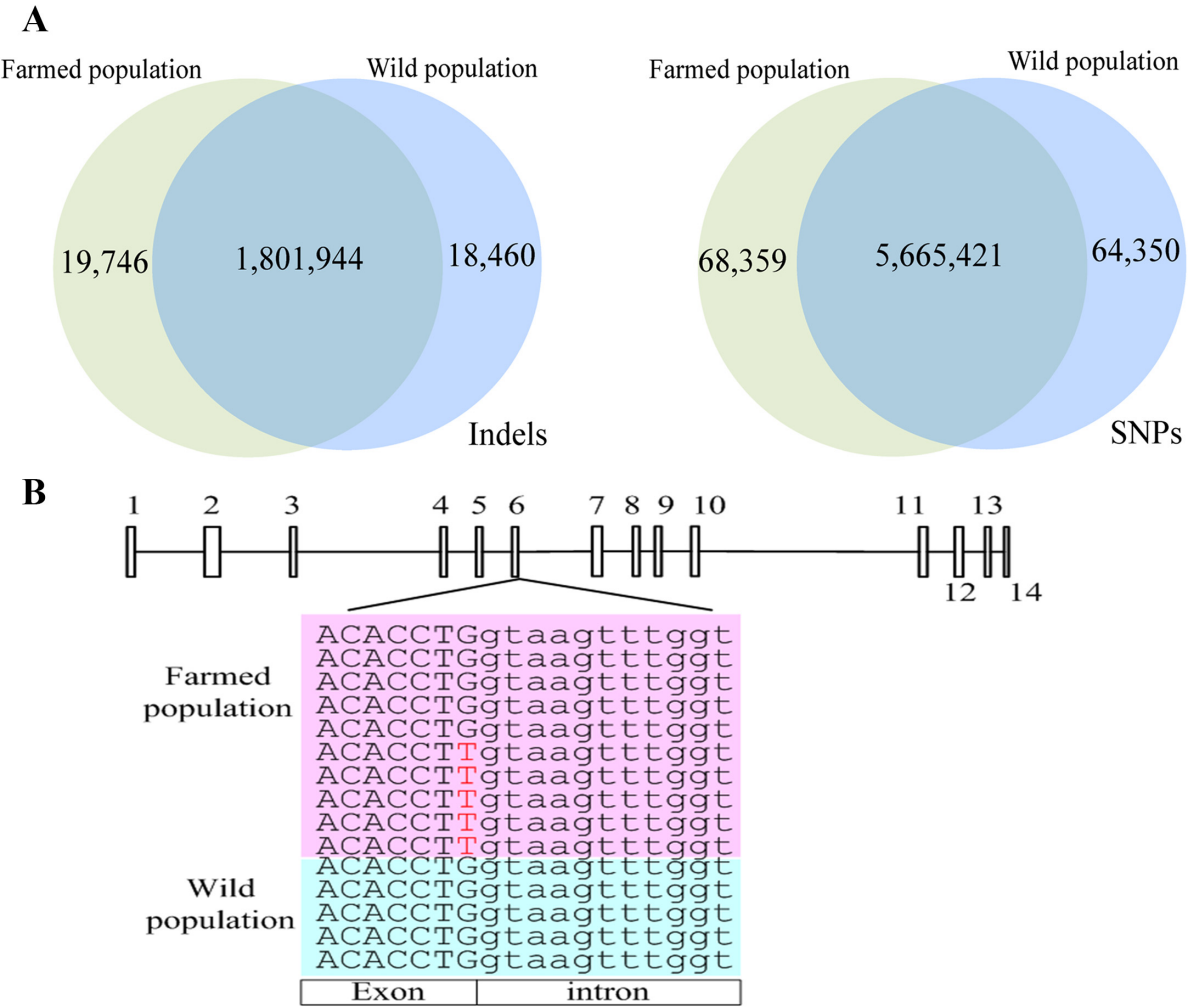
Sequence variations between farmed and wild populations**. A**. Venn diagrams showing the number and distribution of indels and SNPs between the farmed and wild populations. **B**. Differences in the number of SNPs on the exons of *chitobiase*. The rectangles indicate the 14 exons of the *chitobiase* gene and the lines between the 14 rectangles indicate introns; the pink matrix represents reads from the farmed population, and the blue matrix represents reads from the wild population. Bases denoted by capital letters are located on exons, whereas those denoted by small letters are located on introns.

Chromosome-level genomes allow resequencing and population genetic studies. We performed a preliminary assay to detect sequence variation by sequencing 2 genomic DNA pools of wild population and farmed population. A total of 50.4 and 46.7 Gb of Illumina clean reads were finally generated in farmed and wild samples, respectively. More than 89% of the reads were aligned to the reference genome with BWA (v0.7.10-r789) [36]. The PCR duplicates (duplicates introduced by PCR) were removed with MarkDuplicates in the Picard (Picard, RRID:SCR_006525) toolkit [58]. Single-nucleotide polymorphisms (SNPs) and small indels (≤10 bp) were identified with GATK (GATK, RRID:SCR_001876) version 3.7 [59] with default parameters and the addition of 3 extra thresholds to discard unreliable items during post-filter analysis, namely, (i) any 2 SNPs located within 5 bp from each other, (ii) any 2 indels located within 10 bp from each other, and (iii) any SNPs located within 5 bp from an indel. Finally, we identified 5,733,780 SNPs and 1,821,690 small indels in the farmed population and 5,719,771 SNPs and 1,820,404 small indels in the wild population. Similar distribution patterns of SNPs and indels were detected between the farmed and wild population (Supplementary Fig. S1) when 99% of the SNPs/indels were shared by both populations (Fig. 4A), reflecting that only ∼1% of the sequence variations were farmed population specific (FPS) or wild population specific (WPS). We focused on the differential variations located in the flanking regions and genic regions, between the farmed and wild populations, to identify candidate genes and causal mutations related to morphological traits. The software SnpEff version 2.0.5 [60] was applied to detect the effect of SNPs/indels by comparing the loci of SNPs/indels with those of protein-coding genes, which revealed that 59 genes carrying FPS SNPs/indels and 57 genes carrying WPS SNPs/indels underwent loss of translational start sites, gain or loss of stop codons, or variants in the acceptor/donor of splicing sites. Some variations were observed to cluster in farmed population (Fig. 4B), implicating a potential influence on morphological diversity. In addition, gene presence/absence variation might play a role in determining phenotypic traits [25, 51], which should be included in future resequencing analyses.

### Chromosome synteny and evolution in bivalves

**Figure 5:**
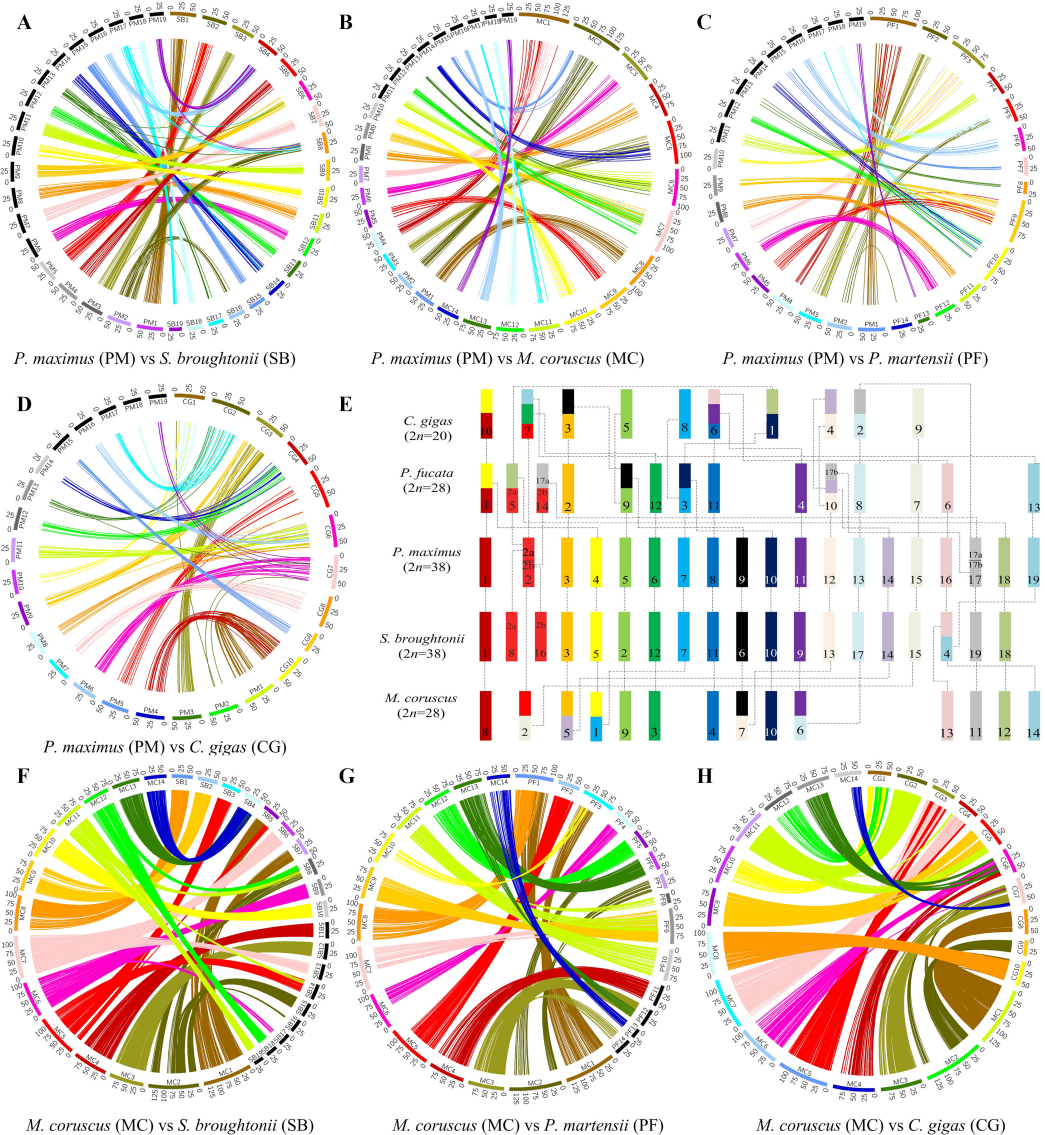
Chromosome synteny. **A**. Alignment of king scallop and blood clam chromosomes. **B**. Alignment of king scallop and hard-shelled mussel chromosomes. **C**. Alignment of king scallop and pearl oyster chromosomes. **D**. Alignment of king scallop and Pacific oyster chromosomes. **E**. Rearrangements between the chromosomes of king scallop and those of 4 other bivalve species. The king scallop chromosomes are represented by bars of different colors, and synteny and rearrangements in the chromosomes of the 4 other bivalves are indicated by different blocks, whose colors correspond to those of the reference king scallop chromosomes; the dashed lines indicate the corresponding evolutionary relationship. **F**. Alignment of hard-shelled mussel and blood clam chromosomes. **G**. Alignment of hard-shelled mussel and pearl oyster chromosomes. **H**. Alignment of hard-shelled mussel and Pacific oyster chromosomes. The king scallop linkage groups are labeled as PM 1–19, the blood clam chromosomes as SB 1–19, the hard-shelled mussel chromosomes as MC 1–14, the pearl oyster chromosomes as PF 1–14, and the Pacific oyster chromosomes as CG 1–10. Scale unit, Mb. A–D, F–H. The circularized blocks represent the chromosomes of the 5 bivalves. Aligned homologous genes are connected by ribbons, shown in different colors depending on their chromosome location.

To investigate the evolution of the mussel chromosomes, gene collinearity was constructed by aligning the genes of the king scallop *P. maximus* to the reference genomes of the blood clam *S. broughtonii*, the hard-shelled mussel *M. coruscus*, the pearl oyster *P. martensii*, and the Pacific oyster *C. gigas* using MCscan (version 0.8). The parameters of the MCscan alignment were set as -s, 7; k, 150; m, 250; e, 1e^−10^. We identified 404 scallop-vs-clam, 276 scallop-vs-mussel, 159 scallop–vs–pearl oyster, and 232 scallop–vs–Pacific oyster syntenic blocks, which included 10,055, 4,716, 3,636, and 5,009 genes of blood clam, hard-shelled mussel, pearl oyster, and Pacific oyster, respectively. The mean gene number per syntenic block was 21.4. King scallop and blood clam had the highest gene collinearity (Fig. 5A), consistent with their close phylogenetic relationship in the Bivalvia clade [61] (Fig. 3D). The chromosome synteny illustrated that large-scale rearrangements are rare between scallop and mussel but frequent between scallop and oysters (Fig. 5B–D), as exemplified by considerable structural variations between the scallop and the Pacific oyster genomes (Fig. 5D). The identified cross-chromosome rearrangements between the scallop and mussel genomes were different from those between the genomes of scallop and the 2 oyster species (Fig. 5B–E). The scallop linkage groups (PM) 1, 5, 6, 8, 10, 16, 17, 18, and 19 were syntenic to a single mussel chromosome (MC) 8, 9, 3, 4, 10, 13, 11, 12, and 14, respectively. PM 2 and 15 were aligned to the same reference, MC 2; similarly, PM 3 and 14 aligned to MC 5, PM 4 and 7 aligned to MC 1, PM 9 and 12 aligned to MC 7, and PM 11 and 13 aligned to MC 6. Comparatively, some additional chromosome rearrangements occurred between scallop and the 2 oyster species, especially the Pacific oyster. Both the Pacific oyster chromosome 9 and the pearl oyster chromosome 7 were predominantly syntenic to the scallop PM 15, suggesting that they might carry conserved genomic regions with the same origin (Fig. 5C–E). Among all the syntenic chromosomes, we did not observe any chromosome to be entirely conserved in all of the bivalve genomes. Intriguingly, almost all of the chromosome rearrangements between the mussel and the oyster genomes were different (Fig. 5E), implicating independent chromosome fusion events. In addition, the high gene collinearity between the hard-shelled mussel and another 3 bivalves, the Pacific oyster, blood clam, and pearl oyster, also reflected the satisfactory quality of the hard-shelled mussel assemblies (Fig. 5F–H). The identification of such diverse chromosome rearrangements suggested a complex evolutionary history of bivalve chromosomes.

### Metamorphosis-related transcriptome analysis

To profile gene expression during development and metamorphosis in hard-shelled mussels, RNA-seq analysis was conducted at 5 developmental stages: trochophore, D-veliger, umbo, pediveliger, and juvenile (PRJNA689932). The quantification of gene expression enabled the detection of 33,743 transcripts with TPMs > 0 at all stages (Supplementary Table S4). The limma statistical method was used to detect differentially expressed genes on the basis of linear models [62]. Using the trochophore as control, 5,795; 6,163; 9,308; and 7,486 upregulated genes [log_2_(fold-change) > 1 and adjusted *P* < 0.05] were identified in D-veliger, umbo, pediveliger, and juvenile larvae, respectively. Functional annotation indicated that these were mainly involved in “environmental information processing” (“signal transduction” and “signaling molecules and interaction”) and “cellular processes” (“transport and catabolism”), in agreement with the key role of signal transduction and the endocrine system in larval development [17].

**Figure 6:**
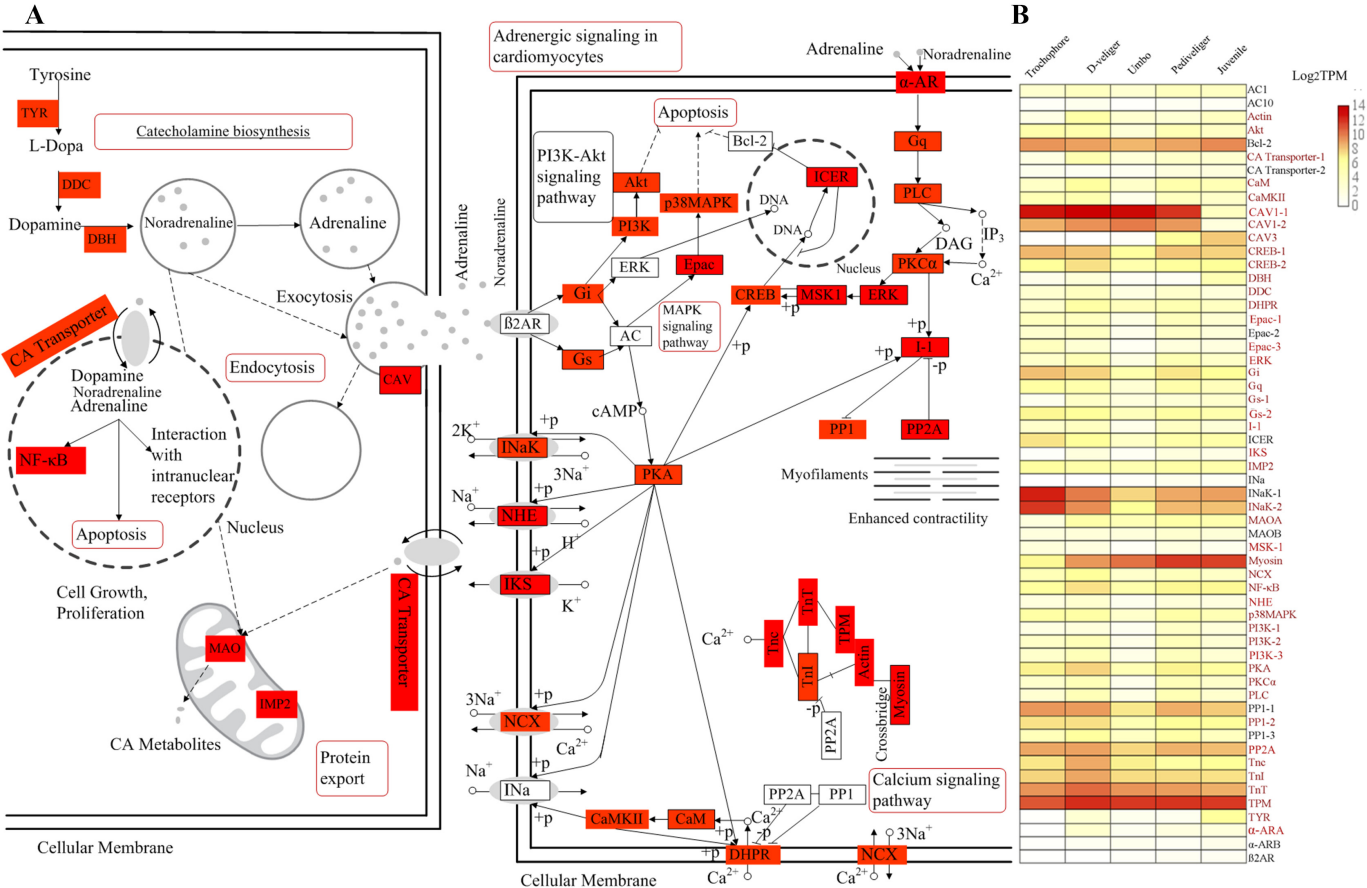
Spatial and temporal expression of genes involved in development and metamorphosis. **A**. Expression pattern of genes implied in the pathways of catecholamine biosynthesis and adrenergic signaling in cardiomyocytes, according to KEGG-based annotation. Red rectangles indicate upregulated genes during development, red rectangles with black edge indicate upregulated genes at Pediveliger stage and metamorphosis, and white rectangles denote genes that were identified during KEGG analysis but whose expression did not change. Red bubbles represent the most important pathways in which the upregulated genes are involved. **B**. Heat map showing the expression levels of all genes involved in the pathways of catecholamine biosynthesis and adrenergic signaling in cardiomyocytes across 5 developmental stages. These quantification results of gene expression are the averages of 3 replicate samples.

Because the ability to effectuate metamorphosis develops during the pediveliger stage [17], we investigate the 774 up-regulated genes during the transition from the umbo to the pediveliger stage. Functional annotation revealed that they were mainly used in a network of 6 related pathways: “adrenergic signaling in cardiomyocytes,” “calcium signaling pathway,” “MAPK signaling pathway,” “protein export,” “endocytosis,” and “catecholamine biosynthesis” (Fig. 6A), which have been reported to be involved in settlement and metamorphosis [18, 63]. The expression of most of the genes involved in these pathways increased during ≥1 period (Fig. 6B). Among them, 20 genes have been functionally identified to be associated with metamorphosis (Supplementary Table S5) and 26 up-regulated encompassing from the umbo to the pediveliger stages belonged to the categories “adrenergic signaling in cardiomyocytes,” “calcium signaling pathway,” and “catecholamine transport,” which was consistent with the findings of a recent proteome study on larval settlement and the metamorphosis of oysters [63–66]. Although some additional pathways, such as “phagosome” and “oxytocin signaling pathway,” are also detected, we did not analyze their function in detail because we still lack evidence on their involvement in metamorphosis. In summary, the analysis of the involved pathways revealed that biosynthesis, transport, and transduction of catecholamines might be critical for the completion of metamorphosis.

### Assembly assessment

The quality of the assembled genome was validated in terms of completeness, accuracy of the assemblies, and conservation of synteny. Alignment of Illumina reads against the reference genome revealed insert sizes of PE sequencing libraries of ∼300–350 bp and a mapping rate of >96.7%. We assayed the genome completeness using BUSCO (BUSCO, RRID:SCR_015008) v4.1.4 referencing metazoan and molluscan gene sets. In the metazoan dataset, the present assemblies have 89.4% complete (of which 1.0% were duplicated), 1.9% incomplete, and 8.7% missing BUSCOs, corresponding to a recovery of 91.3% of the entire BUSCO set. In the molluscan dataset, 85.5% complete (of which 1.3% were duplicated), 0.8% incomplete, and 13.7% missing BUSCOs were recorded, corresponding to 86.3% of the entire BUSCO set. Motifs with the characteristics of telomeric repeats were detected in 23 termini of the 13 chromosomes, suggesting the completeness of the assemblies (Supplementary Table S6). The accuracy of the genome assembly was evaluated by calling sequence variants through the alignment of Illumina sequencing data against the genome. Sequence alignment with BCFtools (version 1.3) [67] revealed 368,991 homozygous SNP loci, reflecting an error rate of <0.02% in the genome assemblies. In addition, the highly conserved synteny and the strict correspondence of chromosome fusion points and gene assignment identified between the hard-shelled mussel and king scallop genomes (Fig. 5B) were indicative of a high-quality assembly of the hard-shelled mussel genome because the king scallop genome is considered as the best-scaffolded genome available for bivalves [68].

## Conclusion

The chromosome-level assemblies of the hard-shelled mussel genome presented here are a well-assembled and annotated resource that could facilitate a wide range of research in mussels, bivalves, and molluscs. The outputs of this study shed light on chromosome evolution in bivalves, resulting in the regulation of the molecular pathways involved in larval metamorphosis. As one of the chromosome-level genome assemblies of bivalves, this genome dataset will serve as a high-quality genome platform for comparative genomics at the chromosome level.

## Data Availability

All of the raw Illumina and ONT read data underlying this article were deposited to NCBI SRA and the assembled genome was deposited to GenBank, under the accession No. PRJNA578350. Gene expression data in different developmental stages is released under the accession No. PRJNA689255. The corresponding genome sequences and read alignments (VCF files) are available in Figshare [69] and GigaDB [70].

## Additional Files

**Supplementary Table S1**. Repetitive sequences in the hard-shelled mussel genome

**Supplementary Table S2**. Overview of the predicted non-coding RNAs

**Supplementary Table S3**. Bidirectional BLASTp between the previously published gene models of the hard-shelled mussel and the predicted gene models in this study

**Supplementary Table S4**. Gene expression profiles during 5 developmental stages

**Supplementary Table S5**. Genes involved in the pathways of catecholamine biosynthesis and adrenergic signaling in the cardiomyocytes were reported to affect metamorphosis

**Supplementary Table S6**. Information on the motifs with the characteristic of telomeric repeats

**Supplementary Figure S1**. Circles showing genome-wide SNPs and indels from the farmed and wild populations. From the outer to the inner circle: first circle, marker distribution across 14 pseudochromosomes at a megabase scale; green circle, SNP density across the whole genome; red circle, indel density.

## Abbreviations

α-ARA: alpha-1A adrenergic receptor-like; α-ARB: adrenergic receptor alpha-1B; β2AR: adrenergic receptor beta-2; AC1: adenylate cyclase 1; AC10: adenylate cyclase 10; Akt: RAC serine/threonine-protein kinase; BLAST: Basic Local Alignment Search Tool; bp: base pairs; BUSCO: Benchmarking Universal Single-Copy Orthologs; BWA: Burrows-Wheeler Aligner; CaM: calmodulin; CaMKII: calcium/calmodulin-dependent protein kinase (CaM kinase) II; CAV1: caveolin 1; CAV3: caveolin 3; CREB: cyclic AMP-responsive element-binding protein; DBH: dopamine beta-monooxygenase; DDC: aromatic-L-amino-acid decarboxylase; DHPR: voltage-dependent calcium channel gamma-1; Epac: Rap guanine nucleotide exchange factor; ERK: mitogen-activated protein kinase 1/3; FPS: farmed population specific; GATK: Genome Analysis Tool Kit; Gb: gigabase pairs; GC: guanine-cytosine; Gi: guanine nucleotide-binding protein G(i) subunit alpha; GO: Gene Ontology; Gq: guanine nucleotide-binding protein G(q) subunit alpha; Gs: guanine nucleotide-binding protein G(s) subunit alpha; Hi-C: high-throughput chromosome conformation capture; ICER: cAMP response element modulator; IKS: potassium voltage-gated channel KQT-like subfamily member 1; IMP2: mitochondrial inner membrane protease subunit 2; INaK: sodium/potassium-transporting ATPase subunit alpha; kb: kilobase pairs; KEGG: Kyoto Encyclopedia of Genes and Genomes; MAOA: monoamine oxidase A; MAOB: monoamine oxidase B; Mb: megabase pairs; MSK1: ribosomal protein S6 kinase alpha-5; NCBI: National Center for Biotechnology Information; NCX: solute carrier family 8 (sodium/calcium exchanger); NF-κB: nuclear factor NF-kappa-B p105 subunit; NHE: solute carrier family 9 (sodium/hydrogen exchanger); ONT: Oxford Nanopore Technologies; p38MAPK: p38 MAP kinase; PASA: Program to Assemble Spliced Alignments; PE: paired end; PI3K: phosphatidylinositol-4,5-bisphosphate 3-kinase catalytic subunit alpha/beta/delta; PKA: protein kinase A; PKCα: classical protein kinase C alpha type; PLC: phosphatidylinositol phospholipase C; PP1: serine/threonine-protein phosphatase PP1 catalytic subunit; RAxML: Randomized Axelerated Maximum Likelihood; RNA-Seq: RNA sequencing; SNP: single-nucleotide polymorphism; SRA: Sequence Read Archive; SSR: simple sequence repeat; TnI: Troponin I; TPMs: transcripts per million; TPM: tropomyosin; tRNA: transfer RNA; TYR: tyrosinase; WPS: wild population specific.

## Competing Interests

The authors declare that they have no competing interests.

## Funding

This study was supported by the National Key Research and Development Program of China (2018YFD0900601), Key Program for International Science and Technology Cooperation Projects of Ministry of Science and Technology of China (No. 2018YFD0900101), Key Special Project for Introduced Talents Team of Southern Marine Science and Engineering Guangdong Laboratory (Guangzhou) (GML2019ZD0402), the National Natural Science Foundation of China (No. 41,876,159, No. 41,606,147, No. 31,802,321), Program of Shanghai Academic Research Leader (20XD1421800), "Science and Technology Innovation Action Plan" The Belt and Road International Joint Laboratory Construction Projects (590750500), the Shanghai Sailing Program (18YF1410000), China Postdoctoral Science Foundation (2019M6614770), and Program for study on genetic resources, environment and strategy of mussel culture in coast of Gouqi Island offshore.

## Authors' Contributions

J.L.Y., Y.L., and X.L. designed and supervised the study. K.C., J.K.X, Y.T.Z., and Y.F.L. collected the samples and extracted the genomic DNA and RNA. Y.L., J.L., and D.D.F. performed genome assembly and bioinformatics analysis. J.L.Y., D.D.F., X.L., J.L., and Y.L. wrote the original manuscript. All authors reviewed the manuscript.

## Supplementary Material

giab024_GIGA-D-20-00287_Original_Submission

giab024_GIGA-D-20-00287_Revision_1

giab024_GIGA-D-20-00287_Revision_2

giab024_Response_to_Reviewer_Comments_Original_Submission

giab024_Response_to_Reviewer_Comments_Revision_1

giab024_Reviewer_1_Report_Original_Submission Marco Gerdol -- 10/19/2020 Reviewed

giab024_Reviewer_1_Report_Revision_1 Marco Gerdol -- 2/2/2021 Reviewed

giab024_Reviewer_2_Report_Original_Submission Nathan Kenny -- 10/24/2020 Reviewed

giab024_Reviewer_2_Report_Revision_1 Nathan Kenny -- 1/30/2021 Reviewed

giab024_Reviewer_3_Report_Original_Submission Yi-Jyun Luo -- 10/26/2020 Reviewed

giab024_Reviewer_3_Report_Revision_1 Yi-Jyun Luo -- 2/18/2021 Reviewed

giab024_Supplemental_Files

## References

[1] FAO. The State of World Fisheries and Aquaculture. Rome: FAO; 2018.

[2] Amini S, Kolle S, Petrone L, et al. Preventing mussel adhesion using lubricant-infused materials. Science. 2017;357(6352):668–673. 28818939 10.1126/science.aai8977

[3] Yang JL, Li YF, Guo XP, et al. The effect of carbon nanotubes and titanium dioxide incorporated in PDMS on biofilm community composition and subsequent mussel plantigrade settlement. Biofouling. 2016;32(7):763–777. 27348759 10.1080/08927014.2016.1197210

[4] Yang JL, Shen PJ, Liang X, et al. Larval settlement and metamorphosis of the mussel *Mytilus coruscus* in response to monospecific bacterial biofilms. Biofouling. 2013;29(3):247–259. 23452123 10.1080/08927014.2013.764412

[5] Liang X, Peng LH, Zhang S, et al. Polyurethane, epoxy resin and polydimethylsiloxane altered biofilm formation and mussel settlement. Chemosphere. 2019;218:599–608. 30502698 10.1016/j.chemosphere.2018.11.120

[6] Odonnell MJ, George MN, Carrington E. Mussel byssus attachment weakened by ocean acidification. Nat Clim Chang. 2013;3(6):587–590.

[7] Ramesh K, Hu MY, Thomsen J, et al. Mussel larvae modify calcifying fluid carbonate chemistry to promote calcification. Nat Commun. 2017;8(1):1709. 29167466 10.1038/s41467-017-01806-8 PMC5700083

[8] Thomsen J, Stapp L, Haynert K, et al. Naturally acidified habitat selects for ocean acidification–tolerant mussels. Sci Adv. 2017;3(4):e1602411. 28508039 10.1126/sciadv.1602411 PMC5406135

[9] Bitter MC, Kapsenberg L, Gattuso J, et al. Standing genetic variation fuels rapid adaptation to ocean acidification. Nat Commun. 2019;10(1):5821. 31862880 10.1038/s41467-019-13767-1 PMC6925106

[10] Briand J . Marine antifouling laboratory bioassays: an overview of their diversity. Biofouling. 2009;25(4):297–311. 19191083 10.1080/08927010902745316

[11] Petrone L, Kumar A, Sutanto CN, et al. Mussel adhesion is dictated by time-regulated secretion and molecular conformation of mussel adhesive proteins. Nat Commun. 2015;6(1):8737. 26508080 10.1038/ncomms9737 PMC4640085

[12] Zeng ZS, Guo XP, Cai XS, et al. Pyomelanin from *Pseudoalteromonas lipolytica* reduces biofouling. Microb Biotechnol. 2017;10(6):1718–1731. 28834245 10.1111/1751-7915.12773 PMC5658579

[13] Murgarella M, Puiu D, Novoa B, et al. A first insight into the genome of the filter-feeder mussel *Mytilus galloprovincialis*. PLoS One. 2016;11(3):e0151561. 26977809 10.1371/journal.pone.0151561 PMC4792442

[14] Sun J, Zhang Y, Xu T, et al. Adaptation to deep-sea chemosynthetic environments as revealed by mussel genomes. Nat Ecol Evol. 2017;1(5):121. 28812709 10.1038/s41559-017-0121

[15] Hadfield MG, Paul VG. Natural chemical cues for settlement and metamorphosis of marine invertebrate larvae. In: McClintock JB, Baker JB, eds. Marine Chemical Ecology. Boca Raton, FL: CRC; 2001.

[16] Dobretsov S, Rittschof D. Love at first taste: induction of larval settlement by marine microbes. Int J Mol Sci. 2020;21(3):731. 31979128 10.3390/ijms21030731 PMC7036896

[17] Hadfield MG . Biofilms and marine invertebrate larvae: what bacteria produce that larvae use to choose settlement sites. Annu Rev Mar Sci. 2011;3(1):453–470. 10.1146/annurev-marine-120709-142753 21329213

[18] Shikuma NJ, Antoshechkin I, Medeiros JM, et al. Stepwise metamorphosis of the tubeworm *Hydroides elegans* is mediated by a bacterial inducer and MAPK signaling. Proc Natl Acad Sci U S A. 2016;113(36):10097–10102. 27551098 10.1073/pnas.1603142113 PMC5018781

[19] Shikuma NJ, Pilhofer M, Weiss GL, et al. Marine tubeworm metamorphosis induced by arrays of bacterial phage tail–like structures. Science. 2014;343(6170):529–533. 24407482 10.1126/science.1246794 PMC4949041

[20] Kulikova VA, Lyashenko SA, Kolotukhina NK. Seasonal and interannual dynamics of larval abundance of *Mytilus coruscus* Gould, 1861 (Bivalvia: Mytilidae) in Amursky Bay (Peter the Great Bay, Sea of Japan). Russ J Mar Biol. 2011;37(5):342–347.

[21] Li YF, Liu YZ, Chen YW, et al. Two toll-like receptors identified in the mantle of *Mytilus coruscus* are abundant in haemocytes. Fish Shellfish Immunol. 2019;90:134–140. 31055019 10.1016/j.fsi.2019.05.001

[22] Liang X, Zhang XK, Peng LH, et al. The flagellar gene regulates biofilm formation and mussel larval settlement and metamorphosis. Int J Mol Sci. 2020;21(3):710. 31973189 10.3390/ijms21030710 PMC7036800

[23] Yang JL, Li SH, Li YF, et al. Effects of neuroactive compounds, ions and organic solvents on larval metamorphosis of the mussel *Mytilus coruscus*. Aquaculture. 2013; 396–399:106–112.

[24] Li RH, Zhang WJ, Lu JK, et al. The whole-genome sequencing and hybrid assembly of *Mytilus coruscus*. Front Genet. 2020;11:440. 32457802 10.3389/fgene.2020.00440 PMC7227121

[25] Gerdol M, Moreira R, Cruz F, et al. Massive gene presence-absence variation shapes an open pan-genome in the Mediterranean mussel. Genome Biol. 2020;21(1):275. 33168033 10.1186/s13059-020-02180-3 PMC7653742

[26] Li YL, Sun XQ, Hu XL, et al. Scallop genome reveals molecular adaptations to semi-sessile life and neurotoxins. Nat Commun. 2017;8(1):1721. 29167427 10.1038/s41467-017-01927-0 PMC5700196

[27] Wang S, Zhang J, Jiao W, et al. Scallop genome provides insights into evolution of bilaterian karyotype and development. Nat Ecol Evol. 2017;1:120. 28812685 10.1038/s41559-017-0120 PMC10970998

[28] Sokolov EP . An improved method for DNA isolation from mucopolysaccharide-rich molluscan tissues. J Mollusc Stud. 2000;66(4):573–575.

[29] Van Berkum NL, Lieberman-Aiden E, Williams L, et al. Hi-C: A method to study the three-dimensional architecture of genomes. J Vis Exp. 2010;39:e1869. 10.3791/1869 PMC3149993 20461051

[30] Marçais G, Kingsford C. A fast, lock-free approach for efficient parallel counting of occurrences of *k*-mers. Bioinformatics. 2011;27(6):764–770. 21217122 10.1093/bioinformatics/btr011 PMC3051319

[31] Vurture GW, Sedlazeck FJ, Nattestad M, et al. GenomeScope: Fast reference-free genome profiling from short reads. Bioinformatics. 2017;33(14):2202–2204. 28369201 10.1093/bioinformatics/btx153 PMC5870704

[32] Ieyama H, Kameoka O, Tan T, et al. Chromosomes and nuclear DNA contents of some species in Mytilidae. Venus. 1994;53:327–331.

[33] Koren S, Walenz BP, Berlin K, et al. Canu: Scalable and accurate long-read assembly via adaptive *k*-mer weighting and repeat separation. Genome Res. 2017;27(5):722–736. 28298431 10.1101/gr.215087.116 PMC5411767

[34] Vaser R, Sović I, Nagarajan N, et al. Fast and accurate de novo genome assembly from long uncorrected reads. Genome Res. 2017;27(5):737–746. 28100585 10.1101/gr.214270.116 PMC5411768

[35] Walker BJ, Abeel T, Shea T, et al. Pilon: An integrated tool for comprehensive microbial variant detection and genome assembly improvement. PLoS One. 2014;9(11):e112963. 25409509 10.1371/journal.pone.0112963 PMC4237348

[36] Li H, Durbin R. Fast and accurate short read alignment with Burrows–Wheeler transform. Bioinformatics. 2009;25(14):1754–1760. 19451168 10.1093/bioinformatics/btp324 PMC2705234

[37] Burton JN, Adey A, Patwardhan RP, et al. Chromosome-scale scaffolding of de novo genome assemblies based on chromatin interactions. Nat Biotechnol. 2013;31(12):1119–1125. 24185095 10.1038/nbt.2727 PMC4117202

[38] Zhuang BX . A preliminary study on the chromosome of marine bivalve, *Mytilus coruscus*. Zool Res. 1984;5(3):57–60.

[39] Smit A, Hubley R. RepeatModeler Open-1.0. 2008. http://www.repeatmasker.org/RepeatModeler/.Accessed 7 April 2021.

[40] Smit A, Hubley R, Green P. RepeatMasker Open-4.0. 2015. http://www.repeatmasker.org/. Accessed 7 April 2021.

[41] Nawrocki EP, Eddy SR. Infernal 1.1: 100-fold faster RNA homology searches. Bioinformatics. 2013;29(22):2933–2935. 24008419 10.1093/bioinformatics/btt509 PMC3810854

[42] Korf I . Gene finding in novel genomes. BMC Bioinformatics. 2004;5(1):59. 15144565 10.1186/1471-2105-5-59 PMC421630

[43] Slater GSC, Birney E. Automated generation of heuristics for biological sequence comparison. BMC Bioinformatics. 2005;6(1):31. 15713233 10.1186/1471-2105-6-31 PMC553969

[44] Grabherr MG, Haas BJ, Yassour M, et al. Trinity: Reconstructing a full-length transcriptome without a genome from RNA-Seq data. Nat Biotechnol. 2011;29(7):644. 21572440 10.1038/nbt.1883 PMC3571712

[45] Trapnell C, Williams BA, Pertea G, et al. Transcript assembly and quantification by RNA-Seq reveals unannotated transcripts and isoform switching during cell differentiation. Nat Biotechnol. 2010;28(5):511. 20436464 10.1038/nbt.1621 PMC3146043

[46] Haas BJ, Salzberg SL, Zhu W, et al. Automated eukaryotic gene structure annotation using EVidenceModeler and the Program to Assemble Spliced Alignments. Genome Biol. 2008;9(1):R7. 18190707 10.1186/gb-2008-9-1-r7 PMC2395244

[47] Zdobnov EM, Apweiler R. InterProScan–an integration platform for the signature-recognition methods in InterPro. Bioinformatics. 2001;17(9):847–848. 11590104 10.1093/bioinformatics/17.9.847

[48] Ashburner M, Ball CA, Blake JA, et al. Gene ontology: Tool for the unification of biology. Nat Genet. 2000;25(1):25. 10802651 10.1038/75556 PMC3037419

[49] Kanehisa M, Goto S, Kawashima S, et al. The KEGG resource for deciphering the genome. Nucleic Acids Res. 2004;32(90001):277–280. 10.1093/nar/gkh063 PMC308797 14681412

[50] Boeckmann B, Bairoch A, Apweiler R, et al. The SWISS-PROT protein knowledgebase and its supplement TrEMBL in 2003. Nucleic Acids Res. 2003;31(1):365–370. 12520024 10.1093/nar/gkg095 PMC165542

[51] Calcino AD, Kenny NJ, Gerdol M. Single individual structural variant detection uncovers widespread hemizygosity in molluscs. bioRxiv. 2020, 10.1101/2020.09.15.298695. PMC8059565 33813894

[52] Li L, Stoeckert CJ, Roos DS. OrthoMCL: Identification of ortholog groups for eukaryotic genomes. Genome Res. 2003;13(9):2178–2189. 12952885 10.1101/gr.1224503 PMC403725

[53] Edgar RC . MUSCLE: Multiple sequence alignment with high accuracy and high throughput. Nucleic Acids Res. 2004;32(5):1792–1797. 15034147 10.1093/nar/gkh340 PMC390337

[54] Stamatakis A . RAxML-VI-HPC: Maximum likelihood-based phylogenetic analyses with thousands of taxa and mixed models. Bioinformatics. 2006;22(21):2688–2690. 16928733 10.1093/bioinformatics/btl446

[55] Kumar S, Stecher G, Suleski M, et al. TimeTree: A resource for timelines, timetrees, and divergence times. Mol Biol Evol. 2017;34(7):1812–1819. 28387841 10.1093/molbev/msx116

[56] Yang Z . PAML: A program package for phylogenetic analysis by maximum likelihood. Comput Appl Biosci. 1997;13:555–556. 9367129 10.1093/bioinformatics/13.5.555

[57] Rambaut A . FigTree, a graphical viewer of phylogenetic trees. 2007;. http://tree.bio.ed.ac.uk/software/figtree/. http://tree.bio.ed.ac.uk/software/figtree/.http://tree.bio.ed.ac.uk/software/figtree/. Accessed 7 April 2021.

[58] Broad Institute. PicardToolkit. 2019. http://broadinstitute.github.io/picard/. Accessed 7 April 2021.

[59] Mckenna A, Hanna M, Banks E, et al. The Genome Analysis Toolkit: A MapReduce framework for analyzing next-generation DNA sequencing data. Genome Res. 2010;20(9):1297–1303. 20644199 10.1101/gr.107524.110 PMC2928508

[60] Cingolani P, Platts AE, Wang LL, et al. A program for annotating and predicting the effects of single nucleotide polymorphisms, SnpEff: SNPs in the genome of *Drosophila melanogaster* strain w1118. Fly. 2012;6(2):80–92. 22728672 10.4161/fly.19695 PMC3679285

[61] Liu FY, Li YL, Yu HW, et al. MolluscDB: An integrated functional and evolutionary genomics database for the hyper-diverse animal phylum Mollusca. Nucleic Acids Res. 2021;49(D1):D988–D997. 33219670 10.1093/nar/gkaa918 PMC7779068

[62] Smyth GK, Ritchie M, Thorne N, et al. LIMMA: Linear models for microarray data. In Bioinformatics and Computational Biology Solutions Using R and Bioconductor. Statistics for Biology and Health; 2005.

[63] Di G, Xiao X, Tong MH, et al. Proteome of larval metamorphosis induced by epinephrine in the Fujian oyster *Crassostrea angulata*. BMC Genomics. 2020;21(1):675. 32993483 10.1186/s12864-020-07066-z PMC7525975

[64] Eisenhofer G, Tian H, Holmes C, et al. Tyrosinase: A developmentally specific major determinant of peripheral dopamine. FASEB J. 2003;17(10):1248–1255. 12832289 10.1096/fj.02-0736com

[65] Bonar DB, Coon SL, Walch M, et al. Control of oyster settlement and metamorphosis by endogenous and exogenous chemical cues. Bull Mar Sci. 1990;46:484–498.

[66] Joyce A, Vogeler S. Molluscan bivalve settlement and metamorphosis: Neuroendocrine inducers and morphogenetic responses. Aquaculture. 2018;487:64–82.

[67] Narasimhan VM, Danecek P, Scally A, et al. BCFtools/RoH: A hidden Markov model approach for detecting autozygosity from next-generation sequencing data. Bioinformatics. 2016;32(11):1749–1751. 26826718 10.1093/bioinformatics/btw044 PMC4892413

[68] Kenny NJ, Mccarthy S, Dudchenko O, et al. The gene-rich genome of the scallop *Pecten maximus*. Gigascience. 2020;9(5):giaa037. 32352532 10.1093/gigascience/giaa037 PMC7191990

[69] Feng DD . The hard-shelled mussel *Mytilus coruscus -* gene models, annotations and related files of the whole genome. Figshare. 2020, 10.6084/m9.figshare.10259618.v1.

[70] Yang J, Feng D, Liu J, et al. Supporting data for “Chromosome-level genome assembly of the hard-shelled mussel *Mytilus coruscus*, a widely distributed species from the temperate areas of East Asia.”. GigaScience Database. 2021. 10.5524/100874. PMC8063583 33891010

