# Supplementary material for: Chromosome-level genome assembly of the hard-shelled mussel Mytilus coruscus, a widely distributed species from the temperate areas of East Asia
Source: Gigascience. 2021 Apr 23;10(4):giab024. doi: 10.1093/gigascience/giab024 (PMC8063583; doi:10.1093/gigascience/giab024)
Supplement: giab024_GIGA-D-20-00287_Original_Submission [file giab024_giga-d-20-00287_original_submission.pdf]

GigaScience

A chromosome-level genome assembly of the hard-shelled mussel Mytilus coruscus widely distributed in temperate seas of East Asia

--Manuscript Draft--

**Manuscript Number:**	GIGA-D-20-00287
**Full Title:**	A chromosome-level genome assembly of the hard-shelled mussel Mytilus coruscus widely distributed in temperate seas of East Asia
**Article Type:**	Data Note
**Funding Information:**	the Natioanl Key Research and Development Program of China (2018YFD0900601) — Doctoral Yi-Feng Li<br>International Science and Technology Cooperation Programme (CN) (2018YFD0900101) — Professor Ying Lu<br>the National Natural Science Foundation of China (41876159) — Professor Jin-Long Yang<br>the National Natural Science Foundation of China (41606147) — Doctoral Xiao Liang<br>the National Natural Science Foundation of China (31802321) — Doctoral Yi-Feng Li<br>Guangdong Key Laboratory of Fermentation and Enzyme Engineering (CN) (GML2019ZD0402) — Professor Jin-Long Yang<br>the Shanghai Sailing Program (18YF1410000) — Doctoral Yi-Feng Li<br>Program for study on genetic resources, environment and strategy of mussel culture in coast of Gouqi Island offshore — Professor Jin-Long Yang

Funding source	Recipient
the Natioanl Key Research and Development Program of China (2018YFD0900601)	Doctoral Yi-Feng Li
International Science and Technology Cooperation Programme (CN) (2018YFD0900101)	Professor Ying Lu
the National Natural Science Foundation of China (41876159)	Professor Jin-Long Yang
the National Natural Science Foundation of China (41606147)	Doctoral Xiao Liang
the National Natural Science Foundation of China (31802321)	Doctoral Yi-Feng Li
Guangdong Key Laboratory of Fermentation and Enzyme Engineering (CN) (GML2019ZD0402)	Professor Jin-Long Yang
the Shanghai Sailing Program (18YF1410000)	Doctoral Yi-Feng Li
Program for study on genetic resources, environment and strategy of mussel culture in coast of Gouqi Island offshore	Professor Jin-Long Yang

Abstract:

Background

The hard-shelled mussel (Mytilus coruscus) is widely distributed in temperate seas of East Asia, as well as an important commercial bivalve in China. A chromosome-level genome information for this species will not only contribute to the development of the hard-shelled mussel genetic breeding but also to the studies of larval ecology, climate change biology, marine biology, aquaculture, biofouling and antifouling.

Findings

We applied a combined strategy of Illumina sequencing, Oxford Nanopore and high-through chromosome conformation capture technologies to construct a chromosome-level genome of the hard-shelled mussel, which has a total length of 1.57 Gb and a contig N50 length of 1.49 Mb. Approximately 90.9% of the assemblies are anchored on 14 linkage groups. The completeness test exhibits that the genome carries 91.9% of core metazoan orthologs. Gene modeling annotated 37,478 protein-coding genes and 26,917 non-coding RNA loci. The phylogenetic analysis illustrated the closest relationship between M. coruscus and the clade of Modiolus philippinarum and Bathymodiolus platifron . Deep whole genome re-sequencing reveals the sequence variations might be associated with phenotype diversity between farmed and wild populations. A conserved chromosome synteny was observed between hard-shelled mussel and king scallop, speculating their sharing same origins in evolution. Transcriptome profile revealed that the catecholamine biosynthesis and adrenergic signaling in cardiomyocytes pathways were involved in metamorphosis development.

Conclusions

The chromosome-level genome assembly of marine mussel will provide novel insights into mussel genome evolution and serve as a fundamental platform for studying on

Powered by Editorial Manager® and ProduXion Manager® from Aries Systems Corporation

	planktonic-sessile transition, genetic diversity and genomic breeding.
**Corresponding Author:**	Ying Lu<br>Shanghai Ocean University<br>Shanghai, CHINA
**Corresponding Author Secondary Information:**	
**Corresponding Author's Institution:**	Shanghai Ocean University
**Corresponding Author's Secondary Institution:**	
**First Author:**	Jin-Long Yang
**First Author Secondary Information:**	
**Order of Authors:**	Jin-Long Yang
	Dan-Dan Feng
	Jie Liu
	Jia-Kang Xu
	Ke Chen
	Yi-Feng Li
	You-Ting Zhu
	Xiao Liang
	Ying Lu
**Order of Authors Secondary Information:**	
**Additional Information:**	
**Question**	**Response**
Are you submitting this manuscript to a special series or article collection?	No
**Experimental design and statistics**<br><br>Full details of the experimental design and statistical methods used should be given in the Methods section, as detailed in our Minimum Standards Reporting Checklist. Information essential to interpreting the data presented should be made available in the figure legends.<br><br>Have you included all the information requested in your manuscript?	Yes
**Resources**<br><br>A description of all resources used, including antibodies, cell lines, animals and software tools, with enough	Yes

information to allow them to be uniquely identified, should be included in the Methods section. Authors are strongly encouraged to cite Research Resource Identifiers (RRIDs) for antibodies, model organisms and tools, where possible.<br><br>Have you included the information requested as detailed in our Minimum Standards Reporting Checklist?	
**Availability of data and materials**<br><br>All datasets and code on which the conclusions of the paper rely must be either included in your submission or deposited in publicly available repositories (where available and ethically appropriate), referencing such data using a unique identifier in the references and in the “Availability of Data and Materials” section of your manuscript.<br><br>Have you have met the above requirement as detailed in our Minimum Standards Reporting Checklist?	Yes

Manuscript Click here to access/download;Manuscript;Maintext0923_NoReference.doc

A chromosome-level genome assembly of the hard-shelled mussel *Mytilus coruscus* widely distributed in temperate seas of East Asia

Jin-Long Yang[1,2,3,4,*], Dan-Dan Feng[1,2,4], Jie Liu[1,2,4], Jia-Kang Xu[1,2], Ke Chen[1,2], Yi-Feng Li[1,2], You-Ting Zhu[1,2], Xiao Liang[1,2], Ying Lu[1,2,*]

[1] International Research Center for Marine Biosciences, Ministry of Science and Technology, Shanghai Ocean University, Shanghai, China

[2] Key Laboratory of Exploration and Utilization of Aquatic Genetic Resources, Ministry of Education, Shanghai Ocean University, Shanghai, China

[3] Southern Marine Science and Engineering Guangdong Laboratory, Guangzhou, China

[4] These authors contributed equally: Jin-Long Yang, Dan-Dan Feng, Jie Liu.

* Corresponding author. E-mail: jlyang@shou.edu.cn, yinglu@shou.edu.cn

Tel: + 86-21-61900403; Fax: + 86-21-61900405

Abstract

Background: The hard-shelled mussel (*Mytilus coruscus*) is widely distributed in temperate seas of East Asia, as well as an important commercial bivalve in China. A chromosome-level genome information for this species will not only contribute to the development of the hard-shelled mussel genetic breeding but also to the studies of larval ecology, climate change biology, marine biology, aquaculture, biofouling and antifouling. **Findings:** We applied a combined strategy of Illumina sequencing, Oxford Nanopore and high-through chromosome conformation capture technologies to construct a chromosome-level genome of the hard-shelled mussel, which has a total length of 1.57 Gb and a contig N50 length of 1.49 Mb. Approximately 90.9% of the assemblies are anchored on 14 linkage groups. The completeness test exhibits that the genome carries 91.9% of core metazoan orthologs. Gene modeling annotated 37,478 protein-coding genes and 26,917 non-coding RNA loci. The phylogenetic analysis illustrated the closest relationship between *M. coruscus* and the clade of *Modiolus philippinarum* and *Bathymodiolus platifron*. Deep whole genome re-sequencing reveals the sequence variations might be associated with phenotype diversity between farmed and wild populations. A conserved chromosome synteny was observed between hard-shelled mussel and king scallop, speculating their sharing same origins in evolution. Transcriptome profile revealed that the catecholamine biosynthesis and adrenergic signaling in cardiomyocytes pathways were involved in metamorphosis development. **Conclusions:** The chromosome-level genome assembly of marine mussel will provide novel insights into mussel genome evolution and serve as a fundamental platform for

studying on planktonic-sessile transition, genetic diversity and genomic breeding.

Keywords: *Mytilus coruscus*, genome sequencing, Hi-C, chromosome, metamorphosis

Context

Marine mussels, which belong to the phylum Mollusc, settle on most immersed surfaces of substrata and play a crucial role in marine ecosystems. As healthy and sustainable food items, these sea mussels are beneficial for humans due to the high economic value for fishery and aquaculture, constituting more than 8% of mollusc aquaculture production [1]. Simultaneously, mussels are also known as typical macrofouling organisms that result in detrimental economic and ecological consequences for the maritime and aquaculture industries [2-4]. Mussels have been used as model organisms for adaptation to climate change, integrative ecomechanics, biomaterials, larval ecology, settlement and metamorphosis, adhesion, bacteria-host interaction, biofouling and antifouling studies [5-12]. Although their significance in biology, ecology and economy, whole genome information of marine mussels is limited [13, 14] and lack of these related knowledge postpones our understanding molecular basis on the adaption, evolution, breeding, genetic manipulation, bacteria-host interaction, settlement mechanism.

As with a dozen of marine invertebrates, marine mussels also possess a free-swimming larval phase. After this stage, these minute larvae will settle on the substrata and finish metamorphosis transition, accompanied with dramatic remodeling of anatomy [4, 15]. Multiple physicochemical stimuli play critical roles in the process of

larval settlement and metamorphosis [15-17]. Thus, understanding of larvae-juvenile transition process is still a keystone question in marine biology, larval ecology, aquaculture, biofouling and antifouling [4, 15, 18, 19]. The finding that chemical cues from bacterial biofilms trigger settlement and metamorphosis is universal in metazoan [15, 16, 18].

The hard-shelled mussel (*Mytilus coruscus* Gould 1861, NCBI Taxonomy ID: 42192**, Fig. 1**) mainly inhabits the temperate along the coastal waters of China, Japan, Korea and Far East of Russia, covering from East China Sea to Sea of Japan [20]. In China, the hard-shelled mussel is an important commercial bivalve as well as a typical macrofouling organism. As a sessile marine bivalve, the hard-shelled mussel need adapt to hostile and complex environments of intertidal regions. Most of studies focused on the planktonic-sessile transition mechanism of receptor and biofilm regulation, host-bacteria interaction, aquaculture and biofouling and antifouling studies in this species [3-5, 12, 21-23]. Up to date, chromosome level genome in the genus *Mytilus* is absent, although a low quality draft genome of *M. coruscus* has been reported [24], as well as *M. galloprovincialis* [13]. Lacking whole-genome information has hindered the development of the hard-shelled mussel genetic breeding, larval ecology, climate change biology, marine biology, aquaculture, biofouling and antifouling studies.

In this study, we reported a chromosome-level assembly of the hard-shelled mussel genome using a combined strategy of Illumina sequencing, Oxford Nanopore Technologies (ONT) and high-through chromosome conformation capture (Hi-C) technologies. We validated the genome assemblies by means of chromosome synteny

analysis, comparing with the most published chromosome-level molluscan genomes. The larvaes at five early developmental stages were put into the RNA-Seq analysis to profile gene expressions during the metamorphosis development. When the chromosome-level genome data sets are accessible [25, 26], it will facilitate the study of comparative genomics on chromosome rearrangements crossing different species.

Methods

Sample information and collection

The wild individuals for genome sequencing were collected from the coast of Shengsi, Zhejiang province, where is the central coast of Chinese mainland and one of the original and main breeding areas of the hard-shelled mussel in China. The farmed and wild adults were also collected from the coast of Shengsi (122.77′E 30.73′N) and (122.74′E 30.71′N), respectively (**Fig. 1**). A female wild adult with mature ovary was dissected for the adductor muscle to isolate high molecular weight genomic DNA for sequencing of reference genome. The DNAs extracted from the farmed and wild populations (10 individuals each population) were pooled for the genome re-sequencing, respectively. The adductor muscle, mantle, gill, digestive gland, hemocyte, labial palp, female gonad, male gonad, foot, gut tissues were dissected from fresh samples for transcriptome sequencing to be assistant with prediction of protein-coding genes. The larvas, including trochophore, D-veliger, umbo, pediveliger and juvenile, were collected for transcriptome sequencing to profile differentially expressed genes (DEGs).

Isolation of genomic DNA and RNA

Genomic DNA was extracted from fresh adductor muscle tissue using SDS extraction method, which was used for sequencing on ONT PromethION platform (Oxford Nanopore Technologies, UK). Using the TIANamp Marine Animals DNA Kit (Tiangen, China), the DNA for whole genome re-sequencing was extracted from muscle of five female and five male individuals in each population, respectively. Using RNAiso Plus kit (TaKaRa, Japan), total RNA were extracted from ten different tissues of five female and five male individuals in each population, respectively, as well as the larvaes at five developmental stages. Fresh muscle cells were crosslinked with formaldehyde and the digestion, marking of DNA ends and blunt-end ligation were performed as in the previous study [27], the purified DNA was used for Hi-C.

Genome sequencing with different technologies.

A combined sequencing strategy was applied to the hard-shelled mussel genome (**Fig. 2**). Qualified DNA was filtered to get large segments using the BluePippin^{TM}System. The large segments DNA was used to prep a library using ONT Template prep kit and NEB Next FFPE DNA Repair Mix kit (NEB, New England, USA). The high quality library of average 20 kb in length was sequenced on the ONT PromethION platform with corresponding R9 cell and ONT sequencing reagents kit. The genomic DNA was sequenced using the MinION portable DNA sequencer with the 48 hours run script (Oxford Nanopore), which generated a total of 246.8 Gb data (**Table 1**).

Sequencing of Hi-C and genome survey libraries were performed on the Illumina

sequencing platform. Briefly, the extracted DNA were fragmentized into 300–350 bp size using an E210 Focused-ultrasonicator (Covaris, Woburn, MA, USA). Construction of the paired-end libraries encompassed the successive steps of end repair, poly(A) addition, barcode index, purification and PCR amplification. The libraries were sequenced with Illumina novaseq 6000 platform to generate 150 bp paired-end reads.

The qualified RNA extracted from the same tissues of ten individuals were equally mixed for RNA sequencing. The mRNA was enriched by poly(A) using Oligo-d(T) Magnetic Beads. Sequencing libraries were prepared using NEBNext® Ultra™ RNA Library Prep Kit for Illumina® (NEB, USA) following manufacturer`s recommendations. A total of ten libraries were sequenced on the Illumina novaseq 6000 platform in 150 bp paired-end model.

Genome survey and contig assembly

Genome size of the hard-shelled mussel was estimated using *K*-mer-based method implemented in GCE (v1.0.014) with values of 19-mers [28]. The *K*-mers refers to all the *k*-mer frequency distribution from a read obtained through the Illumina DNA sequencing. The occurrence peak of the assemblies is at 86 and the junior one is at 43 (**Fig. 3a**). The size (*G*) of genome was estimated by the calculation formula: $G = (N - S) / P$, where *G* is the estimated genome size, *N* is the total number of *K*-mer frequency, *S* is the number of single or unique *K*-mer words and *P* is *K*-mer occurrence number of the main peak frequency. As a result, the genome size is (141,038,363,649 - 4,929,816,595) / 86= 1.58 Gb, very close to the total assemblies (1.57 Gb). Owing to

high heterozygous rate, the previous report over-estimated the hard-shelled mussel genome size at around 1.9 Gb [24], which is much greater than the real size of 1.57 Gb – 1.58 Gb by 21%. Alignment of the survey reads to the chromosome assemblies was indicative of a heterozygous rate of 0.92%. Average GC content of genome is approximately 32%.

The genome assembly with the long read data followed three methods. Long reads were *de novo* assembled using the Canu v1.5 with default parameters [29], followed by error correction by Racon v1.3.1 [30]. Then further polishing with Illumina short read data was conducted using Pilon v1.22 [31]. The final assemblies is around 1.57 Gb in size, consisting of 6,449 contigs with an overall N50 length of 1.49 Mb, while the previously published draft genome only has a contig N50 length of 0.66 Mb [24].

Anchor of the contigs on chromosomes with Hi-C

The genome assemblies of hard-shelled mussel were constructed using the Hi-C technology to generate the interaction information among contigs. The fresh adductor muscle was used to prepare the Hi-C library. The Hi-C library was sequenced on Illumina novaseq 6000 platform, which produced 249.6 Gb reads (**Table 1**). When the reads were aligned to the assembled contigs using the BWA aligner v0.7.10-r789 [32], Lachesis v2e27abb was applied to anchor the contigs onto the chromosomes with the agglomerative hierarchical clustering method [33]. Finally, 2,029 contigs representing 90.9% of the total assemblies were successfully anchored into 14 chromosomes (**Table 2**), which is consistent with the karyotype [34]. N50 length of the anchored

contigs is over 1.7 Mb, which is 1.14 times the assemblies from ONT long reads.

Genome annotation

A *de novo* repeat annotation of the hard-shelled mussel genome was carried out with RepeatModeler (version1.0.11) [35] and RepeatMasker (version 4.0.7) [36]. RepeatModeler was used to construct the repeat library, complemented by two programs, RECON and RepeatScout. The yielded consensus sequences were manually checked by aligning to the GenBank database (nt and nr; released October 2019) to avoid grouping high-copied genes into the repetitive sequence library by mistake. The final repeat library consisted of 2,264 consensus sequences and their classification information, which was used to run RepeatMasker against the genome assemblies. The repetitive sequences occupied 735.6 Mb in length, representing 47.4% of the total genome length (**Supplementary Table S1**). Simple sequence repeats (SSRs) were identified by Tandem Repeats Finder V 4.04. Only the monomers, dimers, trimers, tetramers, pentamers and hexamers with at least 4 repeat units were collected. Total length of the SSRs is around 138.0 Kb, consisting of 5,324 SSRs,

The conserved non-coding RNAs were predicted with the Rfam 11.0 databases. Putative microRNAs (miRNAs) and ribosomal RNAs (rRNAs) were predicted using Infernal (version 1.1.2) [37], and transnfer RNAs (tRNAs) were predicted with tRNAscan-SE v2.0.3. A total of 9,186 miRNAs, 342 rRNAs and 1,881 tRNAs were detected, respectively (**Supplementary Table S2**).

Protein-coding genes were predicted using a combined strategy of *ab initio*

prediction, homology-based prediction and transcriptome-based prediction methods (**Fig. 2**). The *ab initio* prediction was conducted with the softwares of Augustus (version 3.1) [38], GlimmerHMM (version 1.2) [39] and SNAP (version 2006-07-28) [40]. For the homology-based prediction, protein sequences of two closed mollusc species (*Modiolus philippinarum* and *Bathymodiolus platifrons*) downloaded from the GenBank were aligned to the genome assemblies with Exonerate (version 2.2.0) [41]. Parallelly, the transcriptome data from ten tissues (GenBank SRA accession ID: PRJNA578350) were put into a *de novo* assemble with Trinity (version 2.4.0) [42] and Cufflinks (version 2.2.1) [43]. Outputs of both assemblers were integrated with the Program to Assemble Spliced Alignments (PASA, version 2.3.3) [44]. When all of these predictions were merged using EVidenceModeler (v1.1.0) [44], a total of 37,478 final gene models were generated (**Table 3)**, which was less than previously-published 42,684 gene models in the draft genome because it introduced over 20% heterozygous redundances in the assemblies [24]. Using InterPro (version 5.22-61.0) [45], GO [46], KEGG [47], Swissprot [48] and NCBI non-redundant proteins (NR) databases, 35,471 protein-coding genes (94.6% of total 37,478 gene models) were annotated by one or more functional databases (**Table 4**; **Fig. 3b**). All of the information was illustrated in a genome landscape map (**Fig. 3c**).

Phylogenetic analysis

The gene clusters were identified among 12 selected genome, *Chlamys farreri* (PRJNA185465), *Pinctada fucata martensii* (GCA_002216045.1), *M. philippinarum*

(GCA_002080025.1), *Crassostrea gigas* (GCF_000297895.1), *B. platifrons* (GCA_002080005.1), *Mizuhopecten yessoensis (GCA_002113885.2),* *Penaeus vannamei* (ASM378908v1), *Pecten maximus* (GCA 902652985.1), *Scapharca* (*Anadara*) *broughtonii* (PRJNA521075), *Pomacea canaliculata* **(**PRJNA427478), *Haliotis discus hannai* (PRJNA317403) and *M. coruscus* using OrthoMCL (version1.4) [49]. Amino acid sequences of 448 single-copy genes were concatenated to create a super gene. The concatenated sequences from 12 species were aligned using MUSCLE [50]. The multiple sequence alignments were used to infer the phylogenetic relationship with the RAxML version 8 [51] based on the Maximum-likehood model. The phylogenetic relationship visualized with FigTree (version 1.4.3) [52] reflected the closest relationship between *M. coruscus* and the clade of *M. philippinarum* and *B. platifrons*, of which the divergence time was approximately 129 Mya (**Fig. 3d**).

Whole genome re-sequencing of farmed and wild individuals

To compare sequence variations between farmed and wild populations, whole genome re-sequencing was performed in two genomic DNA pools, one from ten farmed individuals and the other from ten wild individuals. A total of 50.4 Gb and 46.7 Gb Illumina clean reads were finally generated in farmed and wild sample, respectively. Approximately 89.2% and 88.9% of reads in the two pools were aligned to the reference genome with BWA v0.7.10-r789 [32] to remove PCR-duplicates (duplicates introduced by PCR) with Picard [53]. The SNPs and small indels (10 bp or less) were identified using the GATK version 3.7 [54] with default parameters, in which three additional

thresholds were used to discarded unreliable items in the post-filter: 1) any two SNPs located within 5 bp; 2) any two indels located within 10 bp; 3) any SNPs located within 5bp to an indel. Thus, we identified 5,733,780 SNPs and 1,821,690 small indels in farmed population, while 5,719,771 and 1,820,404 in wild one. These SNPs and indels exhibited the similar distributions in the genome (**Fig. 4a**). Comparison of the loci revealed that nearly 99% of the SNPs/indels were shared by both two populations (**Fig. 4b**), which reflected that only around 1% of the sequence variations were farmed-population-specific (FPS) or wild-population-specific (WPS). We focused on those differential variations between the farmed and wild population located in flanking regions and genic regions to identify candidate genes and casual mutations related to morphology traits. A software SnpEff version 2.0.5 [55] was then used to predict the variant effect of SNPs/indels by comparing loci between SNPs/indels and protein-coding genes, which detected that 59 genes carrying FPS SNPs/indels (FPSG) and 57 carrying WPS SNPs/indels (WPSG) were functionally affected by lost translational start site, gained/lost stop codon or the variants in acceptor/donor of splicing sites. Of them, eight FPSGs and four WPSGs were annotated in KEGG database [47], which are mainly involved in genetic information processing, metabolism, and signaling and cellular processes (**Supplementary Table S3**). Among the annotated genes, hexosaminidase, alternatively named as chitobiase, has been reported to regulate prismatic shell formation [56, 57]. Chitobiase, as one of chitinolytic enzymes, was proposed to regulate the thinness of chitin and play an important role in the formation of calcite prismatic layer. In practice, morphology of mollusk shell will change as

generation succession. Mutation in chitobiase will influence the shell morphology [58], which implying the association of gene mutation and transformation of morphology of shell.

Chromosome synteny and evolution in bivalves

To investigate the evolution of the hard-shelled mussel chromosomes, gene collinearity was determined by aligning the king scallop *P. maximus* genes to the reference genomes of the blood clam *S. broughtonii*, the hard-shelled mussel *M. coruscus*, the pearl oyster *P. martensii* and the pacific oyster *C. gigas*, respectively, using MCscan (version 0.8). The parameters of the MCscan alignment were as follows: -s 7 –k 150 –m 250 –e 1e-10. We identified 404 scallop-vs-clam, 276 scallop-vs-mussel, 159 scallop-vs-pearl-oyster and 232 scallop-vs-pacific-oyster syntenic blocks, which localized 10,055, 4,716, 3,636, and 5,009 genes in blood clam, hard-shelled mussel, pearl oyster and pacific oyster, respectively. The mean gene number per block was 21.4 genes. It was observed that king scallop and blood clam had the highest gene collinearity, consistent with their closest phylogenetic relationship in the Bivalvia clade. The chromosome synteny illustrated that rare large-scale rearrangements between scallop and mussel, but frequent between scallop and oysters (**Fig. 5b-d**), such as considerable structural variations between the scallop and the pacific oyster (**Fig. 5d**). The identified cross-chromosome rearrangements between scallop and mussel were different from those between scallop and the two oysters (**Fig. 5b-e**). Scallop linkage groups (PM) 1, 5, 6, 8, 10, 16, 17, 18 and 19 were syntenic to a single mussel

Chromosome (MC) 8, 9, 3, 4, 10, 13, 11, 12, and 14, respectively. PM 2 and 15 were aligned to the same reference MC 2, similarly PM 3 and 14 aligned to MC 5, PM 4 and 7 aligned to MC 1, PM 9 and 12 aligned to MC 7, and PMs 11 and 13 aligned to MC 6. Comparatively, a little more chromosome rearrangement occurred between scallop, especially the pacific oyster, and the two oysters. Both the pacific oyster Chromosome 9 and the pearl oyster Chromosome 7 was predominantly syntenic to scallop PM 15, suggesting that they might carry the conserved genome regions with the same originals (**Fig. 5c-e**). Among all of the syntenic chromosomes, we did not observe any chromosomes to be entirely conserved by all of the bivalve genomes. Intriguingly, almost all of the chromosomes rearrangements between the mussel and the oyster genomes were different (**Fig. 5e**), implicating the independent chromosome fusion events. These identified diverse chromosome rearrangements suggested a complex evolutionary history in the bivalve chromosomes.

Transcriptome related to metamorphosis

To profile the gene expressions during development and metamorphosis in hard-shelled mussel, an RNA-seq analysis was conducted in five developmental stages, including trochophore, D-veliger, umbo larva, pediveliger and juvenile. The quantified gene expressions indicated that 27,118 transcripts were detected with the FPKM > 0 during all the stages (**Supplementary Table S4**). Using trochophore as the control, 5,560, 5,236, 5,560 and 7,289 up-regulated genes ($\log_2$(Fold-change) > 1 and $P < 0.05$) were identified in D-veliger, umbo larva, pediveliger and juvenile, respectively.

Functional annotation indicated that they were mainly involved in “environmental information processing” (“signal transduction” and “signaling molecules and interaction”) and “cellular processes” (“transport and catabolism”), agreeing with the key role of signal transduction and endocrine system in the larvae development [17].

Since capability of metamorphosis occurs during the pediveliger stage [17], we identified 315 up-regulated genes during the period from umbo to pediveliger, which were predominantly annotated to be employed in “adrenergic signaling in cardiomyocytes”, “calcium signaling pathway”, “MAPK signaling pathway”, “protein export”, “endocytosis” and “catecholamine biosynthesis” pathways (**Fig. 6a**). Of these genes, 14 exhibited the increased expression during the early stage from trochophore to D-veliger (**Fig. 6b**), which clustered in “adrenergic signaling in cardiomyocytes”, “calcium signaling pathway” and “catecholamine biosynthesis”. Most of them were constantly expressed in high levels during the following four stages. In the “catecholamine biosynthesis pathway”, the genes encoding tyrosinase, aromatic-L-amino-acid decarboxylase and dopamine beta-monooxygenase exhibited increased expression during the initial two stages (trochopore and D-veliger), which catalyzes three successive reactions of biosynthesis of L-dopa, dopamine (DA) and noradrenaline (NE) [59] (**Fig. 6**). DA and NE have been identified to activate and regulate metamorphosis, respectively [60, 61]. In addition, CAV-1 and CAV-2 were more highly expressed during the stages from trochopore to pediveliger, consistent with its reported larvae features in mollusk and annelid [62]. Products from catecholamine biosynthesis and exocytosis probably influxed into adrenergic signaling pathway [63], of which the

adrenergic-like receptors are functionally localized around heart in oyster umbo-veliger larvae [64]. Taken together, analysis of involved pathways exhibited that biosynthesis, transport, and transduction of catecholamine should be critical to completeness of metamorphosis. Signal transduction controlling the metamorphosis development seemed to activate during the first two stages, trochophore and D-veliger, although the major morphologic changes represented in the transition from pediveliger to juvenile.

Assembly assessment

Quality of the assembled genome was validated on terms of the completeness, accuracy of the assemblies and conservation of synteny. Alignment of the Illumina reads against the reference genome exhibited that insert sizes of paired-end sequencing libraries clustered at 300 - 350 bp and the mapping rate was over 96.7%. BUSCO test was indicative of a 91.9% genome completeness when 89.98% of core metazoan orthologs were completely identified in the assemblies. Accuracy of the genome assembly was evaluated by calling sequence variants with the alignments of Illumina data against the genome. Processing of the sequence alignment with the BCFtools (version 1.3) [65] detected 368,991 homozygous SNP loci, reflecting an error rate of less than 0.02% in genome assemble. Besides, a high conserved synteny and strict correspondence of chromosome fusion and assignment identified between the hard-shelled mussel and king scallop (Fig. 5b) stand for a qualified assembly in hard-shelled mussel since the king scallop genome is considered as the best-scaffolded genome available for bivalves [66]. The high heterozygosity (0.92%) was estimated by aligning survey reads to the

assemblies, less than the wild king scallop (1.71%) but greater than the inbred pacific oyster (0.73%) [66].

Conclusion

The chromosome-level genome assembly of hard-shelled mussel presented here is a well-assembled and annotated resource that will facilitate a wide range of researches in mussel, bivalve, and molluscan. Outputs of this study shed light on the chromosome evolution in bivalve, such as genetic diversities between the farmed and the wild populations and regulation molecular pathways involved in larval metamorphosis. As one of the best-assembled bivalve genomes, this genome data set will serve as a high-quality genome platform for comparative genomics in chromosome level.

Availability of Supporting Data and Materials

All of the raw Illumina and ONT reads were deposited to NCBI Sequence Read Archive and the assembled genome was deposited to GenBank under the accession number PRJNA578350. The corresponding genome sequences, read alignments (VCF files) and other supporting files were stored in Figshare [67] and GigaDB [68].

Abbreviations

FPKM: Fragments Per Kilobase per Million mapped fragments; GATK: Genome Analysis Tool Kit; GO: Gene Ontolog; KEGG: Kyoto Encyclopedia of Genes and Genomes; AC1: adenylate cyclase 1; AC10: adenylate cyclase 10; Akt: RAC

serine/threonine-protein kinase; CaM: calmodulin; CaMKII: calcium/calmodulin-dependent protein kinase (CaM kinase) II; CAV1: caveolin 1; CAV3: caveolin 3; CREB: cyclic AMP-responsive element-binding protein; DBH: dopamine beta-monooxygenase; DDC: aromatic-L-amino-acid decarboxylase; DHPR: voltage-dependent calcium channel gamma-1; Epac: Rap guanine nucleotide exchange factor; ERK: mitogen-activated protein kinase 1/3; Gi: guanine nucleotide-binding protein G(i) subunit alpha; Gq: guanine nucleotide-binding protein G(q) subunit alpha; Gs: guanine nucleotide-binding protein G(s) subunit alpha; ICER: cAMP response element modulator; IKS: potassium voltage-gated channel KQT-like subfamily member 1; IMP2: mitochondrial inner membrane protease subunit 2; INaK: sodium/potassium-transporting ATPase subunit alpha; MAOA: monoamine oxidase A; MAOB: monoamine oxidase B; MSK1: ribosomal protein S6 kinase alpha-5; NCX : solute carrier family 8 (sodium/calcium exchanger); NF-κB: nuclear factor NF-kappa-B p105 subunit; NHE: solute carrier family 9 (sodium/hydrogen exchanger); p38MAPK: p38 MAP kinase; PI3K: phosphatidylinositol-4,5-bisphosphate 3-kinase catalytic subunit alpha/beta/delta; PKA: protein kinase A; PKCα: classical protein kinase C alpha type; PLC: phosphatidylinositol phospholipase C; PP1: serine/threonine-protein phosphatase PP1 catalytic subunit; TnI: Troponin I; TPM: tropomyosin; TYR: tyrosinase; α-ARA: alpha-1A adrenergic receptor-like; α-ARB: adrenergic receptor alpha-1B; β2AR: adrenergic receptor beta-2.

Competing Interests

The authors declare no competing interests.

Funding

This study was supported by the National Key Research and Development Program of China (2018YFD0900601), Key Program for International Science and Technology Cooperation Projects of Ministry of Science and Technology of China (No. 2018YFD0900101), the National Natural Science Foundation of China (No. 41876159, No. 41606147, No. 31802321), Key Special Project for Introduced Talents Team of Southern Marine Science and Engineering Guangdong Laboratory (Guangzhou) (GML2019ZD0402), the Shanghai Sailing Program (18YF1410000) and Program for study on genetic resources, environment and strategy of mussel culture in coast of Gouqi Island offshore.

Authors Contributions

J.L.Y., Y.L. and X.L. designed and supervised the study. K.C., J.K.X, Y.T.Z, Y.F.L. collected the samples and extracted the genomic DNA and RNA. Y.L., J.L. and D.D.F. performed genome assembly and bioinformatics analysis. J.L.Y., D.D.F., X.L., J.L. and Y.L. wrote the original manuscript. All authors reviewed the manuscript.

Figure legends

Figure 1. Sequenced individuals and sampling sites. **a.** Pictures of the sequenced individuals collected in Shengsi. The wild *M. coruscus* adult was used in genome sequencing. Both wild and farmed populations were used in re-sequencing. **b.** The geographic locations of the sampling sites.

Figure 2. Workflow of genome sequencing and annotation. The rectangles indicate the steps of the data treatment and the diamonds indicate the output or input data.

Figure 3. Annotation and evolution. **a.** Distribution of 19-mer frequency. Values of *K*-mers are plotted against the frequency (*y* axis) of their occurrence (*x* axis). **b.** The Venn diagram indicates that the genes are annotated with one or more databases. **c.** Genome landscape of *M. coruscus*. The chromosomes were labelled as LG01 to LG14. From outer to inner circos: 5, maker distribution across 14 chromosomes at megabase scales; 4, gene density across the whole genome; 3, SNP density; 2 and 1, repetitive sequences and GC content across the genome. 1-5 are drawn in non-overlapping 0.1Mb sliding windows. Length of chromosomes is measured by the sale (Mb) on the outer circles. **d.** Phylogenetic tree based on protein sequences from 8 metazoan genomes, including *Chlamys farreri* (PRJNA185465), *Pinctada fucata martensii* (GCA_002216045.1), *Modiolus philippinarum* (GCA_002080025.1), *Crassostrea gigas* (GCF_000297895.1), *Mytilus coruscus*, *Bathymodiolus platifrons* (GCA_002080005.1), *Mizuhopecten yessoensis* (GCA_002113885.2), *Penaeus vannamei* (ASM378908v1), *Pecten*

maximus (GCA 902652985.1), *Scapharca* (*Anadara*) *broughtonii* (PRJNA521075), *Pomacea canaliculata* **(**PRJNA427478), *Haliotis discus hannai* (PRJNA317403).

Figure 4. Sequence variations between farmed and wild populations**. a.** Circos to show genome-wide SNPs and Indels from the farmed and wild population, respectively. From outer to inner circos: the first circle, maker distribution across 14 chromosomes at megabase scales; the green circle, SNP density across the whole genome; the red circle, Indel density. **b.** Venns to show the number distribution of Indels from the farmed and wild population, as well as SNPs.

Figure 5. Chromosome synteny. **a.** Chromosome alignment of the king scallop and the blood clam. **b.** Chromosome alignment of the king scallop and the hard-shelled mussel. **c**. Chromosome alignment of the king scallop and the pearl oyster. **d**. Chromosome alignment of the king scallop and the Pacific Oyster. The king scallop linkage groups are labeled as PM 1 to 19, the blood clam chromosomes labeled as SB 1 to 19, the hard-shelled mussel chromosomes labeled as MC1 to 14, the pearl oyster chromosomes labeled as PF1 to 14 and the Pacific Oyster chromosomes labeled as CG 1 to 10. Scale unit, Mb. a-d. The circularized blocks represent the chromosomes of five bivalves. Aligned homologous genes are connected by ribbons, shown in different colors depending on their chromosome locations. **e.** Chromosome rearrangement between the king scallop and other four bivalves. The king scallop chromosomes are represented by different colors bars, synteny and rearrangement of the other four bivalves

chromosomes are indicated by different blocks, corresponding to the reference king scallop chromosome.

Figure 6. Spatial and temporal expression of genes involved development and metamorphosis. **a.** Catecholamines biosynthesis and adrenergic signaling in cardiomyocytes pathway annotation of genes generated by KEGG. Red rectangle indicates up-regulated genes during development and metamorphosis, white rectangle denotes genes that were identified in the KEGG analysis and didn`t represent expression changes. Red bubble represents the important pathways involved the up-regulated genes. **b.** Heatmap shows expression level across five developmental stages of all genes involved in catecholamines biosynthesis and adrenergic signaling in cardiomyocytes pathways.

Table caption

Table 1. Statistics of whole genome sequencing using Illumina and ONT

Table 2. Anchored contigs on chromosomes with Hi-C technology

Table 3. General statistics of predicted protein-coding genes

Table 4. General statistics of gene function annotation

Additional Files

Supplementary Table S1. Repetitive sequences in the hard-shelled mussle genome

Supplementary Table S2. Overview of predicted non-coding RNAs

Supplementary Table S3. KEGG annotation information of the genes carrying population-specific SNP/INDELs

Supplementary Table S4. Quantified gene expressions normalized to FPKM values

References

1. FAO. The state of world fisheries and aquaculture. 2018.
2. Amini S, Kolle S, Petrone L, et al. Preventing mussel adhesion using lubricant-infused materials. Science 2017; **357**:668-673.
3. Yang JL, Li YF, Guo XP, et al. The effect of carbon nanotubes and titanium dioxide incorporated in PDMS on biofilm community composition and subsequent mussel plantigrade settlement. Biofouling 2016; **32**:763-777.
4. Yang JL, Shen PJ, Liang X, et al. Larval settlement and metamorphosis of the mussel *Mytilus coruscus* in response to monospecific bacterial biofilms. Biofouling 2013; **29**:247-259.
5. Liang X, Peng LH, Zhang S, et al. Polyurethane, epoxy resin and polydimethylsiloxane altered biofilm formation and mussel settlement. Chemosphere 2019; **218**:599-608.
6. Odonnell MJ, George MN, Carrington E. Mussel byssus attachment weakened by ocean acidification. Nature Climate Change 2013; **3**:587-590.
7. Ramesh K, Hu MY, Thomsen J, et al. Mussel larvae modify calcifying fluid carbonate chemistry to promote calcification. Nature Communications 2017; **8**:1709.
8. Thomsen J, Stapp L, Haynert K, et al. Naturally acidified habitat selects for

ocean acidification–tolerant mussels. Science Advances 2017; **3**:e1602411.

9. Bitter MC, Kapsenberg L, Gattuso J, et al. Standing genetic variation fuels rapid adaptation to ocean acidification. Nature Communications 2019; **10**:1-10.

10. Briand J. Marine antifouling laboratory bioassays: an overview of their diversity. Biofouling 2009; **25**:297-311.

11. Petrone L, Kumar A, Sutanto CN, et al. Mussel adhesion is dictated by time-regulated secretion and molecular conformation of mussel adhesive proteins. Nature Communications 2015; **6**:8737-8737.

12. Zeng ZS, Guo XP, Cai XS, et al. Pyomelanin from *Pseudoalteromonas lipolytica* reduces biofouling. Microbial Biotechnology 2017; **10**:1718-1731.

13. Murgarella M, Puiu D, Novoa B, et al. A first insight into the genome of the filter-feeder mussel *Mytilus galloprovincialis*. PLoS One 2016; **11**:e0151561.

14. Sun J, Zhang Y, Xu T, et al. Adaptation to deep-sea chemosynthetic environments as revealed by mussel genomes. Nature Ecology and Evolution 2017; **1**:0121.

15. Hadfield MG, Paul VG. In *Marine chemical ecology* (ed. McClintock, J.B & Baker, J.B) Ch13. CRC Press, 2001.

16. Dobretsov S, Rittschof D. Love at first taste: induction of larval settlement by marine microbes. International Journal of Molecular Sciences 2020; **21**:731.

17. Hadfield MG. Biofilms and marine invertebrate larvae: what bacteria produce that larvae use to choose settlement sites. Annual Review of Marine Science 2011; **3**:453-470.

18. Shikuma NJ, Antoshechkin I, Medeiros JM, et al. Stepwise metamorphosis of the tubeworm *Hydroides elegans* is mediated by a bacterial inducer and MAPK signaling. Proceedings of the National Academy of Sciences of the United States of America 2016; **113**:10097-10102.

19. Shikuma NJ, Pilhofer M, Weiss GL, et al. Marine tubeworm metamorphosis induced by arrays of bacterial phage tail–like structures. Science 2014; **343**:529-533.

20. Kulikova VA, Lyashenko SA, Kolotukhina NK. Seasonal and interannual dynamics of larval abundance of *Mytilus coruscus* Gould, 1861 (Bivalvia: Mytilidae) in Amursky Bay (Peter the Great Bay, Sea of Japan). Russian Journal of Marine Biology 2011; **37**:342-347.

21. Li YF, Liu YZ, Chen YW, et al. Two toll-like receptors identified in the mantle of *Mytilus coruscus* are abundant in haemocytes. Fish & shellfish immunology 2019; **90**:134-140.

22. Liang X, Zhang XK, Peng LH, et al. The flagellar gene regulates biofilm formation and mussel larval settlement and metamorphosis. International Journal of Molecular Sciences 2020; **21**:710.

23. Yang JL, Li SH, Li YF, et al. Effects of neuroactive compounds, ions and organic solvents on larval metamorphosis of the mussel *Mytilus coruscus*. Aquaculture 2013; **396-399**:106-112.

24. Li RH, Zhang WJ, Lu JK, et al. The whole-genome sequencing and hybrid assembly of *Mytilus coruscus*. Frontiers in Genetics 2020; **11**:1-6.

25. Li YL, Sun XQ, Hu XL, et al. Scallop genome reveals molecular adaptations to semi-sessile life and neurotoxins. Nature Communications 2017; **8**:1721-1721.

26. Wang S, Zhang J, Jiao W, et al. Scallop genome provides insights into evolution of bilaterian karyotype and development. Nature ecology & evolution 2017; **1**:0120.

27. Van Berkum NL, Lieberman-Aiden E, Williams L, et al. Hi-C: A method to study the three-dimensional architecture of genomes. Journal of Visualized Experiments 2010; **39**:e1869.

28. Liu BH, Shi YJ, Yuan JY, et al. Estimation of genomic characteristics by analyzing k-mer frequency in de novo genome projects. Quantitative Biology 2013:https://arxiv.org/abs/1308.2012.

29. Koren S, Walenz BP, Berlin K, et al. Canu: scalable and accurate long-read assembly via adaptive k-mer weighting and repeat separation. Genome research 2017; **27**:722-736.

30. Vaser R, Sović I, Nagarajan N, et al. Fast and accurate de novo genome assembly from long uncorrected reads. Genome research 2017; **27**:737-746.
31. Walker BJ, Abeel T, Shea T, et al. Pilon: an integrated tool for comprehensive microbial variant detection and genome assembly improvement. PloS one 2014; **9**:e112963.
32. Li H, Durbin R. Fast and accurate short read alignment with Burrows–Wheeler transform. Bioinformatics 2009; **25**:1754-1760.
33. Burton JN, Adey A, Patwardhan RP, et al. Chromosome-scale scaffolding of de novo genome assemblies based on chromatin interactions. Nature Biotechnology 2013; **31**:1119-1125.
34. Zhuang BX. A preliminary study on the chromosome of marine bivalve, *Mytilus coruscus*. Zoological Research 1984; **S2**.
35. Smit A, Hubley R. RepeatModeler Open-1.0. 2008:http://www.repeatmasker.org/.
36. Smit A, Hubley R, Green P. RepeatMasker Open-4.0. 2015:http://www.repeatmasker.org/.
37. Nawrocki EP, Eddy SR. Infernal 1.1: 100-fold faster RNA homology searches. Bioinformatics 2013; **29**:2933-2935.
38. Stanke M, Keller O, Gunduz I, et al. AUGUSTUS: *ab initio* prediction of alternative transcripts. Nucleic acids research 2006; **34**:W435-W439.
39. Majoros WH, Pertea M, Salzberg SL. TigrScan and GlimmerHMM: two open source *ab initio* eukaryotic gene-finders. Bioinformatics 2004; **20**:2878-2879.
40. Korf I. Gene finding in novel genomes. BMC Bioinformatics 2004; **5**:59.
41. Slater GSC, Birney E. Automated generation of heuristics for biological sequence comparison. BMC bioinformatics 2005; **6**:31.
42. Grabherr MG, Haas BJ, Yassour M, et al. Trinity: reconstructing a full-length transcriptome without a genome from RNA-Seq data. Nature biotechnology 2011; **29**:644.
43. Trapnell C, Williams BA, Pertea G, et al. Transcript assembly and quantification by RNA-Seq reveals unannotated transcripts and isoform

switching during cell differentiation. Nature biotechnology 2010; **28**:511.

44. Haas BJ, Salzberg SL, Zhu W, et al. Automated eukaryotic gene structure annotation using EVidenceModeler and the Program to Assemble Spliced Alignments. Genome biology 2008; **9**:R7.

45. Zdobnov EM, Apweiler R. InterProScan–an integration platform for the signature-recognition methods in InterPro. Bioinformatics 2001; **17**:847-848.

46. Ashburner M, Ball CA, Blake JA, et al. Gene ontology: tool for the unification of biology. Nature genetics 2000; **25**:25.

47. Kanehisa M, Goto S, Kawashima S, et al. The KEGG resource for deciphering the genome. Nucleic Acids Research 2004; **32**:277-280.

48. Boeckmann B, Bairoch A, Apweiler R, et al. The SWISS-PROT protein knowledgebase and its supplement TrEMBL in 2003. Nucleic acids research 2003; **31**:365-370.

49. Li L, Stoeckert CJ, Roos DS. OrthoMCL: identification of ortholog groups for eukaryotic genomes. Genome research 2003; **13**:2178-2189.

50. Edgar RC. MUSCLE: multiple sequence alignment with high accuracy and high throughput. Nucleic acids research 2004; **32**:1792-1797.

51. Stamatakis A. RAxML-VI-HPC: maximum likelihood-based phylogenetic analyses with thousands of taxa and mixed models. Bioinformatics 2006; **22**:2688-2690.

52. Rambaut A. FigTree, a graphical viewer of phylogenetic trees. 2007:http://tree.bio.ed.ac.uk/software/figtree/.

53. PicardToolkit. Broad Institute, GitHub Repository 2019:http://broadinstitute.github.io/picard/.

54. Mckenna A, Hanna M, Banks E, et al. The Genome Analysis Toolkit: A MapReduce framework for analyzing next-generation DNA sequencing data. Genome Research 2010; **20**:1297-1303.

55. Cingolani P, Platts AE, Wang LL, et al. A program for annotating and predicting the effects of single nucleotide polymorphisms, SnpEff: SNPs in the genome of *Drosophila melanogaster* strain w1118. Fly 2012; **6**:80-92.

56. Marie B, Joubert C, Tayalé A, et al. Different secretory repertoires control the biomineralization processes of prism and nacre deposition of the pearl oyster shell. Proceedings of the National Academy of Sciences 2012; **109**:20986-20991.
57. Feng D, Li Q, Yu H, et al. Identification of conserved proteins from diverse shell matrix proteome in *Crassostrea gigas*: characterization of genetic bases regulating shell formation. Scientific reports 2017; **7**:45754.
58. Kintsu H, Okumura T, Negishi L, et al. Crystal defects induced by chitin and chitinolytic enzymes in the prismatic layer of *Pinctada fucata*. Biochemical and biophysical research communications 2017; **489**:89-95.
59. Eisenhofer G, Tian H, Holmes C, et al. Tyrosinase: a developmentally specific major determinant of peripheral dopamine. The FASEB Journal 2003; **17**:1248-1255.
60. Bonar DB, Coon SL, Walch M, et al. Control of oyster settlement and metamorphosis by endogenous and exogenous chemical cues. Bulletin of Marine Science 1990; **46**:484-498.
61. Joyce A, Vogeler S. Molluscan bivalve settlement and metamorphosis: neuroendocrine inducers and morphogenetic responses. Aquaculture 2018; **487**:64-82.
62. Wang J, Zhang L, Lian S, et al. Evolutionary transcriptomics of metazoan biphasic life cycle supports a single intercalation origin of metazoan larvae. Nature Ecology and Evolution 2020:1-12.
63. Grimm M, Kurz T, Schwarz M, et al. Presynaptic regulation of cardiac norepinephrine release in Ischemia. Journal of Cardiovascular Pharmacology 2001; **38**:58-68.
64. Liang X, Chen K, Li Y-F, et al. An α2-adrenergic receptor is involved in larval metamorphosis in the mussel, *Mytilus coruscus*. Biofouling 2019; **35**:986-996.
65. Narasimhan VM, Danecek P, Scally A, et al. BCFtools/RoH: a hidden Markov model approach for detecting autozygosity from next-generation sequencing data. Bioinformatics 2016; **32**:1749-1751.

66. Kenny NJ, Mccarthy S, Dudchenko O, et al. The Gene-Rich Genome of the Scallop *Pecten maximus*. bioRxiv 2020.

67. Feng DD. The hard-shelled mussel *Mytilus coruscus* gene models, annotatins and related files of the whole genome. Figshare 2020:doi:10.6084/m6089.figshare.10259618.

68. Yang JL, Feng DD, Liu J, et al. Supporting data for "A chromosome-level genome assembly of the hard-shelled mussel *Mytilus coruscus* widely distributed in temperate seas of East Asia". GigaScience Database 2020.

Click here to access/download;Table;Table 1-4.docx

Table 1 Statistics of genome sequencing

**Types**	**Method**	**Library size (bp)**	**Reads number**	**Clean data (Gb)**	**length (bp)**	**coverage (x)**
Genome	Illumina	300-350	1,235,384,620	160.6G	150	104×
Genome	ONT	20,000	11,108,773	246.8G	30,945 (N50)	159×
Genome	Hi-C	\	832,911,978	249.6G	150	161×
Transcriptome	Illumina	300-350	787,692,308	102.4G	150	\

Table 2 Anchored contigs on linkage groups with Hi-C technology.

LG	Length (bp)	Gene Number	Contig N50 length (bp)	Contig Number
LG01	141,585,364	3,535	2,274,693	122
LG02	144,576,766	3,347	3,700,000	88
LG03	99,268,963	2,454	1,068,300	196
LG04	99,542,347	2,554	894,135	225
LG05	122,084,758	3,159	2,900,000	96
LG06	102,382,230	2,442	2,078,006	106
LG07	122,148,919	2,720	3,437,001	91
LG08	101,363,610	2,456	2,665,365	138
LG09	90,511,107	2,243	1,458,983	124
LG10	94,491,177	2,295	1,062,238	172
LG11	85,619,405	1,927	619,639	249
LG12	76,129,233	1,754	767,559	180
LG13	79,962,191	1,837	2,050,444	117
LG14	63,392,598	1,391	1,000,000	125
**Total**	**1,423,058,668**	**34,114**	**1,700,000**	**2,029**

Table 3 General statistics of protein-coding gene modeling

Gene set		Number	Average transcript length (bp)	Average CDS length (bp)	Average exons per gene	Average exon length (bp)	Average intron length (bp)
De novo	SNAP	52,359	15,377	488	4.8	101	3,894
	GlimmerHMM	196,665	7,017	525	3.3	157	2,776
	Augustus	67,930	8,512	1,036	4.1	250	2,380
Homolog	*B. platifrons*	34,836	10,631	784	3.6	217	3,778
	M. philippinarum	27,088	7,174	643	2.8	227	3,568
Trans.orf/RNAseq		53,578	16,183	966	6.0	275	2,900
Final EVM models		37,478	14,735	1,290	5.9	217	2,727

Table 4 Quantity of gene function annotations

Type		Number	Percentage (%)
Total		37,478	100.0
Annotated	InterPro	32,821	87.6
	GO	18,497	49.4
	KEGG	7,625	20.3
	Swissprot	16,868	45.0
	NR	31,489	84.0
Annotated		35,471	94.6
Unannotated		2,007	5.4

Figure 1. Sequenced individuals and sampling sites.

Click here to access/download;Figure;Fig1 Sequenced individuals and sampling sites.tif

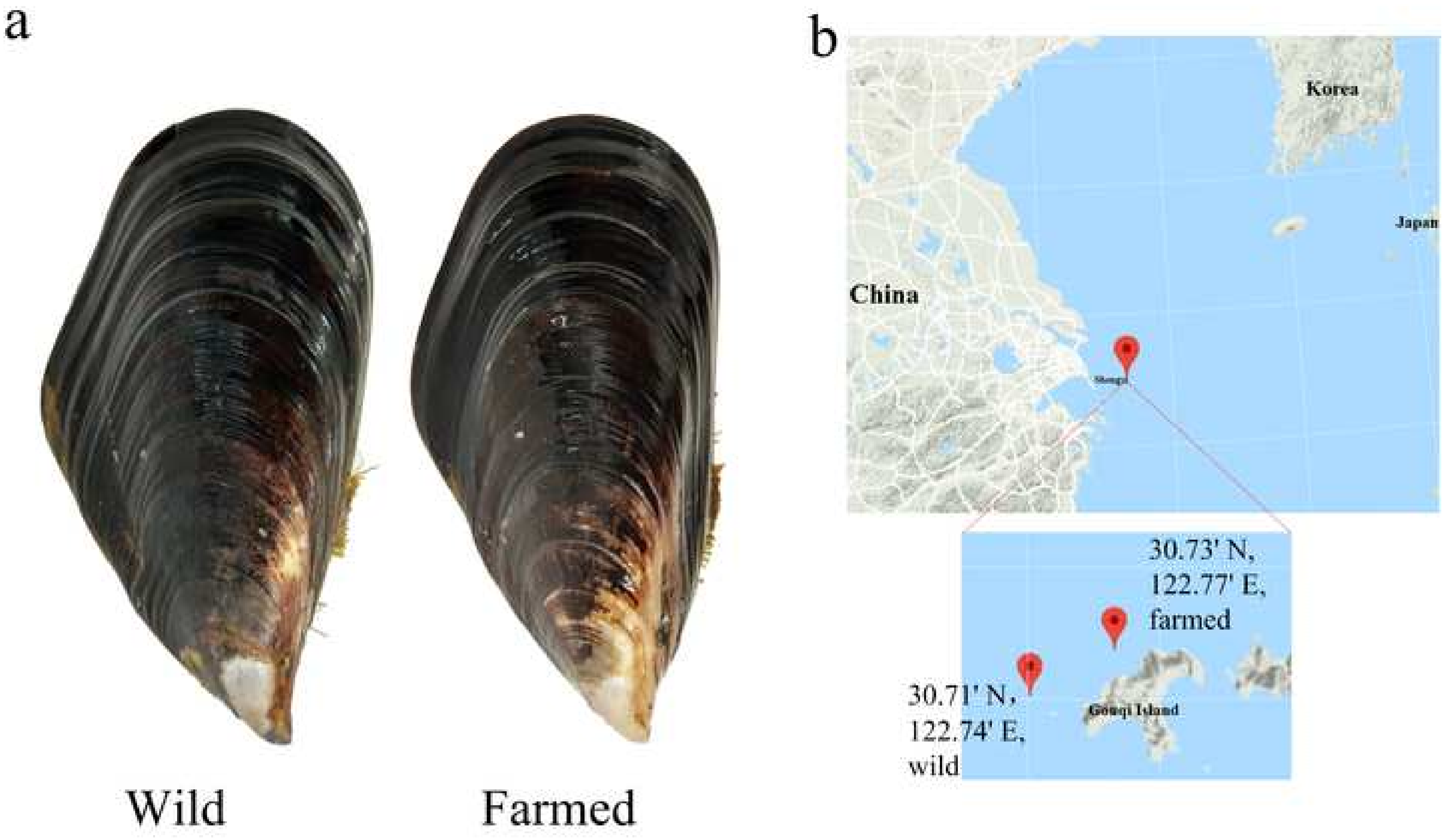

Figure 2. Workflow of genome sequencing and annotation.

Click here to access/download;Figure;Fig2 Workflow of genome sequencing and annotation.tif

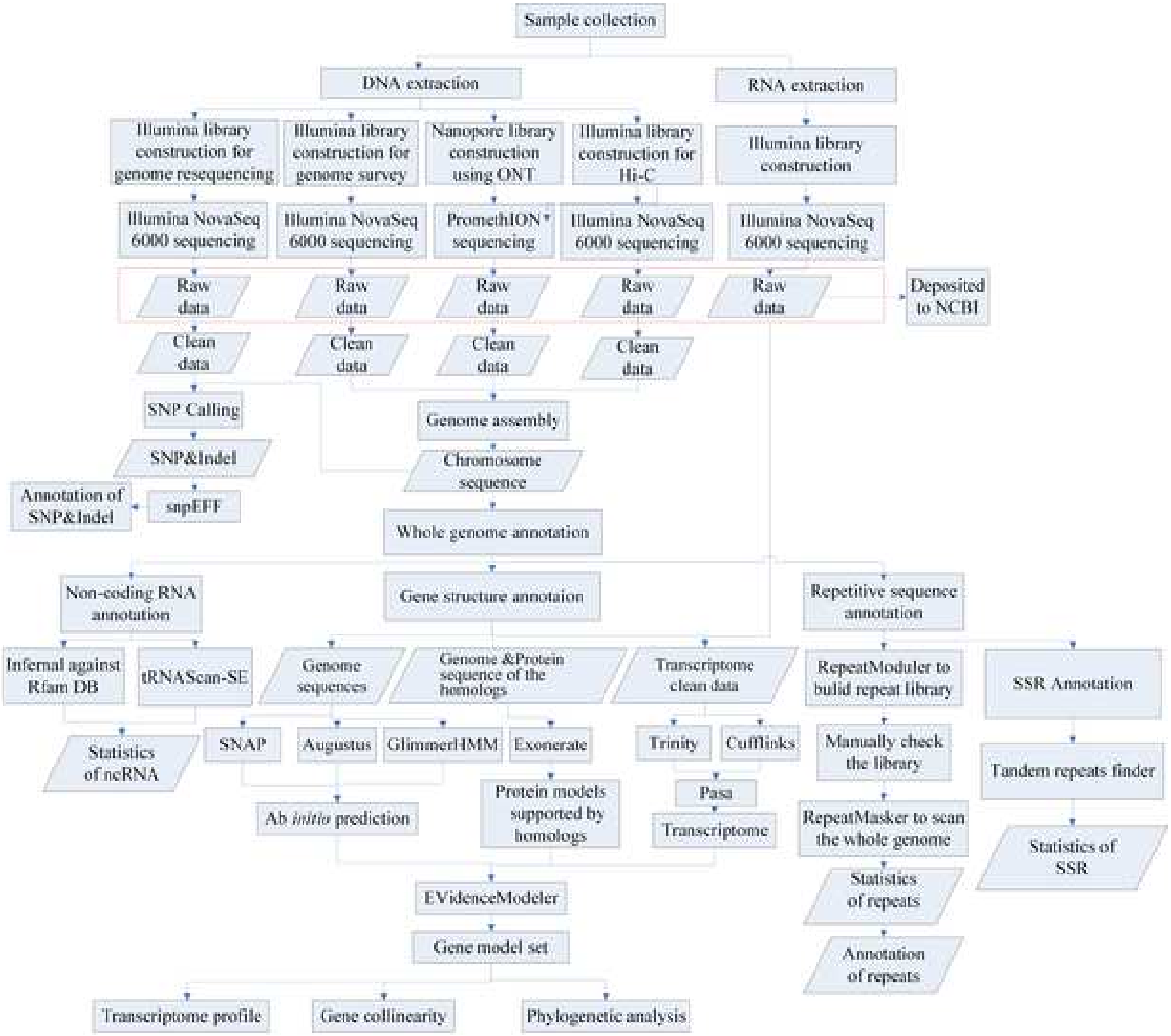

Figure 3. Annotation and evolution.

Click here to access/download;Figure;Fig3 genome annotation and evolution.tif

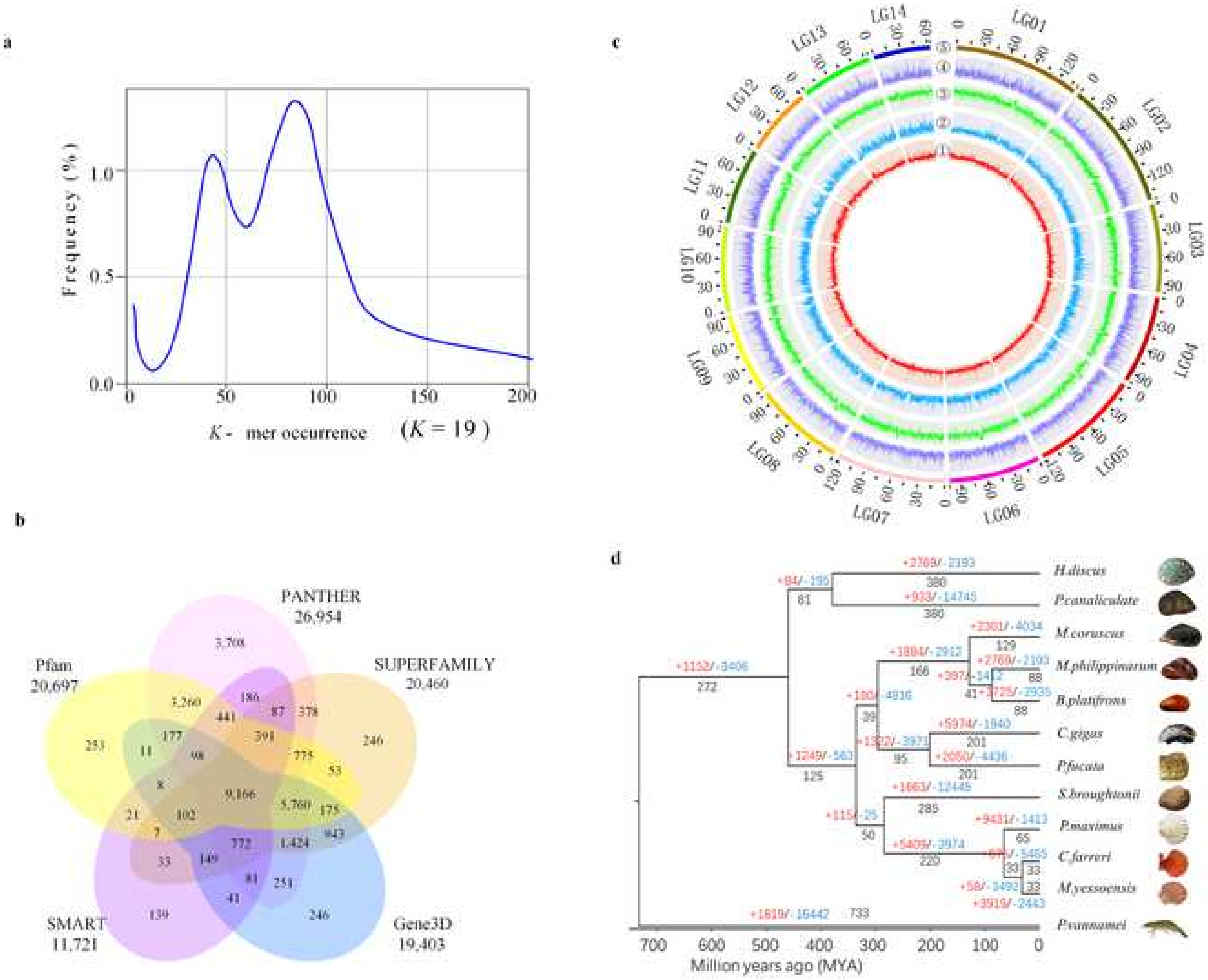

Figure 4. Sequence variations between farmed and wild populations.

Click here to access/download;Figure;Fig4 resequence of farmed and wild populations.tiff

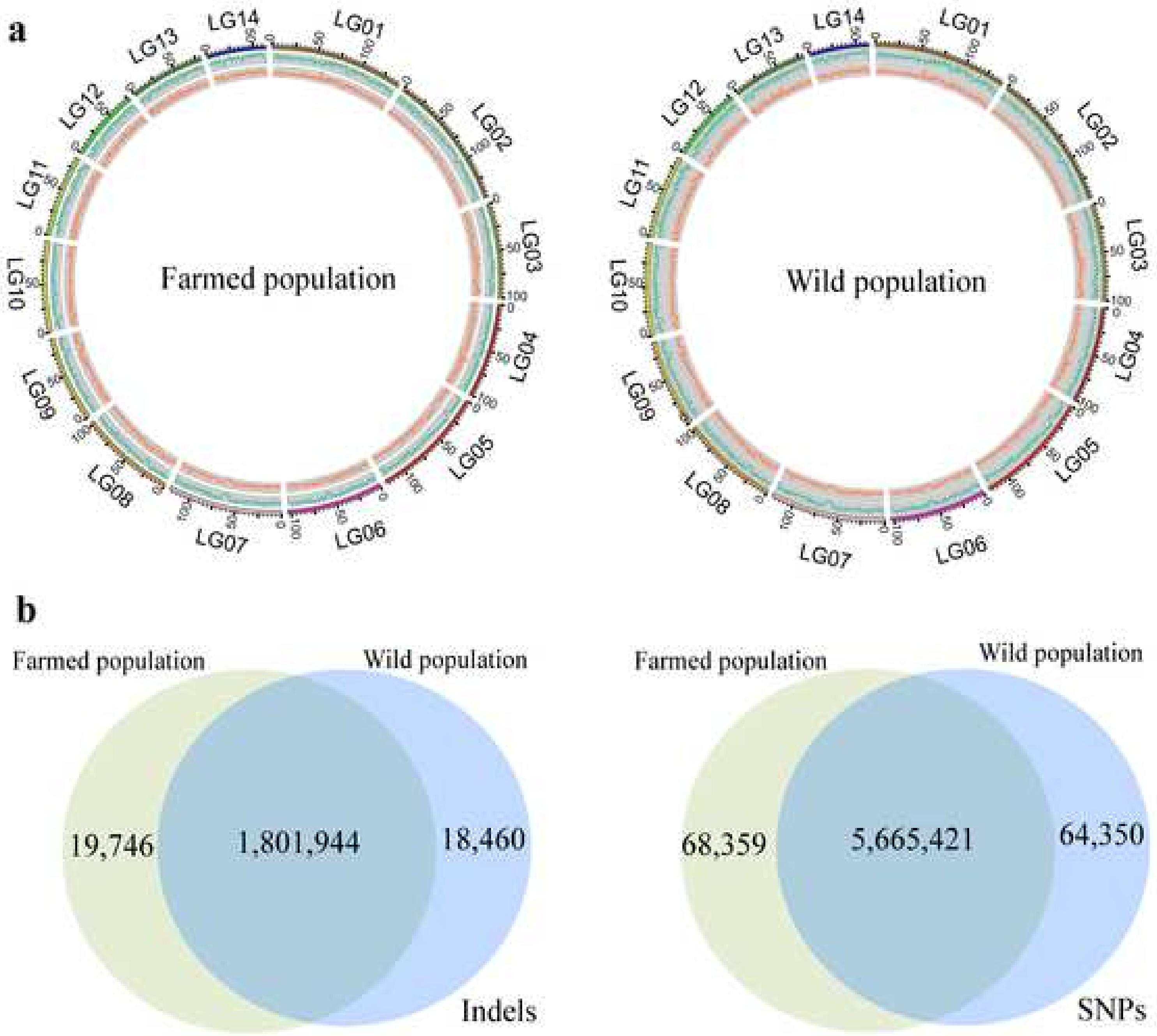

Figure 5. Chromosome synteny.

Click here to access/download;Figure;Fig5 Chromosome synteny.tif

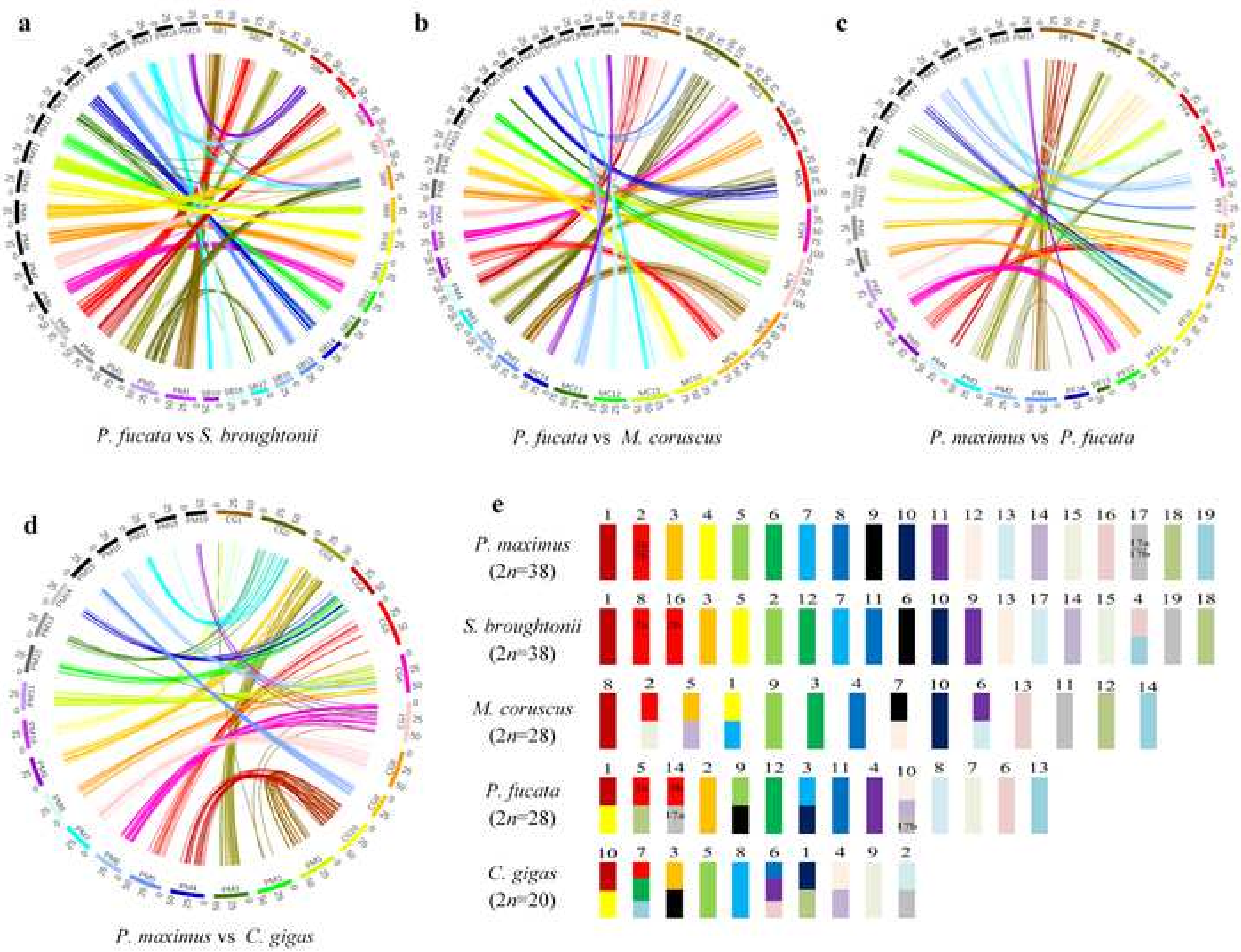

Figure 6. Spatial and temporal expression of genes involved development and metamorphosis.

Click here to access/download;Figure;Fig6 Adrenergic signaling.jpg

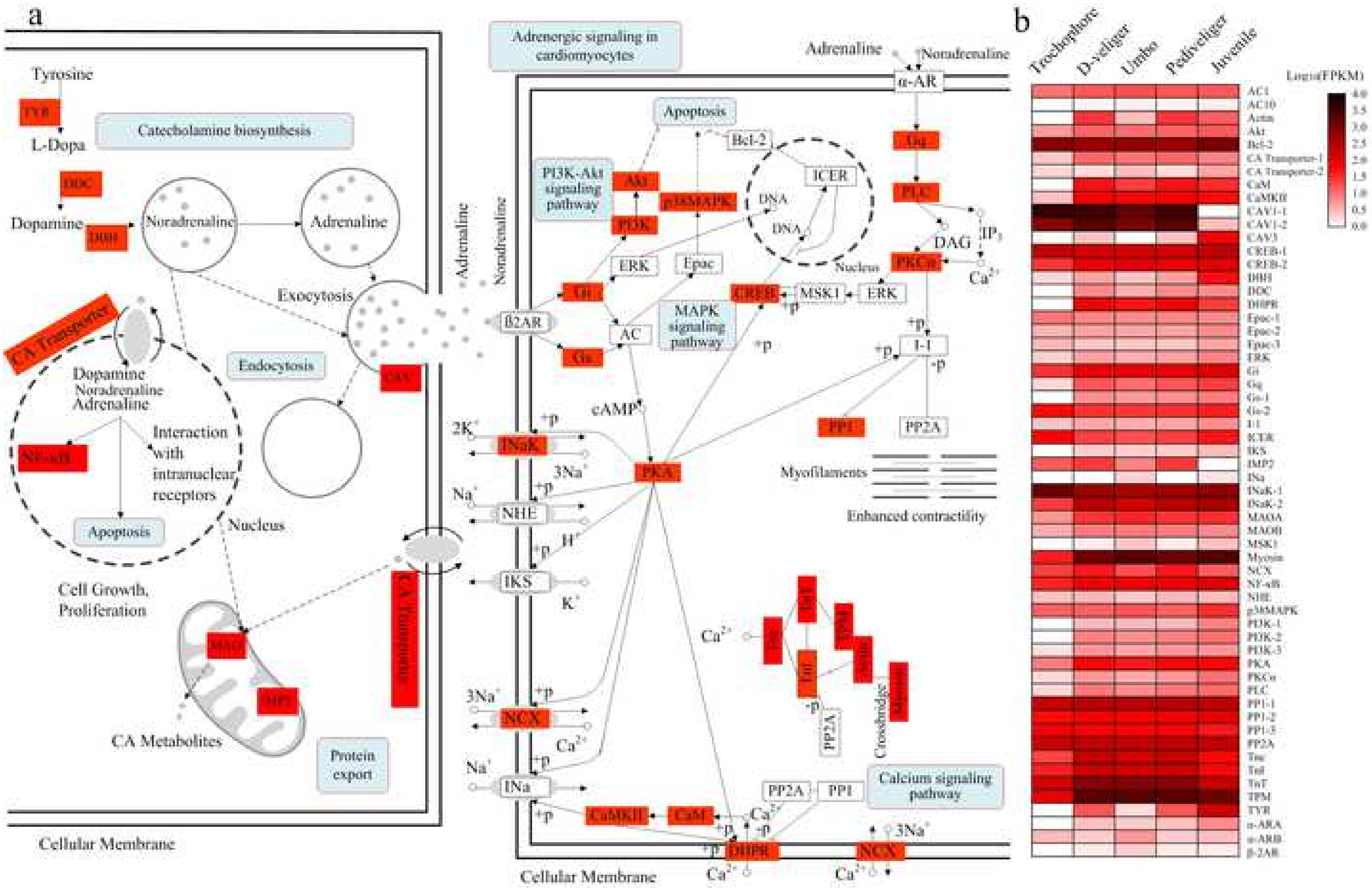

Supplementary Table S1. Repetitive sequences in the hard-shelled mussle genome

Click here to access/download
Supplementary Material
Supplementary Table S1 Repetitive sequences.xls

Supplementary Table S2. Overview of predicted non-coding RNAs

Click here to access/download
Supplementary Material
Supplementary Table S2 non-coding RNAs.xls

Supplementary Table S3. KEGG annotation information of the genes carrying population-specific SNP/INDELs

Click here to access/download
Supplementary Material
Supplementary Table S3 genes carrying specific variations.xlsx

Supplementary Table S4. Quantified gene expressions normalized to FPKM values

Click here to access/download

Supplementary Material

Supplementary Table S4 The gene expression.xlsx

Cover letter Click here to access/download;Personal Cover;Cover letter.docx

Dear Editor,

We would like to submit the enclosed manuscript entitled "A chromosome-level genome assembly of the hard-shelled mussel *Mytilus coruscus* distributed in East Asia seas", which we wish to be considered for publication in GigaScience. All of the authors agree to the submission of this paper. We certify that the submission is original work and is not under review at any other publication.

As an important commercial bivalve, the hard-shelled mussel is widely distributed the temperate seas of East Asia, crossing East China Sea, Bohai Sea, Yellow Sea, and Sea of Japan. Most of the studies in this species (e.g. the planktonic-sessile transition mechanism, population diversity, biofouling/antifouling studies) are faced with the problem of lacking the reference genome, which makes it difficult to figure out the molecular mechanism. Hence, we firstly report a chromosome-level genome of the hard-shelled mussel using a combined strategy of Illumina, ONT and Hi-C technologies. The final 1.57 Gb assemblies have a contig N50 length of 1.49 Mb, of which over 90.0% are anchored on 14 chromosomes. Completeness of the genome with BUSCO test exhibits around 92%. Noticeably, we take advantage of this reference genome to perform three biological tries in study of chromosome evolution, whole genome re-sequencing of farmed and wild populations and transcriptome profiling of larvae metamorphosis. The identified diverse chromosome rearrangements by synteny suggest a complex evolutionary history in the bivalve chromosomes. Whole genome re-sequencing between farmed and wild populations reveals association of sequence variations with shell divergence during the development. Analysis of RNA-Seq data from differernt developmental stages concluded that activation of signal transduction at very early stages is important to metamorphosis in mussel.

The hard-shelled mussel genome will provide novel insights into mussel genome evolution, enhancing our efforts to search for new genes in future mussel planktonic-sessile transition, genetic diversity and breeding programs. We hope this paper is suitable for GigaScience.

The following is a list of possible reviewers for your consideration:

1) Prof. Deborah M. Power E-mail: dpower@ualg.pt

Centre of Marine Sciences, Universidade do Algarve, Campus de Gambelas, 8005-139 Faro, Portugal

2) Prof. Shi Wang E-mail: swang@ouc.edu.cn

Key Laboratory of Marine Genetics and Breeding (Ministry of Education), Ocean University of China, Qingdao 266003, China.

3) Prof. Jian-Wen Qiu E-mail: qiujw@hkbu.edu.hk

Department of Biology, Hong Kong Baptist University, Kowloon, Hong Kong

We deeply appreciate your consideration of our manuscript, and we look forward to receiving comments from the reviewers. If you have any queries, please don't hesitate to contact us at the address below.

Best regards.

Yours sincerely,

Jin-Long Yang E-mail: jlyang@shou.edu.cn

Ying Lu E-mail: yinglu@shou.edu.cn

College of Fisheries and Life Science, Shanghai Ocean University, Shanghai, China

No. 999 Hucheng Huan Road, Pudong New Area, Shanghai
