# Supplementary material for: Chromosome-level genome assembly of the hard-shelled mussel Mytilus coruscus, a widely distributed species from the temperate areas of East Asia
Source: Gigascience. 2021 Apr 23;10(4):giab024. doi: 10.1093/gigascience/giab024 (PMC8063583; doi:10.1093/gigascience/giab024)
Supplement: giab024_GIGA-D-20-00287_Revision_1 [file giab024_giga-d-20-00287_revision_1.pdf]

GigaScience

Chromosome-level genome assembly of the hard-shelled mussel Mytilus coruscus, a widely distributed species from the temperate areas of East Asia

--Manuscript Draft--

**Manuscript Number:**	GIGA-D-20-00287R1
**Full Title:**	Chromosome-level genome assembly of the hard-shelled mussel Mytilus coruscus, a widely distributed species from the temperate areas of East Asia
**Article Type:**	Data Note
**Funding Information:**	

Funder	Recipient
the Natioanl Key Research and Development Program of China (2018YFD0900601)	Doctoral Yi-Feng Li
International Science and Technology Cooperation Programme (CN) (2018YFD0900101)	Professor Ying Lu
the National Natural Science Foundation of China (41876159)	Professor Jin-Long Yang
the National Natural Science Foundation of China (41606147)	Doctoral Xiao Liang
the National Natural Science Foundation of China (31802321)	Doctoral Yi-Feng Li
Guangdong Key Laboratory of Fermentation and Enzyme Engineering (CN) (GML2019ZD0402)	Professor Jin-Long Yang
the Shanghai Sailing Program (18YF1410000)	Doctoral Yi-Feng Li
China Postdoctoral Science Foundation (2019M6614770)	Doctoral Dan-Dan Feng
Program for study on genetic resources, environment and strategy of mussel culture in coast of Gouqi Island offshore	Professor Jin-Long Yang

Abstract:

Background: The hard-shelled mussel (Mytilus coruscus) is widely distributed in the temperate seas of East Asia, and is an important commercial bivalve in China. Chromosome-level genome information of this species will not only contribute to the development of hard-shelled mussel genetic breeding, but also to studies on larval ecology, climate change biology, marine biology, aquaculture, biofouling, and antifouling. Findings: We applied a combination of Illumina sequencing, Oxford Nanopore Technologies sequencing, and high-throughput chromosome conformation capture technologies to construct a chromosome-level genome of the hard-shelled mussel, with a total length of 1.57 Gb and a median contig length of 1.49 Mb. Approximately 90.9% of the assemblies were anchored to 14 linage groups. Comparison to the Core Eukaryotic Genes Mapping Approach (CEGMA) metazoan complement revealed that the genome carried 91.9% of core metazoan orthologs. Gene modeling enabled the annotation of 37,478 protein-coding genes and 26,917 non-coding RNA loci. Phylogenetic analysis showed that M . coruscus is a sister taxon to the clade including Modiolus philippinarum and Bathymodiolus platifrons . Conserved chromosome synteny was observed between hard-shelled mussel and king scallop, suggesting that this is shared ancestrally. Transcriptomic profiling indicated that the pathways of catecholamine biosynthesis and adrenergic signaling in cardiomyocytes might be involved in metamorphosis. Conclusions: The chromosome-level assembly of the hard-shelled mussel genome will provide novel insights into mussel genome evolution and serve as a fundamental platform for studies regarding the planktonic-sessile transition, genetic diversity, and genomic breeding of this bivalve.

Corresponding Author:

Ying Lu
Shanghai Ocean University
Shanghai, CHINA

Powered by Editorial Manager® and ProduXion Manager® from Aries Systems Corporation

Corresponding Author Secondary Information:	
Corresponding Author's Institution:	Shanghai Ocean University
Corresponding Author's Secondary Institution:	
First Author:	Jin-Long Yang
First Author Secondary Information:	
Order of Authors:	Jin-Long Yang
	Dan-Dan Feng
	Jie Liu
	Jia-Kang Xu
	Ke Chen
	Yi-Feng Li
	You-Ting Zhu
	Xiao Liang
	Ying Lu
Order of Authors Secondary Information:	
Response to Reviewers:	RESPONSE LETTER<br>Ying Lu, January 2021<br>Dear Dr. Hans Zauner<br>Giga Science<br><br>Manuscript GIGA-D-20-00287R1.<br><br>Dear Reviewers and Editor, thank you for your time and valuable help in improving this manuscript. Please find below our detailed response letter (answers in blue) addressing in the comments.<br><br>Reviewer Comments:<br>Reviewer #1:<br><br>The manuscript by Yang and colleagues reports a high quality genome assembly for the mussel Mytilus coruscus. Although this is not the first genome assembly published for this species, this resource is an improvement compared with the previous version, due to the use of Hi-C libraries and a better management of heterozygous genomic regions. Hence, the contents of this work appear to be appropriate for a data note article. there are however several points that would require some additional information to be added, and bits of text that need to be modified to improve the flow of the text.<br>Dear reviewer, thank you for your comments and suggestions to improve this manuscript. As requested, we included more information about PAV and the genomic coverage, improved the language by a native English speaker, and made other corrections as suggested.<br><br>General comments:<br>I would suggest the authors to specify the sequencing coverage achieved somewhere in the text (i.e. which coverage was obtained with ONT reads? Which coverage was obtained with Illumina PE? Etc.). This is present in Table 1, but it should be also mentioned in the text.<br>Thanks for your suggestion, we have specified the sequencing coverage in the text (Line 127, Line 134 and Line 136).<br><br>The authors emphasized the high heterozygosity of the genome, pointing out the possible links between SNPs and phenotypic variation. The authors may not be aware of the very recent discoveries that currently indicate that bivalve genomes are characterized by significant hemizygosity and structural variants that affect gene

content, resulting in massive gene presence/absence variation. While the authors are not currently required to update this work with a detailed analysis of PAV, I think the text might benefit from some additional points of discussion, especially considering the fact that a congeneric mussel species, M. galloprovincialis, has been shown to be characterized by an astounding level of intraspecific genomic variation (see the preprint by Gerdol et al. 2019, which has recently been accepted for publication and should become available online in the matter of a few weeks on genome Biology). Also see the preprint by Calcino and colleagues here:
https://www.biorxiv.org/content/10.1101/2020.09.15.298695v1
Thanks for your suggestion. We have checked two related papers to understand the hemizygosity and PAV as described. The papers discovery that bivalve genomes are characterized by significant hemizygosity and structural variants that affect gene content. We cite that reference of the PAV in Line 225-226 and Line 277-279 as “which might be owing to the widespread hemizygosity and massive gene presence/absence variation (PAV) (Gerdol et al. 2020; Calcino et al. 2020)” and “In addition, PAV may play a role in determining phenotypic traits (Gerdol et al. 2020; Calcino et al. 2020), which should be included in the future re-sequencing analyses.”
Reference:
Gerdol M, Moreira R, Cruz F, et al. Massive gene presence-absence variation shapes an open pan-genome in the Mediterranean mussel. Genome Biology 2020; 21:275.
Calcino AD, Kenny NJ, Gerdol M. Single individual structural variant detection uncovers widespread hemizygosity in molluscs. bioRxiv 2020; 298695.

In general, I would recommend the authors to involve a native English-speaking colleague in the revision of the text, as several grammar errors and oddly constructed sentences are present throughout the text.
Thanks for your suggestion. The revised manuscript has been professionally edited by a native English-speaking colleague. The main alternations are highlighted in the revision.

Abstract
1.4: -correct "high-through"; I guess the authors were referring to "high throughput" here.
We replaced “high-through” with “high throughput” (Line 28).

1.5:-Correct "platifron" with "paltifrons"
We revised “platifron” into “paltifrons” (Line 36).

1.6:-"speculating their sharing same origins in evolution" please correct this odd wording.
Sorry for the confusion, we have corrected the sentence as “suggesting that this is shared ancestrally” (Line 38).

List of detailed comments
1.7:-Mussels have been also used as sentinel organisms for biomonitoring, and this information could be added to the list
Thanks for your suggestion, we have added biomonitoring in Line 54.

1.8:-"As with a dozen of marine invertebrates". This is unclear; I guess the authors meant "As several other marine invertebrates"
We rewrote the sentence (Line 61), as follows:
“As many other marine invertebrates, marine mussels also possess a free-swimming larval phase.”

1.9:-When talking about the M. galloprovincialis genome assembly, the authors only refer to the paper by Murgarella and colleagues, whereas an improved version has been recently accepted for publication on Genome Biology (this should be probably available online within a few weeks). The text is available as a preprint, see Gerdol et al. https://www.biorxiv.org/content/10.1101/781377v1
Thanks for your notification, we added the reference of an improved genome of M. galloprovincialis by Gerdol et al (Line 80).

Methods
1.10:-This section in particular suffers from the presence of several issues with the

	quality of the language used, that should be improved.<br>Thanks for your suggestion, we have revised this section to improve the language by a native English speaker.<br><br>1.11:-When talking about the k-mer graph, please refer to the homozygous and heterozygous peaks (instead of "junior peak"<br>Thanks for your comment. The homozygous and heterozygous peaks are clarified in the revision (Line 152-154). Calculation of the k-mer occurrence is improved using GenomeScope.<br><br>1.12:-"very close to the total assemblies (1.57 Gb)". I think it would be worth mentioning that this is also not far from the c-value previously estimated by cytogenetic studies (see Ieyama, H., O. Kameoka, T. Tan, and J. Yamasaki (1994). Chromosomes and nuclear DNA contents of some species of Mytilidae. Venus 53: 327-331)<br>Thanks for your notification. We added the reference of the genome size estimated by cytogenetic studies (Ieyama et al. 1994) (Line 156).<br>Reference:<br>Ieyama H, Kameoka O, Tan T, et al. Chromosomes and nuclear DNA contents of some species in Mytilidae. Venus (Japanese Journal of Malacology) 1994; 53:327-331.<br><br>1.13:-"which is much greater than the real size of 1.57 Gb". This is also in line with what has been observed for M. galloprovincialis by Gerdol et al.<br>Yes. The same observation are added in Line 158-160, as follows:<br>“This kind of over-estimation for genome size usually occurred to the fragmented assemblies, like the recently published M. galloprovincialis genome, in which considerable heterozygous redundancies seem to be included in the assemblies.”<br>Reference:<br>Gerdol M, Moreira R, Cruz F, et al. Massive gene presence-absence variation shapes an open pan-genome in the Mediterranean mussel. Genome Biology 2020; 21:275.<br><br>1.14:-"The yielded consensus sequences were manually checked by aligning to the GenBank database". This is a clever strategy, but I think it should be explained a bit better here.<br>Thanks. We revised the sentence as "The yielded consensus sequences were manually checked by aligning to the genes from the GenBank database (nt and nr; released in October 2019) to avoid that sequences of the high-copy genes are masked in following process with RepeatMasker".<br><br>1.15:-"which was less than previously-published 42,684 gene models in the draft genome because it introduced over 20% heterozygous redundancies in the assemblies". I agree with this consideration, but in light worth the recent findings about widespread hemizygosity and massive gene presence/absence variation in M. galloprovincialis, the authors might want to update the text with a few additional considerations.<br>We agree with that widespread hemizygosity and massive gene PAV probably cause the redundancies since it has been identified in M. galloprovincialis, as well as other molluscs (Gerdol et al. 2020; Calcino et al. 2020), as follows (Line 222-226):<br>"Using a bidirectional BLASTp between the two assemblies, we observed that an considerable heterozygous redundancies (over 20%) were probably included into the previous draft assemblies (Supplementary Table 3), which might be owing to the widespread hemizygosity and massive gene presence/absence variation (PAV) (Gerdol et al. 2020; Calcino et al. 2020) or assembling errors."<br>Reference:<br>Gerdol M, Moreira R, Cruz F, et al. Massive gene presence-absence variation shapes an open pan-genome in the Mediterranean mussel. Genome Biology 2020; 21:275.<br>Calcino AD, Kenny NJ, Gerdol M. Single individual structural variant detection uncovers widespread hemizygosity in molluscs. bioRxiv 2020; 298695.<br><br>1.16:-"448 single-copy genes". How were such genes identified? Was BUSCO/OrthoDB used for this?<br>Sorry for your confusion, we used OrthoDB to find the single-copy gene (Line 237).<br><br>1.17:-"Whole genome re-sequencing of farmed and wild individuals". The data

Powered by Editorial Manager® and ProduXion Manager® from Aries Systems Corporation

provided here are potentially interesting for a preliminary analysis, but the authors should keep in mind (and briefly discuss) the possibility that higher-order structural variants which include gene PAV might have a very important role on phenotypic traits.
Thanks for your notification, we have included the PAV in the discussion in whole genome re-sequencing, as follows:
"In addition, PAV may play a role in determining phenotypic traits (Gerdol et al. 2020; Calcino et al. 2020), which should be included in the future re-sequencing analyses."
Reference:
Gerdol M, Moreira R, Cruz F, et al. Massive gene presence-absence variation shapes an open pan-genome in the Mediterranean mussel. Genome Biology 2020; 21:275.
Calcino AD, Kenny NJ, Gerdol M. Single individual structural variant detection uncovers widespread hemizygosity in molluscs. bioRxiv 2020; 298695.

1.18:-"consistent with their closest phylogenetic relationship in the Bivalvia clade" please add a reference for this.
Thanks. We add the Reference (Liu et al. 2020) for this.
Liu F, Li Y, Yu H, Zhang L, Hu J, Bao Z, Wang S. MolluscDB: an integrated functional and evolutionary genomics database for the hyper-diverse animal phylum Mollusca. Nucleic Acids Res. 2020 Nov 21:gkaa1166. doi: 10.1093/nar/gkaa1166. Epub ahead of print. PMID: 33219684.

1.19:-It would have been more appropriate to use TPM instead of FPKM ? ? , as this metric allows a more reliable comparison among samples.
Thanks for your suggestion, we use TPM instead of FPKM in transcriptome analysis (Supplementary Table S4, Line 318-320).

1.20:-"indicative of a 91.9% genome completeness when 89.98% of core metazoan orthologs were completely identified in the assemblies." This is somewhat unclear. Does 91.9% indicate present BUSCOs and 89.98% "present and complete"? Adding specific information concerning fragmented BUSCOs and duplicated or missing BUSCOs would help here.
As your suggested, we revised the sentence as "We assayed the genome completeness using Benchmark Universal Single-Copy Orthologs BUSCO v4.1.4 referencing metazoan and molluscan gene sets. In the metazoan dataset, the current assemblies have 89.4% complete (of which 1.0% were duplicated), 1.9% incomplete and 8.7% missing BUSCOs, corresponding to a recovery of 91.3% of the entire BUSCO set. In the molluscan dataset, 85.5% complete (of which 1.3% were duplicated), 0.8% incomplete and 13.7% missing BUSCOs were recorded, corresponding to 86.3% of the entire BUSCO set."

Reviewer #2: Yang et al present the genome assembly of the hard-shelled mussel Mytilus coruscus, alongside gene predictions and analysis of genome content. The work in assembling the genome and predicting genes is technically sound. However, the presentation of this work is inprecise, not yet of publishable standard, and would benefit from careful editing for science and language before re-submission. There are also several scientific points that need to be addressed, to ensure that the claims made in the manuscript are proportionate to the evidence presented. I have noted these below.

Major points to address:
2.1: 1) The authors claim that their genome represents a chromosome-level assembly of the genome of this species. This claim is based on the combination of reasonably long contigs into scaffolds using Lachesis based on linkage. To be able to firmly claim that these represent a "chromosome level assembly" it is necessary to evaluate the degree to which these pseudomolecules are assembled. Table 2 should provide data on the extent of gaps (total Ns) in each chromosome, and in the text, the size distribution of gaps, and information about them, should be noted. Are these, for instance, estimated and set at 100/1000 Ns? or are these a true reflection of the gap size? Is there any evidence of telomeric sequence at each end?
Thanks so much for your suggestions, we list the length (Ns) in the extents of the gaps (Table 2) in the revision. All of the gaps are set at 100 Ns, not the true reflection of the gap size. Total length of the gaps is 201.5 kbps (filled with 201.5 kbp Ns; Table 2; Line 180-181). We detect the characteristic motifs of telomeric sequences in 23 termini of the 13 chromosomes, suggesting the completeness of the assemblies (Supplementary

Powered by Editorial Manager® and ProduXion Manager® from Aries Systems Corporation

Table S6; Line 359-361).

2.2: 2) There is a stark difference in estimated genome size between the previously published genome for this species and this resource. It would be useful to map the previous (draft) assembly of Li et al to this assembly and determine what percentage of the huge missing fragment (21%) of that assembly is truly missing from this assembly, and why. Does this represent uncollapsed heterozygosity (which would map twice to the same loci, presumably), intraspecific hemizygosity variation, or contamination in the previous genome resource? Or is it perhaps a problem of missing data in the assembly presented here? Any of these answers would be useful for understanding the genome of this species.
This is the good point. When the previous assemblies are aligned to present ones, a total of 141.8 Mb of genome sequences duplicates in the previous version while only 49.7 Mb in this resource (mapping rate of the Illumina reads against our assemblies was over 96.7%), indicating more heterozygous redundancies in the previous drafts. As far as the intraspecific hemizygosity variation reported in the M. galloprovincialis genome and other molluscan genomes (Gerdol et al. 2020; Calcino et al. 2020) is concerned, we do not have the evidence to clarify whether intraspecific hemizygosity variation results in different sizes of the assemblies. However, this reference is cited in the revision to demonstrate that over-estimation of the genome size sometime occurred to the draft assemblies (Line 158-160). In addition, comparative analysis of gene models also suggests considerably heterozygous duplicates in the previously published drafts (see response to 2.4).
Reference:
Gerdol M, Moreira R, Cruz F, et al. Massive gene presence-absence variation shapes an open pan-genome in the Mediterranean mussel. Genome Biology 2020; 21:275.
Calcino AD, Kenny NJ, Gerdol M. Single individual structural variant detection uncovers widespread hemizygosity in molluscs. bioRxiv 2020; 298695.

2.3: 3) Genome size estimation is carried out by a mathematical derivation directly from the highest peak size. This, however, partially excludes from consideration the heterozygous portion of the genome. As the assembly has been polished with Racon and Pilon, heterozygosity could also be underestimated by mapping estimates. It is recommended that alternate genome size estimates are provided. Genomescope (http://qb.cshl.edu/genomescope/genomescope2.0/) is a simple-to-use option that will provide more nuanced information regarding genome size and heterozygosity.
As you suggested, we re-estimated the genome size and heterozygous rate in the revision using Genomescope. The assessment of genome size by K-mer counting using GenomeScope suggested a complete genome size of approximately 1.51 Gb (Fig. 3a) (Line 149-151; Line 154-156). The present genome had a heterozygous rate of 1.39 %, calculated by GenomeScope (Line 167-168).

2.4: 4) how many of the gene models found in Li et al are present/absent from the final gene set presented here? Were these used in the EVidenceModeler merge step? It is noted that "37,478 final gene models were generated (Table 3), which was less than previously-published 42,684 gene models in the draft genome because it introduced over 20% heterozygous redundancies in the assemblies". Please provide more information on how this was determined, as these extra genes could also represent recent duplicates, which should not be removed from consideration. This could build upon the results of 2) above.
The previous draft genome reported that the protein-coding gene set consists of 42,684 models (Li et al. 2020). However, we find 58,540 genes uploaded in GenBank, which is consistent with the gene numbers in their gff file. We compare the constructed gene families between the previous version and our annotations, using their 58,540 genes and our 37,478 genes. The gene duplicates are identified in the gene clusters of the two assemblies (see the following Table), in which A for the previously published genomes; B for the genome assemblies in this study. Quantity of the A-specific gene clusters that only consist of the genes from the previously published genome is significant higher than the B-specific ones that only consist of the genes from the assemblies in this study. Alignments against the NR database and repeat sequence library exhibits that 12,123 A-specific gene clusters (20.71% of 58,540) are annotated as transposable elements. The genes clustered in the families with more A members is much more than those in the families with more B members. We also find some genes with the same loci, splicing and even intron sequences. All of the information reflected

a significant over-estimation in both genome size and quantity of protein-coding genes (Line 222-226).
Supplementary Table S3. Bidirectional BLASTp between the previously published gene models of the hard-shelled mussel and the predicted gene models in this study.
Relationship type of gene members in each familyQuantity of gene families
(gene numbers in brackets)
Published draft assemblies (A)Assemblies in this study (B)
One to one15,265 (15,265)15,265 (15,265)
One (A) to many (B)281 (281)281 (780)
Many (A) to one (B)3,531 (10,781)3,531 (3,531)
Many to many541 (2,904)541 (1,556)
A = B180 (413)180 (413)
A > B327 (2,369)327 (889)
A < B34 (122)34 (254)
Unique (only A or B)3,569 (12,154)538 (1,688)

Reference: Li RH, Zhang WJ, Lu JK, et al. The whole-genome sequencing and hybrid assembly of Mytilus coruscus. Frontiers in Genetics 2020; 11:1-6.

2.5: 5) The phylogeny as presented needs further consideration. Was concatenation of genes performed before alignment? (page 11) This could introduce errors at the start and end of each gene as they can artifactually be aligned to non-homologous sequences. This should be checked, and repeated correctly if necessary (with alignment performed gene-by-gene, then concatenating the alignments). -What maximum likelihood model was used? what other settings? how many bootstraps? Please note in text (page 11). -How was divergence time calculated?
Sorry for the confusion. Alignment of one-to-one single copy genes is prior to concatenation of the alignments. The corresponding sentences “448 single-copy genes identified by OrthoDB were aligned and concatenated. The amino acid sequences were first aligned using MUSCLE, which were further concatenated to create one supergene sequence for each species and formed a data matrix” (Line 236-239) are corrected in the revision.
Line240-246: The phylogenetic relationship was constructed using the Maximum-likehood model in RAxML version 8 with the optimal substitution model of PROTGAMMAJTT. Robustness of the maximum-likelihood tree was assessed using the bootstrap method (100 pseudo-replicates). Furthermore, the single-copy orthologs and one reference divergence time on the root node obtained from TimeTree database (http://www.timetree.org) were used to calibrate the divergence dates of other nodes on this phylogenetic tree by MCMCTREE tool in PAML package.

2.6: 6) In the "Whole genome re-sequencing of farmed and wild individuals" section, the assumption that sequence variations are farmed- population-specific (FPS) or wild-population-specific (WPS) is flawed as it is based on a tiny sample (20 individuals) of the enormous diversity of this species. It is not convincing to claim that these variants are unique to either farmed populations or wild populations - they are just observed to be different here due to the limited sampling. The depth of sequencing is also very low per individual (around 2.5x) and SNPs/indels could be missed. This section, and the claims made from it in the abstract and conclusions, need to be substantially reworked to avoid drawing universal conclusions from what are only initial pilot results.
Thanks for your suggestions. We re-write this section and weaken the claims made from it in the abstract and conclusion since this is just a preliminary try in the genome study. A simple case is added in the revision to illustrate the diversities between farmed and wild populations. We only make a brief speculation that sequence variation might be associated with morphological diversity (Line 276-277).

2.7: 7) The differential expression analysis in larvae is not convincing. Many of the genes cherry-picked for discussion and shown in Fig 6 are expressed in all samples. As only single libraries were sequenced for each larval life stage, claims for differential expression are only very weakly supported. It is good practice to use a minimum of 3 separate samples per condition for DE analysis, and preferentially more. The authors should moderate the strength of the conclusions drawn in the "Transcriptome related to metamorphosis" section considerably, in light of the strength of some of the evidence presented.
Thanks for your suggestion. We have used 3 biological replicates’ RNA-Seq data of

Powered by Editorial Manager® and ProduXion Manager® from Aries Systems Corporation

five developmental stages (SRR13364385、SRR13364374、SRR13364373、SRR13364371、SRR13364370、SRR13364369、SRR13364368、SRR13364367、SRR13364383、SRR13364382、SRR13364381、SRR13364380、SRR13364378、SRR13364377、SRR13364376) to analyze the differential expression with normalized gene expression levels TPM (Line 317-319). We moderated the conclusion by removing the strong claims as “Signal transduction controlling the metamorphosis development seemed to activate during the first two stages, trochophore and D-veliger, although the major morphologic changes represented in the transition from pediveliger to juvenile”.

Minor points:

2.8: -The authors are often too strong in their criticism of the earlier genomes for this species and Mytilus. For instance "a low quality draft genome of M. coruscus has been reported" (pg 4). That resource is not as well-contiged, but saying it is low quality is not justified. Perhaps "Draft versions of the genomes of M. coruscus and M. galloprovincialis have been reported". This kind of strong claim should be toned down throughout the manuscript.
We deleted “a low quality” and corrected the sentence as “a draft genome of M. coruscus and an improved genome of M. galloprovincialis have been reported” (Line 79-80).

2.9: - Many of the steps shown in Fig 2 (e.g. read cleaning) are not covered in sufficient detail in the manuscript. Please ensure that the steps required to recapitulate this work are provided.
Thanks. We added the details to describe the steps in Fig 2, as follows:
Line 143-146: The raw reads from Illumina sequencing platform were cleaned using FastQC45 and HTQC46 by the following steps: (a) filtered reads with adapter sequence; (b) filtered PE reads with one reads more than 10% N bases; (c) filtered PE reads with any end has more than 50% inferior quality (≤5) bases.
Line 189-192: The yielded consensus sequences were manually checked by alignment to genes from the GenBank database (nt and nr; released in October 2019) to avoid that sequences of the high-copy genes are masked in following treatment with RepeatMasker.
Line 236-246: Gene clusters were identified among 12 selected genomes … calibrate the divergence dates of other nodes on this phylogenetic tree using the MCMCTREE tool in the PAML package.

2.10: -What settings were used for OrthoMCL?
The settings used for OrthoMCL are a BLASTp value cutoff of 1e−5 and an inflation parameter of 1.5 (Line 236).

2.11: -What settings were used to detect PCR duplicates with Picard?
Thanks for your notification, the duplicate reads were removed with the MarkDuplicates tool of Picard (Line 257-258).

2.12: Fig 3d: caniculata seems to be mis-spelled
Thanks for your notification, we have corrected “canaliculate” into “canaliculata” in Fig 3d.

2.13: Fig 5: Why is P. fucata highlighted? Why not show P. maximus vs Mytilus coruscus? It is the most relevant for this paper. Fig 5a and Fig 5b might be the wrong images?
We illustrate the chromosome synteny of P. maximus vs S. broughtonii, P. maximus vs M. coruscus, P. maximus vs P. fucata, and P. maximus vs C. gigas (Figure 5). We did not highlight P. fucata. Given that the genome of P. maximus was reported to be a slow-evolving genome with many ancestral features, the P. maximus is selected as a reference to compare with other four chromosome-level bivalves.

Note on language and scientific accuracy:
2.14: Throughout the manuscript there are minor errors in written english, which regularly introduce scientifically inaccurate statements. I have noted some of these below but my list is not complete, and the authors may wish to have their manuscript read over more thoroughly before resubmission. I have not had the time to correct all

Powered by Editorial Manager® and ProduXion Manager® from Aries Systems Corporation

	the errors present in the manuscript.<br>Thanks for your suggestion. The revised manuscript has been professionally edited by a native English-speaking colleague. The main alternations are highlighted in the revision.<br><br>2.15: Throughout: Please refer to the species name or the common name, but not "marine mussel" when you mean Mytilus coruscus - most mussels are marine. Similarly, do not use this to refer to all mussels.<br>Thanks for your notification, we have referred to the common name in revision (Line 41).<br><br>2.16: Title: the authors should consider introducing a comma into their title, breaking it into precise units: e.g. "A chromosome-level genome assembly of the hard-shelled mussel Mytilus coruscus, a widely distributed species from temperate areas of East Asia"<br>As your suggested, we corrected the title as "Chromosome-level genome assembly of the hard-shelled mussel Mytilus coruscus, a widely distributed species from the temperate areas of East Asia"<br><br>Abstract:<br>2.17: -no "A" in : A chromosome-level genome information<br>Thanks for your notification, we have deleted "A" in : A chromosome-level genome information (Line 24).<br><br>2.18: -high-through - do you mean high-throughput?<br>Thanks for your notification, we have corrected “high-through” as “high-throughput” (Line 28).<br><br>2.19: -" The completeness test exhibits" - I think you mean "comparison to the CEGMA metazoan complement reveals"<br>Thanks for your notification, we have corrected as "Comparison to the Core Eukaryotic Genes Mapping Approach (CEGMA) metazoan complement revealed" (Line 28).<br><br>2.20:-No "The" in "The phylogenetic analysis"<br>Thanks for your notification, we have revised "The phylogenetic analysis " into " Phylogenetic analysis " (Line 35).<br><br>2.21:-"the closest relationship between" - this is not true. I think you mean "phylogenetic analysis shows M. coruscus is the sister taxon to the clade comprised of Modiolus philippinarum and Bathymodiolus platifrons". Note spelling of last species<br>Thanks for your notification, we have revised the describtion of " the closest relationship between " into " Phylogenetic analysis showed that M. coruscus is a sister taxon to the clade including Modiolus philippinarum and Bathymodiolus platifrons. ", and we have corrected "Bathymodiolus paltifrons " into "Bathymodiolus platifrons " (Line 35-36).<br><br>2.22:-No "A", in "A conserved chromosome synteny "<br>Thanks for your notification, we have deleted "A" in "A conserved chromosome synteny" (Line 36).<br><br>2.23:-"speculating their sharing same origins in evolution" do you mean "suggesting that this is shared ancestrally"? Because the former is contentious<br>Thanks for your notification, we have corrected the sentence as "suggesting that this is shared ancestrally" (Line 38).<br><br>2.24:-no on in "studying on"<br>Thanks for your notification, we have deleted "on" in "studying on" (Line 42).<br><br>Context:<br>2.25:-phylum Mollusca (not Mollusc).<br>Thanks for your notification, we have corrected "Mollusc" as "Mollusca" (Line 47).<br><br>2.26:-"sea mussels". This is an inprecise phrase. Perhaps just use "mussels"<br>Thanks for your notification, we have revised "sea mussels " into "mussels" (Line 49).

2.27:- " Although their significance" - should read "Although they are significant for biology, ecology and the economy"
Thanks for your notification, we have revised " Although their significance in biology, ecology and economy " into " Although they are significant for biology, ecology and the economy " (Line 56-57).

2.28:- need an "and" before ", settlement mechanism."
Thanks for your notification, we have add an "and" before "settlement mechanism" (Line 60).

2.29:-"As with a dozen of marine invertebrates" - this is a deeply inaccurate statement. Perhaps "As with many marine invertebrates".
Thanks for your notification, we have revised " As with a dozen of marine invertebrates " into " As many marine invertebrates" (Line 61).

2.30:-"modeling of their anatomy " not "modeling of anatomy "
Thanks for your notification, we have revised " modeling of anatomy " into " remodeling of their anatomy " (Line 63).

2.31:-"trigger settlement and metamorphosis is universal in metazoan" - this is not true. Humans, for instance, are metazoans
Thanks for your notification, we have corrected "universal in" as " widespread among " (Line 68).

2.32:- "temperate areas" not "the temperate"
Thanks for your notification, we have revised " the temperate " into " temperate areas " (Line 71).

2.33:-"need adapt..." should read "needs to adapt to the hostile..."
Thanks for your notification, we have revised "need adapt to the hostile" into " needs to adapt to the hostile" (Line 74-75).

2.34:-"Up to date, chromosome level genome" should read "To date, a chromosomal-level genome"
Thanks for your notification, we have revised "Up to date, chromosome level genome" into " To date, no genome of any member of the genus Mytilus " (Line 78).

2.35:-"Lacking whole-genome information" should read "The lack of whole-genome information".
Thanks for your notification, we have revised " Lacking whole-genome information " into " The lack of whole-genome information " (Line 80-81).

2.36:-"The larvaes at five ..." should read "Larvae at five....".
Thanks for your notification, we have revised " The larvaes at five ... " into " Larvae at five.... " (Line 89).
2.36:-"gene expression" not "gene expressions"
Thanks for your notification, we have corrected "gene expressions" into "gene expression" (Line 90).

Methods:
2.38:-"where is the central coast of Chinese mainland" should read "the central coast of the Chinese mainland"
Thanks for your notification, we have revised "where is the central coast of Chinese mainland" into "which is the central coast of the Chinese mainland" (Line 97).

2.39:- "a" needed, A female wild adult with a mature ovary (although these are probably paired but difficult to detect - if paired this would be "with mature ovaries".)
Thanks for your notification, we have added " a " in " mature ovary " (Line 100), which was reported to be a mature ovary in mussel.

2.40:-" for the adductor muscle to isolate high molecular weight genomic DNA for sequencing of reference genome" should read ", with the adductor muscle taken for isolation of high molecular weight genomic DNA, for sequencing of the reference

	genome".<br>As your suggested, we have corrected the sentence as " and the adductor muscle was collected to isolate high-molecular-weight genomic DNA for the sequencing of the reference genome " (Line 101-102).<br><br>2.41:- no s "The DNAs"<br>Thanks for your notification, we have revised " The DNAs " into " The DNA" (Line 102).<br><br>2.42:-" to be assistant " should read "to assist with"<br>Thanks for your notification, we have revised " to be assistant " into " to assist with " (Line 101-106).<br><br>2.43:-" using SDS extraction method," should read " using the SDS extraction method," and a reference to this protocol should be given.<br>Thanks for your notification, we have added " the " in " using the SDS extraction method," and provided the reference (Eugene. 2000) (Line 109-110).<br>Sokolov EP. An improved method for DNA isolation from mucopolysaccharide-rich molluscan tissues, Journal of Molluscan Studies, 2000; 66 (4): 573–575, https://doi.org/10.1093/mollus/66.4.573.<br><br>2.44:-"total RNA were extracted" should read " total RNA was extracted "<br>Thanks for your notification, we have revised " were " into " was " (Line 114).<br><br>2.45:-"as well as the larvaes" should read "as well as larvae".<br>Thanks for your notification, we have corrected "larvaes" as " larvae".<br><br>2.46:"to get large segments " should read "to extract large fragments". fragments should be used instead of segments throughout this section.".<br>Thanks for your notification, we have revised " to get large segments " into " to extract large fragments " (Line 123). And we have corrected " segments " as " fragments ".<br><br>2.47:- The high quality library of average 20 kb in length was sequenced on the ONT PromethION platform with corresponding R9 cell and ONT sequencing reagents kit. The genomic DNA was sequenced using the MinION portable DNA sequencer with the 48 hours run script (Oxford Nanopore), which generated a total of 246.8 Gb data" were both the minion and promethion used? please make this clearer.<br>Sorry for your confusion, we only used PromethION platform and deleted the description of MinION portable DNA sequencer.<br><br>2.48:-" were fragmentized" should read " were fragmented"<br>Thanks for your notification, we have read "were fragmentized " into " was fragmented " (Line 129).<br><br>2.49:-novaseq needs a capital<br>Thanks for your notification, we have corrected " novaseq " as " NovaSeq " (Line 133).<br><br>2.50:- "by poly(A)" should read "for poly(A) transcripts". Which protocol was used?<br>Sorry for your confusion, we described the protocol as " The sample was enriched in mRNA by extracting poly(A) transcripts from total RNA using oligo-d(T) magnetic beads." (Line 138-139).<br><br>2.51:-" in 150 bp paired-end model." should read " in 150 bp paired-end mode."<br>Thanks for your notification, we have revised " in 150 bp paired-end model " into " in 150 bp paired-end mode " (Line 142).<br><br>2.52:-"Genome size of the hard-shelled " needs a "The" before<br>Thanks for your notification, we have revised " Genome size of the hard-shelled " into " The size of the hard-shelled mussel genome" (Line 149).<br><br>2.53: -" Average GC content of genome" needs a the before genome.<br>Thanks for your notification, we have revised " Average GC content of genome " into " an average GC content of genome " (Line 168).

	2.54: -"The final assemblies is around 1.57 Gb" should be "The final assembly is around 1.57 Gb"<br>Sorry for the confusion, we have corrected the grammar (Line 165).<br><br>2.55: -"The genome assemblies of hard-shelled mussel" again should be assembly<br>Thanks for your notification, we have revised " The genome assemblies of hard-shelled mussel " into " The genome assembly of hard-shelled mussel " (Line 172).<br><br>2.56: -"with the softwares of Augustus (version 3.1) [38], GlimmerHMM (version 1.2) [39] and SNAP (version 2006-07-28) " should read "with Augustus (version 3.1) [38], GlimmerHMM (version 1.2) [39] and SNAP (version 2006-07-28) software"<br>Thanks for your notification, we have revised " with the softwares of Augustus (version 3.1) [38], GlimmerHMM (version 1.2) [39] and SNAP (version 2006-07-28) " into " using the Augustus (version 3.1) [38], GlimmerHMM (version 1.2) [39] and SNAP (version 2006-07-28) software " (Line 207-208).<br><br>2.57: -"protein sequences of two closed mollusc species" do you mean two closely related mollusc species?<br>Thanks for your suggestion, we have revised " two closed mollusc species " into "two closely related mollusc species" (Line 209).<br><br>2.58: -"Parallelly," should be "In parallel"<br>Thanks for your notification, we have revised " Parallelly," into "In parallel" (Line 211).<br><br>2.59: -"put into a de novo assemble" should be "assembled de novo"<br>Thanks for your notification, we have revised "put into a de novo assemble " into " assembled de novo " (Line 213).<br><br>2.60: -transnfer mis-spelled, Pg 9 (= transfer)<br>Thanks for your notification, we have corrected "transnfer" as "transfer " (Line 202).<br><br>2.61: -"The gene clusters were identified among 12 selected genome" should be "Gene clusters were identified among 12 selected genomes"<br>Thanks for your notification, we have revised "The gene clusters were identified among 12 selected genome " into " Gene clusters were identified among 12 selected genomes " (Line 229).<br><br>2.62: -"reflected the closest relationship between M. coruscus and the clade of M. philippinarum and B. platifrons," This is oddly stated. I think you mean "M. coruscus was found to be the sister taxon to the clade containing M. philippinarum and B. platifrons". Also, how was the divergence time calculated?<br>Thanks, we corrected the sentence as "M. coruscus is a sister taxon to the clade containing M. philippinarum and B. platifrons" (Line 248-249).<br><br>Sorry for the confusion, we revised the sentence into "single-copy orthologs and one reference divergence time on the root node obtained from the TimeTree database were used to calibrate the divergence dates of other nodes on this phylogenetic tree using the MCMCTREE tool in the PAML package" (Line 243-246).<br><br>2.63: - s needed, " in farmed and wild sample, respectively" should be " in farmed and wild samples, respectively"<br>Thanks for your notification, we have revised "in farmed and wild sample, respectively" into " in farmed and wild samples, respectively " (Line 255).<br><br>2.64:-"while 5,719,771 and 1,820,404 in wild one" should read "and 5,719,771 and 1,820,404 in wild populations"<br>Thanks for your notification, we have revised "while 5,719,771 and 1,820,404 in wild one " into " and 5,719,771 and 1,820,404 in wild populations "<br><br>2.65:-"The chromosome synteny illustrated that rare large-scale rearrangements between scallop and mussel, but frequent between scallop and oysters" should be rewritten "Chromosome synteny illustrates that large-scale rearrangements are rare between scallop and mussel, but more frequent between scallop and oysters"<br>Thanks for your notification, we have corrected the sentence as "Chromosome synteny

illustrates that large-scale rearrangements are rare between scallop and mussel, but more frequent between scallop and oysters" (Line 292-294).

2.66:-No s "almost all of the chromosomes rearrangements " - should be "almost all of the chromosome rearrangements "
Thanks for your notification, we have revised " almost all of the chromosomes rearrangements " into " almost all of the chromosome rearrangements " (Line 308).

2.67:-"To profile the gene expressions" should be "To profile gene expression"
Thanks for your notification, we have revised " To profile the gene expressions " into " To profile gene expression "

2.68:-"Quality of the assembled genome" should read "The quality of the assembled genome.... "
Thanks for your notification, we have revised "Quality of the assembled genome " into " The quality of the assembled genome " (Line 349).

2.69:-"in genome assemble" should read "in the genome assembly"
Thanks for your notification, we have revised " in genome assemble " into " in the genome assembly " (Line 364-365).

2.70:-"facilitate a wide range of researches in mussel, bivalve, and molluscan." needs another word after molluscan - molluscan biology, maybe?
Sorry for the confusion, we have corrected as " mussels, bivalves, and mollusks " (Line 374).

2.71:-"evolution in bivalve" should be "evolution in bivalves"
Thanks for your notification, we have revised " evolution in bivalve " into " evolution in bivalves " (Line 375).

2.72:-"As one of the best-assembled bivalve genomes" - this is too strong a claim given the evidence presented.
Thanks for your suggestions, we have revised "As one of the best-assembled bivalve genomes" into " As one of the chromosome-level genome assemblies in Bivalve " (Line 376-377).

2.73:Please note there are numerous additional language problems to correct, and this is beyond the scope of my review. I suggest a careful re-reading of the manuscript before resubmission.
Sorry for the confusion, we have re-read and revised the manuscript thoroughly. The revised manuscript has been professionally edited by a native English-speaking colleague.

Reviewer #3: This study presented a high-quality genome of the mussel Mytilus coruscus. Using a mixed strategy to combine Illumina short reads and Nanopore long reads followed by scaffolding with Hi-C, the authors generated a chromosomal-level genome assembly. They further re-sequenced farmed and wild individuals to detect SNP and indel differences among the two populations. The authors then focused on the pathways related to larval settlement and metamorphosis using RNA-seq analysis. Overall, the genome quality looks good, but I have a few questions on how the authors analyzed and interpreted genome and transcriptome data.

Major comments:

1. Although the authors assess the genome completeness with the BUSCO test, a single BUSCO percentage value is not informative when considering the concept of an orthologs finding strategy (i.e. a comparative approach, reference points are needed). To better show the genome completeness, the authors are encouraged to perform the BUSCO test on all close-related available mollusc genomes.
Thanks for your suggestion, we assayed the genome completeness using Benchmark Universal Single-Copy Orthologs BUSCO v4.1.4 referencing metazoan and molluscan gene sets. In the metazoan dataset, the current assemblies have 89.4% complete (of which 1.0% were duplicated), 1.9% incomplete and 8.7% missing BUSCOs, corresponding to a recovery of 91.3% of the entire BUSCO set. In the molluscan

dataset, 85.5% complete (of which 1.3% were duplicated), 0.8% incomplete and 13.7% missing BUSCOs were recorded, corresponding to 86.3% of the entire BUSCO set (Line 352-361).
In addition, we performed the BUSCO tests using these close-related available bivalve genomes (see the following table) to show the recovery (Complete + imcomplete) of the entire BUSCO set,
SpeciesMetazoaMollusca
Pinctada fucata martensii90.1%84.0%
Pecten maximus96.5%95.9%
Mytilus coruscus91.3%86.3%
Mytilus coruscus previous version94.5%88.7%
Modiolus philippinarum90.1%84.0%
Bathymodiolus platifrons93.7%90.1%
Venustaconcha ellipsiformis74.5%54.9%

2. Figure 4a: Using Circos to show genome-wide SNPs and indels between farmed and wild populations doesn't seem informative. I don't know what the readers should expect to see from this panel. If there is no information, then consider removing it from the main figure. Instead, the authors should show a few specific examples, such as the SNP differences at the locus of chitobiase mentioned in the main text. Only listing KEGG or GO terms such as "genetic information processing", "metabolism", and "signaling and cellular processes" is too general and provides no useful information to the readers.
Thanks for your suggestions, we have put the Circos in supplementary Figures and provided the specific example of SNP differences at the locus of chitobiase in the main text and Figure 4b. The speculation of functions have been removed in the revision, because the evidence is absent. We re-write this section and weaken the claims made from it in the abstract and conclusion since this is just a preliminary try in the genome study.

3. Since the genome of the mussel Mytilus coruscus has been previously published, the main point of this paper seems to be their chromosome-level assembly. However, the advantage of having a chromosome-level genome in this manuscript is not apparently demonstrated. And the analysis of Figure 5 is not clear, especially for Figure 5e. The authors are encouraged to pay more attention to this part and present better data to demonstrate the benefit of having a chromosome-level assembly.
Thanks for your suggestions, we re-edit the Figure 5 by adding the subtitiles for the chromosome synteny of P. maximus vs S. broughtonii, P. maximus vs M. coruscus, P. maximus vs P. fucata, and P. maximus vs C. gigas and the dashed lines to indicate the corresponding evolution relationship (Fig. 5e).

4. Figure 6: I understand that the authors tried to use KEGG annotation to make sense of their RNA-seq data, but do mussels have cardiomyocytes? If not, how can a cardiomyocyte pathway be directly applied to a set of mussel genes? For example, actin and myosin are ubiquitous genes as cytoskeleton or component of muscle fibers. What is the rationale to link authors' assumption by just looking at these general gene expressions? Similar to this line, other signaling genes, such as NF-κB and many other protein kinases, also play roles in many different pathways. I do not think that the authors can conclude anything from randomly selecting a set of genes in the cell type that are not existing in the species they analyzed.
Most of the KEGG pathways are constructed by the model animals or plants, not by the mussels. So we focus the pathways that have been reported to be related to metamorphosis in mussel. We analyzed the up-regulated genes during the period from umbo to pediveliger, of which 26 genes are involved in “adrenergic signaling in cardiomyocytes”, “calcium signaling pathway”, “MAPK signaling pathway”, “protein export”, “endocytosis” and “catecholamine biosynthesis” pathways. These pathways are reported to be involved in settlement and metamorphosis [18, 66]. Most of the involved genes are functionally identified to be associated with metamorphosis development (Supplementary Table S5). Selection of these genes are based on their function information, not from a random selection. Most of our observations are consistent with exist study of metamorphosis development. Noticeably, mussels have cardiomyocyte, like most of mollusca species (watts et al, 1981; Kodirov 2011). The recent proteome analysis (Di et al. 2020) and ISH (Yang et al. 2012) identify that the "adrenergic signaling in cardiomyocytes" pathway is functional during metamorphosis

	of oyster, reflecting its importance in regulation of metamorphosis.<br>This transcriptome analyses of larva tissues provide a preliminary try to take advantage of current reference genome to investigate the metamorphosis development. Hence, we weaken the speculating claims in the revision, such as discarding the previous hypothesis that signal transduction controlling the metamorphosis development seemed to activate during the first two stages. The instructive suggestion is raised in the end of the section, instead.<br>Reference:<br>Watts, J.A., Koch, R.A., Greenberg, M.J. and Pierce, S.K. (1981), Ultrastructure of the heart of the marine mussel, Geukensia demissa. J. Morphol., 170: 301-319.<br>Kodirov, S. A. (2011). The neuronal control of cardiac functions in Molluscs. Comparative Biochemistry and Physiology Part A: Molecular & Integrative Physiology, 160(2), 102-116.<br>Di, G., Xiao, X., Tong, M.H. et al. (2020), Proteome of larval metamorphosis induced by epinephrine in the Fujian oyster Crassostrea angulata. BMC Genomics 21, 675.<br>Yang, B., Qin, J., Shi, B., Han, G., Chen, J., Huang, H., and Ke, C. (2012). Molecular characterization and functional analysis of adrenergic like receptor during larval metamorphosis in Crassostrea angulata. Aquaculture 366-367, 54-61.<br><br>5: Furthermore, the heatmap is also not informative. Do these genes differentially expressed at a particular stage? What is the statistical method that the authors use to evaluate differentially expressed genes? With their RNA-seq analysis, the authors expose their weakness in the developmental process of mussels. The whole study is confusing and inconclusive.<br>A supplementary table corresponding to the heatmap (Fig.6) is added in the revision, which lists the detailed description of gene functions and the related references. Most of the DEGs in the heatmap are differentially expressed during at least one stage. Quantified gene expression levels are normalized to the TPM values in the revision. This Limma statistical methodologise are suitable to detect differentially expressed genes based on linear models (Smyth et al. 2005). To ensure that the claims are proportionate to the evidence presented，we moderate the conclusion by constructive suggestions instead of the strong claims in the revision<br>Reference:<br>Smyth GK, Ritchie M, Thorne N, et al. LIMMA: linear models for microarray data. In Bioinformatics and Computational Biology Solutions Using R and Bioconductor. Statistics for Biology and Health. 2005.
**Additional Information:**	
**Question**	**Response**
Are you submitting this manuscript to a special series or article collection?	No
**Experimental design and statistics**<br><br>Full details of the experimental design and statistical methods used should be given in the Methods section, as detailed in our Minimum Standards Reporting Checklist. Information essential to interpreting the data presented should be made available in the figure legends.<br><br>Have you included all the information requested in your manuscript?	Yes
**Resources**	Yes

Powered by Editorial Manager® and ProduXion Manager® from Aries Systems Corporation

A description of all resources used, including antibodies, cell lines, animals and software tools, with enough information to allow them to be uniquely identified, should be included in the Methods section. Authors are strongly encouraged to cite Research Resource Identifiers (RRIDs) for antibodies, model organisms and tools, where possible.<br><br>Have you included the information requested as detailed in our Minimum Standards Reporting Checklist?	
**Availability of data and materials**<br><br>All datasets and code on which the conclusions of the paper rely must be either included in your submission or deposited in publicly available repositories (where available and ethically appropriate), referencing such data using a unique identifier in the references and in the “Availability of Data and Materials” section of your manuscript.<br><br>Have you have met the above requirement as detailed in our Minimum Standards Reporting Checklist?	Yes

Powered by Editorial Manager® and ProduXion Manager® from Aries Systems Corporation

LaTeX - Other (clo, cls, sty etc.)

Click here to access/download;LaTeX - Other (clo, cls, sty etc.);Response to comments of the editor and

RESPONSE LETTER

Ying Lu, January 2021

Dear Dr. Hans Zauner

Giga Science

Manuscript GIGA-D-20-00287R1.

Dear Reviewers and Editor, thank you for your time and valuable help in improving this manuscript. Please find below our detailed response letter (answers in blue) addressing in the comments.

Reviewer Comments:

Reviewer #1:

The manuscript by Yang and colleagues reports a high quality genome assembly for the mussel *Mytilus coruscus*. Although this is not the first genome assembly published for this species, this resource is an improvement compared with the previous version, due to the use of Hi-C libraries and a better management of heterozygous genomic regions. Hence, the contents of this work appear to be appropriate for a data note article. there are however several points that would require some additional information to be added, and bits of text that need to be modified to improve the flow of the text.

Dear reviewer, thank you for your comments and suggestions to improve this manuscript. As requested, we included more information about PAV and the genomic coverage, improved the language by a native English speaker, and made other corrections as suggested.

General comments:

I would suggest the authors to specify the sequencing coverage achieved somewhere in the text (i.e. which coverage was obtained with ONT reads? Which coverage was obtained with Illumina PE? Etc.). This is present in Table 1, but it should be also mentioned in the text.

Thanks for your suggestion, we have specified the sequencing coverage in the text (Line 127, Line 134 and Line 136).

The authors emphasized the high heterozygosity of the genome, pointing out the possible links between SNPs and phenotypic variation. The authors may not be aware of the very recent discoveries that currently indicate that bivalve genomes are characterized by significant hemizygosity and structural variants that affect gene content, resulting in massive gene presence/absence variation. While the authors are not currently required to update this work with a detailed analysis of PAV, I think the text might benefit from some additional points of discussion, especially considering the fact that a congeneric mussel species, *M. galloprovincialis***,** has been shown to be characterized by an astounding level of intraspecific genomic variation (see the preprint by Gerdol et al. 2019, which has recently been accepted for publication and should become available online in the matter of a few weeks on genome Biology). Also see the preprint by Calcino and colleagues here: https://www.biorxiv.org/content/10.1101/2020.09.15.298695v1

Thanks for your suggestion. We have checked two related papers to understand the hemizygosity and PAV as described. The papers discovery that bivalve genomes are characterized by significant hemizygosity and structural variants that affect gene content. We cite that reference of the PAV in Line 225-226 and Line 277-279 as "which might be owing to the widespread hemizygosity and massive gene presence/absence variation (PAV) (Gerdol et al. 2020; Calcino et al. 2020)" and "In addition, PAV may play a role in determining phenotypic traits (Gerdol et al. 2020; Calcino et al. 2020), which should be included in the future re-sequencing analyses."

Reference:

Gerdol M, Moreira R, Cruz F, et al. Massive gene presence-absence variation shapes an open pan-genome in the Mediterranean mussel. Genome Biology 2020; 21:275.

Calcino AD, Kenny NJ, Gerdol M. Single individual structural variant detection uncovers widespread hemizygosity in molluscs. bioRxiv 2020; 298695.

In general, I would recommend the authors to involve a native English-speaking colleague in the revision of the text, as several grammar errors and oddly constructed sentences are present throughout the text.

Thanks for your suggestion. The revised manuscript has been professionally edited by a native English-speaking colleague. The main alternations are highlighted in the revision.

Abstract

1.4: -correct "high-through"; I guess the authors were referring to "high throughput" here.

We replaced “high-through” with “high throughput” (Line 28).

1.5:-Correct "platifron" with "paltifrons"

We revised “platifron” into “paltifrons” (Line 36).

1.6:-"speculating their sharing same origins in evolution" please correct this odd wording.

Sorry for the confusion, we have corrected the sentence as “suggesting that this is shared ancestrally” (Line 38).

List of detailed comments

1.7:-Mussels have been also used as sentinel organisms for biomonitoring, and this information could be added to the list

Thanks for your suggestion, we have added biomonitoring in Line 54.

1.8:-"As with a dozen of marine invertebrates". This is unclear; I guess the authors meant "As several other marine invertebrates"

We rewrote the sentence (Line 61), as follows:

“As many other marine invertebrates, marine mussels also possess a free-swimming larval phase.”

1.9:-When talking about the *M. galloprovincialis* genome assembly, the authors only refer to the paper by Murgarella and colleagues, whereas an improved version has been recently accepted for publication on Genome Biology (this should be probably available online within a few weeks). The text is available as a preprint, see Gerdol et al. https://www.biorxiv.org/content/10.1101/781377v1

Thanks for your notification, we added the reference of an improved genome of *M. galloprovincialis* by Gerdol et al (Line 80).

Methods

1.10:-This section in particular suffers from the presence of several issues with the quality of the language used, that should be improved.

Thanks for your suggestion, we have revised this section to improve the language by a native English speaker.

1.11:-When talking about the k-mer graph, please refer to the homozygous and heterozygous peaks (instead of "junior peak"

Thanks for your comment. The homozygous and heterozygous peaks are clarified in the revision (Line 152-154). Calculation of the k-mer occurrence is improved using GenomeScope.

1.12:-"very close to the total assemblies (1.57 Gb)". I think it would be worth mentioning that this is also not far from the c-value previously estimated by cytogenetic studies (see Ieyama, H., O. Kameoka, T. Tan, and J. Yamasaki (1994). Chromosomes and nuclear DNA contents of some species of Mytilidae. Venus 53: 327-331)

Thanks for your notification. We added the reference of the genome size estimated by cytogenetic studies (Ieyama et al. 1994) (Line 156).

Reference:

Ieyama H, Kameoka O, Tan T, et al. Chromosomes and nuclear DNA contents of some species in Mytilidae. Venus (Japanese Journal of Malacology) 1994; 53:327-331.

1.13:-"which is much greater than the real size of 1.57 Gb". This is also in line with what has been observed for *M. galloprovincialis* by Gerdol et al.

Yes. The same observation are added in Line 158-160, as follows:

“This kind of over-estimation for genome size usually occurred to the fragmented assemblies, like the recently published M. galloprovincialis genome, in which considerable heterozygous redundancies seem to be included in the assemblies.”

Reference:

Gerdol M, Moreira R, Cruz F, et al. Massive gene presence-absence variation shapes an open pan-genome in the Mediterranean mussel. Genome Biology 2020; 21:275.

1.14:-"The yielded consensus sequences were manually checked by aligning to the GenBank database". This is a clever strategy, but I think it should be explained a bit better here.

Thanks. We revised the sentence as "The yielded consensus sequences were manually checked by aligning to the genes from the GenBank database (nt and nr; released in October 2019) to avoid that sequences of the high-copy genes are masked in following process with RepeatMasker".

1.15:-"which was less than previously-published 42,684 gene models in the draft genome because it introduced over 20% heterozygous redundancies in the assemblies". I agree with this consideration, but in light worth the recent findings about widespread hemizygosity and massive gene presence/absence variation in *M. galloprovincialis*, the authors might want to update the text with a few additional considerations.

We agree with that widespread hemizygosity and massive gene PAV probably cause the redundancies since it has been identified in M. galloprovincialis, as well as other molluscs (Gerdol et al. 2020; Calcino et al. 2020), as follows (Line 222-226):

"Using a bidirectional BLASTp between the two assemblies, we observed that an considerable heterozygous redundancies (over 20%) were probably included into the previous draft assemblies (Supplementary Table 3), which might be owing to the widespread hemizygosity and massive gene presence/absence variation (PAV) (Gerdol et al. 2020; Calcino et al. 2020) or assembling errors."

Reference:

Gerdol M, Moreira R, Cruz F, et al. Massive gene presence-absence variation shapes an open pan-genome in the Mediterranean mussel. Genome Biology 2020; 21:275.

Calcino AD, Kenny NJ, Gerdol M. Single individual structural variant detection uncovers widespread hemizygosity in molluscs. bioRxiv 2020; 298695.

1.16:-"448 single-copy genes". How were such genes identified? Was BUSCO/OrthoDB used for this?

Sorry for your confusion, we used OrthoDB to find the single-copy gene (Line 237).

1.17:-"Whole genome re-sequencing of farmed and wild individuals". The data provided here are potentially interesting for a preliminary analysis, but the authors should keep in mind (and briefly discuss) the possibility that higher-order structural variants which include gene PAV might have a very important role on phenotypic traits.

Thanks for your notification, we have included the PAV in the discussion in whole genome re-sequencing, as follows:

"In addition, PAV may play a role in determining phenotypic traits (Gerdol et al. 2020; Calcino et al. 2020), which should be included in the future re-sequencing analyses."

Reference:

Gerdol M, Moreira R, Cruz F, et al. Massive gene presence-absence variation shapes an open pan-genome in the Mediterranean mussel. Genome Biology 2020; 21:275.

Calcino AD, Kenny NJ, Gerdol M. Single individual structural variant detection uncovers widespread hemizygosity in molluscs. bioRxiv 2020; 298695.

1.18:-"consistent with their closest phylogenetic relationship in the Bivalvia clade" please add a reference for this.

Thanks. We add the Reference (Liu et al. 2020) for this.

Liu F, Li Y, Yu H, Zhang L, Hu J, Bao Z, Wang S. MolluscDB: an integrated functional and evolutionary genomics database for the hyper-diverse animal phylum Mollusca. Nucleic Acids Res. 2020 Nov 21:gkaa1166. doi: 10.1093/nar/gkaa1166. Epub ahead of print. PMID: 33219684.

1.19:-It would have been more appropriate to use TPM instead of FPKM？？, as this metric allows a more reliable comparison among samples.

Thanks for your suggestion, we use TPM instead of FPKM in transcriptome analysis (Supplementary Table S4, Line 318-320).

1.20:-"indicative of a 91.9% genome completeness when 89.98% of core metazoan orthologs were completely identified in the assemblies." This is somewhat unclear. Does 91.9% indicate present BUSCOs and 89.98% "present and complete"? Adding specific information concerning fragmented BUSCOs and duplicated or missing BUSCOs would help here.

As your suggested, we revised the sentence as "We assayed the genome completeness using Benchmark Universal Single-Copy Orthologs BUSCO v4.1.4 referencing metazoan and molluscan gene sets. In the metazoan dataset, the current assemblies have 89.4% complete (of which 1.0% were duplicated), 1.9% incomplete and 8.7% missing BUSCOs, corresponding to a recovery of 91.3% of the entire BUSCO set. In the molluscan dataset, 85.5% complete (of which 1.3% were duplicated), 0.8% incomplete and 13.7% missing BUSCOs were recorded, corresponding to 86.3% of the entire BUSCO set."

Reviewer #2: Yang et al present the genome assembly of the hard-shelled mussel Mytilus coruscus, alongside gene predictions and analysis of genome content. The work in assembling the genome and predicting genes is technically sound. However, the presentation of this work is inprecise, not yet of publishable standard, and would benefit from careful editing for science and language before re-submission. There are also several scientific points that need to be addressed, to ensure that the claims made in the manuscript are proportionate to the evidence presented. I have noted these below.

Major points to address:

2.1: 1) The authors claim that their genome represents a chromosome-level assembly of the genome of this species. This claim is based on the combination of reasonably long contigs into scaffolds using Lachesis based on linkage. To be able to firmly claim that these represent a "chromosome level assembly" it is necessary to evaluate the degree to which these pseudomolecules are assembled. Table 2 should provide data on the extent of gaps (total Ns) in each chromosome, and in the text, the size distribution of gaps, and information about them, should be noted. Are these, for instance, estimated and set at 100/1000 Ns? or are these a true reflection of the gap size? Is there any evidence of telomeric sequence at each end?

Thanks so much for your suggestions, we list the length (Ns) in the extents of the gaps (Table 2) in the revision. All of the gaps are set at 100 Ns, not the true reflection of the gap size. Total length of the gaps is 201.5 kbps (filled with 201.5 kbp Ns; Table 2; Line 180-181). We detect the characteristic motifs of telomeric sequences in 23 termini of the 13 chromosomes, suggesting the completeness of the assemblies (Supplementary Table S6; Line 359-361).

2.2: 2) There is a stark difference in estimated genome size between the previously published genome for this species and this resource. It would be useful to map the previous (draft) assembly of Li et al to this assembly and determine what percentage of the huge missing fragment (21%) of that assembly is truly missing from this assembly, and why. Does this represent uncollapsed heterozygosity (which would map twice to the same loci, presumably), intraspecific hemizygosity variation, or contamination in the previous genome resource? Or is it perhaps a problem of missing data in the assembly presented here? Any of these answers would be useful for understanding the genome of this species.

This is the good point. When the previous assemblies are aligned to present ones, a total of 141.8 Mb of genome sequences duplicates in the previous version while only 49.7 Mb in this resource (mapping rate of the Illumina reads against our assemblies was over 96.7%), indicating more heterozygous redundancies in the previous drafts. As far as the intraspecific hemizygosity variation reported in the *M. galloprovincialis* genome and other molluscan genomes (Gerdol et al. 2020; Calcino et al. 2020) is concerned, we do not have the evidence to clarify whether intraspecific hemizygosity variation results in different sizes of the assemblies. However, this reference is cited in the revision to demonstrate that over-estimation of the genome size sometime occurred to the draft assemblies (Line 158-160). In addition, comparative analysis of gene models also suggests considerably heterozygous duplicates in the previously published drafts (see response to 2.4).

Reference:

Gerdol M, Moreira R, Cruz F, et al. Massive gene presence-absence variation shapes an open pan-genome in the Mediterranean mussel. Genome Biology 2020; 21:275.

Calcino AD, Kenny NJ, Gerdol M. Single individual structural variant detection uncovers widespread hemizygosity in molluscs. bioRxiv 2020; 298695.

2.3: 3) Genome size estimation is carried out by a mathematical derivation directly from the highest peak size. This, however, partially excludes from consideration the heterozygous portion of the genome. As the assembly has been polished with Racon and Pilon, heterozygosity could also be underestimated by mapping estimates. It is recommended that alternate genome size estimates are provided. Genomescope

(http://qb.cshl.edu/genomescope/genomescope2.0/) is a simple-to-use option that will provide more nuanced information regarding genome size and heterozygosity.

As you suggested, we re-estimated the genome size and heterozygous rate in the revision using Genomescope. The assessment of genome size by *K*-mer counting using GenomeScope suggested a complete genome size of approximately 1.51 Gb (**Fig. 3a**) (Line 149-151; Line 154-156). The present genome had a heterozygous rate of 1.39 %, calculated by GenomeScope (Line 167-168).

2.4: 4) how many of the gene models found in Li et al are present/absent from the final gene set presented here? Were these used in the EVidenceModeler merge step? It is noted that "37,478 final gene models were generated (Table 3), which was less than previously-published 42,684 gene models in the draft genome because it introduced over 20% heterozygous redundancies in the assemblies". Please provide more information on how this was determined, as these extra genes could also represent recent duplicates, which should not be removed from consideration. This could build upon the results of 2) above.

The previous draft genome reported that the protein-coding gene set consists of 42,684 models (Li et al. 2020). However, we find 58,540 genes uploaded in GenBank, which is consistent with the gene numbers in their gff file. We compare the constructed gene families between the previous version and our annotations, using their 58,540 genes and our 37,478 genes. The gene duplicates are identified in the gene clusters of the two assemblies (see the following Table), in which A for the previously published genomes; B for the genome assemblies in this study. Quantity of the A-specific gene clusters that only consist of the genes from the previously published genome is significant higher than the B-specific ones that only consist of the genes from the assemblies in this study. Alignments against the NR database and repeat sequence library exhibits that 12,123 A-specific gene clusters (20.71% of 58,540) are annotated as transposable elements. The genes clustered in the families with more A members is much more than those in the families with more B members. We also find some genes with the same loci, splicing and even intron sequences. All of the information reflected a significant over-estimation in both genome size and quantity of protein-coding genes (Line 222-226).

Supplementary Table S3. Bidirectional BLASTp between the previously published gene models of the hard-shelled mussel and the predicted gene models in this study.

Relationship type of gene members in each family	Quantity of gene families (gene numbers in brackets)	
	Published draft assemblies (A)	Assemblies in this study (B)
One to one	15,265 (15,265)	15,265 (15,265)
One (A) to many (B)	281 (281)	281 (780)
Many (A) to one (B)	3,531 (10,781)	3,531 (3,531)
Many to many	541 (2,904)	541 (1,556)
A = B	180 (413)	180 (413)
A > B	327 (2,369)	327 (889)
A < B	34 (122)	34 (254)
Unique (only A or B)	3,569 (12,154)	538 (1,688)

Reference: Li RH, Zhang WJ, Lu JK, et al. The whole-genome sequencing and hybrid assembly of *Mytilus coruscus*. Frontiers in Genetics 2020; **11**:1-6.

2.5: 5) The phylogeny as presented needs further consideration. Was concatenation of genes performed before alignment? (page 11) This could introduce errors at the start and end of each gene as they can artifactually be aligned to non-homologous sequences. This should be checked, and repeated correctly if necessary (with alignment performed gene-by-gene, then concatenating the alignments). -What maximum likelihood model was used? what other settings? how many bootstraps? Please note in text (page 11). -How was divergence time calculated?

Sorry for the confusion. Alignment of one-to-one single copy genes is prior to concatenation of the alignments. The corresponding sentences "448 single-copy genes identified by OrthoDB were aligned and concatenated. The amino acid sequences were first aligned using MUSCLE, which were further concatenated to create one supergene sequence for each species and formed a data matrix" (Line 236-239) are corrected in the revision.

Line240-246: The phylogenetic relationship was constructed using the Maximum-likehood model in RAxML version 8 with the optimal substitution model of PROTGAMMAJTT. Robustness of the maximum-likelihood tree was assessed using the bootstrap method (100 pseudo-replicates). Furthermore, the single-copy orthologs and one reference divergence time on the root node obtained from

TimeTree database (http://www.timetree.org) were used to calibrate the divergence dates of other nodes on this phylogenetic tree by MCMCTREE tool in PAML package.

2.6: 6) In the "Whole genome re-sequencing of farmed and wild individuals" section, the assumption that sequence variations are farmed- population-specific (FPS) or wild-population-specific (WPS) is flawed as it is based on a tiny sample (20 individuals) of the enormous diversity of this species. It is not convincing to claim that these variants are unique to either farmed populations or wild populations - they are just observed to be different here due to the limited sampling. The depth of sequencing is also very low per individual (around 2.5x) and SNPs/indels could be missed. This section, and the claims made from it in the abstract and conclusions, need to be substantially reworked to avoid drawing universal conclusions from what are only initial pilot results.
Thanks for your suggestions. We re-write this section and weaken the claims made from it in the abstract and conclusion since this is just a preliminary try in the genome study. A simple case is added in the revision to illustrate the diversities between farmed and wild populations. We only make a brief speculation that sequence variation might be associated with morphological diversity (Line 276-277).

2.7: 7) The differential expression analysis in larvae is not convincing. Many of the genes cherry-picked for discussion and shown in Fig 6 are expressed in all samples. As only single libraries were sequenced for each larval life stage, claims for differential expression are only very weakly supported. It is good practice to use a minimum of 3 separate samples per condition for DE analysis, and preferentially more. The authors should moderate the strength of the conclusions drawn in the "Transcriptome related to metamorphosis" section considerably, in light of the strength of some of the evidence presented.
Thanks for your suggestion. We have used 3 biological replicates' RNA-Seq data of five developmental stages (SRR13364385、SRR13364374、SRR13364373、SRR13364371、SRR13364370、SRR13364369、SRR13364368、SRR13364367、SRR13364383、SRR13364382、SRR13364381、SRR13364380、SRR13364378、SRR13364377、SRR13364376) to analyze the differential expression with normalized

gene expression levels TPM (Line 317-319). We moderated the conclusion by removing the strong claims as “Signal transduction controlling the metamorphosis development seemed to activate during the first two stages, trochophore and D-veliger, although the major morphologic changes represented in the transition from pediveliger to juvenile”.

Minor points:

2.8: -The authors are often too strong in their criticism of the earlier genomes for this species and Mytilus. For instance "a low quality draft genome of M. coruscus has been reported" (pg 4). That resource is not as well-contiged, but saying it is low quality is not justified. Perhaps "Draft versions of the genomes of M. coruscus and M. galloprovincialis have been reported". This kind of strong claim should be toned down throughout the manuscript.
We deleted “a low quality” and corrected the sentence as “a draft genome of *M. coruscus* and an improved genome of *M. galloprovincialis* have been reported” (Line 79-80).

2.9: - Many of the steps shown in Fig 2 (e.g. read cleaning) are not covered in sufficient detail in the manuscript. Please ensure that the steps required to recapitulate this work are provided.
Thanks. We added the details to describe the steps in Fig 2, as follows:
Line 143-146: The raw reads from Illumina sequencing platform were cleaned using FastQC45 and HTQC46 by the following steps: (a) filtered reads with adapter sequence; (b) filtered PE reads with one reads more than 10% N bases; (c) filtered PE reads with any end has more than 50% inferior quality (≤5) bases.
Line 189-192: The yielded consensus sequences were manually checked by alignment to genes from the GenBank database (nt and nr; released in October 2019) to avoid that sequences of the high-copy genes are masked in following treatment with RepeatMasker.
Line 236-246: Gene clusters were identified among 12 selected genomes … calibrate the divergence dates of other nodes on this phylogenetic tree using the MCMCTREE tool in the PAML package.

2.10: -What settings were used for OrthoMCL?

The settings used for OrthoMCL are a BLASTp value cutoff of 1e−5 and an inflation parameter of 1.5 (Line 236).

2.11: -What settings were used to detect PCR duplicates with Picard?

Thanks for your notification, the duplicate reads were removed with the MarkDuplicates tool of Picard (Line 257-258).

2.12: Fig 3d: caniculata seems to be mis-spelled

Thanks for your notification, we have corrected "*canaliculate*" into "*canaliculata*" in Fig 3d.

2.13: Fig 5: Why is P. fucata highlighted? Why not show *P. maximus* vs *Mytilus coruscus*? It is the most relevant for this paper. Fig 5a and Fig 5b might be the wrong images?

We illustrate the chromosome synteny of *P. maximus* vs *S. broughtonii*, *P. maximus* vs *M. coruscus*, *P. maximus* vs *P. fucata*, and *P. maximus* vs *C. gigas* (Figure 5). We did not highlight P. fucata. Given that the genome of *P. maximus* was reported to be a slow-evolving genome with many ancestral features, the *P. maximus* is selected as a reference to compare with other four chromosome-level bivalves.

Note on language and scientific accuracy:

2.14: Throughout the manuscript there are minor errors in written english, which regularly introduce scientifically inaccurate statements. I have noted some of these below but my list is not complete, and the authors may wish to have their manuscript read over more thoroughly before resubmission. I have not had the time to correct all the errors present in the manuscript.

Thanks for your suggestion. The revised manuscript has been professionally edited by a native English-speaking colleague. The main alternations are highlighted in the revision.

2.15: Throughout: Please refer to the species name or the common name, but not "marine mussel" when you mean Mytilus coruscus - most mussels are marine. Similarly, do not use this to refer to all mussels.

Thanks for your notification, we have referred to the common name in revision (Line 41).

2.16: Title: the authors should consider introducing a comma into their title, breaking it into precise units: e.g. "A chromosome-level genome assembly of the hard-shelled mussel Mytilus coruscus, a widely distributed species from temperate areas of East Asia"

As your suggested, we corrected the title as "Chromosome-level genome assembly of the hard-shelled mussel Mytilus coruscus, a widely distributed species from the temperate areas of East Asia"

Abstract:

2.17: -no "A" in : A chromosome-level genome information

Thanks for your notification, we have deleted "A" in : A chromosome-level genome information (Line 24).

2.18: -high-through - do you mean high-throughput?

Thanks for your notification, we have corrected “high-through” as “high-throughput” (Line 28).

2.19: -" The completeness test exhibits" - I think you mean "comparison to the CEGMA metazoan complement reveals"

Thanks for your notification, we have corrected as "Comparison to the Core Eukaryotic Genes Mapping Approach (CEGMA) metazoan complement revealed" (Line 28).

2.20:-No "The" in "The phylogenetic analysis"

Thanks for your notification, we have revised "The phylogenetic analysis " into " Phylogenetic analysis " (Line 35).

2.21:-"the closest relationship between" - this is not true. I think you mean "phylogenetic analysis shows M. coruscus is the sister taxon to the clade comprised of Modiolus philippinarum and Bathymodiolus platifrons". Note spelling of last species

Thanks for your notification, we have revised the describtion of " the closest relationship between " into " Phylogenetic analysis showed that *M. coruscus* is a sister taxon to the clade including *Modiolus philippinarum* and *Bathymodiolus platifrons*. ", and we have corrected "*Bathymodiolus paltifrons* " into "*Bathymodiolus platifrons* " (Line 35-36).

2.22:-No "A", in "A conserved chromosome synteny "

Thanks for your notification, we have deleted "A" in "A conserved chromosome synteny" (Line 36).

2.23:-"speculating their sharing same origins in evolution" do you mean "suggesting that this is shared ancestrally"? Because the former is contentious

Thanks for your notification, we have corrected the sentence as "suggesting that this is shared ancestrally" (Line 38).

2.24:-no on in "studying on"

Thanks for your notification, we have deleted "on" in "studying on" (Line 42).

Context:

2.25:-phylum Mollusca (not Mollusc).

Thanks for your notification, we have corrected "Mollusc" as "Mollusca" (Line 47).

2.26:-"sea mussels". This is an inprecise phrase. Perhaps just use "mussels"

Thanks for your notification, we have revised "sea mussels " into "mussels" (Line 49).

2.27:- " Although their significance" - should read "Although they are significant for biology, ecology and the economy"

Thanks for your notification, we have revised " Although their significance in biology, ecology and economy " into " Although they are significant for biology, ecology and the economy " (Line 56-57).

2.28:- need an "and" before ", settlement mechanism."

Thanks for your notification, we have add an "and" before "settlement mechanism" (Line 60).

2.29:-"As with a dozen of marine invertebrates" - this is a deeply inaccurate statement. Perhaps "As with many marine invertebrates".

Thanks for your notification, we have revised " As with a dozen of marine invertebrates " into " As many marine invertebrates" (Line 61).

2.30:-"modeling of their anatomy " not "modeling of anatomy "

Thanks for your notification, we have revised " modeling of anatomy " into " remodeling of their anatomy " (Line 63).

2.31:-"trigger settlement and metamorphosis is universal in metazoan" - this is not true. Humans, for instance, are metazoans

Thanks for your notification, we have corrected "universal in" as " widespread among " (Line 68).

2.32:- "temperate areas" not "the temperate"

Thanks for your notification, we have revised " the temperate " into " temperate areas " (Line 71).

2.33:-"need adapt..." should read "needs to adapt to the hostile..."

Thanks for your notification, we have revised "need adapt to the hostile" into " needs to adapt to the hostile" (Line 74-75).

2.34:-"Up to date, chromosome level genome" should read "To date, a chromosomal-level genome"

Thanks for your notification, we have revised "Up to date, chromosome level genome" into " To date, no genome of any member of the genus *Mytilus* " (Line 78).

2.35:-"Lacking whole-genome information" should read "The lack of whole-genome information".

Thanks for your notification, we have revised " Lacking whole-genome information " into " The lack of whole-genome information " (Line 80-81).

2.36:-"The larvaes at five ..." should read "Larvae at five....".

Thanks for your notification, we have revised " The larvaes at five ... " into " Larvae at five.... " (Line 89).
2.36:-"gene expression" not "gene expressions"
Thanks for your notification, we have corrected "gene expressions" into "gene expression" (Line 90).

Methods:
2.38:-"where is the central coast of Chinese mainland" should read "the central coast of the Chinese mainland"
Thanks for your notification, we have revised "where is the central coast of Chinese mainland" into "which is the central coast of the Chinese mainland" (Line 97).

2.39:- "a" needed, A female wild adult with a mature ovary (although these are probably paired but difficult to detect - if paired this would be "with mature ovaries".)
Thanks for your notification, we have added " a " in " mature ovary " (Line 100), which was reported to be a mature ovary in mussel.

2.40:-" for the adductor muscle to isolate high molecular weight genomic DNA for sequencing of reference genome" should read ", with the adductor muscle taken for isolation of high molecular weight genomic DNA, for sequencing of the reference genome".
As your suggested, we have corrected the sentence as " and the adductor muscle was collected to isolate high-molecular-weight genomic DNA for the sequencing of the reference genome " (Line 101-102).

2.41:- no s "The DNAs"
Thanks for your notification, we have revised " The DNAs " into " The DNA" (Line 102).

2.42:-" to be assistant " should read "to assist with"
Thanks for your notification, we have revised " to be assistant " into " to assist with " (Line 101-106).

2.43:-" using SDS extraction method," should read " using the SDS extraction method," and a reference to this protocol should be given.

Thanks for your notification, we have added " the " in " using the SDS extraction method," and provided the reference (Eugene. 2000) (Line 109-110).

Sokolov EP. An improved method for DNA isolation from mucopolysaccharide-rich molluscan tissues, Journal of Molluscan Studies, 2000; 66 (4): 573–575, https://doi.org/10.1093/mollus/66.4.573.

2.44:-"total RNA were extracted" should read " total RNA was extracted "

Thanks for your notification, we have revised " were " into " was " (Line 114).

2.45:-"as well as the larvaes" should read "as well as larvae".

Thanks for your notification, we have corrected "larvaes" as " larvae".

2.46:"to get large segments " should read "to extract large fragments". fragments should be used instead of segments throughout this section.".

Thanks for your notification, we have revised " to get large segments " into " to extract large fragments " (Line 123). And we have corrected " segments " as " fragments ".

2.47:- The high quality library of average 20 kb in length was sequenced on the ONT PromethION platform with corresponding R9 cell and ONT sequencing reagents kit. The genomic DNA was sequenced using the MinION portable DNA sequencer with the 48 hours run script (Oxford Nanopore), which generated a total of 246.8 Gb data" were both the minion and promethion used? please make this clearer.

Sorry for your confusion, we only used PromethION platform and deleted the description of MinION portable DNA sequencer.

2.48:-" were fragmentized" should read " were fragmented"

Thanks for your notification, we have read "were fragmentized " into " was fragmented " (Line 129).

2.49:-novaseq needs a capital

Thanks for your notification, we have corrected " novaseq " as " NovaSeq " (Line 133).

2.50:- "by poly(A)" should read "for poly(A) transcripts". Which protocol was used?
Sorry for your confusion, we described the protocol as " The sample was enriched in mRNA by extracting poly(A) transcripts from total RNA using oligo-d(T) magnetic beads." (Line 138-139).

2.51:-" in 150 bp paired-end model." should read " in 150 bp paired-end mode."
Thanks for your notification, we have revised " in 150 bp paired-end model " into " in 150 bp paired-end mode " (Line 142).

2.52:-"Genome size of the hard-shelled " needs a "The" before
Thanks for your notification, we have revised " Genome size of the hard-shelled " into " The size of the hard-shelled mussel genome" (Line 149).

2.53: -" Average GC content of genome" needs a the before genome.
Thanks for your notification, we have revised " Average GC content of genome " into " an average GC content of genome " (Line 168).

2.54: -"The final assemblies is around 1.57 Gb" should be "The final assembly is around 1.57 Gb"
Sorry for the confusion, we have corrected the grammar (Line 165).

2.55: -"The genome assemblies of hard-shelled mussel" again should be assembly
Thanks for your notification, we have revised " The genome assemblies of hard-shelled mussel " into " The genome assembly of hard-shelled mussel " (Line 172).

2.56: -"with the softwares of Augustus (version 3.1) [38], GlimmerHMM (version 1.2) [39] and SNAP (version 2006-07-28) " should read "with Augustus (version 3.1) [38], GlimmerHMM (version 1.2) [39] and SNAP (version 2006-07-28) software"

Thanks for your notification, we have revised " with the softwares of Augustus (version 3.1) [38], GlimmerHMM (version 1.2) [39] and SNAP (version 2006-07-28) " into " using the Augustus (version 3.1) [38], GlimmerHMM (version 1.2) [39] and SNAP (version 2006-07-28) software " (Line 207-208).

2.57: -"protein sequences of two closed mollusc species" do you mean two closely related mollusc species?

Thanks for your suggestion, we have revised " two closed mollusc species " into "two closely related mollusc species" (Line 209).

2.58: -"Parallelly," should be "In parallel"

Thanks for your notification, we have revised " Parallelly," into "In parallel" (Line 211).

2.59: -"put into a de novo assemble" should be "assembled de novo"

Thanks for your notification, we have revised "put into a de novo assemble " into " assembled de novo " (Line 213).

2.60: -transnfer mis-spelled, Pg 9 (= transfer)

Thanks for your notification, we have corrected "transnfer" as "transfer " (Line 202).

2.61: -"The gene clusters were identified among 12 selected genome" should be "Gene clusters were identified among 12 selected genomes"

Thanks for your notification, we have revised "The gene clusters were identified among 12 selected genome " into " Gene clusters were identified among 12 selected genomes " (Line 229).

2.62: -"reflected the closest relationship between M. coruscus and the clade of M. philippinarum and B. platifrons," This is oddly stated. I think you mean "M. coruscus was found to be the sister taxon to the clade containing M. philippinarum and B. platifrons". Also, how was the divergence time calculated?

Thanks, we corrected the sentence as "*M. coruscus* is a sister taxon to the clade containing *M. philippinarum* and *B. platifrons*" (Line 248-249).

Sorry for the confusion, we revised the sentence into "single-copy orthologs and one reference divergence time on the root node obtained from the TimeTree database were used to calibrate the divergence dates of other nodes on this phylogenetic tree using the MCMCTREE tool in the PAML package" (Line 243-246).

2.63: - s needed, " in farmed and wild sample, respectively" should be " in farmed and wild samples, respectively"
Thanks for your notification, we have revised "in farmed and wild sample, respectively" into " in farmed and wild samples, respectively " (Line 255).

2.64:-"while 5,719,771 and 1,820,404 in wild one" should read "and 5,719,771 and 1,820,404 in wild populations"
Thanks for your notification, we have revised "while 5,719,771 and 1,820,404 in wild one " into " and 5,719,771 and 1,820,404 in wild populations "

2.65:-"The chromosome synteny illustrated that rare large-scale rearrangements between scallop and mussel, but frequent between scallop and oysters" should be rewritten "Chromosome synteny illustrates that large-scale rearrangements are rare between scallop and mussel, but more frequent between scallop and oysters"
Thanks for your notification, we have corrected the sentence as "Chromosome synteny illustrates that large-scale rearrangements are rare between scallop and mussel, but more frequent between scallop and oysters" (Line 292-294).

2.66:-No s "almost all of the chromosomes rearrangements " - should be "almost all of the chromosome rearrangements "
Thanks for your notification, we have revised " almost all of the chromosomes rearrangements " into " almost all of the chromosome rearrangements " (Line 308).

2.67:-"To profile the gene expressions" should be "To profile gene expression"
Thanks for your notification, we have revised " To profile the gene expressions " into " To profile gene expression "

2.68:-"Quality of the assembled genome" should read "The quality of the assembled genome.... "

Thanks for your notification, we have revised "Quality of the assembled genome " into " The quality of the assembled genome " (Line 349).

2.69:-"in genome assemble" should read "in the genome assembly"
Thanks for your notification, we have revised " in genome assemble " into " in the genome assembly " (Line 364-365).

2.70:-"facilitate a wide range of researches in mussel, bivalve, and molluscan." needs another word after molluscan - molluscan biology, maybe?
Sorry for the confusion, we have corrected as " mussels, bivalves, and mollusks " (Line 374).

2.71:-"evolution in bivalve" should be "evolution in bivalves"
Thanks for your notification, we have revised " evolution in bivalve " into " evolution in bivalves " (Line 375).

2.72:-"As one of the best-assembled bivalve genomes" - this is too strong a claim given the evidence presented.
Thanks for your suggestions, we have revised "As one of the best-assembled bivalve genomes" into " As one of the chromosome-level genome assemblies in Bivalve " (Line 376-377).

2.73:Please note there are numerous additional language problems to correct, and this is beyond the scope of my review. I suggest a careful re-reading of the manuscript before resubmission.
Sorry for the confusion, we have re-read and revised the manuscript thoroughly. The revised manuscript has been professionally edited by a native English-speaking colleague.

Reviewer #3: This study presented a high-quality genome of the mussel Mytilus coruscus. Using a mixed strategy to combine Illumina short reads and Nanopore long reads followed by scaffolding with Hi-C, the authors generated a chromosomal-level genome assembly. They further re-sequenced farmed and wild individuals to detect SNP and indel differences among the two populations. The authors then focused on

the pathways related to larval settlement and metamorphosis using RNA-seq analysis. Overall, the genome quality looks good, but I have a few questions on how the authors analyzed and interpreted genome and transcriptome data.

Major comments:

1. Although the authors assess the genome completeness with the BUSCO test, a single BUSCO percentage value is not informative when considering the concept of an orthologs finding strategy (i.e. a comparative approach, reference points are needed). To better show the genome completeness, the authors are encouraged to perform the BUSCO test on all close-related available mollusc genomes.

Thanks for your suggestion, we assayed the genome completeness using Benchmark Universal Single-Copy Orthologs BUSCO v4.1.4 referencing metazoan and molluscan gene sets. In the metazoan dataset, the current assemblies have 89.4% complete (of which 1.0% were duplicated), 1.9% incomplete and 8.7% missing BUSCOs, corresponding to a recovery of 91.3% of the entire BUSCO set. In the molluscan dataset, 85.5% complete (of which 1.3% were duplicated), 0.8% incomplete and 13.7% missing BUSCOs were recorded, corresponding to 86.3% of the entire BUSCO set (Line 352-361).

In addition, we performed the BUSCO tests using these close-related available bivalve genomes (see the following table) to show the recovery (Complete + imcomplete) of the entire BUSCO set,

Species	Metazoa	Mollusca
Pinctada fucata martensii	90.1%	84.0%
Pecten maximus	96.5%	95.9%
Mytilus coruscus	91.3%	86.3%
Mytilus coruscus previous version	94.5%	88.7%
Modiolus philippinarum	90.1%	84.0%
Bathymodiolus platifrons	93.7%	90.1%
Venustaconcha ellipsiformis	74.5%	54.9%

2. Figure 4a: Using Circos to show genome-wide SNPs and indels between farmed and wild populations doesn't seem informative. I don't know what the readers should expect to see from this panel. If there is no information, then consider removing it from the main figure. Instead, the authors should show a few specific examples, such

as the SNP differences at the locus of chitobiase mentioned in the main text. Only listing KEGG or GO terms such as "genetic information processing", "metabolism", and "signaling and cellular processes" is too general and provides no useful information to the readers.
Thanks for your suggestions, we have put the Circos in supplementary Figures and provided the specific example of SNP differences at the locus of chitobiase in the main text and Figure 4b. The speculation of functions have been removed in the revision, because the evidence is absent. We re-write this section and weaken the claims made from it in the abstract and conclusion since this is just a preliminary try in the genome study.

3. Since the genome of the mussel Mytilus coruscus has been previously published, the main point of this paper seems to be their chromosome-level assembly. However, the advantage of having a chromosome-level genome in this manuscript is not apparently demonstrated. And the analysis of Figure 5 is not clear, especially for Figure 5e. The authors are encouraged to pay more attention to this part and present better data to demonstrate the benefit of having a chromosome-level assembly.
Thanks for your suggestions, we re-edit the Figure 5 by adding the subtitiles for the chromosome synteny of P. maximus vs S. broughtonii, P. maximus vs M. coruscus, P. maximus vs P. fucata, and P. maximus vs C. gigas and the dashed lines to indicate the corresponding evolution relationship (Fig. 5e).

4. Figure 6: I understand that the authors tried to use KEGG annotation to make sense of their RNA-seq data, but do mussels have cardiomyocytes? If not, how can a cardiomyocyte pathway be directly applied to a set of mussel genes? For example, actin and myosin are ubiquitous genes as cytoskeleton or component of muscle fibers. What is the rationale to link authors' assumption by just looking at these general gene expressions? Similar to this line, other signaling genes, such as NF-κB and many other protein kinases, also play roles in many different pathways. I do not think that the authors can conclude anything from randomly selecting a set of genes in the cell type that are not existing in the species they analyzed.
Most of the KEGG pathways are constructed by the model animals or plants, not by the mussels. So we focus the pathways that have been reported to be related to metamorphosis in mussel. We analyzed the up-regulated genes during the period from

umbo to pediveliger, of which 26 genes are involved in “adrenergic signaling in cardiomyocytes”, “calcium signaling pathway”, “MAPK signaling pathway”, “protein export”, “endocytosis” and “catecholamine biosynthesis” pathways. These pathways are reported to be involved in settlement and metamorphosis [18, 66]. Most of the involved genes are functionally identified to be associated with metamorphosis development (Supplementary Table S5). Selection of these genes are based on their function information, not from a random selection. Most of our observations are consistent with exist study of metamorphosis development. Noticeably, mussels have cardiomyocyte, like most of mollusca species (watts et al, 1981; Kodirov 2011). The recent proteome analysis (Di et al. 2020) and ISH (Yang et al. 2012) identify that the "adrenergic signaling in cardiomyocytes" pathway is functional during metamorphosis of oyster, reflecting its importance in regulation of metamorphosis. This transcriptome analyses of larva tissues provide a preliminary try to take advantage of current reference genome to investigate the metamorphosis development. Hence, we weaken the speculating claims in the revision, such as discarding the previous hypothesis that signal transduction controlling the metamorphosis development seemed to activate during the first two stages. The instructive suggestion is raised in the end of the section, instead.

Reference:

Watts, J.A., Koch, R.A., Greenberg, M.J. and Pierce, S.K. (1981), Ultrastructure of the heart of the marine mussel, Geukensia demissa. J. Morphol., 170: 301-319.

Kodirov, S. A. (2011). The neuronal control of cardiac functions in Molluscs. Comparative Biochemistry and Physiology Part A: Molecular & Integrative Physiology, 160(2), 102-116.

Di, G., Xiao, X., Tong, M.H. et al. (2020), Proteome of larval metamorphosis induced by epinephrine in the Fujian oyster Crassostrea angulata. BMC Genomics 21, 675.

Yang, B., Qin, J., Shi, B., Han, G., Chen, J., Huang, H., and Ke, C. (2012). Molecular characterization and functional analysis of adrenergic like receptor during larval metamorphosis in Crassostrea angulata. Aquaculture 366-367, 54-61.

5: Furthermore, the heatmap is also not informative. Do these genes differentially expressed at a particular stage? What is the statistical method that the authors use to evaluate differentially expressed genes? With their RNA-seq analysis, the authors

expose their weakness in the developmental process of mussels. The whole study is confusing and inconclusive.

A supplementary table corresponding to the heatmap (Fig.6) is added in the revision, which lists the detailed description of gene functions and the related references. Most of the DEGs in the heatmap are differentially expressed during at least one stage. Quantified gene expression levels are normalized to the TPM values in the revision. This Limma statistical methodologise are suitable to detect differentially expressed genes based on linear models (Smyth et al. 2005). To ensure that the claims are proportionate to the evidence presented，we moderate the conclusion by constructive suggestions instead of the strong claims in the revision

Reference:

Smyth GK, Ritchie M, Thorne N, et al. LIMMA: linear models for microarray data. In Bioinformatics and Computational Biology Solutions Using R and Bioconductor. Statistics for Biology and Health. 2005.

Manuscript

Click here to access/download;Manuscript;Manuscript-maintext_Jan23Revision_highlighted.docx

Chromosome-level genome assembly of the hard-shelled mussel *Mytilus coruscus*, a widely distributed species from the temperate areas of East Asia

Jin-Long Yang[1,2,3,†,*], Dan-Dan Feng[1,2, †], Jie Liu[1,2, †], Jia-Kang Xu[1,2], Ke Chen[1,2], Yi-Feng Li[1,2], You-Ting Zhu[1,2], Xiao Liang[1,2], Ying Lu[1,2,*]

[1] International Research Center for Marine Biosciences, Ministry of Science and Technology, Shanghai Ocean University, Shanghai, China

[2] Key Laboratory of Exploration and Utilization of Aquatic Genetic Resources, Ministry of Education, Shanghai Ocean University, Shanghai, China

[3] Southern Marine Science and Engineering Guangdong Laboratory, Guangzhou, China

[†] These authors contributed equally: Jin-Long Yang, Dan-Dan Feng, Jie Liu.

* Corresponding author. E-mail: jlyang@shou.edu.cn, yinglu@shou.edu.cn

Tel: + 86-21-61900403; Fax: + 86-21-61900405

Abstract

Background: The hard-shelled mussel (*Mytilus coruscus*) is widely distributed in the temperate seas of East Asia, and is an important commercial bivalve in China. Chromosome-level genome information of this species will not only contribute to the development of hard-shelled mussel genetic breeding, but also to studies on larval ecology, climate change biology, marine biology, aquaculture, biofouling, and antifouling. **Findings:** We applied a combination of Illumina sequencing, Oxford Nanopore Technologies sequencing, and high-throughput chromosome conformation capture technologies to construct a chromosome-level genome of the hard-shelled mussel, with a total length of 1.57 Gb and a median contig length of 1.49 Mb. Approximately 90.9% of the assemblies were anchored to 14 linage groups. Comparison to the Core Eukaryotic Genes Mapping Approach (CEGMA) metazoan complement revealed that the genome carried 91.9% of core metazoan orthologs. Gene modeling enabled the annotation of 37,478 protein-coding genes and 26,917 non-coding RNA loci. Phylogenetic analysis showed that *M. coruscus* is a sister taxon to the clade including *Modiolus philippinarum* and *Bathymodiolus platifrons*. Conserved chromosome synteny was observed between hard-shelled mussel and king scallop, suggesting that this is shared ancestrally. Transcriptomic profiling indicated that the pathways of catecholamine biosynthesis and adrenergic signaling in cardiomyocytes might be involved in metamorphosis. **Conclusions:** The chromosome-level assembly of the hard-shelled mussel genome will provide novel insights into mussel genome evolution and serve as a fundamental platform for studies regarding the planktonic-

sessile transition, genetic diversity, and genomic breeding of this bivalve.

Keywords: *Mytilus coruscus*, genome sequencing, Hi-C, chromosome, metamorphosis

Context

Marine mussels, which belong to the phylum Mollusca, settle on most immersed surfaces of substrata and play a crucial role in marine ecosystems. As healthy and sustainable food items, these mussels are beneficial for humans due to the high economic value for fishery and aquaculture, constituting more than 8% of mollusc aquaculture production [1]. Simultaneously, mussels are also known as typical macrofouling organisms that result in detrimental economic and ecological consequences for the maritime and aquaculture industries [2-4]. Mussels have been used as model organisms for adaptation to climate change, biomonitoring, integrative ecomechanics, biomaterials, larval ecology, settlement and metamorphosis, adhesion, bacteria-host interaction, biofouling and antifouling studies [5-12]. Although they are significant for biology, ecology and the economy, whole genome information of marine mussels is limited [13, 14] and lack of these related knowledge postpones our understanding molecular basis on the adaption, evolution, breeding, genetic manipulation, bacteria-host interaction, and settlement mechanism.

As many other marine invertebrates, marine mussels also possess a free-swimming larval phase. After this stage, these minute larvae will settle on the substrata and finish metamorphosis transition, accompanied with dramatic remodeling of their anatomy [4, 15]. Multiple physicochemical stimuli play critical roles in the process of larval

settlement and metamorphosis [15-17]. Thus, understanding of larvae-juvenile transition process is still a keystone question in marine biology, larval ecology, aquaculture, biofouling and antifouling [4, 15, 18, 19]. The finding that chemical cues from bacterial biofilms trigger settlement and metamorphosis is widespread among metazoan [15, 16, 18].

The hard-shelled mussel (*Mytilus coruscus* Gould 1861, NCBI Taxonomy ID: 42192**, Fig. 1**) mainly inhabits temperate areas along the coastal waters of China, Japan, Korea and Far East of Russia, covering from East China Sea to Sea of Japan [20]. In China, the hard-shelled mussel is an important commercial bivalve as well as a typical macrofouling organism. As a sessile marine bivalve, the hard-shelled mussel needs to adapt to the hostile and complex environments of intertidal regions. Most of studies focused on the planktonic-sessile transition mechanism of receptor and biofilm regulation, host-bacteria interaction, aquaculture and biofouling and antifouling studies in this species [3-5, 12, 21-23]. To date, no genome of any member of the genus *Mytilus* has been assembled at the chromosome level, although a draft genome of *M. coruscus* [24] and an improved genome of *M. galloprovincialis* [13, 25] have been reported. The lack of whole-genome information has hindered the development of the hard-shelled mussel genetic breeding, larval ecology, climate change biology, marine biology, aquaculture, biofouling and antifouling studies.

In this study, we report a chromosome-level assembly of the hard-shelled mussel genome obtained by combining Illumina sequencing, Oxford Nanopore Technologies (ONT) sequencing, and high-throughput chromosome conformation capture (Hi-C)

technologies. We validated the genome assemblies by chromosome synteny analysis, comparing them with the published chromosome-level genomes of the most studied mollusks. Larvae at five early developmental stages were subjected to RNA sequencing (RNA-seq) analysis for the profiling of gene expression during metamorphosis. Accessible chromosome-level genome datasets [26, 27] will facilitate comparative genomics studies on chromosome rearrangements across different species.

Methods

Sample information and collection

Wild individuals for genome sequencing were collected from the coast of Shengsi, Zhejiang province, which is the central coast of the Chinese mainland, and one of the original and main breeding areas of the hard-shelled mussel in China. Farmed and wild adults were also collected from the coast of Shengsi (122.77E 30.73N and 122.74E 30.71N, respectively) (**Fig. 1**). A female wild adult with a mature ovary was dissected, and the adductor muscle was collected to isolate high-molecular-weight genomic DNA for the sequencing of the reference genome. The DNA extracted from the farmed and wild populations (10 individuals per population) was pooled for genome re-sequencing. Adductor muscle, mantle, gill, digestive gland, hemocyte, labial palp, female gonad, male gonad, foot, and gut tissues were dissected from fresh samples for transcriptome sequencing to assist with the prediction of protein-coding genes.

Isolation of genomic DNA and RNA

Genomic DNA was extracted from fresh adductor muscle tissue using the SDS extraction method [28], and then used for sequencing on an ONT PromethION platform (Oxford Nanopore Technologies, UK). Using the TIANamp Marine Animals DNA kit (Tiangen, China), DNA for whole genome re-sequencing was extracted from the muscles of five female and five male individuals from each population. Using the RNAiso Plus kit (TaKaRa, Japan), total RNA was extracted from 10 different tissues of five female and five male individuals from each population to obtain a large gene expression dataset. Fresh muscle cells were crosslinked with formaldehyde, and digestion, marking of DNA ends, and blunt-end ligation were performed as described in a previous study [29]. The purified DNA was used for Hi-C.

Genome sequencing with different technologies

A combined sequencing strategy was applied to obtain the hard-shelled mussel genome (**Fig. 2**). Qualified DNA was filtered using a BluePippin^{TM} System to extract large fragments. The large-fragment DNA was employed to construct a library using the ONT Template prep kit and the NEB Next FFPE DNA Repair Mix kit [New England Biolabs (NEB), USA]. A high-quality library with an average length of 20 kb was sequenced on the ONT PromethION platform with the corresponding R9 cell and ONT sequencing reagent kit. A total of 246.8 Gb of data (~159× coverage) were generated (**Table 1**).

Sequencing of Hi-C and genome survey libraries was performed on an Illumina sequencing platform. Briefly, the extracted DNA was fragmented to a size of 300–350 bp using an E210 Focused Ultrasonicator (Covaris, USA). The construction of paired-

end libraries encompassed the successive steps of end repair, poly(A) addition, barcode indexing, purification, and PCR amplification. The libraries were sequenced with the Illumina NovaSeq 6000 platform (Illumina, USA) to generate 150-bp paired-end reads. Sequencing of the Hi-C libraries generated a total of 249.6 Gb of data (~161× coverage), and sequencing of the genome survey libraries generated a total of 160.6 Gb of data (~104× coverage).

The qualified RNA extracted from the same tissues of 10 individuals was equally mixed for RNA-seq. The sample was enriched in mRNA by extracting poly(A) transcripts from total RNA using oligo-d(T) magnetic beads. Sequencing libraries were prepared using the NEBNext® Ultra™ RNA Library Prep Kit for Illumina® (NEB, USA) following the manufacturer's recommendations. A total of 10 libraries were sequenced on the Illumina NovaSeq 6000 platform in a 150-bp paired-end mode.

The raw reads from Illumina sequencing platform were cleaned using FastQC45 and HTQC46 by the following steps: (a) filtered reads with adapter sequence; (b) filtered PE reads with one reads more than 10% N bases; (c) filtered PE reads with any end has more than 50% inferior quality (≤5) bases.

Genome survey and contig assembly

The size of the hard-shelled mussel genome was estimated using the *K*-mer-based method implemented in Jellyfish (version 2.3.0) with values of 51-mers [30] and GenomeScope (10,000× cut-off) [31]. *K*-mers refer to all the *k*-mer frequency distributions from a read obtained through Illumina DNA sequencing. The homozygous

peak of the assembly was at a 57× coverage and the heterozygous peak was at a 28× coverage (**Fig. 3a**). The assessment of genome size by *K*-mer counting suggested a complete genome size of approximately 1.51 Gb (**Fig. 3a**), which is close to the final assembly(1.57 Gb) and cytogenetic estimates [32]. Sequence alignment between the previous assembly (1.90 Gb) [24] and the one in this study revealed considerable heterozygous redundancies in the former. This kind of overestimation of genome size usually occurs in fragmented assembly, like the recently published *M. galloprovincialis* genome [25].

Genome assembly from long-read data was carried out following three methods. First, long reads were *de novo* assembled using the Canu v1.5 software with default parameters [33]; next, error correction was performed with Racon v1.3.1 [34]. Then, further polishing with Illumina short-read data was conducted using Pilon v1.22 [35]. The final assembly was approximately 1.57 Gb in size, consisting of 6,449 contigs with an overall median length (N50) of 1.49 Mb, while the previously published draft genome only had an N50 of 0.66 Mb [24]. The present genome had a heterozygous rate of 1.39 % (also calculated by GenomeScope) and an average GC content of approximately 32%.

Anchoring of the contigs to pseudo-moleculars with Hi-C data

To complete the assembly of the hard-shelled mussel genome, Hi-C technology was carried out to generate information on the interactions among contigs. DNA from fresh adductor muscle tissue was used to prepare a Hi-C library. This was then sequenced on

the Illumina NovaSeq 6000 platform, producing 249.6 Gb of reads (**Table 1**). These reads were aligned to the assembled contigs using BWA aligner v0.7.10-r789 [36]. Lachesis v2e27abb was applied to anchor the contigs onto the linkage groups using the agglomerative hierarchical clustering method [37]. Finally, 2,029 contigs representing 90.9% of the total assemblies were successfully anchored to 14 chromosomes (**Table 2**); this number was consistent with the outputs of the karyotype [38]. The unclosed gaps only occupies 0.014% of the assembly (201,500 bp), which is filled with Ns. The N50 of the anchored contigs was over 1.7 Mb, around 1.14 times of the initial assemblies from the ONT long reads.

Genome annotation

A *de novo* repeat annotation of the hard-shelled mussel genome was carried out using RepeatModeler (version 1.0.11) [39] and RepeatMasker (version 4.0.7) [40]. RepeatModeler was used to construct the repeat library, which was then examined using two other programs, RECON and RepeatScout. The yielded consensus sequences were manually checked by aligning to the genes from the GenBank database (nt and nr; released in October 2019) to avoid that sequences of the high-copy genes are masked in following process with RepeatMasker. The final repeat library consisted of 2,264 consensus sequences with the respective classification information, which was used to run RepeatMasker against the genome assemblies. The repetitive sequences constituted a length of 735.6 Mb, representing 47.4% of the total genome length (**Supplementary Table S1**). Simple sequence repeats (SSRs) were identified using Tandem Repeats

Finder V 4.04. Only monomers, dimers, trimers, tetramers, pentamers, and hexamers with at least four repeat units were considered. The total length of the 5,324 identified SSRs was approximately 138.0 kb.

Conserved non-coding RNAs were predicted using the Rfam 11.0 databases. Putative microRNAs (miRNAs) and ribosomal RNAs (rRNAs) were predicted using Infernal (version 1.1.2) [41], and transfer RNAs (tRNAs) were predicted with tRNAscan-SE v2.0.3. A total of 9,186 miRNAs, 342 rRNAs, and 1,881 tRNAs were detected (**Supplementary Table S2**).

Protein-coding genes were predicted using a combined strategy of *ab initio* prediction, homology-based prediction, and transcriptome-based prediction (**Fig. 2**). The *ab initio* prediction was conducted using the Augustus (version 3.1) [38], GlimmerHMM (version 1.2) [39], and SNAP (version 2006-07-28) software [42]. For homology-based prediction, protein sequences of two closely related mollusk species (*Modiolus philippinarum* and *Bathymodiolus platifrons*), downloaded from GenBank, were aligned to the genome assemblies using Exonerate (version 2.2.0) [43]. In parallel, transcriptomic data from 10 tissues (GenBank SRA accession ID: PRJNA578350) were assembled *de novo* using Trinity (version 2.4.0) [44] and Cufflinks (version 2.2.1) [45]. The outputs of both assemblers were integrated using the Program to Assemble Spliced Alignments (PASA, version 2.3.3) [46]. After merging of all of these predictions using EVidenceModeler (v1.1.0) [46], a total of 37,478 final gene models were generated (**Table 3)**, a number lower than that of the previously published 42,684 gene models in the draft genome [24]. Functional annotations displayed that 35,471 protein-coding

genes (94.6% of the 37,478 gene models) have the alignment to one or more of the InterPro (version 5.22-61.0) [47], GO [48], KEGG [49], Swissprot [50] and NCBI non-redundant protein (NR) functional databases (**Table 4**; **Fig. 3b**). This information is illustrated in a genome landscape map (**Fig. 3c**). Using a bidirectional BLASTp between the two assemblies, we observed that an considerable heterozygous redundancies (over 20%) were probably included into the previous draft assemblies (Supplementary Table S3), which might be owing to the widespread hemizygosity and massive gene presence/absence variation (PAV) [25, 51] or assembling errors.

Phylogenetic analysis

Gene clusters were identified among 12 selected genomes, namely those of *Chlamys farreri* (PRJNA185465), *Pinctada fucata martensii* (GCA_002216045.1), *M. philippinarum* (GCA_002080025.1), *Crassostrea gigas* (GCF_000297895.1), *B. platifrons* (GCA_002080005.1), *Mizuhopecten yessoensis (GCA_002113885.2)*, *Penaeus vannamei* (ASM378908v1), *Pecten maximus* (GCA 902652985.1), *Scapharca* (*Anadara*) *broughtonii* (PRJNA521075), *Pomacea canaliculata* **(**PRJNA427478), *Haliotis discus hannai* (PRJNA317403), and *M. coruscus*, using OrthoMCL (version 1.4) with a BLASTp cut-off value of 10^{-5} and an inflation value of 1.5 [52]. A total of 448 single-copy genes identified by OrthoDB were aligned and concatenated. The amino acid sequences were first aligned using MUSCLE [53], and then further concatenated to create one supergene sequence for each species and form a data matrix. The phylogenetic relationships among different supergenes were then assessed using a

maximum-likelihood model in RAxML version 8 [54] with the optimal substitution model of PROTGAMMAJTT. The robustness of the maximum-likelihood tree was assessed using the bootstrap method (100 pseudo-replicates). Furthermore, single-copy orthologs and one reference divergence time on the root node obtained from the TimeTree database [55] were used to calibrate the divergence dates of other nodes on this phylogenetic tree using the MCMCTREE tool in the PAML package [56]. Visualization of phylogenetic relationships with FigTree (version 1.4.3) [57] suggested that *M. coruscus* is a sister taxon to the clade containing *M. philippinarum* and *B. platifrons*, with a divergence time of approximately 129 Mya (**Fig. 3d**).

Whole genome re-sequencing of farmed and wild individuals

Chromosome-level genome is important for re-sequencing and population genetic. We performed a preliminary try to detect sequence variation by sequencing two genomic DNA pools of wild population and farmed population. A total of 50.4 Gb and 46.7 Gb of Illumina clean reads were finally generated in farmed and wild samples, respectively. Over 89% reads were aligned to the reference genome with BWA (v0.7.10-r789) [36]. The PCR duplicates (duplicates introduced by PCR) were removed with MarkDuplicates in the Picard toolkit [58]. SNPs and small indels (10 bp or less) were identified with GATK (version 3.7) [59] with default parameters and the addition of three extra thresholds to discard unreliable items during post-filter analysis, namely: 1) any two SNPs located within 5 bp from each other; 2) any two indels located within 10 bp from each other; and 3) any SNPs located within 5 bp from an indel. Finally, we

identified 5,733,780 SNPs and 1,821,690 small indels in the farmed population and 5,719,771 SNPs and 1,820,404 small indels in the wild population. Similar distribution patterns of SNPs and indels were detected between the farmed and wild population (**Supplementary Fig. S1**) when nearly 99% of the SNPs/indels were shared by both populations (**Fig. 4a**), reflecting that only approximately 1% of the sequence variations were farmed population specific (FPS) or wild population specific (WPS). We focused on the differential variations located in the flanking regions and genic regions, between the farmed and wild populations, to identify candidate genes and causal mutations related to morphological traits. The software SnpEff version 2.0.5 [60] was applied to detect the effect of SNPs/indels by comparing the loci of SNPs/indels with those of protein-coding genes, which revealed that 59 genes carrying FPS SNPs/indels (FPSGs) and 57 genes carrying WPS SNPs/indels (WPSGs) underwent loss of translational start sites, gain or loss of stop codons, or variants in the acceptor/donor of splicing sites. Some variations were observed to cluster in farmed population (**Fig. 4b**), implicating a potentially influence to morphological diversity. In addition, PAV may play a role in determining phenotypic traits [25, 51], which should be included in the future re-sequencing analyses.

Chromosome synteny and evolution in bivalves

To investigate the evolution of the mussel chromosomes, gene collinearity was constructed by aligning the genes of the king scallop *P. maximus* to the reference genomes of the blood clam *S. broughtonii*, the hard-shelled mussel *M. coruscus*, the

pearl oyster *P. martensii*, and the Pacific oyster *C. gigas* using MCscan (version 0.8). The parameters of the MCscan alignment were set as -s, 7; k, 150; m, 250; e, $1e^{-10}$. We identified 404 scallop-vs-clam, 276 scallop-vs-mussel, 159 scallop-vs-pearl-oyster, and 232 scallop-vs-pacific-oyster syntenic blocks, which included 10,055, 4,716, 3,636, and 5,009 genes of blood clam, hard-shelled mussel, pearl oyster and Pacific oyster, respectively. The mean gene number per syntenic block was 21.4. King scallop and blood clam had the highest gene collinearity, consistent with their close phylogenetic relationship in the Bivalvia clade [61] (Fig. 3d). The chromosome synteny illustrated that large-scale rearrangements are rare between scallop and mussel, but frequent between scallop and oysters (Fig. 5b–d), as exemplified by considerable structural variations between the scallop and the Pacific oyster genomes (Fig. 5d). The identified cross-chromosome rearrangements between the scallop and mussel genomes were different from those between the genomes of scallop and the two oyster species (Fig. 5b–e). The scallop linkage groups (PM) 1, 5, 6, 8, 10, 16, 17, 18, and 19 were syntenic to a single mussel chromosome (MC) 8, 9, 3, 4, 10, 13, 11, 12, and 14, respectively. PM 2 and 15 were aligned to the same reference, MC 8; similarly, PM 3 and 14 aligned to MC 5, PM 4 and 7 aligned to MC 1, PM 9 and 12 aligned to MC 7, and PM 11 and 13 aligned to MC 6. Comparatively, some additional chromosome rearrangements occurred between scallop and the two oyster species, especially the Pacific oyster. Both the Pacific oyster chromosome 9 and the pearl oyster chromosome 7 were predominantly syntenic to the scallop PM 15, suggesting that they might carry conserved genomic regions with the same origin (Fig. 5c–e). Among all the syntenic

chromosomes, we did not observe any chromosome to be entirely conserved in all of the bivalve genomes. Intriguingly, almost all of the chromosome rearrangements between the mussel and the oyster genomes were different (Fig. 5e), implicating independent chromosome fusion events. The identification of such diverse chromosome rearrangements suggested a complex evolutionary history of bivalve chromosomes.

Metamorphosis-related transcriptome analysis

To profile gene expression during development and metamorphosis in hard-shelled mussels, RNA-seq analysis was conducted at five developmental stages: trochophore, D-veliger, umbo, pediveliger, and juvenile (PRJNA689932). The quantification of gene expression enabled the detection of 33,743 transcripts with the TPM > 0 at all stages (**Supplementary Table S4**). The limma statistical method was used to detect DEGs based on linear models [62]. Using the trochophore as control, 5,795; 6,163; 9,308; and 7,486 upregulated genes [$\log_2$(fold-change) > 1 and adjusted $P < 0.05$] were identified in D-veliger, umbo, pediveliger, and juvenile larvae, respectively. Functional annotation indicated that these were mainly involved in “environmental information processing” (“signal transduction” and “signaling molecules and interaction”) and “cellular processes” (“transport and catabolism”), in agreement with the key role of signal transduction and the endocrine system in larval development [17].

Since the ability to effectuate metamorphosis develops during the pediveliger

stage [17], we investigate the 774 up-regulated genes during the transition from the umbo to the pediveliger stage. Functional annotation revealed that they were mainly employed in a network of six related pathways: “adrenergic signaling in cardiomyocytes,” “calcium signaling pathway,” “MAPK signaling pathway,” “protein export,” “endocytosis,” and “catecholamine biosynthesis” (Fig. 6a), which have been reported to be involved in settlement and metamorphosis [18, 63]. The expression of most of the genes involved in these pathways increased during one or more periods (Fig. 6b). Among them, 20 genes have been functionally identified to be associated with metamorphosis (**Supplementary Table S5**) and 26 up-regulated encompassing from the umbo to the pediveliger stages belonged to the categories “adrenergic signaling in cardiomyocytes,” “calcium signaling pathway,” and “catecholamine transport”, which was consistent with the findings of a recent proteome study on larval settlement and the metamorphosis of oysters [63-66]. Although some additional pathways, such as “phagosome” and “oxytocin signaling pathway”, are also detected, we did not analyze them in detail because still lacking evidence on their involvement in metamorphosis. In summary, the analysis of the involved pathways revealed that biosynthesis, transport, and transduction of catecholamines might be critical for the completion of metamorphosis.

Assembly assessment

The quality of the assembled genome was validated in terms of completeness, accuracy of the assemblies, and conservation of synteny. Alignment of Illumina reads against the

reference genome revealed insert sizes of paired-end sequencing libraries of approximately 300–350 bp and a mapping rate of over 96.7%. We assayed the genome completeness using Benchmark Universal Single-Copy Orthologs BUSCO v4.1.4 referencing metazoan and molluscan gene sets. In the metazoan dataset, the current assemblies have 89.4% complete (of which 1.0% were duplicated), 1.9% incomplete and 8.7% missing BUSCOs, corresponding to a recovery of 91.3% of the entire BUSCO set. In the molluscan dataset, 85.5% complete (of which 1.3% were duplicated), 0.8% incomplete and 13.7% missing BUSCOs were recorded, corresponding to 86.3% of the entire BUSCO set. Motifs with the characteristics of telomeric repeats were detected in 23 termini of the 13 chromosomes, suggesting the completeness of the assemblies (Supplementary Table S6). The accuracy of the genome assembly was evaluated by calling sequence variants through the alignment of Illumina sequencing data against the genome. Sequence alignment with the BCFtools (version 1.3) [67] revealed 368,991 homozygous SNP loci, reflecting an error rate of less than 0.02% in the genome assembly. In addition, the highly conserved synteny and the strict correspondence of chromosome fusion points and gene assignment identified between the hard-shelled mussel and king scallop genomes (Fig. 5b) were indicative of a qualified assembly of the hard-shelled mussel genome, since the king scallop genome is considered as the best-scaffolded genome available for bivalves [68].

Conclusion

The chromosome-level assembly of the hard-shelled mussel genome presented here is

a well-assembled and annotated resource that would facilitate a wide range of research in mussels, bivalves, and mollusks. The outputs of this study shed light on the chromosome evolution in bivalves, resulting in the regulation of the molecular pathways involved in larval metamorphosis. As one of the chromosome-level genome assemblies of bivalves, this genome data set will serve as a high-quality genome platform for comparative genomics at the chromosome level.

Availability of Supporting Data and Materials

All of the raw Illumina and ONT reads were deposited to NCBI Sequence Read Archive and the assembled genome was deposited to GenBank under the accession number PRJNA578350. The corresponding genome sequences and read alignments (VCF files) were stored in Figshare [69] and GigaDB [68].

Abbreviations

TPM: the Transcripts per Million; GATK: Genome Analysis Tool Kit; GO: Gene Ontolog; KEGG: Kyoto Encyclopedia of Genes and Genomes; AC1: adenylate cyclase 1; AC10: adenylate cyclase 10; Akt: RAC serine/threonine-protein kinase; CaM: calmodulin; CaMKII: calcium/calmodulin-dependent protein kinase (CaM kinase) II; CAV1: caveolin 1; CAV3: caveolin 3; CREB: cyclic AMP-responsive element-binding protein; DBH: dopamine beta-monooxygenase; DDC: aromatic-L-amino-acid decarboxylase; DHPR: voltage-dependent calcium channel gamma-1; Epac: Rap guanine nucleotide exchange factor; ERK: mitogen-activated protein kinase 1/3; Gi: guanine nucleotide-binding protein G(i) subunit alpha; Gq: guanine nucleotide-binding

protein G(q) subunit alpha; Gs: guanine nucleotide-binding protein G(s) subunit alpha; ICER: cAMP response element modulator; IKS: potassium voltage-gated channel KQT-like subfamily member 1; IMP2: mitochondrial inner membrane protease subunit 2; INaK: sodium/potassium-transporting ATPase subunit alpha; MAOA: monoamine oxidase A; MAOB: monoamine oxidase B; MSK1: ribosomal protein S6 kinase alpha-5; NCX : solute carrier family 8 (sodium/calcium exchanger); NF-κB: nuclear factor NF-kappa-B p105 subunit; NHE: solute carrier family 9 (sodium/hydrogen exchanger); p38MAPK: p38 MAP kinase; PI3K: phosphatidylinositol-4,5-bisphosphate 3-kinase catalytic subunit alpha/beta/delta; PKA: protein kinase A; PKCα: classical protein kinase C alpha type; PLC: phosphatidylinositol phospholipase C; PP1: serine/threonine-protein phosphatase PP1 catalytic subunit; TnI: Troponin I; TPM: tropomyosin; TYR: tyrosinase; α-ARA: alpha-1A adrenergic receptor-like; α-ARB: adrenergic receptor alpha-1B; β2AR: adrenergic receptor beta-2.

Competing Interests

The authors declare no competing interests.

Funding

This study was supported by the National Key Research and Development Program of China (2018YFD0900601), Key Program for International Science and Technology Cooperation Projects of Ministry of Science and Technology of China (No. 2018YFD0900101), the National Natural Science Foundation of China (No. 41876159,

No. 41606147, No. 31802321), Key Special Project for Introduced Talents Team of Southern Marine Science and Engineering Guangdong Laboratory (Guangzhou) (GML2019ZD0402), the Shanghai Sailing Program (18YF1410000), China Postdoctoral science foundation (2019M6614770) and Program for study on genetic resources, environment and strategy of mussel culture in coast of Gouqi Island offshore.

Authors Contributions

J.L.Y., Y.L. and X.L. designed and supervised the study. K.C., J.K.X, Y.T.Z, Y.F.L. collected the samples and extracted the genomic DNA and RNA. Y.L., J.L. and D.D.F. performed genome assembly and bioinformatics analysis. J.L.Y., D.D.F., X.L., J.L. and Y.L. wrote the original manuscript. All authors reviewed the manuscript.

Figure legends

Figure 1. Sequenced individuals and sampling sites. **a.** Pictures of the sequenced individuals collected in Shengsi. A wild *M. coruscus* adult was used for genome sequencing. Both wild and farmed populations were used for re-sequencing. **b.** The geographic locations of the sampling sites.

Figure 2. Workflow of genome sequencing and annotation. The rectangles indicate the steps of data treatment and the diamonds indicate output or input data.

Figure 3. Annotation and evolution. **a.** GenomeScope plot of the 51-mer k-mer content within the hard-shelled mussel genome. Estimates of genome size and read data were shown. **b.** Venn diagram indicating the number of genes that were annotated in one or more databases. **c.** Genomic landscape of *M. coruscus*. The chromosomes were labeled as LG01 to LG14. From the outer to the inner circle: 5, marker distribution across 14 chromosomes at a megabase scale; 4, gene density across the whole genome; 3, SNP density; 2 and 1, number of repetitive sequences and GC content across the genome. 1–5 are drawn in non-overlapping 0.1-Mb sliding windows. The length of chromosomes is defined by the scale (Mb) on the outer circles. **d.** Phylogenetic tree based on protein sequences from 12 metazoan genomes, namely those of *Chlamys farreri* (PRJNA185465), *Pinctada fucata martensii* (GCA_002216045.1), *Modiolus philippinarum* (GCA_002080025.1), *Crassostrea gigas* (GCF_000297895.1), *Mytilus coruscus*, *Bathymodiolus platifrons* (GCA_002080005.1), *Mizuhopecten yessoensis*

(GCA_002113885.2), *Penaeus vannamei* (ASM378908v1), *Pecten maximus* (GCA 902652985.1), *Scapharca* (*Anadara*) *broughtonii* (PRJNA521075), *Pomacea canaliculata* (PRJNA427478), and *Haliotis discus hannai* (PRJNA317403).

Figure 4. Sequence variations between farmed and wild populations**. a.** Venn diagrams showing the number and distribution of indels and SNPs between the farmed and wild populations. **b.** Differences in the number of SNPs on the exons of chitobiase. The rectangles indicate the 14 exons of the chitobiase gene and the lines between the 14 rectangles indicate introns; the pink matrix represents reads from the farmed population, and the blue matrix represents reads from the wild population. Bases denoted by capital letters are located on exons, whereas those denoted by small letters are located on introns.

Figure 5. Chromosome synteny. **a.** Alignment of king scallop and blood clam chromosomes. **b.** Alignment of king scallop and hard-shelled mussel chromosomes. **c**. Alignment of king scallop and pearl oyster chromosomes. **d**. Alignment of king scallop and Pacific oyster chromosomes. The king scallop linkage groups are labeled as PM 1 to 19, the blood clam chromosomes as SB 1 to 19, the hard-shelled mussel chromosomes as MC 1 to 14, the pearl oyster chromosomes as PF 1 to 14, and the Pacific oyster chromosomes as CG 1 to 10. Scale unit, Mb. **a**–**d.** The circularized blocks represent the chromosomes of the five bivalves. Aligned homologous genes are connected by ribbons, shown in different colors depending on their chromosome

location. **e.** Rearrangements between the chromosomes of king scallop and those of four other bivalve species. The king scallop chromosomes are represented by bars of different colors, and synteny and rearrangements in the chromosomes of the four other bivalves are indicated by different blocks, whose colors correspond to those of the reference king scallop chromosomes, the dashed lines indicate the corresponding evolution relationship.

Figure 6. Spatial and temporal expression of genes involved in development and metamorphosis. **a.** Expression pattern of genes implied in the pathways of catecholamine biosynthesis and adrenergic signaling in cardiomyocytes, according to KEGG-based annotation. Red rectangles indicate upregulated genes during development and metamorphosis, red rectangles with black edge indicate upregulated genes at Pediveliger stage and white rectangles denote genes that were identified during KEGG analysis but whose expression did not change. Red bubbles represent the most important pathways in which the upregulated genes are involved. **b.** Heatmap showing the expression levels of all genes involved in the pathways of catecholamine biosynthesis and adrenergic signaling in cardiomyocytes across five developmental stages.

Table captions

Table 1. Statistics of whole genome sequencing using Illumina and ONT

Table 2. Results of contig anchoring on pseudochromosomes using Hi-C data

Table 3. General statistics of the predicted protein-coding genes

Table 4. General statistics of gene functional annotation

Additional Files

Supplementary Table S1. Repetitive sequences in the hard-shelled mussel genome

Supplementary Table S2. Overview of the predicted non-coding RNAs

Supplementary Table S3. Bidirectional BLASTp between the previously published gene models of the hard-shelled mussel and the predicted gene models in this study.

Supplementary Table S4. Gene expression profiles during five developmental stages

Supplementary Table S5. Genes involved in the pathways of catecholamine biosynthesis and adrenergic signaling in the cardiomyocytes were reported to affect metamorphosis.

Supplementary Table S6. Information of the motifs with the characteristic of telomeric repeats

Supplementary Figure S1. Circles showing genome-wide SNPs and indels from the farmed and wild populations. From the outer to the inner circle: first circle, marker distribution across 14 pseudochromosomes at a megabase scale; green circle, SNP density across the whole genome; red circle, indel density.

References

1. FAO. The state of world fisheries and aquaculture. 2018.
2. Amini S, Kolle S, Petrone L, et al. Preventing mussel adhesion using lubricant-infused materials. Science 2017; **357**:668-673.
3. Yang JL, Li YF, Guo XP, et al. The effect of carbon nanotubes and titanium dioxide incorporated in PDMS on biofilm community composition and subsequent mussel plantigrade settlement. Biofouling 2016; **32**:763-777.
4. Yang JL, Shen PJ, Liang X, et al. Larval settlement and metamorphosis of the mussel *Mytilus coruscus* in response to monospecific bacterial biofilms. Biofouling 2013; **29**:247-259.
5. Liang X, Peng LH, Zhang S, et al. Polyurethane, epoxy resin and polydimethylsiloxane altered biofilm formation and mussel settlement. Chemosphere 2019; **218**:599-608.
6. Odonnell MJ, George MN, Carrington E. Mussel byssus attachment weakened by ocean acidification. Nature Climate Change 2013; **3**:587-590.
7. Ramesh K, Hu MY, Thomsen J, et al. Mussel larvae modify calcifying fluid carbonate chemistry to promote calcification. Nature Communications 2017; **8**:1709.
8. Thomsen J, Stapp L, Haynert K, et al. Naturally acidified habitat selects for ocean acidification–tolerant mussels. Science Advances 2017; **3**:e1602411.
9. Bitter MC, Kapsenberg L, Gattuso J, et al. Standing genetic variation fuels rapid adaptation to ocean acidification. Nature Communications 2019; **10**:1-10.
10. Briand J. Marine antifouling laboratory bioassays: an overview of their diversity. Biofouling 2009; **25**:297-311.
11. Petrone L, Kumar A, Sutanto CN, et al. Mussel adhesion is dictated by time-regulated secretion and molecular conformation of mussel adhesive proteins. Nature Communications 2015; **6**:8737-8737.
12. Zeng ZS, Guo XP, Cai XS, et al. Pyomelanin from *Pseudoalteromonas lipolytica* reduces biofouling. Microbial Biotechnology 2017; **10**:1718-1731.
13. Murgarella M, Puiu D, Novoa B, et al. A first insight into the genome of the filter-feeder mussel *Mytilus galloprovincialis*. PLoS One 2016; **11**:e0151561.
14. Sun J, Zhang Y, Xu T, et al. Adaptation to deep-sea chemosynthetic environments as revealed by mussel genomes. Nature Ecology and Evolution 2017; **1**:0121.
15. Hadfield MG, Paul VG. In *Marine chemical ecology* (ed. McClintock, J.B & Baker, J.B) Ch13. CRC Press, 2001.
16. Dobretsov S, Rittschof D. Love at first taste: induction of larval settlement by marine microbes. International Journal of Molecular Sciences 2020; **21**:731.
17. Hadfield MG. Biofilms and marine invertebrate larvae: what bacteria produce that larvae use to choose settlement sites. Annual Review of Marine Science 2011; **3**:453-470.
18. Shikuma NJ, Antoshechkin I, Medeiros JM, et al. Stepwise metamorphosis of the tubeworm *Hydroides elegans* is mediated by a bacterial inducer and MAPK signaling. Proceedings of the National Academy of Sciences of the United States of America 2016; **113**:10097-10102.
19. Shikuma NJ, Pilhofer M, Weiss GL, et al. Marine tubeworm metamorphosis induced by arrays of bacterial phage tail–like structures. Science 2014; **343**:529-533.

20. Kulikova VA, Lyashenko SA, Kolotukhina NK. Seasonal and interannual dynamics of larval abundance of *Mytilus coruscus* Gould, 1861 (Bivalvia: Mytilidae) in Amursky Bay (Peter the Great Bay, Sea of Japan). Russian Journal of Marine Biology 2011; **37**:342-347.
21. Li YF, Liu YZ, Chen YW, et al. Two toll-like receptors identified in the mantle of *Mytilus coruscus* are abundant in haemocytes. Fish & shellfish immunology 2019; **90**:134-140.
22. Liang X, Zhang XK, Peng LH, et al. The flagellar gene regulates biofilm formation and mussel larval settlement and metamorphosis. International Journal of Molecular Sciences 2020; **21**:710.
23. Yang JL, Li SH, Li YF, et al. Effects of neuroactive compounds, ions and organic solvents on larval metamorphosis of the mussel *Mytilus coruscus*. Aquaculture 2013; **396-399**:106-112.
24. Li RH, Zhang WJ, Lu JK, et al. The whole-genome sequencing and hybrid assembly of *Mytilus coruscus*. Frontiers in Genetics 2020; **11**:1-6.
25. Gerdol M, Moreira R, Cruz F, et al. Massive gene presence-absence variation shapes an open pan-genome in the Mediterranean mussel. Genome Biology 2020; **21**:275.
26. Li YL, Sun XQ, Hu XL, et al. Scallop genome reveals molecular adaptations to semi-sessile life and neurotoxins. Nature Communications 2017; **8**:1721-1721.
27. Wang S, Zhang J, Jiao W, et al. Scallop genome provides insights into evolution of bilaterian karyotype and development. Nature ecology & evolution 2017; **1**:0120.
28. Sokolov EP. An improved method for DNA isolation from mucopolysaccharide-rich molluscan tissues. Journal of Molluscan Studies 2000; **66**:573-575.
29. Van Berkum NL, Lieberman-Aiden E, Williams L, et al. Hi-C: A method to study the three-dimensional architecture of genomes. Journal of Visualized Experiments 2010; **39**:e1869.
30. Marçais G, Kingsford C. A fast, lock-free approach for efficient parallel counting of occurrences of k-mers. Bioinformatics 2011; **27**:764-770.
31. Vurture GW, Sedlazeck FJ, Nattestad M, et al. GenomeScope: fast reference-free genome profiling from short reads. Bioinformatics 2017; **33**:2202-2204.
32. Ieyama H, Kameoka O, Tan T, et al. Chromosomes and nuclear DNA contents of some species in Mytilidae. Venus (Japanese Journal of Malacology) 1994; **53**:327-331.
33. Koren S, Walenz BP, Berlin K, et al. Canu: scalable and accurate long-read assembly via adaptive k-mer weighting and repeat separation. Genome research 2017; **27**:722-736.
34. Vaser R, Sović I, Nagarajan N, et al. Fast and accurate de novo genome assembly from long uncorrected reads. Genome research 2017; **27**:737-746.
35. Walker BJ, Abeel T, Shea T, et al. Pilon: an integrated tool for comprehensive microbial variant detection and genome assembly improvement. PloS one 2014; **9**:e112963.
36. Li H, Durbin R. Fast and accurate short read alignment with Burrows–Wheeler transform. Bioinformatics 2009; **25**:1754-1760.
37. Burton JN, Adey A, Patwardhan RP, et al. Chromosome-scale scaffolding of de novo genome assemblies based on chromatin interactions. Nature Biotechnology 2013; **31**:1119-1125.
38. Zhuang BX. A preliminary study on the chromosome of marine bivalve, *Mytilus coruscus*. Zoological Research 1984; **S2**.
39. Smit A, Hubley R. RepeatModeler Open-1.0. 2008:http://www.repeatmasker.org/.
40. Smit A, Hubley R, Green P. RepeatMasker Open-4.0. 2015:http://www.repeatmasker.org/.

41. Nawrocki EP, Eddy SR. Infernal 1.1: 100-fold faster RNA homology searches. Bioinformatics 2013; **29**:2933-2935.
42. Korf I. Gene finding in novel genomes. BMC Bioinformatics 2004; **5**:59.
43. Slater GSC, Birney E. Automated generation of heuristics for biological sequence comparison. BMC bioinformatics 2005; **6**:31.
44. Grabherr MG, Haas BJ, Yassour M, et al. Trinity: reconstructing a full-length transcriptome without a genome from RNA-Seq data. Nature biotechnology 2011; **29**:644.
45. Trapnell C, Williams BA, Pertea G, et al. Transcript assembly and quantification by RNA-Seq reveals unannotated transcripts and isoform switching during cell differentiation. Nature biotechnology 2010; **28**:511.
46. Haas BJ, Salzberg SL, Zhu W, et al. Automated eukaryotic gene structure annotation using EVidenceModeler and the Program to Assemble Spliced Alignments. Genome biology 2008; **9**:R7.
47. Zdobnov EM, Apweiler R. InterProScan–an integration platform for the signature-recognition methods in InterPro. Bioinformatics 2001; **17**:847-848.
48. Ashburner M, Ball CA, Blake JA, et al. Gene ontology: tool for the unification of biology. Nature genetics 2000; **25**:25.
49. Kanehisa M, Goto S, Kawashima S, et al. The KEGG resource for deciphering the genome. Nucleic Acids Research 2004; **32**:277-280.
50. Boeckmann B, Bairoch A, Apweiler R, et al. The SWISS-PROT protein knowledgebase and its supplement TrEMBL in 2003. Nucleic acids research 2003; **31**:365-370.
51. Calcino AD, Kenny NJ, Gerdol M. Single individual structural variant detection uncovers widespread hemizygosity in molluscs. bioRxiv 2020:298695.
52. Li L, Stoeckert CJ, Roos DS. OrthoMCL: identification of ortholog groups for eukaryotic genomes. Genome research 2003; **13**:2178-2189.
53. Edgar RC. MUSCLE: multiple sequence alignment with high accuracy and high throughput. Nucleic acids research 2004; **32**:1792-1797.
54. Stamatakis A. RAxML-VI-HPC: maximum likelihood-based phylogenetic analyses with thousands of taxa and mixed models. Bioinformatics 2006; **22**:2688-2690.
55. Kumar S, Stecher G, Suleski M, et al. TimeTree: a resource for timelines, timetrees, and divergence times. Mol Biol Evol 2017; **34**:1812-1819.
56. Yang Z. PAML: a program package for phylogenetic analysis by maximum likelihood. Computer applications in the biosciences 1997; **13**:555-556.
57. Rambaut A. FigTree, a graphical viewer of phylogenetic trees. 2007:http://tree.bio.ed.ac.uk/software/figtree/.
58. PicardToolkit. Broad Institute, GitHub Repository 2019:http://broadinstitute.github.io/picard/.
59. Mckenna A, Hanna M, Banks E, et al. The Genome Analysis Toolkit: A MapReduce framework for analyzing next-generation DNA sequencing data. Genome Research 2010; **20**:1297-1303.
60. Cingolani P, Platts AE, Wang LL, et al. A program for annotating and predicting the effects of single nucleotide polymorphisms, SnpEff: SNPs in the genome of *Drosophila melanogaster* strain w1118. Fly 2012; **6**:80-92.
61. Liu FY, Li YL, Yu HW, et al. MolluscDB: an integrated functional and evolutionary

genomics database for the hyper-diverse animal phylum Mollusca. Nucleic Acids Res 2020;**49**:D1556.

62. Smyth GK, Ritchie M, Thorne N, et al. LIMMA: linear models for microarray data. In Bioinformatics and Computational Biology Solutions Using R and Bioconductor. Statistics for Biology and Health. 2005.
63. Di G, Xiao X, Tong MH, et al. Proteome of larval metamorphosis induced by epinephrine in the Fujian oyster *Crassostrea angulata*. BMC Genomics 2020; **21**:675.
64. Eisenhofer G, Tian H, Holmes C, et al. Tyrosinase: a developmentally specific major determinant of peripheral dopamine. The FASEB Journal 2003; **17**:1248-1255.
65. Bonar DB, Coon SL, Walch M, et al. Control of oyster settlement and metamorphosis by endogenous and exogenous chemical cues. Bulletin of Marine Science 1990; **46**:484-498.
66. Joyce A, Vogeler S. Molluscan bivalve settlement and metamorphosis: neuroendocrine inducers and morphogenetic responses. Aquaculture 2018; **487**:64-82.
67. Narasimhan VM, Danecek P, Scally A, et al. BCFtools/RoH: a hidden Markov model approach for detecting autozygosity from next-generation sequencing data. Bioinformatics 2016; **32**:1749-1751.
68. Kenny NJ, Mccarthy S, Dudchenko O, et al. The Gene-Rich Genome of the Scallop *Pecten maximus*. GigaScience 2020;**9**:giaa037.
69. Feng DD. The hard-shelled mussel *Mytilus coruscus* gene models, annotatins and related files of the whole genome. Figshare 2020:doi:10.6084/m6089.figshare.10259618.

Manuscript

Click here to access/download;Manuscript;Manuscript-maintext_Jan23Revision.docx

Chromosome-level genome assembly of the hard-shelled mussel *Mytilus coruscus*, a widely distributed species from the temperate areas of East Asia

Jin-Long Yang[1,2,3,†,*], Dan-Dan Feng[1,2, †], Jie Liu[1,2, †], Jia-Kang Xu[1,2], Ke Chen[1,2], Yi-Feng Li[1,2], You-Ting Zhu[1,2], Xiao Liang[1,2], Ying Lu[1,2,*]

[1] International Research Center for Marine Biosciences, Ministry of Science and Technology, Shanghai Ocean University, Shanghai, China

[2] Key Laboratory of Exploration and Utilization of Aquatic Genetic Resources, Ministry of Education, Shanghai Ocean University, Shanghai, China

[3] Southern Marine Science and Engineering Guangdong Laboratory, Guangzhou, China

[†] These authors contributed equally: Jin-Long Yang, Dan-Dan Feng, Jie Liu.

* Corresponding author. E-mail: jlyang@shou.edu.cn, yinglu@shou.edu.cn

Tel: + 86-21-61900403; Fax: + 86-21-61900405

Abstract

Background: The hard-shelled mussel (*Mytilus coruscus*) is widely distributed in the temperate seas of East Asia, and is an important commercial bivalve in China. Chromosome-level genome information of this species will not only contribute to the development of hard-shelled mussel genetic breeding, but also to studies on larval ecology, climate change biology, marine biology, aquaculture, biofouling, and antifouling. **Findings:** We applied a combination of Illumina sequencing, Oxford Nanopore Technologies sequencing, and high-throughput chromosome conformation capture technologies to construct a chromosome-level genome of the hard-shelled mussel, with a total length of 1.57 Gb and a median contig length of 1.49 Mb. Approximately 90.9% of the assemblies were anchored to 14 linage groups. Comparison to the Core Eukaryotic Genes Mapping Approach (CEGMA) metazoan complement revealed that the genome carried 91.9% of core metazoan orthologs. Gene modeling enabled the annotation of 37,478 protein-coding genes and 26,917 non-coding RNA loci. Phylogenetic analysis showed that *M. coruscus* is a sister taxon to the clade including *Modiolus philippinarum* and *Bathymodiolus platifrons*. Conserved chromosome synteny was observed between hard-shelled mussel and king scallop, suggesting that this is shared ancestrally. Transcriptomic profiling indicated that the pathways of catecholamine biosynthesis and adrenergic signaling in cardiomyocytes might be involved in metamorphosis. **Conclusions:** The chromosome-level assembly of the hard-shelled mussel genome will provide novel insights into mussel genome evolution and serve as a fundamental platform for studies regarding the planktonic-

sessile transition, genetic diversity, and genomic breeding of this bivalve.

Keywords: *Mytilus coruscus*, genome sequencing, Hi-C, chromosome, metamorphosis

Context

Marine mussels, which belong to the phylum Mollusca, settle on most immersed surfaces of substrata and play a crucial role in marine ecosystems. As healthy and sustainable food items, these mussels are beneficial for humans due to the high economic value for fishery and aquaculture, constituting more than 8% of mollusc aquaculture production [1]. Simultaneously, mussels are also known as typical macrofouling organisms that result in detrimental economic and ecological consequences for the maritime and aquaculture industries [2-4]. Mussels have been used as model organisms for adaptation to climate change, biomonitoring, integrative ecomechanics, biomaterials, larval ecology, settlement and metamorphosis, adhesion, bacteria-host interaction, biofouling and antifouling studies [5-12]. Although they are significant for biology, ecology and the economy, whole genome information of marine mussels is limited [13, 14] and lack of these related knowledge postpones our understanding molecular basis on the adaption, evolution, breeding, genetic manipulation, bacteria-host interaction, and settlement mechanism.

As many other marine invertebrates, marine mussels also possess a free-swimming larval phase. After this stage, these minute larvae will settle on the substrata and finish metamorphosis transition, accompanied with dramatic remodeling of their anatomy [4, 15]. Multiple physicochemical stimuli play critical roles in the process of larval

settlement and metamorphosis [15-17]. Thus, understanding of larvae-juvenile transition process is still a keystone question in marine biology, larval ecology, aquaculture, biofouling and antifouling [4, 15, 18, 19]. The finding that chemical cues from bacterial biofilms trigger settlement and metamorphosis is widespread among metazoan [15, 16, 18].

The hard-shelled mussel (*Mytilus coruscus* Gould 1861, NCBI Taxonomy ID: 42192**, Fig. 1**) mainly inhabits temperate areas along the coastal waters of China, Japan, Korea and Far East of Russia, covering from East China Sea to Sea of Japan [20]. In China, the hard-shelled mussel is an important commercial bivalve as well as a typical macrofouling organism. As a sessile marine bivalve, the hard-shelled mussel needs to adapt to the hostile and complex environments of intertidal regions. Most of studies focused on the planktonic-sessile transition mechanism of receptor and biofilm regulation, host-bacteria interaction, aquaculture and biofouling and antifouling studies in this species [3-5, 12, 21-23]. To date, no genome of any member of the genus *Mytilus* has been assembled at the chromosome level, although a draft genome of *M. coruscus* [24] and an improved genome of *M. galloprovincialis* [13, 25] have been reported. The lack of whole-genome information has hindered the development of the hard-shelled mussel genetic breeding, larval ecology, climate change biology, marine biology, aquaculture, biofouling and antifouling studies.

In this study, we report a chromosome-level assembly of the hard-shelled mussel genome obtained by combining Illumina sequencing, Oxford Nanopore Technologies (ONT) sequencing, and high-throughput chromosome conformation capture (Hi-C)

technologies. We validated the genome assemblies by chromosome synteny analysis, comparing them with the published chromosome-level genomes of the most studied mollusks. Larvae at five early developmental stages were subjected to RNA sequencing (RNA-seq) analysis for the profiling of gene expression during metamorphosis. Accessible chromosome-level genome datasets [26, 27] will facilitate comparative genomics studies on chromosome rearrangements across different species.

Methods

Sample information and collection

Wild individuals for genome sequencing were collected from the coast of Shengsi, Zhejiang province, which is the central coast of the Chinese mainland, and one of the original and main breeding areas of the hard-shelled mussel in China. Farmed and wild adults were also collected from the coast of Shengsi (122.77E 30.73N and 122.74E 30.71N, respectively) (**Fig. 1**). A female wild adult with a mature ovary was dissected, and the adductor muscle was collected to isolate high-molecular-weight genomic DNA for the sequencing of the reference genome. The DNA extracted from the farmed and wild populations (10 individuals per population) was pooled for genome re-sequencing. Adductor muscle, mantle, gill, digestive gland, hemocyte, labial palp, female gonad, male gonad, foot, and gut tissues were dissected from fresh samples for transcriptome sequencing to assist with the prediction of protein-coding genes.

Isolation of genomic DNA and RNA

Genomic DNA was extracted from fresh adductor muscle tissue using the SDS extraction method [28], and then used for sequencing on an ONT PromethION platform (Oxford Nanopore Technologies, UK). Using the TIANamp Marine Animals DNA kit (Tiangen, China), DNA for whole genome re-sequencing was extracted from the muscles of five female and five male individuals from each population. Using the RNAiso Plus kit (TaKaRa, Japan), total RNA was extracted from 10 different tissues of five female and five male individuals from each population to obtain a large gene expression dataset. Fresh muscle cells were crosslinked with formaldehyde, and digestion, marking of DNA ends, and blunt-end ligation were performed as described in a previous study [29]. The purified DNA was used for Hi-C.

Genome sequencing with different technologies

A combined sequencing strategy was applied to obtain the hard-shelled mussel genome (**Fig. 2**). Qualified DNA was filtered using a BluePippin^{TM} System to extract large fragments. The large-fragment DNA was employed to construct a library using the ONT Template prep kit and the NEB Next FFPE DNA Repair Mix kit [New England Biolabs (NEB), USA]. A high-quality library with an average length of 20 kb was sequenced on the ONT PromethION platform with the corresponding R9 cell and ONT sequencing reagent kit. A total of 246.8 Gb of data (~159× coverage) were generated (**Table 1**).

Sequencing of Hi-C and genome survey libraries was performed on an Illumina sequencing platform. Briefly, the extracted DNA was fragmented to a size of 300–350 bp using an E210 Focused Ultrasonicator (Covaris, USA). The construction of paired-

end libraries encompassed the successive steps of end repair, poly(A) addition, barcode indexing, purification, and PCR amplification. The libraries were sequenced with the Illumina NovaSeq 6000 platform (Illumina, USA) to generate 150-bp paired-end reads. Sequencing of the Hi-C libraries generated a total of 249.6 Gb of data (~161× coverage), and sequencing of the genome survey libraries generated a total of 160.6 Gb of data (~104× coverage).

The qualified RNA extracted from the same tissues of 10 individuals was equally mixed for RNA-seq. The sample was enriched in mRNA by extracting poly(A) transcripts from total RNA using oligo-d(T) magnetic beads. Sequencing libraries were prepared using the NEBNext® Ultra™ RNA Library Prep Kit for Illumina® (NEB, USA) following the manufacturer's recommendations. A total of 10 libraries were sequenced on the Illumina NovaSeq 6000 platform in a 150-bp paired-end mode.

The raw reads from Illumina sequencing platform were cleaned using FastQC45 and HTQC46 by the following steps: (a) filtered reads with adapter sequence; (b) filtered PE reads with one reads more than 10% N bases; (c) filtered PE reads with any end has more than 50% inferior quality (≤5) bases.

Genome survey and contig assembly

The size of the hard-shelled mussel genome was estimated using the *K*-mer-based method implemented in Jellyfish (version 2.3.0) with values of 51-mers [30] and GenomeScope (10,000× cut-off) [31]. *K*-mers refer to all the *k*-mer frequency distributions from a read obtained through Illumina DNA sequencing. The homozygous

peak of the assembly was at a 57× coverage and the heterozygous peak was at a 28× coverage (**Fig. 3a**). The assessment of genome size by *K*-mer counting suggested a complete genome size of approximately 1.51 Gb (**Fig. 3a**), which is close to the final assembly(1.57 Gb) and cytogenetic estimates [32]. Sequence alignment between the previous assembly (1.90 Gb) [24] and the one in this study revealed considerable heterozygous redundancies in the former. This kind of overestimation of genome size usually occurs in fragmented assembly, like the recently published *M. galloprovincialis* genome [25].

Genome assembly from long-read data was carried out following three methods. First, long reads were *de novo* assembled using the Canu v1.5 software with default parameters [33]; next, error correction was performed with Racon v1.3.1 [34]. Then, further polishing with Illumina short-read data was conducted using Pilon v1.22 [35]. The final assembly was approximately 1.57 Gb in size, consisting of 6,449 contigs with an overall median length (N50) of 1.49 Mb, while the previously published draft genome only had an N50 of 0.66 Mb [24]. The present genome had a heterozygous rate of 1.39 % (also calculated by GenomeScope) and an average GC content of approximately 32%.

Anchoring of the contigs to pseudo-moleculars with Hi-C data

To complete the assembly of the hard-shelled mussel genome, Hi-C technology was carried out to generate information on the interactions among contigs. DNA from fresh adductor muscle tissue was used to prepare a Hi-C library. This was then sequenced on

the Illumina NovaSeq 6000 platform, producing 249.6 Gb of reads (**Table 1**). These reads were aligned to the assembled contigs using BWA aligner v0.7.10-r789 [36]. Lachesis v2e27abb was applied to anchor the contigs onto the linkage groups using the agglomerative hierarchical clustering method [37]. Finally, 2,029 contigs representing 90.9% of the total assemblies were successfully anchored to 14 chromosomes (**Table 2**); this number was consistent with the outputs of the karyotype [38]. The unclosed gaps only occupies 0.014% of the assembly (201,500 bp), which is filled with Ns. The N50 of the anchored contigs was over 1.7 Mb, around 1.14 times of the initial assemblies from the ONT long reads.

Genome annotation

A *de novo* repeat annotation of the hard-shelled mussel genome was carried out using RepeatModeler (version 1.0.11) [39] and RepeatMasker (version 4.0.7) [40]. RepeatModeler was used to construct the repeat library, which was then examined using two other programs, RECON and RepeatScout. The yielded consensus sequences were manually checked by aligning to the genes from the GenBank database (nt and nr; released in October 2019) to avoid that sequences of the high-copy genes are masked in following process with RepeatMasker. The final repeat library consisted of 2,264 consensus sequences with the respective classification information, which was used to run RepeatMasker against the genome assemblies. The repetitive sequences constituted a length of 735.6 Mb, representing 47.4% of the total genome length (**Supplementary Table S1**). Simple sequence repeats (SSRs) were identified using Tandem Repeats

Finder V 4.04. Only monomers, dimers, trimers, tetramers, pentamers, and hexamers with at least four repeat units were considered. The total length of the 5,324 identified SSRs was approximately 138.0 kb.

Conserved non-coding RNAs were predicted using the Rfam 11.0 databases. Putative microRNAs (miRNAs) and ribosomal RNAs (rRNAs) were predicted using Infernal (version 1.1.2) [41], and transfer RNAs (tRNAs) were predicted with tRNAscan-SE v2.0.3. A total of 9,186 miRNAs, 342 rRNAs, and 1,881 tRNAs were detected (**Supplementary Table S2**).

Protein-coding genes were predicted using a combined strategy of *ab initio* prediction, homology-based prediction, and transcriptome-based prediction (**Fig. 2**). The *ab initio* prediction was conducted using the Augustus (version 3.1) [38], GlimmerHMM (version 1.2) [39], and SNAP (version 2006-07-28) software [42]. For homology-based prediction, protein sequences of two closely related mollusk species (*Modiolus philippinarum* and *Bathymodiolus platifrons*), downloaded from GenBank, were aligned to the genome assemblies using Exonerate (version 2.2.0) [43]. In parallel, transcriptomic data from 10 tissues (GenBank SRA accession ID: PRJNA578350) were assembled *de novo* using Trinity (version 2.4.0) [44] and Cufflinks (version 2.2.1) [45]. The outputs of both assemblers were integrated using the Program to Assemble Spliced Alignments (PASA, version 2.3.3) [46]. After merging of all of these predictions using EVidenceModeler (v1.1.0) [46], a total of 37,478 final gene models were generated (**Table 3)**, a number lower than that of the previously published 42,684 gene models in the draft genome [24]. Functional annotations displayed that 35,471 protein-coding

genes (94.6% of the 37,478 gene models) have the alignment to one or more of the InterPro (version 5.22-61.0) [47], GO [48], KEGG [49], Swissprot [50] and NCBI non-redundant protein (NR) functional databases (**Table 4**; **Fig. 3b**). This information is illustrated in a genome landscape map (**Fig. 3c**). Using a bidirectional BLASTp between the two assemblies, we observed that an considerable heterozygous redundancies (over 20%) were probably included into the previous draft assemblies (Supplementary Table S3), which might be owing to the widespread hemizygosity and massive gene presence/absence variation (PAV) [25, 51] or assembling errors.

Phylogenetic analysis

Gene clusters were identified among 12 selected genomes, namely those of *Chlamys farreri* (PRJNA185465), *Pinctada fucata martensii* (GCA_002216045.1), *M. philippinarum* (GCA_002080025.1), *Crassostrea gigas* (GCF_000297895.1), *B. platifrons* (GCA_002080005.1), *Mizuhopecten yessoensis (GCA_002113885.2)*, *Penaeus vannamei* (ASM378908v1), *Pecten maximus* (GCA 902652985.1), *Scapharca* (*Anadara*) *broughtonii* (PRJNA521075), *Pomacea canaliculata* **(**PRJNA427478), *Haliotis discus hannai* (PRJNA317403), and *M. coruscus*, using OrthoMCL (version 1.4) with a BLASTp cut-off value of 10^{-5} and an inflation value of 1.5 [52]. A total of 448 single-copy genes identified by OrthoDB were aligned and concatenated. The amino acid sequences were first aligned using MUSCLE [53], and then further concatenated to create one supergene sequence for each species and form a data matrix. The phylogenetic relationships among different supergenes were then assessed using a

maximum-likelihood model in RAxML version 8 [54] with the optimal substitution model of PROTGAMMAJTT. The robustness of the maximum-likelihood tree was assessed using the bootstrap method (100 pseudo-replicates). Furthermore, single-copy orthologs and one reference divergence time on the root node obtained from the TimeTree database [55] were used to calibrate the divergence dates of other nodes on this phylogenetic tree using the $MCMC_{TREE}$ tool in the PAML package [56]. Visualization of phylogenetic relationships with FigTree (version 1.4.3) [57] suggested that *M. coruscus* is a sister taxon to the clade containing *M. philippinarum* and *B. platifrons,* with a divergence time of approximately 129 Mya (**Fig. 3d**).

Whole genome re-sequencing of farmed and wild individuals

Chromosome-level genome is important for re-sequencing and population genetic. We performed a preliminary try to detect sequence variation by sequencing two genomic DNA pools of wild population and farmed population. A total of 50.4 Gb and 46.7 Gb of Illumina clean reads were finally generated in farmed and wild samples, respectively. Over 89% reads were aligned to the reference genome with BWA (v0.7.10-r789) [36]. The PCR duplicates (duplicates introduced by PCR) were removed with MarkDuplicates in the Picard toolkit [58]. SNPs and small indels (10 bp or less) were identified with GATK (version 3.7) [59] with default parameters and the addition of three extra thresholds to discard unreliable items during post-filter analysis, namely: 1) any two SNPs located within 5 bp from each other; 2) any two indels located within 10 bp from each other; and 3) any SNPs located within 5 bp from an indel. Finally, we

identified 5,733,780 SNPs and 1,821,690 small indels in the farmed population and 5,719,771 SNPs and 1,820,404 small indels in the wild population. Similar distribution patterns of SNPs and indels were detected between the farmed and wild population (**Supplementary Fig. S1**) when nearly 99% of the SNPs/indels were shared by both populations (**Fig. 4a**), reflecting that only approximately 1% of the sequence variations were farmed population specific (FPS) or wild population specific (WPS). We focused on the differential variations located in the flanking regions and genic regions, between the farmed and wild populations, to identify candidate genes and causal mutations related to morphological traits. The software SnpEff version 2.0.5 [60] was applied to detect the effect of SNPs/indels by comparing the loci of SNPs/indels with those of protein-coding genes, which revealed that 59 genes carrying FPS SNPs/indels (FPSGs) and 57 genes carrying WPS SNPs/indels (WPSGs) underwent loss of translational start sites, gain or loss of stop codons, or variants in the acceptor/donor of splicing sites. Some variations were observed to cluster in farmed population (**Fig. 4b**), implicating a potentially influence to morphological diversity. In addition, PAV may play a role in determining phenotypic traits [25, 51], which should be included in the future re-sequencing analyses.

Chromosome synteny and evolution in bivalves

To investigate the evolution of the mussel chromosomes, gene collinearity was constructed by aligning the genes of the king scallop *P. maximus* to the reference genomes of the blood clam *S. broughtonii*, the hard-shelled mussel *M. coruscus*, the

pearl oyster *P. martensii*, and the Pacific oyster *C. gigas* using MCscan (version 0.8). The parameters of the MCscan alignment were set as -s, 7; k, 150; m, 250; e, $1e^{-10}$. We identified 404 scallop-vs-clam, 276 scallop-vs-mussel, 159 scallop-vs-pearl-oyster, and 232 scallop-vs-pacific-oyster syntenic blocks, which included 10,055, 4,716, 3,636, and 5,009 genes of blood clam, hard-shelled mussel, pearl oyster and Pacific oyster, respectively. The mean gene number per syntenic block was 21.4. King scallop and blood clam had the highest gene collinearity, consistent with their close phylogenetic relationship in the Bivalvia clade [61] (Fig. 3d). The chromosome synteny illustrated that large-scale rearrangements are rare between scallop and mussel, but frequent between scallop and oysters (Fig. 5b–d), as exemplified by considerable structural variations between the scallop and the Pacific oyster genomes (Fig. 5d). The identified cross-chromosome rearrangements between the scallop and mussel genomes were different from those between the genomes of scallop and the two oyster species (Fig. 5b–e). The scallop linkage groups (PM) 1, 5, 6, 8, 10, 16, 17, 18, and 19 were syntenic to a single mussel chromosome (MC) 8, 9, 3, 4, 10, 13, 11, 12, and 14, respectively. PM 2 and 15 were aligned to the same reference, MC 8; similarly, PM 3 and 14 aligned to MC 5, PM 4 and 7 aligned to MC 1, PM 9 and 12 aligned to MC 7, and PM 11 and 13 aligned to MC 6. Comparatively, some additional chromosome rearrangements occurred between scallop and the two oyster species, especially the Pacific oyster. Both the Pacific oyster chromosome 9 and the pearl oyster chromosome 7 were predominantly syntenic to the scallop PM 15, suggesting that they might carry conserved genomic regions with the same origin (Fig. 5c–e). Among all the syntenic

chromosomes, we did not observe any chromosome to be entirely conserved in all of the bivalve genomes. Intriguingly, almost all of the chromosome rearrangements between the mussel and the oyster genomes were different (Fig. 5e), implicating independent chromosome fusion events. The identification of such diverse chromosome rearrangements suggested a complex evolutionary history of bivalve chromosomes.

Metamorphosis-related transcriptome analysis

To profile gene expression during development and metamorphosis in hard-shelled mussels, RNA-seq analysis was conducted at five developmental stages: trochophore, D-veliger, umbo, pediveliger, and juvenile (PRJNA689932). The quantification of gene expression enabled the detection of 33,743 transcripts with the TPM > 0 at all stages (**Supplementary Table S4**). The limma statistical method was used to detect DEGs based on linear models [62]. Using the trochophore as control, 5,795; 6,163; 9,308; and 7,486 upregulated genes [$\log_2$(fold-change) > 1 and adjusted $P < 0.05$] were identified in D-veliger, umbo, pediveliger, and juvenile larvae, respectively. Functional annotation indicated that these were mainly involved in "environmental information processing" ("signal transduction" and "signaling molecules and interaction") and "cellular processes" ("transport and catabolism"), in agreement with the key role of signal transduction and the endocrine system in larval development [17].

Since the ability to effectuate metamorphosis develops during the pediveliger

stage [17], we investigate the 774 up-regulated genes during the transition from the umbo to the pediveliger stage. Functional annotation revealed that they were mainly employed in a network of six related pathways: “adrenergic signaling in cardiomyocytes,” “calcium signaling pathway,” “MAPK signaling pathway,” “protein export,” “endocytosis,” and “catecholamine biosynthesis” (Fig. 6a), which have been reported to be involved in settlement and metamorphosis [18, 63]. The expression of most of the genes involved in these pathways increased during one or more periods (Fig. 6b). Among them, 20 genes have been functionally identified to be associated with metamorphosis (**Supplementary Table S5**) and 26 up-regulated encompassing from the umbo to the pediveliger stages belonged to the categories “adrenergic signaling in cardiomyocytes,” “calcium signaling pathway,” and “catecholamine transport”, which was consistent with the findings of a recent proteome study on larval settlement and the metamorphosis of oysters [63-66]. Although some additional pathways, such as “phagosome” and “oxytocin signaling pathway”, are also detected, we did not analyze them in detail because still lacking evidence on their involvement in metamorphosis. In summary, the analysis of the involved pathways revealed that biosynthesis, transport, and transduction of catecholamines might be critical for the completion of metamorphosis.

Assembly assessment

The quality of the assembled genome was validated in terms of completeness, accuracy of the assemblies, and conservation of synteny. Alignment of Illumina reads against the

reference genome revealed insert sizes of paired-end sequencing libraries of approximately 300–350 bp and a mapping rate of over 96.7%. We assayed the genome completeness using Benchmark Universal Single-Copy Orthologs BUSCO v4.1.4 referencing metazoan and molluscan gene sets. In the metazoan dataset, the current assemblies have 89.4% complete (of which 1.0% were duplicated), 1.9% incomplete and 8.7% missing BUSCOs, corresponding to a recovery of 91.3% of the entire BUSCO set. In the molluscan dataset, 85.5% complete (of which 1.3% were duplicated), 0.8% incomplete and 13.7% missing BUSCOs were recorded, corresponding to 86.3% of the entire BUSCO set. Motifs with the characteristics of telomeric repeats were detected in 23 termini of the 13 chromosomes, suggesting the completeness of the assemblies (Supplementary Table S6). The accuracy of the genome assembly was evaluated by calling sequence variants through the alignment of Illumina sequencing data against the genome. Sequence alignment with the BCFtools (version 1.3) [67] revealed 368,991 homozygous SNP loci, reflecting an error rate of less than 0.02% in the genome assembly. In addition, the highly conserved synteny and the strict correspondence of chromosome fusion points and gene assignment identified between the hard-shelled mussel and king scallop genomes (Fig. 5b) were indicative of a qualified assembly of the hard-shelled mussel genome, since the king scallop genome is considered as the best-scaffolded genome available for bivalves [68].

Conclusion

The chromosome-level assembly of the hard-shelled mussel genome presented here is

a well-assembled and annotated resource that would facilitate a wide range of research in mussels, bivalves, and mollusks. The outputs of this study shed light on the chromosome evolution in bivalves, resulting in the regulation of the molecular pathways involved in larval metamorphosis. As one of the chromosome-level genome assemblies of bivalves, this genome data set will serve as a high-quality genome platform for comparative genomics at the chromosome level.

Availability of Supporting Data and Materials

All of the raw Illumina and ONT reads were deposited to NCBI Sequence Read Archive and the assembled genome was deposited to GenBank under the accession number PRJNA578350. The corresponding genome sequences and read alignments (VCF files) were stored in Figshare [69] and GigaDB [68].

Abbreviations

TPM: the Transcripts per Million; GATK: Genome Analysis Tool Kit; GO: Gene Ontolog; KEGG: Kyoto Encyclopedia of Genes and Genomes; AC1: adenylate cyclase 1; AC10: adenylate cyclase 10; Akt: RAC serine/threonine-protein kinase; CaM: calmodulin; CaMKII: calcium/calmodulin-dependent protein kinase (CaM kinase) II; CAV1: caveolin 1; CAV3: caveolin 3; CREB: cyclic AMP-responsive element-binding protein; DBH: dopamine beta-monooxygenase; DDC: aromatic-L-amino-acid decarboxylase; DHPR: voltage-dependent calcium channel gamma-1; Epac: Rap guanine nucleotide exchange factor; ERK: mitogen-activated protein kinase 1/3; Gi: guanine nucleotide-binding protein G(i) subunit alpha; Gq: guanine nucleotide-binding

protein G(q) subunit alpha; Gs: guanine nucleotide-binding protein G(s) subunit alpha; ICER: cAMP response element modulator; IKS: potassium voltage-gated channel KQT-like subfamily member 1; IMP2: mitochondrial inner membrane protease subunit 2; INaK: sodium/potassium-transporting ATPase subunit alpha; MAOA: monoamine oxidase A; MAOB: monoamine oxidase B; MSK1: ribosomal protein S6 kinase alpha-5; NCX : solute carrier family 8 (sodium/calcium exchanger); NF-κB: nuclear factor NF-kappa-B p105 subunit; NHE: solute carrier family 9 (sodium/hydrogen exchanger); p38MAPK: p38 MAP kinase; PI3K: phosphatidylinositol-4,5-bisphosphate 3-kinase catalytic subunit alpha/beta/delta; PKA: protein kinase A; PKCα: classical protein kinase C alpha type; PLC: phosphatidylinositol phospholipase C; PP1: serine/threonine-protein phosphatase PP1 catalytic subunit; TnI: Troponin I; TPM: tropomyosin; TYR: tyrosinase; α-ARA: alpha-1A adrenergic receptor-like; α-ARB: adrenergic receptor alpha-1B; β2AR: adrenergic receptor beta-2.

Competing Interests

The authors declare no competing interests.

Funding

This study was supported by the National Key Research and Development Program of China (2018YFD0900601), Key Program for International Science and Technology Cooperation Projects of Ministry of Science and Technology of China (No. 2018YFD0900101), the National Natural Science Foundation of China (No. 41876159,

No. 41606147, No. 31802321), Key Special Project for Introduced Talents Team of Southern Marine Science and Engineering Guangdong Laboratory (Guangzhou) (GML2019ZD0402), the Shanghai Sailing Program (18YF1410000), China Postdoctoral science foundation (2019M6614770) and Program for study on genetic resources, environment and strategy of mussel culture in coast of Gouqi Island offshore.

Authors Contributions

J.L.Y., Y.L. and X.L. designed and supervised the study. K.C., J.K.X, Y.T.Z, Y.F.L. collected the samples and extracted the genomic DNA and RNA. Y.L., J.L. and D.D.F. performed genome assembly and bioinformatics analysis. J.L.Y., D.D.F., X.L., J.L. and Y.L. wrote the original manuscript. All authors reviewed the manuscript.

Figure legends

Figure 1. Sequenced individuals and sampling sites. **a.** Pictures of the sequenced individuals collected in Shengsi. A wild *M. coruscus* adult was used for genome sequencing. Both wild and farmed populations were used for re-sequencing. **b.** The geographic locations of the sampling sites.

Figure 2. Workflow of genome sequencing and annotation. The rectangles indicate the steps of data treatment and the diamonds indicate output or input data.

Figure 3. Annotation and evolution. **a.** GenomeScope plot of the 51-mer k-mer content within the hard-shelled mussel genome. Estimates of genome size and read data were shown. **b.** Venn diagram indicating the number of genes that were annotated in one or more databases. **c.** Genomic landscape of *M. coruscus*. The chromosomes were labeled as LG01 to LG14. From the outer to the inner circle: 5, marker distribution across 14 chromosomes at a megabase scale; 4, gene density across the whole genome; 3, SNP density; 2 and 1, number of repetitive sequences and GC content across the genome. 1–5 are drawn in non-overlapping 0.1-Mb sliding windows. The length of chromosomes is defined by the scale (Mb) on the outer circles. **d.** Phylogenetic tree based on protein sequences from 12 metazoan genomes, namely those of *Chlamys farreri* (PRJNA185465), *Pinctada fucata martensii* (GCA_002216045.1), *Modiolus philippinarum* (GCA_002080025.1), *Crassostrea gigas* (GCF_000297895.1), *Mytilus coruscus*, *Bathymodiolus platifrons* (GCA_002080005.1), *Mizuhopecten yessoensis*

(GCA_002113885.2), *Penaeus vannamei* (ASM378908v1), *Pecten maximus* (GCA 902652985.1), *Scapharca* (*Anadara*) *broughtonii* (PRJNA521075), *Pomacea canaliculata* (PRJNA427478), and *Haliotis discus hannai* (PRJNA317403).

Figure 4. Sequence variations between farmed and wild populations**. a.** Venn diagrams showing the number and distribution of indels and SNPs between the farmed and wild populations. **b.** Differences in the number of SNPs on the exons of chitobiase. The rectangles indicate the 14 exons of the chitobiase gene and the lines between the 14 rectangles indicate introns; the pink matrix represents reads from the farmed population, and the blue matrix represents reads from the wild population. Bases denoted by capital letters are located on exons, whereas those denoted by small letters are located on introns.

Figure 5. Chromosome synteny. **a.** Alignment of king scallop and blood clam chromosomes. **b.** Alignment of king scallop and hard-shelled mussel chromosomes. **c**. Alignment of king scallop and pearl oyster chromosomes. **d**. Alignment of king scallop and Pacific oyster chromosomes. The king scallop linkage groups are labeled as PM 1 to 19, the blood clam chromosomes as SB 1 to 19, the hard-shelled mussel chromosomes as MC 1 to 14, the pearl oyster chromosomes as PF 1 to 14, and the Pacific oyster chromosomes as CG 1 to 10. Scale unit, Mb. **a–d.** The circularized blocks represent the chromosomes of the five bivalves. Aligned homologous genes are connected by ribbons, shown in different colors depending on their chromosome

location. **e.** Rearrangements between the chromosomes of king scallop and those of four other bivalve species. The king scallop chromosomes are represented by bars of different colors, and synteny and rearrangements in the chromosomes of the four other bivalves are indicated by different blocks, whose colors correspond to those of the reference king scallop chromosomes, the dashed lines indicate the corresponding evolution relationship.

Figure 6. Spatial and temporal expression of genes involved in development and metamorphosis. **a.** Expression pattern of genes implied in the pathways of catecholamine biosynthesis and adrenergic signaling in cardiomyocytes, according to KEGG-based annotation. Red rectangles indicate upregulated genes during development， red rectangles with black edge indicate upregulated genes at Pediveliger stage and metamorphosis, and white rectangles denote genes that were identified during KEGG analysis but whose expression did not change. Red bubbles represent the most important pathways in which the upregulated genes are involved. **b.** Heatmap showing the expression levels of all genes involved in the pathways of catecholamine biosynthesis and adrenergic signaling in cardiomyocytes across five developmental stages.

Table captions

Table 1. Statistics of whole genome sequencing using Illumina and ONT

Table 2. Results of contig anchoring on pseudochromosomes using Hi-C data

Table 3. General statistics of the predicted protein-coding genes

Table 4. General statistics of gene functional annotation

Additional Files

Supplementary Table S1. Repetitive sequences in the hard-shelled mussel genome

Supplementary Table S2. Overview of the predicted non-coding RNAs

Supplementary Table S3. Bidirectional BLASTp between the previously published gene models of the hard-shelled mussel and the predicted gene models in this study.

Supplementary Table S4. Gene expression profiles during five developmental stages

Supplementary Table S5. Genes involved in the pathways of catecholamine biosynthesis and adrenergic signaling in the cardiomyocytes were reported to affect metamorphosis.

Supplementary Table S6. Information of the motifs with the characteristic of telomeric repeats

Supplementary Figure S1. Circles showing genome-wide SNPs and indels from the farmed and wild populations. From the outer to the inner circle: first circle, marker distribution across 14 pseudochromosomes at a megabase scale; green circle, SNP density across the whole genome; red circle, indel density.

References

1. FAO. The state of world fisheries and aquaculture. 2018.
2. Amini S, Kolle S, Petrone L, et al. Preventing mussel adhesion using lubricant-infused materials. Science 2017; **357**:668-673.
3. Yang JL, Li YF, Guo XP, et al. The effect of carbon nanotubes and titanium dioxide incorporated in PDMS on biofilm community composition and subsequent mussel plantigrade settlement. Biofouling 2016; **32**:763-777.
4. Yang JL, Shen PJ, Liang X, et al. Larval settlement and metamorphosis of the mussel *Mytilus coruscus* in response to monospecific bacterial biofilms. Biofouling 2013; **29**:247-259.
5. Liang X, Peng LH, Zhang S, et al. Polyurethane, epoxy resin and polydimethylsiloxane altered biofilm formation and mussel settlement. Chemosphere 2019; **218**:599-608.
6. Odonnell MJ, George MN, Carrington E. Mussel byssus attachment weakened by ocean acidification. Nature Climate Change 2013; **3**:587-590.
7. Ramesh K, Hu MY, Thomsen J, et al. Mussel larvae modify calcifying fluid carbonate chemistry to promote calcification. Nature Communications 2017; **8**:1709.
8. Thomsen J, Stapp L, Haynert K, et al. Naturally acidified habitat selects for ocean acidification–tolerant mussels. Science Advances 2017; **3**:e1602411.
9. Bitter MC, Kapsenberg L, Gattuso J, et al. Standing genetic variation fuels rapid adaptation to ocean acidification. Nature Communications 2019; **10**:1-10.
10. Briand J. Marine antifouling laboratory bioassays: an overview of their diversity. Biofouling 2009; **25**:297-311.
11. Petrone L, Kumar A, Sutanto CN, et al. Mussel adhesion is dictated by time-regulated secretion and molecular conformation of mussel adhesive proteins. Nature Communications 2015; **6**:8737-8737.
12. Zeng ZS, Guo XP, Cai XS, et al. Pyomelanin from *Pseudoalteromonas lipolytica* reduces biofouling. Microbial Biotechnology 2017; **10**:1718-1731.
13. Murgarella M, Puiu D, Novoa B, et al. A first insight into the genome of the filter-feeder mussel *Mytilus galloprovincialis*. PLoS One 2016; **11**:e0151561.
14. Sun J, Zhang Y, Xu T, et al. Adaptation to deep-sea chemosynthetic environments as revealed by mussel genomes. Nature Ecology and Evolution 2017; **1**:0121.
15. Hadfield MG, Paul VG. In *Marine chemical ecology* (ed. McClintock, J.B & Baker, J.B) Ch13. CRC Press, 2001.
16. Dobretsov S, Rittschof D. Love at first taste: induction of larval settlement by marine microbes. International Journal of Molecular Sciences 2020; **21**:731.
17. Hadfield MG. Biofilms and marine invertebrate larvae: what bacteria produce that larvae use to choose settlement sites. Annual Review of Marine Science 2011; **3**:453-470.
18. Shikuma NJ, Antoshechkin I, Medeiros JM, et al. Stepwise metamorphosis of the tubeworm *Hydroides elegans* is mediated by a bacterial inducer and MAPK signaling. Proceedings of the National Academy of Sciences of the United States of America 2016; **113**:10097-10102.
19. Shikuma NJ, Pilhofer M, Weiss GL, et al. Marine tubeworm metamorphosis induced by arrays of bacterial phage tail–like structures. Science 2014; **343**:529-533.

20. Kulikova VA, Lyashenko SA, Kolotukhina NK. Seasonal and interannual dynamics of larval abundance of *Mytilus coruscus* Gould, 1861 (Bivalvia: Mytilidae) in Amursky Bay (Peter the Great Bay, Sea of Japan). Russian Journal of Marine Biology 2011; **37**:342-347.
21. Li YF, Liu YZ, Chen YW, et al. Two toll-like receptors identified in the mantle of *Mytilus coruscus* are abundant in haemocytes. Fish & shellfish immunology 2019; **90**:134-140.
22. Liang X, Zhang XK, Peng LH, et al. The flagellar gene regulates biofilm formation and mussel larval settlement and metamorphosis. International Journal of Molecular Sciences 2020; **21**:710.
23. Yang JL, Li SH, Li YF, et al. Effects of neuroactive compounds, ions and organic solvents on larval metamorphosis of the mussel *Mytilus coruscus*. Aquaculture 2013; **396-399**:106-112.
24. Li RH, Zhang WJ, Lu JK, et al. The whole-genome sequencing and hybrid assembly of *Mytilus coruscus*. Frontiers in Genetics 2020; **11**:1-6.
25. Gerdol M, Moreira R, Cruz F, et al. Massive gene presence-absence variation shapes an open pan-genome in the Mediterranean mussel. Genome Biology 2020; **21**:275.
26. Li YL, Sun XQ, Hu XL, et al. Scallop genome reveals molecular adaptations to semi-sessile life and neurotoxins. Nature Communications 2017; **8**:1721-1721.
27. Wang S, Zhang J, Jiao W, et al. Scallop genome provides insights into evolution of bilaterian karyotype and development. Nature ecology & evolution 2017; **1**:0120.
28. Sokolov EP. An improved method for DNA isolation from mucopolysaccharide-rich molluscan tissues. Journal of Molluscan Studies 2000; **66**:573-575.
29. Van Berkum NL, Lieberman-Aiden E, Williams L, et al. Hi-C: A method to study the three-dimensional architecture of genomes. Journal of Visualized Experiments 2010; **39**:e1869.
30. Marçais G, Kingsford C. A fast, lock-free approach for efficient parallel counting of occurrences of k-mers. Bioinformatics 2011; **27**:764-770.
31. Vurture GW, Sedlazeck FJ, Nattestad M, et al. GenomeScope: fast reference-free genome profiling from short reads. Bioinformatics 2017; **33**:2202-2204.
32. Ieyama H, Kameoka O, Tan T, et al. Chromosomes and nuclear DNA contents of some species in Mytilidae. Venus (Japanese Journal of Malacology) 1994; **53**:327-331.
33. Koren S, Walenz BP, Berlin K, et al. Canu: scalable and accurate long-read assembly via adaptive k-mer weighting and repeat separation. Genome research 2017; **27**:722-736.
34. Vaser R, Sović I, Nagarajan N, et al. Fast and accurate de novo genome assembly from long uncorrected reads. Genome research 2017; **27**:737-746.
35. Walker BJ, Abeel T, Shea T, et al. Pilon: an integrated tool for comprehensive microbial variant detection and genome assembly improvement. PloS one 2014; **9**:e112963.
36. Li H, Durbin R. Fast and accurate short read alignment with Burrows–Wheeler transform. Bioinformatics 2009; **25**:1754-1760.
37. Burton JN, Adey A, Patwardhan RP, et al. Chromosome-scale scaffolding of de novo genome assemblies based on chromatin interactions. Nature Biotechnology 2013; **31**:1119-1125.
38. Zhuang BX. A preliminary study on the chromosome of marine bivalve, *Mytilus coruscus*. Zoological Research 1984; **S2**.
39. Smit A, Hubley R. RepeatModeler Open-1.0. 2008:http://www.repeatmasker.org/.
40. Smit A, Hubley R, Green P. RepeatMasker Open-4.0. 2015:http://www.repeatmasker.org/.

41. Nawrocki EP, Eddy SR. Infernal 1.1: 100-fold faster RNA homology searches. Bioinformatics 2013; **29**:2933-2935.
42. Korf I. Gene finding in novel genomes. BMC Bioinformatics 2004; **5**:59.
43. Slater GSC, Birney E. Automated generation of heuristics for biological sequence comparison. BMC bioinformatics 2005; **6**:31.
44. Grabherr MG, Haas BJ, Yassour M, et al. Trinity: reconstructing a full-length transcriptome without a genome from RNA-Seq data. Nature biotechnology 2011; **29**:644.
45. Trapnell C, Williams BA, Pertea G, et al. Transcript assembly and quantification by RNA-Seq reveals unannotated transcripts and isoform switching during cell differentiation. Nature biotechnology 2010; **28**:511.
46. Haas BJ, Salzberg SL, Zhu W, et al. Automated eukaryotic gene structure annotation using EVidenceModeler and the Program to Assemble Spliced Alignments. Genome biology 2008; **9**:R7.
47. Zdobnov EM, Apweiler R. InterProScan–an integration platform for the signature-recognition methods in InterPro. Bioinformatics 2001; **17**:847-848.
48. Ashburner M, Ball CA, Blake JA, et al. Gene ontology: tool for the unification of biology. Nature genetics 2000; **25**:25.
49. Kanehisa M, Goto S, Kawashima S, et al. The KEGG resource for deciphering the genome. Nucleic Acids Research 2004; **32**:277-280.
50. Boeckmann B, Bairoch A, Apweiler R, et al. The SWISS-PROT protein knowledgebase and its supplement TrEMBL in 2003. Nucleic acids research 2003; **31**:365-370.
51. Calcino AD, Kenny NJ, Gerdol M. Single individual structural variant detection uncovers widespread hemizygosity in molluscs. bioRxiv 2020:298695.
52. Li L, Stoeckert CJ, Roos DS. OrthoMCL: identification of ortholog groups for eukaryotic genomes. Genome research 2003; **13**:2178-2189.
53. Edgar RC. MUSCLE: multiple sequence alignment with high accuracy and high throughput. Nucleic acids research 2004; **32**:1792-1797.
54. Stamatakis A. RAxML-VI-HPC: maximum likelihood-based phylogenetic analyses with thousands of taxa and mixed models. Bioinformatics 2006; **22**:2688-2690.
55. Kumar S, Stecher G, Suleski M, et al. TimeTree: a resource for timelines, timetrees, and divergence times. Mol Biol Evol 2017; **34**:1812-1819.
56. Yang Z. PAML: a program package for phylogenetic analysis by maximum likelihood. Computer applications in the biosciences 1997; **13**:555-556.
57. Rambaut A. FigTree, a graphical viewer of phylogenetic trees. 2007:http://tree.bio.ed.ac.uk/software/figtree/.
58. PicardToolkit. Broad Institute, GitHub Repository 2019:http://broadinstitute.github.io/picard/.
59. Mckenna A, Hanna M, Banks E, et al. The Genome Analysis Toolkit: A MapReduce framework for analyzing next-generation DNA sequencing data. Genome Research 2010; **20**:1297-1303.
60. Cingolani P, Platts AE, Wang LL, et al. A program for annotating and predicting the effects of single nucleotide polymorphisms, SnpEff: SNPs in the genome of *Drosophila melanogaster* strain w1118. Fly 2012; **6**:80-92.
61. Liu FY, Li YL, Yu HW, et al. MolluscDB: an integrated functional and evolutionary

genomics database for the hyper-diverse animal phylum Mollusca. Nucleic Acids Res 2020;**49**:D1556.

62. Smyth GK, Ritchie M, Thorne N, et al. LIMMA: linear models for microarray data. In Bioinformatics and Computational Biology Solutions Using R and Bioconductor. Statistics for Biology and Health. 2005.
63. Di G, Xiao X, Tong MH, et al. Proteome of larval metamorphosis induced by epinephrine in the Fujian oyster *Crassostrea angulata*. BMC Genomics 2020; **21**:675.
64. Eisenhofer G, Tian H, Holmes C, et al. Tyrosinase: a developmentally specific major determinant of peripheral dopamine. The FASEB Journal 2003; **17**:1248-1255.
65. Bonar DB, Coon SL, Walch M, et al. Control of oyster settlement and metamorphosis by endogenous and exogenous chemical cues. Bulletin of Marine Science 1990; **46**:484-498.
66. Joyce A, Vogeler S. Molluscan bivalve settlement and metamorphosis: neuroendocrine inducers and morphogenetic responses. Aquaculture 2018; **487**:64-82.
67. Narasimhan VM, Danecek P, Scally A, et al. BCFtools/RoH: a hidden Markov model approach for detecting autozygosity from next-generation sequencing data. Bioinformatics 2016; **32**:1749-1751.
68. Kenny NJ, Mccarthy S, Dudchenko O, et al. The Gene-Rich Genome of the Scallop *Pecten maximus*. GigaScience 2020;**9**:giaa037.
69. Feng DD. The hard-shelled mussel *Mytilus coruscus* gene models, annotatins and related files of the whole genome. Figshare 2020:doi:10.6084/m6089.figshare.10259618.

Figure 1. Sequenced individuals and sampling sites.

Click here to access/download;Figure;Fig1 Sequenced individuals and sampling sites.tif

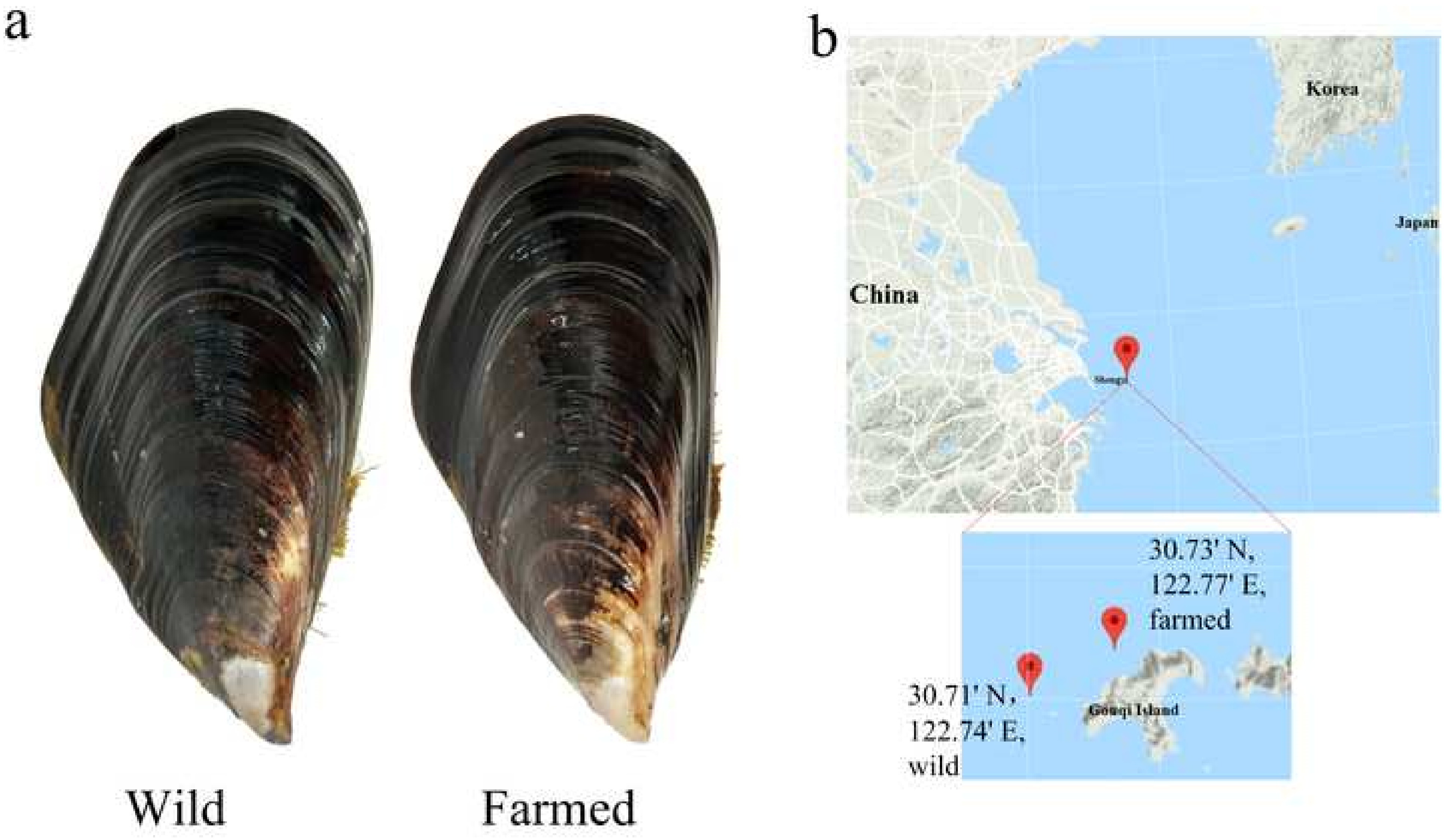

Figure 2. Workflow of genome sequencing and annotation

Click here to access/download;Figure;Figure 2. Workflow of genome sequencing and annotation.png

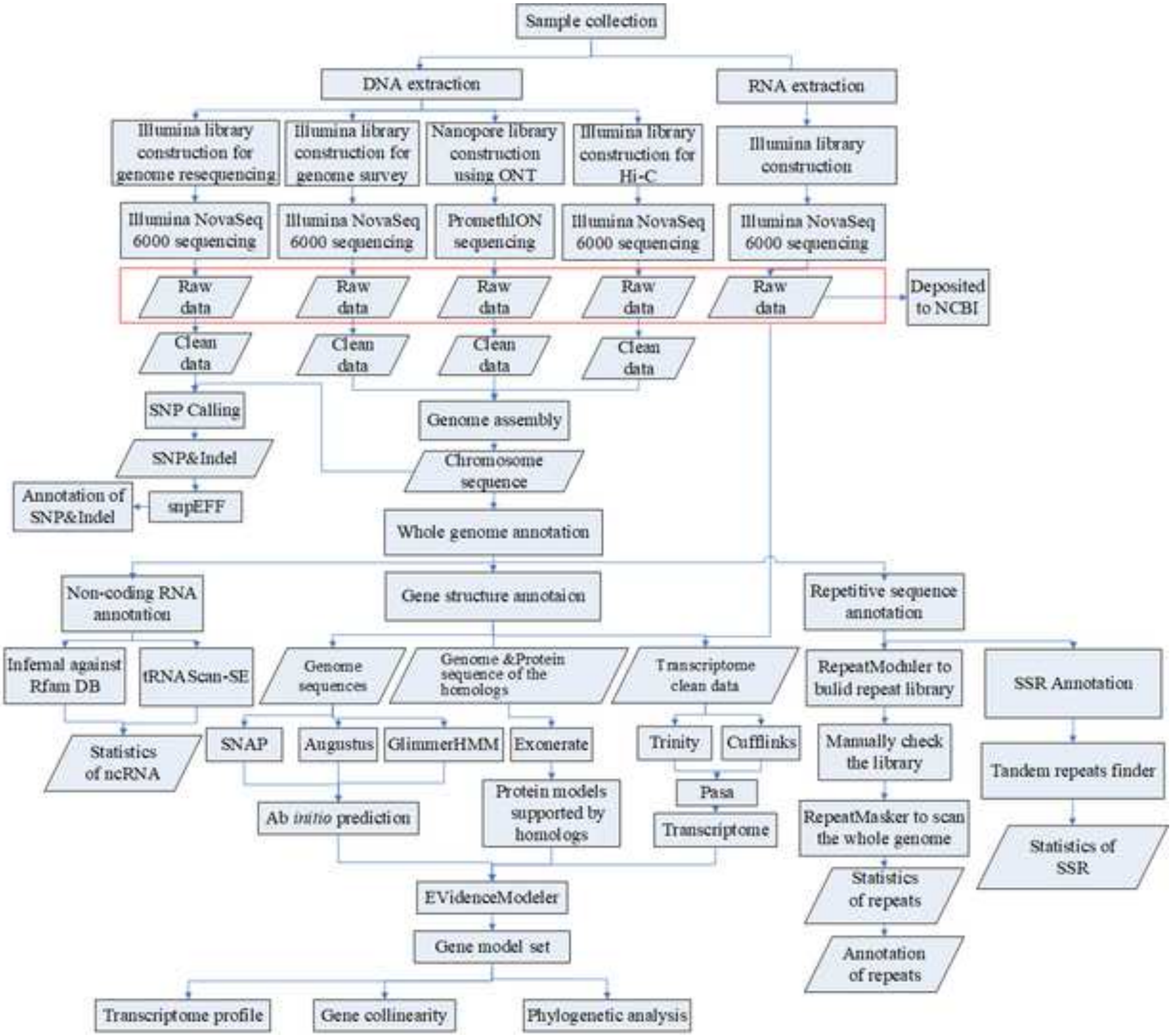

Figure 3. Annotation and evolution

Click here to access/download;Figure;Fig3 genome annotation and evolution.jpg

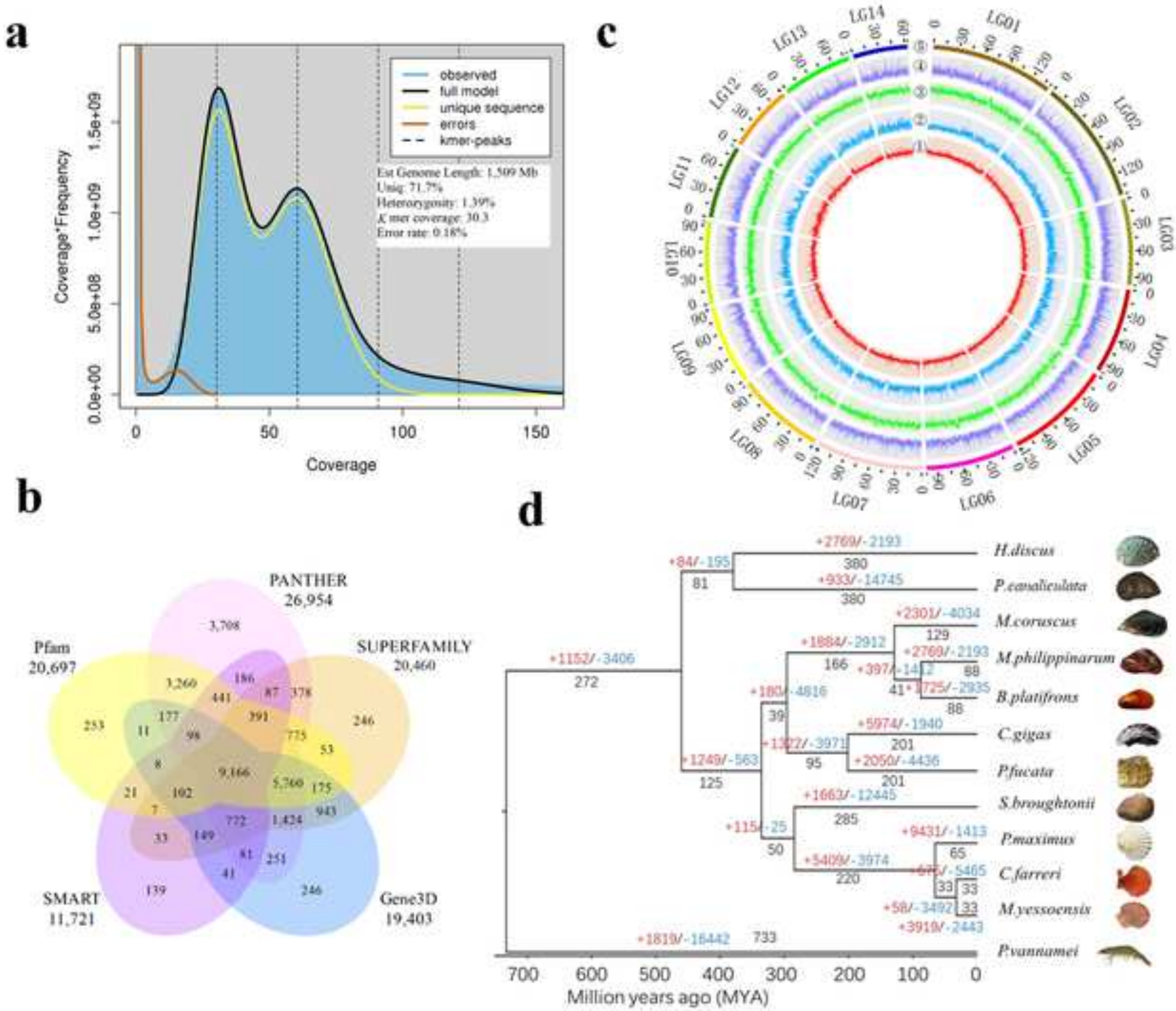

Figure 4. Sequence variations between farmed and wild populations

Click here to access/download;Figure;Fig4 Sequence variations between farmed and wild populations.png

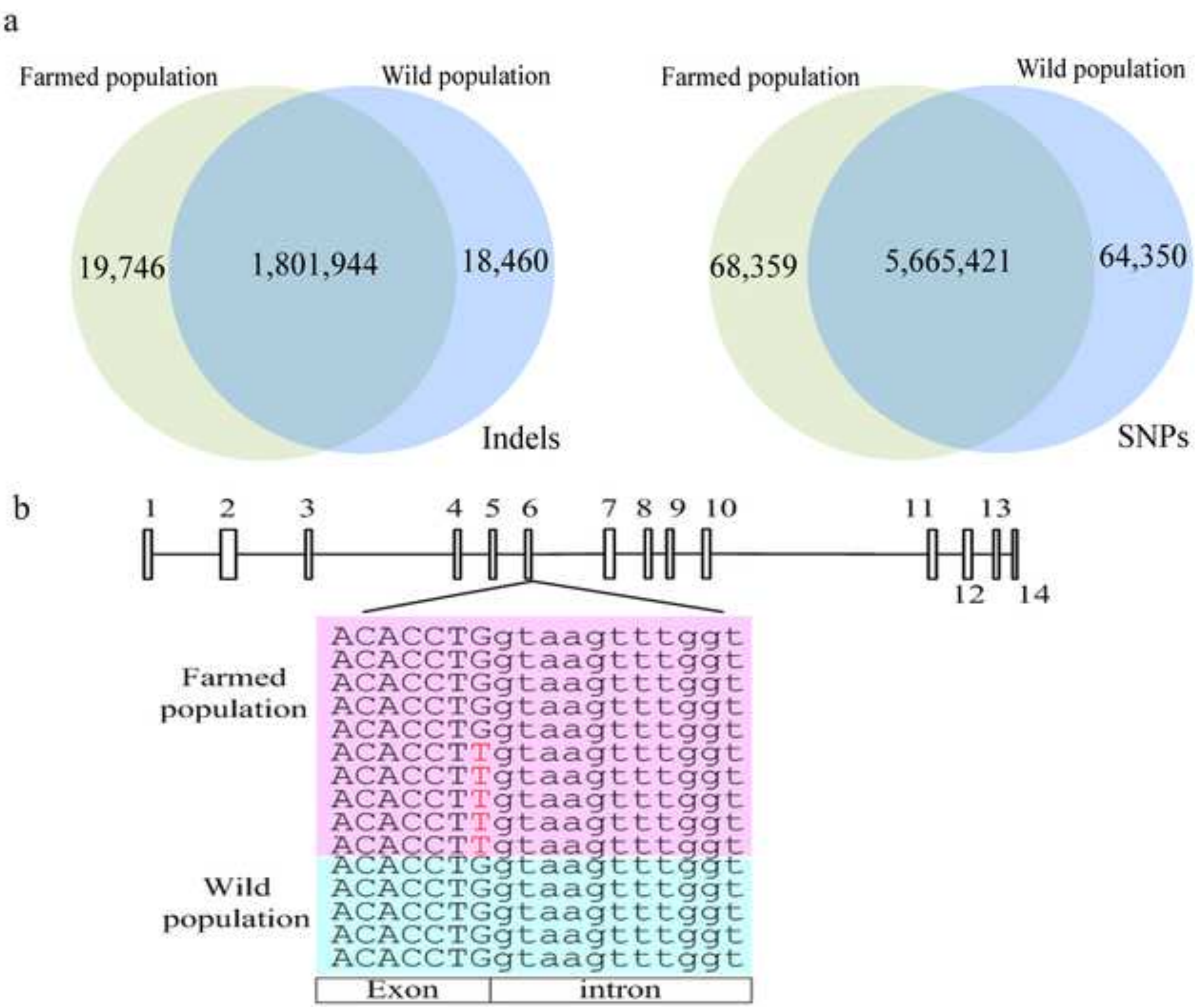

Figure 5. Chromosome synteny

Click here to access/download;Figure;Fig5 Chromosome synteny.jpg

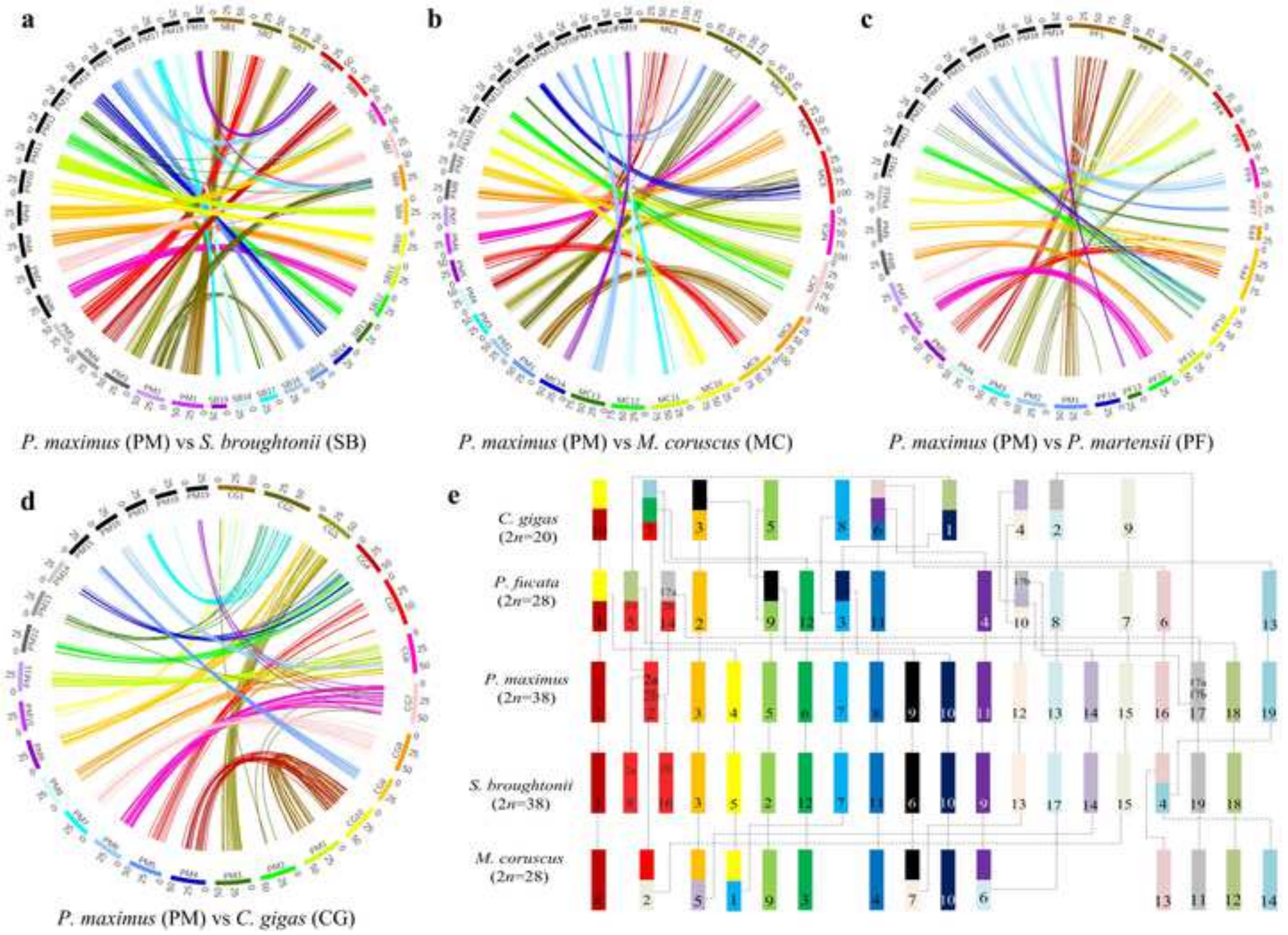

Figure 6. Spatial and temporal expression of genes involved in development and metamorphosis

Click here to access/download;Figure;Fig6 DoapmineSynpatic.png

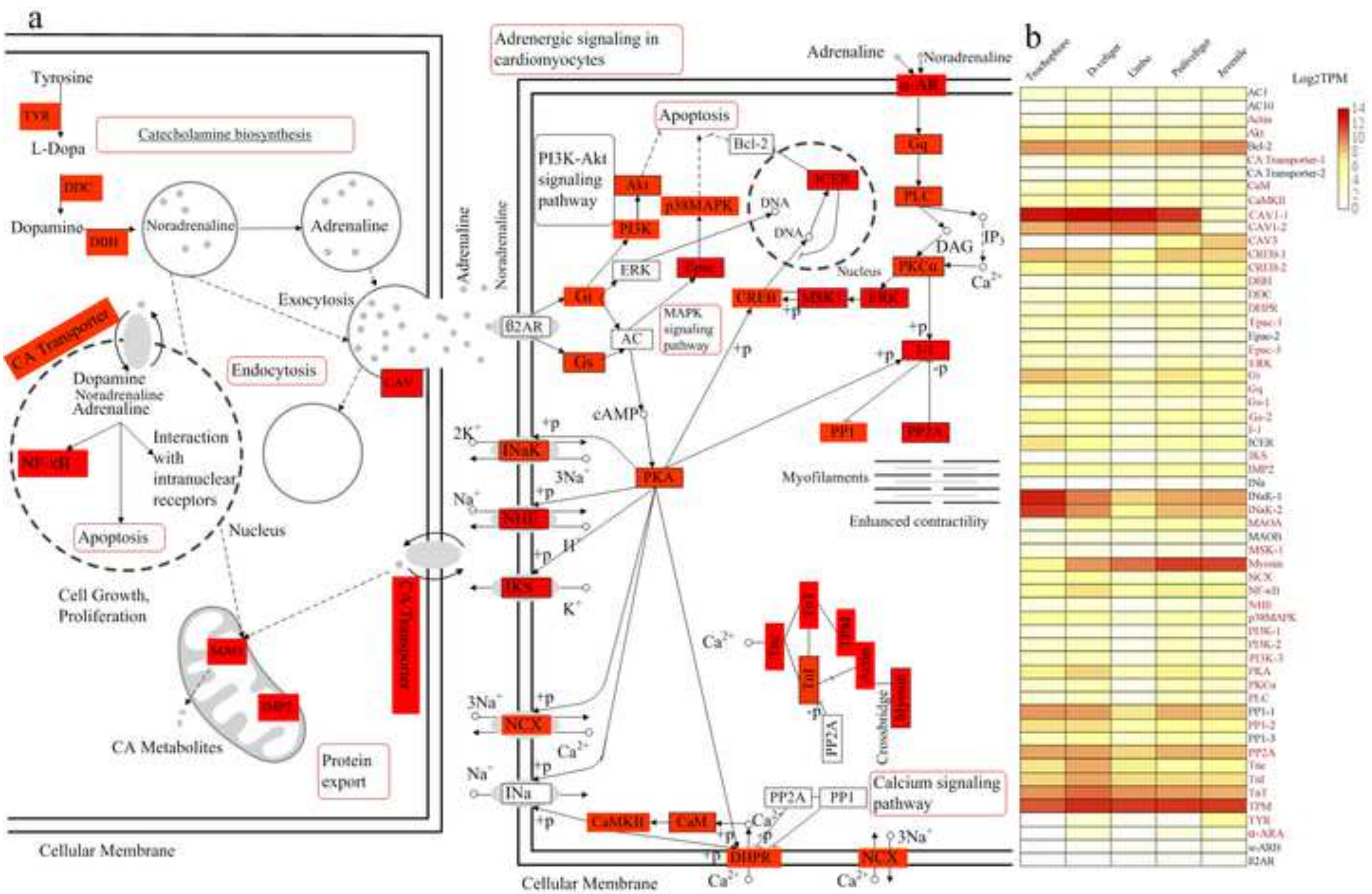

Supplementary Table S1. Repetitive sequences in the hard-shelled mussle genome

Click here to access/download
Supplementary Material
Supplementary Table S1 Repetitive sequences.xls

Supplementary Table S2. Overview of predicted non-coding RNAs

Click here to access/download
Supplementary Material
Supplementary Table S2 non-coding RNAs.xls

Supplementary Table S3. Bidirectional BLASTp

Click here to access/download
Supplementary Material
Supplementary Table S3 Bidirectional BLASTp.docx

Supplementary Table S4. Gene expression profiles during five developmental stages

Click here to access/download
Supplementary Material
Supplementary Table S4 The gene expression profile.xlsx

Supplementary Table S5. Genes involved in metamorphosis

Click here to access/download
Supplementary Material
Supplementary Table S5 Metamorphosis.docx

Supplementary Table S6. Information of the motifs with the characteristic of telomeric repeats

Click here to access/download
Supplementary Material
Supplementary Table S6 Telomeric.xlsx

Personal Cover
Click here to access/download;Personal Cover;Cover_letter_Jan22Revision.docx

GIGA-D-20-00287R1

Dear Editor Prof. Hans Zauner,

We would like to appreciate the editors and the reviewers for taking the time to review the manuscript entitled "Chromosome-level genome assembly of the hard-shelled mussel *Mytilus coruscus*, a widely distributed species from the temperate areas of East Asia ". According to the reviewers' comments, the manuscript is revised by improving the experiments and the descriptions. **All of the corresponding alterations made in the revised main text are highlighted in red**. A point-by-point letter is uploaded to address the comments. At the same time, we have taken the opportunity to make small corrections elsewhere in the revised main test, none of which affect substance. The related data and codes are now available in the public database.

Main alterations in the revision:

1) More comparisons in both genome size estimation and sequence quality are conducted between previously published draft genome and present assembly. Outputs of the different analyses are displayed in the main text and supplementary files.

2) Analysis of metamorphosis-related transcriptome is improved by using three biological replicates' RNA-Seq data of each developmental stage and normalized gene expression levels TPM instead of FPKM. The strong claims are moderated in the revision. This section is also supported by China Postdoctoral Science Foundation (No. 2019M6614770).

3) The section of genome re-sequencing for farmed and wild individuals is re-organized to illustrate the diversities between farmed and wild populations and weaken the claims made in the last version since the re-sequence study is just a preliminary try in genome research.

Looking forward to receiving your positive reply.

Best regards.

Jin-Long Yang (jlyang@shou.edu.cn) & Ying Lu (yinglu@shou.edu.cn)

College of Fisheries and Life Science, Shanghai Ocean University, Shanghai, China
