# Supplementary material for: Chromosome-level genome assembly of the hard-shelled mussel Mytilus coruscus, a widely distributed species from the temperate areas of East Asia
Source: Gigascience. 2021 Apr 23;10(4):giab024. doi: 10.1093/gigascience/giab024 (PMC8063583; doi:10.1093/gigascience/giab024)
Supplement: giab024_GIGA-D-20-00287_Revision_2 [file giab024_giga-d-20-00287_revision_2.pdf]

GigaScience

Chromosome-level genome assembly of the hard-shelled mussel Mytilus coruscus, a widely distributed species from the temperate areas of East Asia

--Manuscript Draft--

**Manuscript Number:**	GIGA-D-20-00287R2
**Full Title:**	Chromosome-level genome assembly of the hard-shelled mussel Mytilus coruscus, a widely distributed species from the temperate areas of East Asia
**Article Type:**	Data Note
**Funding Information:**	the Natioanl Key Research and Development Program of China (2018YFD0900601) — Doctoral Yi-Feng Li<br>International Science and Technology Cooperation Programme (CN) (2018YFD0900101) — Professor Ying Lu<br>the National Natural Science Foundation of China (41876159) — Professor Jin-Long Yang<br>the National Natural Science Foundation of China (41606147) — Doctoral Xiao Liang<br>the National Natural Science Foundation of China (31802321) — Doctoral Yi-Feng Li<br>Guangdong Key Laboratory of Fermentation and Enzyme Engineering (CN) (GML2019ZD0402) — Professor Jin-Long Yang<br>the Shanghai Sailing Program (18YF1410000) — Doctoral Yi-Feng Li<br>Program for study on genetic resources, environment and strategy of mussel culture in coast of Gouqi Island offshore — Professor Jin-Long Yang<br>China Postdoctoral Science Foundation (2019M6614770) — Doctoral Dan-Dan Feng
**Abstract:**	Background: The hard-shelled mussel (Mytilus coruscus) is widely distributed in the temperate seas of East Asia, and is an important commercial bivalve in China. Chromosome-level genome information of this species will not only contribute to the development of hard-shelled mussel genetic breeding, but also to studies on larval ecology, climate change biology, marine biology, aquaculture, biofouling, and antifouling. Findings: We applied a combination of Illumina sequencing, Oxford Nanopore Technologies sequencing, and high-throughput chromosome conformation capture technologies to construct a chromosome-level genome of the hard-shelled mussel, with a total length of 1.57 Gb and a median contig length of 1.49 Mb. Approximately 90.9% of the assemblies were anchored to 14 linkage groups. Comparison to the Core Eukaryotic Genes Mapping Approach (CEGMA) metazoan complement revealed that the genome possesses 91.9% of core metazoan orthologs. Gene modeling enabled the annotation of 37,478 protein-coding genes and 26,917 non-coding RNA loci. Phylogenetic analysis showed that M . coruscus is the sister taxon to the clade including Modiolus philippinarum and Bathymodiolus platifrons . Conserved chromosome synteny was observed between hard-shelled mussel and king scallop, suggesting that this is shared ancestrally. Transcriptomic profiling indicated that the pathways of catecholamine biosynthesis and adrenergic signaling in cardiomyocytes might be involved in metamorphosis. Conclusions: The chromosome-level assembly of the hard-shelled mussel genome will provide novel insights into mussel genome evolution and serve as a fundamental platform for studies regarding the planktonic-sessile transition, genetic diversity, and genomic breeding of this bivalve.
**Corresponding Author:**	Ying Lu<br>Shanghai Ocean University<br>Shanghai, CHINA

Powered by Editorial Manager® and ProduXion Manager® from Aries Systems Corporation

**Corresponding Author Secondary Information:**	
**Corresponding Author's Institution:**	Shanghai Ocean University
**Corresponding Author's Secondary Institution:**	
**First Author:**	Jin-Long Yang
**First Author Secondary Information:**	
**Order of Authors:**	Jin-Long Yang
	Dan-Dan Feng
	Jie Liu
	Jia-Kang Xu
	Ke Chen
	Yi-Feng Li
	You-Ting Zhu
	Xiao Liang
	Ying Lu
**Order of Authors Secondary Information:**	
**Response to Reviewers:**	GIGA-D-20-00287R2.<br><br>Dear Dr. Hans Zauner<br>Giga Science<br><br>Thank you so much for your time and valuable help in improving the manuscript. Please find below our detailed responses addressing the comments (answers in blue).<br><br>Reviewer Comments:<br>Reviewer #2:<br>The response by Yang et al has mostly addressed my concerns. However, I have a few remaining points to address, which should be corrected before acceptance.<br>Major points to address:<br>1) the main text tables have not been provided, and I am unable to assess their contents. I do not need to see these again provided the editor can confirm their veracity.<br>in answer to my major point 1) regarding N content as I cannot see if this information is provided in the table, please ensure that it is noted either in the main text or in the table legend that gaps are preset at 100Ns, and may be longer or shorter. It may be useful to give a "total number of gaps" figure in this table.<br>Yes, total numbers of gaps are listed in Table 2 of the revision, which describes that each gap is preset at 100 Ns (Line 675-676).<br><br>2) for Figure 5 it is still confusing why M. coruscus is not compared to all the other genomes, rather than doing these comparisons with another species (which has been studied previously). Would you like to show more M. coruscus results?<br>Thanks for your suggestion. We construct the synteny between M. coruscus and four reported chromosome-level genomes in the revision (Fig. 5). In the main text (Fig.5), P. maximus is selected as a reference to illustrate gene collinearity (P. maximus vs another 4 species, including M. coruscus) due to its slow evolution. The synteny between the hard-shelled mussel and another three species (Pacific oyster, blood clam, pearl oyster) is added in the Fig. 5g-h of the revision (mentioned in main text Line 309-312, Line 475-478) as the follows:<br>In addition, the high gene collinearity between the hard-shelled mussel and another three bivalves of the Pacific oyster, blood clam and pearl oyster also reflected the satisfied quality of the hard-shelled mussel assemblies (Fig. 5f-h).<br><br>Figure 5. Chromosome synteny. a. Alignment of king scallop and blood clam

Powered by Editorial Manager® and ProduXion Manager® from Aries Systems Corporation

chromosomes. b. Alignment of king scallop and hard-shelled mussel chromosomes. c. Alignment of king scallop and pearl oyster chromosomes. d. Alignment of king scallop and Pacific oyster chromosomes. e. Rearrangements between the chromosomes of king scallop and those of four other bivalve species. The king scallop chromosomes are represented by bars of different colors, and synteny and rearrangements in the chromosomes of the four other bivalves are indicated by different blocks, whose colors correspond to those of the reference king scallop chromosomes, the dashed lines indicate the corresponding evolutionary relationship. f. Alignment of hard-shelled mussel and blood clam chromosomes. g. Alignment of hard-shelled mussel and pearl oyster chromosomes. h. Alignment of hard-shelled mussel and Pacific oyster chromosomes. The king scallop linkage groups are labeled as PM 1 to 19, the blood clam chromosomes as SB 1 to 19, the hard-shelled mussel chromosomes as MC 1 to 14, the pearl oyster chromosomes as PF 1 to 14, and the Pacific oyster chromosomes as CG 1 to 10. Scale unit, Mb. a–d, f-h. The circularized blocks represent the chromosomes of the five bivalves. Aligned homologous genes are connected by ribbons, shown in different colors depending on their chromosome location.

3) in Fig 6b, please indicate in the figure legend whether these results are the averages of your 3 replicate samples, or individual samples
Thanks for your suggestion. We describe the information in the legend of the Fig. 6b that these quantification results of gene expression are the averages of three replicate samples. (Line 495-496).

Minor points:

The manuscript would still benefit from another round of proof reading. I have noted some problems below.
Thanks for your suggestion, we have performed another round of proof reading.

Abstract:

-Linkage misspelled, "14 linage groups"
Sorry for the typo. The misspelling is corrected in the revision (Line 30).

-carried is the wrong word, and the past tense is incorrect here: "carried 91.9% of core metazoan orthologs." Perhaps "possesses 91.9% of the core metazoan ortholog set"
Sorry for the confusion. The sentence is corrected as "the genome possesses 91.9% of the core metazoan ortholog set" (Line 32).

- "the sister taxon" not "a sister taxon" (also in methods section)
Sorry for the mistake. We replace "a sister taxon" by "the sister taxon" in both abstract and method sections (Line 34 and Line 247).

Introduction:
- "due to their high economic" not "due to the high"
Sorry. We have corrected it in the revision. (Line 48).

-"understanding of the larvae-juvenile" not "understanding of larvae-juvenile"
Sorry for the mistake. We correct it in the revision (Line 64).

-"widespread among metazoans" not "widespread among metazoan "
Sorry for the mistake. We correct it in the revision (Line 68).

-"Most studies" not "Most of studies"
Sorry for the mistake. "of " is removed from that phrase (Line 74).

-"hard-shelled mussel genetic breeding" not "the hard-shelled mussel genetic breeding" (no the)
Sorry for the mistake. "the " is removed from that sentence in the revision (Line 80).

- I suggest "molluscs" rather than "mollusks" in the last paragraph, to match your usage elsewhere
Thanks for your suggestion, "mollusks" is replaced by "molluscs" (Line 88 and Line 375).

Powered by Editorial Manager® and ProduXion Manager® from Aries Systems Corporation

	Methods:<br>- no "a"s in "at a 57× coverage and the heterozygous peak was at a 28×" - this should read "at 57× coverage and the heterozygous peak was at 28×"<br>Sorry for the mistake. "a" is removed from "at a 57× coverage and the heterozygous peak was at a 28×" in the revision (Line 152-153).<br><br>-"usually occurs in fragmented assemblies" not "usually occurs in fragmented assembly",<br>Sorry for the mistake. We correct it in the revision (Line 158).<br><br>-"align to one or more of the InterPro " not "have the alignment to one or more of the InterPro"<br>Thanks. The sentence is corrected as "35,471 protein-coding genes (94.6% of the 37,478 gene models) align to one or more of the InterPro " (Line 218).<br><br>-"Chromosome-level genomes allow re-sequencing and population genetic studies." not "Chromosome-level genome is important for re-sequencing and population genetic."<br>As your suggestions, the sentence is corrected as "Chromosome-level genomes allow re-sequencing and population genetic studies" (Line 251).<br><br>-"preliminary assay" not "preliminary try "<br>Thanks for your suggestions. We use “assay", instead (Line 252).<br><br>-"Benchmark Universal Single-Copy Orthologs (BUSCO) v4.1.4" not "Benchmark Universal Single-Copy Orthologs BUSCO v4.1.4"<br>As your suggestion, "Benchmark Universal Single-Copy Orthologs BUSCO v4.1.4" is replaced by "Benchmark Universal Single-Copy Orthologs (BUSCO) v4.1.4" (Line 354).<br><br>Abbreviations"<br>no "the", TPM: Transcripts per Million;<br>As your suggestion, "the" is removed (Line 388).<br><br>Figure 3 legend (and possibly elsewhere) please italicise the k in kmer<br>As your suggestion, we italicize the k in k-mer (Line 148-153, Line 587, Line 593).<br><br>Figure 4 legend: gene names need italics<br>Thanks for your suggestion. We italicize the gene names as chitobiase (Line 460-461).<br><br>Fig 5 legend: "corresponding evolutionary relationship" not "corresponding evolution relationship"<br>Thanks for your suggestion. We correct it in the revision (Line 475).<br><br>-some bold font needed, Additional Files title page (pg 25)<br>Thanks for your suggestion. Bold font is used in the revision (Line 513, Line 515).
**Additional Information:**	
**Question**	**Response**
Are you submitting this manuscript to a special series or article collection?	No
**Experimental design and statistics**<br><br>Full details of the experimental design and statistical methods used should be given in the Methods section, as detailed in our Minimum Standards Reporting Checklist. Information essential to interpreting the	Yes

data presented should be made available in the figure legends.<br><br>Have you included all the information requested in your manuscript?	
**Resources**<br><br>A description of all resources used, including antibodies, cell lines, animals and software tools, with enough information to allow them to be uniquely identified, should be included in the Methods section. Authors are strongly encouraged to cite Research Resource Identifiers (RRIDs) for antibodies, model organisms and tools, where possible.<br><br>Have you included the information requested as detailed in our Minimum Standards Reporting Checklist?	Yes
**Availability of data and materials**<br><br>All datasets and code on which the conclusions of the paper rely must be either included in your submission or deposited in publicly available repositories (where available and ethically appropriate), referencing such data using a unique identifier in the references and in the "Availability of Data and Materials" section of your manuscript.<br><br>Have you have met the above requirement as detailed in our Minimum Standards Reporting Checklist?	Yes

Manuscript
Click here to access/download;Manuscript;GIGA-D-20-00287_forProduction.docx

Chromosome-level genome assembly of the hard-shelled mussel *Mytilus coruscus*, a widely distributed species from the temperate areas of East Asia

Jin-Long Yang[1,2,3,†,*], Dan-Dan Feng[1,2, †], Jie Liu[1,2, †], Jia-Kang Xu[1,2], Ke Chen[1,2], Yi-Feng Li[1,2], You-Ting Zhu[1,2], Xiao Liang[1,2], Ying Lu[1,2, ORCID:] 0000-0002-2509-9209[*]

[1] International Research Center for Marine Biosciences, Ministry of Science and Technology, Shanghai Ocean University, Shanghai, China

[2] Key Laboratory of Exploration and Utilization of Aquatic Genetic Resources, Ministry of Education, Shanghai Ocean University, Shanghai, China

[3] Southern Marine Science and Engineering Guangdong Laboratory, Guangzhou, China

[†] These authors contributed equally: Jin-Long Yang, Dan-Dan Feng, Jie Liu.

* Corresponding author. E-mail: jlyang@shou.edu.cn, yinglu@shou.edu.cn

Tel: + 86-21-61900403; Fax: + 86-21-61900405

Abstract

Background: The hard-shelled mussel (*Mytilus coruscus*) is widely distributed in the temperate seas of East Asia, and is an important commercial bivalve in China. Chromosome-level genome information of this species will not only contribute to the development of hard-shelled mussel genetic breeding, but also to studies on larval ecology, climate change biology, marine biology, aquaculture, biofouling, and antifouling. **Findings:** We applied a combination of Illumina sequencing, Oxford Nanopore Technologies sequencing, and high-throughput chromosome conformation capture technologies to construct a chromosome-level genome of the hard-shelled mussel, with a total length of 1.57 Gb and a median contig length of 1.49 Mb. Approximately 90.9% of the assemblies were anchored to 14 linkage groups. We assayed the genome completeness using Benchmark Universal Single-Copy Orthologs (BUSCO). In the metazoan dataset, the current assemblies have 89.4% complete, 1.9% incomplete and 8.7% missing BUSCOs. Gene modeling enabled the annotation of 37,478 protein-coding genes and 26,917 non-coding RNA loci. Phylogenetic analysis showed that *M. coruscus* is the sister taxon to the clade including *Modiolus philippinarum* and *Bathymodiolus platifrons*. Conserved chromosome synteny was observed between hard-shelled mussel and king scallop, suggesting that this is shared ancestrally. Transcriptomic profiling indicated that the pathways of catecholamine biosynthesis and adrenergic signaling in cardiomyocytes might be involved in metamorphosis. **Conclusions:** The chromosome-level assembly of the hard-shelled mussel genome will provide novel insights into mussel genome evolution and serve as

a fundamental platform for studies regarding the planktonic-sessile transition, genetic diversity, and genomic breeding of this bivalve.

Keywords: *Mytilus coruscus*, genome sequencing, Hi-C, chromosome, metamorphosis

Context

Marine mussels, which belong to the phylum Mollusca, settle on most immersed surfaces of substrata and play a crucial role in marine ecosystems. As healthy and sustainable food items, these mussels are beneficial for humans due to their high economic value for fishery and aquaculture, constituting more than 8% of mollusc aquaculture production [1]. Simultaneously, mussels are also known as typical macrofouling organisms that result in detrimental economic and ecological consequences for the maritime and aquaculture industries [2-4]. Mussels have been used as model organisms for adaptation to climate change, biomonitoring, integrative ecomechanics, biomaterials, larval ecology, settlement and metamorphosis, adhesion, bacteria-host interaction, biofouling and antifouling studies [5-12]. Although they are significant for biology, ecology and the economy, whole genome information of marine mussels is limited [13, 14] and this lack of knowledge postpones our understanding the molecular basis of adaption, evolution, breeding, genetic manipulation, bacteria-host interaction, and settlement mechanism.

As many other marine invertebrates, marine mussels also possess a free-swimming larval phase. After this stage, these minute larvae will settle on the substrata and finish metamorphosis transition, accompanied with dramatic remodeling of their anatomy [4,

15]. Multiple physicochemical stimuli play critical roles in the process of larval settlement and metamorphosis [15-17]. Thus, understanding of the larvae-juvenile transition process is still a keystone question in marine biology, larval ecology, aquaculture, biofouling and antifouling [4, 15, 18, 19]. The finding that chemical cues from bacterial biofilms trigger settlement and metamorphosis is widespread among metazoans [15, 16, 18].

The hard-shelled mussel (*Mytilus coruscus* Gould 1861, NCBI Taxonomy ID: 42192**, Fig. 1**) mainly inhabits temperate areas along the coastal waters of China, Japan, Korea and Far East of Russia, covering from East China Sea to Sea of Japan [20]. In China, the hard-shelled mussel is an important commercial bivalve as well as a typical macrofouling organism. As a sessile marine bivalve, the hard-shelled mussel needs to adapt to the hostile and complex environments of intertidal regions. Most studies focused on the planktonic-sessile transition mechanism of receptor and biofilm regulation, host-bacteria interaction, aquaculture and biofouling and antifouling studies in this species [3-5, 12, 21-23]. To date, no genome of any member of the genus *Mytilus* has been assembled at the chromosome level, although a draft genome of *M. coruscus* [24] and an improved genome of *M. galloprovincialis* [13, 25] have been reported. The lack of whole-genome information has hindered the development of hard-shelled mussel genetic breeding, larval ecology, climate change biology, marine biology, aquaculture, biofouling and antifouling studies.

In this study, we report a chromosome-level assembly of the hard-shelled mussel genome obtained by combining Illumina sequencing, Oxford Nanopore Technologies

(ONT) sequencing, and high-throughput chromosome conformation capture (Hi-C) technologies. We validated the genome assemblies by chromosome synteny analysis, comparing them with the published chromosome-level genomes of the most studied molluscs. Larvae at five early developmental stages were subjected to RNA sequencing (RNA-seq) analysis for the profiling of gene expression during metamorphosis. Accessible chromosome-level genome datasets [26, 27] will facilitate comparative genomics studies on chromosome rearrangements across different species.

Methods

Sample information and collection

Wild individuals for genome sequencing were collected from the coast of Shengsi, Zhejiang province, which is the central coast of the Chinese mainland, and one of the original and main breeding areas of the hard-shelled mussel in China. Farmed and wild adults were also collected from the coast of Shengsi (122.77E 30.73N and 122.74E 30.71N, respectively) (**Fig. 1**). A female wild adult with a mature ovary was dissected, and the adductor muscle was collected to isolate high-molecular-weight genomic DNA for the sequencing of the reference genome. The DNA extracted from the farmed and wild populations (10 individuals per population) was pooled for genome re-sequencing. Adductor muscle, mantle, gill, digestive gland, hemocyte, labial palp, female gonad, male gonad, foot, and gut tissues were dissected from fresh samples for transcriptome sequencing to assist with the prediction of protein-coding genes.

Isolation of genomic DNA and RNA

Genomic DNA was extracted from fresh adductor muscle tissue using the SDS extraction method [28], and then used for sequencing on an ONT PromethION platform (Oxford Nanopore Technologies, UK). Using the TIANamp Marine Animals DNA kit (Tiangen, China), DNA for whole genome re-sequencing was extracted from the muscles of five female and five male individuals from each population. Using the RNAiso Plus kit (TaKaRa, Japan), total RNA was extracted from 10 different tissues of five female and five male individuals from each population to obtain a large gene expression dataset. Fresh muscle cells were crosslinked with formaldehyde, and digestion, marking of DNA ends, and blunt-end ligation were performed as described in a previous study [29]. The purified DNA was used for Hi-C.

Genome sequencing with different technologies

A combined sequencing strategy was applied to obtain the hard-shelled mussel genome (**Fig. 2**). Qualified DNA was filtered using a BluePippin^{TM} System to extract large fragments. The large-fragment DNA was employed to construct a library using the ONT Template prep kit and the NEB Next FFPE DNA Repair Mix kit [New England Biolabs (NEB), USA]. A high-quality library with an average length of 20 kb was sequenced on the ONT PromethION platform with the corresponding R9 cell and ONT sequencing reagent kit. A total of 246.8 Gb of data (~159× coverage) were generated (**Table 1**).

Sequencing of Hi-C and genome survey libraries was performed on an Illumina sequencing platform. Briefly, the extracted DNA was fragmented to a size of 300–350

bp using an E210 Focused Ultrasonicator (Covaris, USA). The construction of paired-end libraries encompassed the successive steps of end repair, poly(A) addition, barcode indexing, purification, and PCR amplification. The libraries were sequenced with the Illumina NovaSeq 6000 platform (Illumina, USA) to generate 150-bp paired-end reads. Sequencing of the Hi-C libraries generated a total of 249.6 Gb of data (~161× coverage), and sequencing of the genome survey libraries generated a total of 160.6 Gb of data (~104× coverage).

The qualified RNA extracted from the same tissues of 10 individuals was equally mixed for RNA-seq. The sample was enriched in mRNA by extracting poly(A) transcripts from total RNA using oligo-d(T) magnetic beads. Sequencing libraries were prepared using the NEBNext® Ultra™ RNA Library Prep Kit for Illumina® (NEB, USA) following the manufacturer's recommendations. A total of 10 libraries were sequenced on the Illumina NovaSeq 6000 platform in a 150-bp paired-end mode.

The raw reads from Illumina sequencing platform were cleaned using FastQC45 and HTQC46 by the following steps: (a) filtered reads with adapter sequence; (b) filtered PE reads with one reads more than 10% N bases; (c) filtered PE reads with any end has more than 50% inferior quality (≤5) bases.

Genome survey and contig assembly

The size of the hard-shelled mussel genome was estimated using the *k*-mer-based method implemented in Jellyfish (version 2.3.0) with values of 51-mers [30] and GenomeScope (10,000× cut-off) [31]. The *k*-mers refer to all the *k*-mer frequency

distributions from a read obtained through Illumina DNA sequencing. The homozygous peak of the assembly was at 57× coverage and the heterozygous peak was at 28× coverage (**Fig. 3a**). The assessment of genome size by *k*-mer counting suggested a complete genome size of approximately 1.51 Gb (**Fig. 3a**), which is close to the final assembly (1.57 Gb) and cytogenetic estimates [32]. Sequence alignment between the previous assembly (1.90 Gb) [24] and the one in this study revealed considerable heterozygous redundancies in the former. This kind of overestimation of genome size usually occurs in fragmented assemblies, like the recently published *M. galloprovincialis* genome [25].

Genome assembly from long-read data was carried out following three methods. First, long reads were *de novo* assembled using the Canu (Canu, RRID:SCR_015880) v1.5 software with default parameters [33]; next, error correction was performed with Racon v1.3.1 [34]. Then, further polishing with Illumina short-read data was conducted using Pilon (Pilon, RRID:SCR_014731) v1.22 [35]. The final assembly was approximately 1.57 Gb in size, consisting of 6,449 contigs with an overall median length (N50) of 1.49 Mb, while the previously published draft genome only had an N50 of 0.66 Mb [24]. The present genome had a heterozygous rate of 1.39 % (also calculated by GenomeScope) and an average GC content of approximately 32%.

Anchoring of the contigs to pseudo-moleculars with Hi-C data

To complete the assembly of the hard-shelled mussel genome, Hi-C technology was carried out to generate information on the interactions among contigs. DNA from fresh

adductor muscle tissue was used to prepare a Hi-C library. This was then sequenced on the Illumina NovaSeq 6000 platform, producing 249.6 Gb of reads (**Table 1**). These reads were aligned to the assembled contigs using BWA (BWA, RRID:SCR_010910) aligner v0.7.10-r789 [36]. Lachesis v2e27abb was applied to anchor the contigs onto the linkage groups using the agglomerative hierarchical clustering method [37]. Finally, 2,029 contigs representing 90.9% of the total assemblies were successfully anchored to 14 chromosomes (**Table 2**); this number was consistent with the outputs of the karyotype [38]. The unclosed gaps only occupies 0.014% of the assembly (201,500 bp), which is filled with Ns (**Table 2**). The N50 of the anchored contigs was over 1.7 Mb, around 1.14 times of the initial assemblies from the ONT long reads.

Genome annotation

A *de novo* repeat annotation of the hard-shelled mussel genome was carried out using RepeatModeler (RepeatModeler, RRID:SCR_015027) version 1.0.11 [39] and RepeatMasker (RepeatMasker, RRID:SCR_012954) version 4.0.7 [40]. RepeatModeler was used to construct the repeat library, which was then examined using two other programs, RECON and RepeatScout (RepeatScout, RRID:SCR_014653). The yielded consensus sequences were manually checked by aligning to the genes from the GenBank database (nt and nr; released in October 2019) to avoid that sequences of the high-copy genes are masked in following process with RepeatMasker. The final repeat library consisted of 2,264 consensus sequences with the respective classification information, which was used to run RepeatMasker against the genome assemblies. The

repetitive sequences constituted a length of 735.6 Mb, representing 47.4% of the total genome length (**Supplementary Table S1**). Simple sequence repeats (SSRs) were identified using Tandem Repeats Finder V 4.04. Only monomers, dimers, trimers, tetramers, pentamers, and hexamers with at least four repeat units were considered. The total length of the 5,324 identified SSRs was approximately 138.0 kb.

Conserved non-coding RNAs were predicted using the Rfam 11.0 databases. Putative microRNAs (miRNAs) and ribosomal RNAs (rRNAs) were predicted using Infernal (Infernal, RRID:SCR_011809) version 1.1.2 [41], and transfer RNAs (tRNAs) were predicted with tRNAscan-SE (tRNAscan-SE, RRID:SCR_010835) v2.0.3. A total of 9,186 miRNAs, 342 rRNAs, and 1,881 tRNAs were detected (**Supplementary Table S2**).

Protein-coding genes were predicted using a combined strategy of *ab initio* prediction, homology-based prediction, and transcriptome-based prediction (**Fig. 2**). The *ab initio* prediction was conducted using the Augustus (Augustus: Gene Prediction, RRID:SCR_008417) version 3.1 [38], GlimmerHMM (GlimmerHMM, RRID:SCR_002654) version 1.2 [39], and SNAP (version 2006-07-28) software [42]. For homology-based prediction, protein sequences of two closely related mollusk species (*Modiolus philippinarum* and *Bathymodiolus platifrons*), downloaded from GenBank, were aligned to the genome assemblies using Exonerate (version 2.2.0) [43]. In parallel, transcriptomic data from 10 tissues (GenBank SRA accession ID: PRJNA578350) were assembled *de novo* using Trinity (Trinity, RRID:SCR_013048) version 2.4.0 [44] and Cufflinks (Cufflinks, RRID:SCR_014597) version 2.2.1 [45].

The outputs of both assemblers were integrated using the Program to Assemble Spliced Alignments (PASA, version 2.3.3) [46]. After merging of all of these predictions using EVidenceModeler (v1.1.0) [46], a total of 37,478 final gene models were generated (**Table 3)**, a number lower than that of the previously published 42,684 gene models in the draft genome [24]. Functional annotations displayed that 35,471 protein-coding genes (94.6% of the 37,478 gene models) align to one or more of the InterPro (version 5.22-61.0) [47], GO [48], KEGG [49], Swissprot [50] and NCBI non-redundant protein (NR) functional databases (**Table 4**; **Fig. 3b**). This information is illustrated in a genome landscape map (**Fig. 3c**). Using a bidirectional BLASTp between the two assemblies, we observed that a considerable heterozygous redundancies (over 20%) were probably included into the previous draft assemblies (**Supplementary Table S3**), which might be owing to the widespread hemizygosity and massive gene presence/absence variation (PAV) [25, 51] or assembling errors.

Phylogenetic analysis

Gene clusters were identified among 12 selected genomes, namely those of *Chlamys farreri* (PRJNA185465), *Pinctada fucata martensii* (GCA_002216045.1), *M. philippinarum* (GCA_002080025.1), *Crassostrea gigas* (GCF_000297895.1), *B. platifrons* (GCA_002080005.1), *Mizuhopecten yessoensis (GCA_002113885.2)*, *Penaeus vannamei* (ASM378908v1), *Pecten maximus* (GCA 902652985.1), *Scapharca* (*Anadara*) *broughtonii* (PRJNA521075), *Pomacea canaliculata* **(**PRJNA427478), *Haliotis discus hannai* (PRJNA317403), and *M. coruscus*, using OrthoMCL

(OrthoMCL DB: Ortholog Groups of Protein Sequences, RRID:SCR_007839) version 1.4 with a BLASTp cut-off value of 10^{-5} and an inflation value of 1.5 [52]. A total of 448 single-copy genes identified by OrthoDB were aligned and concatenated. The amino acid sequences were first aligned using MUSCLE (MUSCLE, RRID:SCR_011812) [53], and then further concatenated to create one supergene sequence for each species and form a data matrix. The phylogenetic relationships among different supergenes were then assessed using a maximum-likelihood model in RAxML (RAxML, RRID:SCR_006086) version 8 [54] with the optimal substitution model of PROTGAMMAJTT. The robustness of the maximum-likelihood tree was assessed using the bootstrap method (100 pseudo-replicates). Furthermore, single-copy orthologs and one reference divergence time on the root node obtained from the TimeTree database [55] were used to calibrate the divergence dates of other nodes on this phylogenetic tree using the $MCMC_{TREE}$ tool in the PAML (PAML, RRID:SCR_014932) package [56]. Visualization of phylogenetic relationships with FigTree (version 1.4.3) [57] suggested that *M. coruscus* is the sister taxon to the clade containing *M. philippinarum* and *B. platifrons*, with a divergence time of approximately 129 Mya (**Fig. 3d**).

Whole genome re-sequencing of farmed and wild individuals

Chromosome-level genomes allow re-sequencing and population genetic studies. We performed a preliminary assay to detect sequence variation by sequencing two genomic DNA pools of wild population and farmed population. A total of 50.4 Gb and 46.7 Gb

of Illumina clean reads were finally generated in farmed and wild samples, respectively. Over 89% reads were aligned to the reference genome with BWA (v0.7.10-r789) [36]. The PCR duplicates (duplicates introduced by PCR) were removed with MarkDuplicates in the Picard (Picard, RRID:SCR_006525) toolkit [58]. SNPs and small indels (10 bp or less) were identified with GATK (GATK , RRID:SCR_001876) version 3.7 [59] with default parameters and the addition of three extra thresholds to discard unreliable items during post-filter analysis, namely: 1) any two SNPs located within 5 bp from each other; 2) any two indels located within 10 bp from each other; and 3) any SNPs located within 5 bp from an indel. Finally, we identified 5,733,780 SNPs and 1,821,690 small indels in the farmed population and 5,719,771 SNPs and 1,820,404 small indels in the wild population. Similar distribution patterns of SNPs and indels were detected between the farmed and wild population (**Supplementary Fig. S1**) when nearly 99% of the SNPs/indels were shared by both populations (**Fig. 4a**), reflecting that only approximately 1% of the sequence variations were farmed population specific (FPS) or wild population specific (WPS). We focused on the differential variations located in the flanking regions and genic regions, between the farmed and wild populations, to identify candidate genes and causal mutations related to morphological traits. The software SnpEff version 2.0.5 [60] was applied to detect the effect of SNPs/indels by comparing the loci of SNPs/indels with those of protein-coding genes, which revealed that 59 genes carrying FPS SNPs/indels (FPSGs) and 57 genes carrying WPS SNPs/indels (WPSGs) underwent loss of translational start sites, gain or loss of stop codons, or variants in the acceptor/donor of splicing sites. Some

variations were observed to cluster in farmed population (**Fig. 4b**), implicating a potentially influence to morphological diversity. In addition, PAV might play a role in determining phenotypic traits [25, 51], which should be included in the future re-sequencing analyses.

Chromosome synteny and evolution in bivalves

To investigate the evolution of the mussel chromosomes, gene collinearity was constructed by aligning the genes of the king scallop *P. maximus* to the reference genomes of the blood clam *S. broughtonii*, the hard-shelled mussel *M. coruscus*, the pearl oyster *P. martensii*, and the Pacific oyster *C. gigas* using MCscan (version 0.8). The parameters of the MCscan alignment were set as -s, 7; k, 150; m, 250; e, $1e^{-10}$. We identified 404 scallop-vs-clam, 276 scallop-vs-mussel, 159 scallop-vs-pearl-oyster, and 232 scallop-vs-pacific-oyster syntenic blocks, which included 10,055, 4,716, 3,636, and 5,009 genes of blood clam, hard-shelled mussel, pearl oyster and Pacific oyster, respectively. The mean gene number per syntenic block was 21.4. King scallop and blood clam had the highest gene collinearity (**Fig. 5a**), consistent with their close phylogenetic relationship in the Bivalvia clade [61] (**Fig. 3d**). The chromosome synteny illustrated that large-scale rearrangements are rare between scallop and mussel, but frequent between scallop and oysters (Fig. **5b–d**), as exemplified by considerable structural variations between the scallop and the Pacific oyster genomes (**Fig. 5d**). The identified cross-chromosome rearrangements between the scallop and mussel genomes were different from those between the genomes of scallop and the two oyster species

(**Fig. 5b–e**). The scallop linkage groups (PM) 1, 5, 6, 8, 10, 16, 17, 18, and 19 were syntenic to a single mussel chromosome (MC) 8, 9, 3, 4, 10, 13, 11, 12, and 14, respectively. PM 2 and 15 were aligned to the same reference, MC 2; similarly, PM 3 and 14 aligned to MC 5, PM 4 and 7 aligned to MC 1, PM 9 and 12 aligned to MC 7, and PM 11 and 13 aligned to MC 6. Comparatively, some additional chromosome rearrangements occurred between scallop and the two oyster species, especially the Pacific oyster. Both the Pacific oyster chromosome 9 and the pearl oyster chromosome 7 were predominantly syntenic to the scallop PM 15, suggesting that they might carry conserved genomic regions with the same origin (**Fig. 5c–e**). Among all the syntenic chromosomes, we did not observe any chromosome to be entirely conserved in all of the bivalve genomes. Intriguingly, almost all of the chromosome rearrangements between the mussel and the oyster genomes were different (**Fig. 5e**), implicating independent chromosome fusion events. In addition, the high gene collinearity between the hard-shelled mussel and another three bivalves of the Pacific oyster, blood clam and pearl oyster also reflected the satisfied quality of the hard-shelled mussel assemblies (Fig. **5f-h**). The identification of such diverse chromosome rearrangements suggested a complex evolutionary history of bivalve chromosomes.

Metamorphosis-related transcriptome analysis

To profile gene expression during development and metamorphosis in hard-shelled mussels, RNA-seq analysis was conducted at five developmental stages: trochophore, D-veliger, umbo, pediveliger, and juvenile (PRJNA689932). The

quantification of gene expression enabled the detection of 33,743 transcripts with the TPM > 0 at all stages (**Supplementary Table S4**). The limma statistical method was used to detect DEGs based on linear models [62]. Using the trochophore as control, 5,795; 6,163; 9,308; and 7,486 upregulated genes [$\log_2$(fold-change) > 1 and adjusted P < 0.05] were identified in D-veliger, umbo, pediveliger, and juvenile larvae, respectively. Functional annotation indicated that these were mainly involved in "environmental information processing" ("signal transduction" and "signaling molecules and interaction") and "cellular processes" ("transport and catabolism"), in agreement with the key role of signal transduction and the endocrine system in larval development [17].

Since the ability to effectuate metamorphosis develops during the pediveliger stage [17], we investigate the 774 up-regulated genes during the transition from the umbo to the pediveliger stage. Functional annotation revealed that they were mainly employed in a network of six related pathways: "adrenergic signaling in cardiomyocytes," "calcium signaling pathway," "MAPK signaling pathway," "protein export," "endocytosis," and "catecholamine biosynthesis" (**Fig. 6a**), which have been reported to be involved in settlement and metamorphosis [18, 63]. The expression of most of the genes involved in these pathways increased during one or more periods (**Fig. 6b**). Among them, 20 genes have been functionally identified to be associated with metamorphosis (**Supplementary Table S5**) and 26 up-regulated encompassing from the umbo to the pediveliger stages belonged to the categories "adrenergic signaling in cardiomyocytes," "calcium signaling pathway," and "catecholamine

transport”, which was consistent with the findings of a recent proteome study on larval settlement and the metamorphosis of oysters [63-66]. Although some additional pathways, such as “phagosome” and “oxytocin signaling pathway”, are also detected, we did not analyze them in detail because still lacking evidence on their involvement in metamorphosis. In summary, the analysis of the involved pathways revealed that biosynthesis, transport, and transduction of catecholamines might be critical for the completion of metamorphosis.

Assembly assessment

The quality of the assembled genome was validated in terms of completeness, accuracy of the assemblies, and conservation of synteny. Alignment of Illumina reads against the reference genome revealed insert sizes of paired-end sequencing libraries of approximately 300–350 bp and a mapping rate of over 96.7%. We assayed the genome completeness using Benchmark Universal Single-Copy Orthologs (BUSCO (BUSCO, RRID:SCR_015008)) v4.1.4 referencing metazoan and molluscan gene sets. In the metazoan dataset, the current assemblies have 89.4% complete (of which 1.0% were duplicated), 1.9% incomplete and 8.7% missing BUSCOs, corresponding to a recovery of 91.3% of the entire BUSCO set. In the molluscan dataset, 85.5% complete (of which 1.3% were duplicated), 0.8% incomplete and 13.7% missing BUSCOs were recorded, corresponding to 86.3% of the entire BUSCO set. Motifs with the characteristics of telomeric repeats were detected in 23 termini of the 13 chromosomes, suggesting the completeness of the assemblies (**Supplementary Table S6**). The accuracy of the

genome assembly was evaluated by calling sequence variants through the alignment of Illumina sequencing data against the genome. Sequence alignment with the BCFtools (version 1.3) [67] revealed 368,991 homozygous SNP loci, reflecting an error rate of less than 0.02% in the genome assemblies. In addition, the highly conserved synteny and the strict correspondence of chromosome fusion points and gene assignment identified between the hard-shelled mussel and king scallop genomes (**Fig. 5b**) were indicative of a high-quality assembly of the hard-shelled mussel genome, since the king scallop genome is considered as the best-scaffolded genome available for bivalves [68].

Conclusion

The chromosome-level assemblies of the hard-shelled mussel genome presented here is a well-assembled and annotated resource that would facilitate a wide range of research in mussels, bivalves, and molluscs. The outputs of this study shed light on the chromosome evolution in bivalves, resulting in the regulation of the molecular pathways involved in larval metamorphosis. As one of the chromosome-level genome assemblies of bivalves, this genome data set will serve as a high-quality genome platform for comparative genomics at the chromosome level.

Availability of Supporting Data and Materials

All of the raw Illumina and ONT reads were deposited to NCBI Sequence Read Archive and the assembled genome was deposited to GenBank, under the accession number PRJNA578350. Gene expression data in different developmental stages is released

under the accession number PRJNA689255. The corresponding genome sequences and read alignments (VCF files) are available in Figshare [69] and GigaDB [70].

Abbreviations

TPM: Transcripts per Million; GATK: Genome Analysis Tool Kit; GO: Gene Ontolog; KEGG: Kyoto Encyclopedia of Genes and Genomes; AC1: adenylate cyclase 1; AC10: adenylate cyclase 10; Akt: RAC serine/threonine-protein kinase; CaM: calmodulin; CaMKII: calcium/calmodulin-dependent protein kinase (CaM kinase) II; CAV1: caveolin 1; CAV3: caveolin 3; CREB: cyclic AMP-responsive element-binding protein; DBH: dopamine beta-monooxygenase; DDC: aromatic-L-amino-acid decarboxylase; DHPR: voltage-dependent calcium channel gamma-1; Epac: Rap guanine nucleotide exchange factor; ERK: mitogen-activated protein kinase 1/3; Gi: guanine nucleotide-binding protein G(i) subunit alpha; Gq: guanine nucleotide-binding protein G(q) subunit alpha; Gs: guanine nucleotide-binding protein G(s) subunit alpha; ICER: cAMP response element modulator; IKS: potassium voltage-gated channel KQT-like subfamily member 1; IMP2: mitochondrial inner membrane protease subunit 2; INaK: sodium/potassium-transporting ATPase subunit alpha; MAOA: monoamine oxidase A; MAOB: monoamine oxidase B; MSK1: ribosomal protein S6 kinase alpha-5; NCX : solute carrier family 8 (sodium/calcium exchanger); NF-κB: nuclear factor NF-kappa-B p105 subunit; NHE: solute carrier family 9 (sodium/hydrogen exchanger); p38MAPK: p38 MAP kinase; PI3K: phosphatidylinositol-4,5-bisphosphate 3-kinase catalytic subunit alpha/beta/delta; PKA: protein kinase A; PKCα: classical protein

kinase C alpha type; PLC: phosphatidylinositol phospholipase C; PP1: serine/threonine-protein phosphatase PP1 catalytic subunit; TnI: Troponin I; TPM: tropomyosin; TYR: tyrosinase; α-ARA: alpha-1A adrenergic receptor-like; α-ARB: adrenergic receptor alpha-1B; β2AR: adrenergic receptor beta-2.

Competing Interests

The authors declare no competing interests.

Funding

This study was supported by the National Key Research and Development Program of China (2018YFD0900601), Key Program for International Science and Technology Cooperation Projects of Ministry of Science and Technology of China (No. 2018YFD0900101), the National Natural Science Foundation of China (No. 41876159, No. 41606147, No. 31802321), Key Special Project for Introduced Talents Team of Southern Marine Science and Engineering Guangdong Laboratory (Guangzhou) (GML2019ZD0402), the Shanghai Sailing Program (18YF1410000), China Postdoctoral science foundation (2019M6614770) and Program for study on genetic resources, environment and strategy of mussel culture in coast of Gouqi Island offshore.

Authors Contributions

J.L.Y., Y.L. and X.L. designed and supervised the study. K.C., J.K.X, Y.T.Z, Y.F.L. collected the samples and extracted the genomic DNA and RNA. Y.L., J.L. and D.D.F.

performed genome assembly and bioinformatics analysis. J.L.Y., D.D.F., X.L., J.L. and Y.L. wrote the original manuscript. All authors reviewed the manuscript.

Figure legends

Figure 1. Sequenced individuals and sampling sites. **a.** Pictures of the sequenced individuals collected in Shengsi. A wild *M. coruscus* adult was used for genome sequencing. Both wild and farmed populations were used for re-sequencing. **b.** The geographic locations of the sampling sites.

Figure 2. Workflow of genome sequencing and annotation. The rectangles indicate the steps of data treatment and the diamonds indicate output or input data.

Figure 3. Annotation and evolution. **a.** GenomeScope plot of the 51-mer content within the hard-shelled mussel genome. Estimates of genome size and read data were shown. **b.** Venn diagram indicating the number of genes that were annotated in one or more databases. **c.** Genomic landscape of *M. coruscus*. The chromosomes were labeled as LG01 to LG14. From the outer to the inner circle: 5, marker distribution across 14 chromosomes at a megabase scale; 4, gene density across the whole genome; 3, SNP density; 2 and 1, number of repetitive sequences and GC content across the genome. 1–5 are drawn in non-overlapping 0.1-Mb sliding windows. The length of chromosomes is defined by the scale (Mb) on the outer circles. **d.** Phylogenetic tree based on protein sequences from 12 metazoan genomes, namely those of *Chlamys farreri* (PRJNA185465), *Pinctada fucata martensii* (GCA_002216045.1), *Modiolus philippinarum* (GCA_002080025.1), *Crassostrea gigas* (GCF_000297895.1), *Mytilus coruscus*, *Bathymodiolus platifrons* (GCA_002080005.1), *Mizuhopecten yessoensis*

(GCA_002113885.2), *Penaeus vannamei* (ASM378908v1), *Pecten maximus* (GCA 902652985.1), *Scapharca* (*Anadara*) *broughtonii* (PRJNA521075), *Pomacea canaliculata* (PRJNA427478), and *Haliotis discus hannai* (PRJNA317403).

Figure 4. Sequence variations between farmed and wild populations**. a.** Venn diagrams showing the number and distribution of indels and SNPs between the farmed and wild populations. **b.** Differences in the number of SNPs on the exons of *chitobiase*. The rectangles indicate the 14 exons of the *chitobiase* gene and the lines between the 14 rectangles indicate introns; the pink matrix represents reads from the farmed population, and the blue matrix represents reads from the wild population. Bases denoted by capital letters are located on exons, whereas those denoted by small letters are located on introns.

Figure 5. Chromosome synteny. **a.** Alignment of king scallop and blood clam chromosomes. **b.** Alignment of king scallop and hard-shelled mussel chromosomes. **c**. Alignment of king scallop and pearl oyster chromosomes. **d**. Alignment of king scallop and Pacific oyster chromosomes. **e.** Rearrangements between the chromosomes of king scallop and those of four other bivalve species. The king scallop chromosomes are represented by bars of different colors, and synteny and rearrangements in the chromosomes of the four other bivalves are indicated by different blocks, whose colors correspond to those of the reference king scallop chromosomes, the dashed lines indicate the corresponding evolutionary relationship. **f.** Alignment of hard-shelled

mussel and blood clam chromosomes. **g.** Alignment of hard-shelled mussel and pearl oyster chromosomes. **h**. Alignment of hard-shelled mussel and Pacific oyster chromosomes. The king scallop linkage groups are labeled as PM 1 to 19, the blood clam chromosomes as SB 1 to 19, the hard-shelled mussel chromosomes as MC 1 to 14, the pearl oyster chromosomes as PF 1 to 14, and the Pacific oyster chromosomes as CG 1 to 10. Scale unit, Mb. **a–d, f-h.** The circularized blocks represent the chromosomes of the five bivalves. Aligned homologous genes are connected by ribbons, shown in different colors depending on their chromosome location.

Figure 6. Spatial and temporal expression of genes involved in development and metamorphosis. **a.** Expression pattern of genes implied in the pathways of catecholamine biosynthesis and adrenergic signaling in cardiomyocytes, according to KEGG-based annotation. Red rectangles indicate upregulated genes during development， red rectangles with black edge indicate upregulated genes at Pediveliger stage and metamorphosis, and white rectangles denote genes that were identified during KEGG analysis but whose expression did not change. Red bubbles represent the most important pathways in which the upregulated genes are involved. **b.** Heatmap showing the expression levels of all genes involved in the pathways of catecholamine biosynthesis and adrenergic signaling in cardiomyocytes across five developmental stages. These quantification results of gene expression are the averages of three replicate samples.

Table captions

Table 1. Statistics of whole genome sequencing using Illumina and ONT

Table 2. Results of contig anchoring on pseudochromosomes using Hi-C data

Table 3. General statistics of the predicted protein-coding genes

Table 4. General statistics of gene functional annotation

Additional Files

Supplementary Table S1. Repetitive sequences in the hard-shelled mussel genome

Supplementary Table S2. Overview of the predicted non-coding RNAs

Supplementary Table S3. Bidirectional BLASTp between the previously published gene models of the hard-shelled mussel and the predicted gene models in this study.

Supplementary Table S4. Gene expression profiles during five developmental stages

Supplementary Table S5. Genes involved in the pathways of catecholamine biosynthesis and adrenergic signaling in the cardiomyocytes were reported to affect metamorphosis.

Supplementary Table S6. Information of the motifs with the characteristic of telomeric repeats

Supplementary Figure S1. Circles showing genome-wide SNPs and indels from the farmed and wild populations. From the outer to the inner circle: first circle, marker distribution across 14 pseudochromosomes at a megabase scale; green circle, SNP density across the whole genome; red circle, indel density.

References

1. FAO. The state of world fisheries and aquaculture. 2018.
2. Amini S, Kolle S, Petrone L, et al. Preventing mussel adhesion using lubricant-infused materials. Science 2017; **357**:668-673.
3. Yang JL, Li YF, Guo XP, et al. The effect of carbon nanotubes and titanium dioxide incorporated in PDMS on biofilm community composition and subsequent mussel plantigrade settlement. Biofouling 2016; **32**:763-777.
4. Yang JL, Shen PJ, Liang X, et al. Larval settlement and metamorphosis of the mussel *Mytilus coruscus* in response to monospecific bacterial biofilms. Biofouling 2013; **29**:247-259.
5. Liang X, Peng LH, Zhang S, et al. Polyurethane, epoxy resin and polydimethylsiloxane altered biofilm formation and mussel settlement. Chemosphere 2019; **218**:599-608.
6. Odonnell MJ, George MN, Carrington E. Mussel byssus attachment weakened by ocean acidification. Nature Climate Change 2013; **3**:587-590.
7. Ramesh K, Hu MY, Thomsen J, et al. Mussel larvae modify calcifying fluid carbonate chemistry to promote calcification. Nature Communications 2017; **8**:1709.
8. Thomsen J, Stapp L, Haynert K, et al. Naturally acidified habitat selects for ocean acidification–tolerant mussels. Science Advances 2017; **3**:e1602411.
9. Bitter MC, Kapsenberg L, Gattuso J, et al. Standing genetic variation fuels rapid adaptation to ocean acidification. Nature Communications 2019; **10**:1-10.
10. Briand J. Marine antifouling laboratory bioassays: an overview of their diversity. Biofouling 2009; **25**:297-311.
11. Petrone L, Kumar A, Sutanto CN, et al. Mussel adhesion is dictated by time-regulated secretion and molecular conformation of mussel adhesive proteins. Nature Communications 2015; **6**:8737-8737.
12. Zeng ZS, Guo XP, Cai XS, et al. Pyomelanin from *Pseudoalteromonas lipolytica* reduces biofouling. Microbial Biotechnology 2017; **10**:1718-1731.
13. Murgarella M, Puiu D, Novoa B, et al. A first insight into the genome of the filter-feeder mussel *Mytilus galloprovincialis*. PLoS One 2016; **11**:e0151561.
14. Sun J, Zhang Y, Xu T, et al. Adaptation to deep-sea chemosynthetic environments as revealed by mussel genomes. Nature Ecology and Evolution 2017; **1**:0121.
15. Hadfield MG, Paul VG. In *Marine chemical ecology* (ed. McClintock, J.B & Baker, J.B) Ch13. CRC Press, 2001.
16. Dobretsov S, Rittschof D. Love at first taste: induction of larval settlement by marine microbes. International Journal of Molecular Sciences 2020; **21**:731.
17. Hadfield MG. Biofilms and marine invertebrate larvae: what bacteria produce that larvae use to choose settlement sites. Annual Review of Marine Science 2011; **3**:453-470.
18. Shikuma NJ, Antoshechkin I, Medeiros JM, et al. Stepwise metamorphosis of the tubeworm *Hydroides elegans* is mediated by a bacterial inducer and MAPK signaling. Proceedings of the National Academy of Sciences of the United States of America 2016; **113**:10097-10102.
19. Shikuma NJ, Pilhofer M, Weiss GL, et al. Marine tubeworm metamorphosis induced by arrays of bacterial phage tail–like structures. Science 2014; **343**:529-533.

20. Kulikova VA, Lyashenko SA, Kolotukhina NK. Seasonal and interannual dynamics of larval abundance of *Mytilus coruscus* Gould, 1861 (Bivalvia: Mytilidae) in Amursky Bay (Peter the Great Bay, Sea of Japan). Russian Journal of Marine Biology 2011; **37**:342-347.
21. Li YF, Liu YZ, Chen YW, et al. Two toll-like receptors identified in the mantle of *Mytilus coruscus* are abundant in haemocytes. Fish & Shellfish Immunology 2019; **90**:134-140.
22. Liang X, Zhang XK, Peng LH, et al. The flagellar gene regulates biofilm formation and mussel larval settlement and metamorphosis. International Journal of Molecular Sciences 2020; **21**:710.
23. Yang JL, Li SH, Li YF, et al. Effects of neuroactive compounds, ions and organic solvents on larval metamorphosis of the mussel *Mytilus coruscus*. Aquaculture 2013; **396-399**:106-112.
24. Li RH, Zhang WJ, Lu JK, et al. The whole-genome sequencing and hybrid assembly of *Mytilus coruscus*. Frontiers in Genetics 2020; **11**:1-6.
25. Gerdol M, Moreira R, Cruz F, et al. Massive gene presence-absence variation shapes an open pan-genome in the Mediterranean mussel. Genome Biology 2020; **21**:275.
26. Li YL, Sun XQ, Hu XL, et al. Scallop genome reveals molecular adaptations to semi-sessile life and neurotoxins. Nature Communications 2017; **8**:1721-1721.
27. Wang S, Zhang J, Jiao W, et al. Scallop genome provides insights into evolution of bilaterian karyotype and development. Nature Ecology & Evolution 2017; **1**:0120.
28. Sokolov EP. An improved method for DNA isolation from mucopolysaccharide-rich molluscan tissues. Journal of Molluscan Studies 2000; **66**:573-575.
29. Van Berkum NL, Lieberman-Aiden E, Williams L, et al. Hi-C: A method to study the three-dimensional architecture of genomes. Journal of Visualized Experiments 2010; **39**:e1869.
30. Marçais G, Kingsford C. A fast, lock-free approach for efficient parallel counting of occurrences of *k*-mers. Bioinformatics 2011; **27**:764-770.
31. Vurture GW, Sedlazeck FJ, Nattestad M, et al. GenomeScope: fast reference-free genome profiling from short reads. Bioinformatics 2017; **33**:2202-2204.
32. Ieyama H, Kameoka O, Tan T, et al. Chromosomes and nuclear DNA contents of some species in Mytilidae. Venus (Japanese Journal of Malacology) 1994; **53**:327-331.
33. Koren S, Walenz BP, Berlin K, et al. Canu: scalable and accurate long-read assembly via adaptive *k*-mer weighting and repeat separation. Genome research 2017; **27**:722-736.
34. Vaser R, Sović I, Nagarajan N, et al. Fast and accurate de novo genome assembly from long uncorrected reads. Genome Research 2017; **27**:737-746.
35. Walker BJ, Abeel T, Shea T, et al. Pilon: an integrated tool for comprehensive microbial variant detection and genome assembly improvement. PloS One 2014; **9**:e112963.
36. Li H, Durbin R. Fast and accurate short read alignment with Burrows–Wheeler transform. Bioinformatics 2009; **25**:1754-1760.
37. Burton JN, Adey A, Patwardhan RP, et al. Chromosome-scale scaffolding of de novo genome assemblies based on chromatin interactions. Nature Biotechnology 2013; **31**:1119-1125.
38. Zhuang BX. A preliminary study on the chromosome of marine bivalve, *Mytilus coruscus*. Zoological Research 1984; **S2**.
39. Smit A, Hubley R. RepeatModeler Open-1.0. 2008:http://www.repeatmasker.org/.
40. Smit A, Hubley R, Green P. RepeatMasker Open-4.0. 2015:http://www.repeatmasker.org/.

41. Nawrocki EP, Eddy SR. Infernal 1.1: 100-fold faster RNA homology searches. Bioinformatics 2013; **29**:2933-2935.
42. Korf I. Gene finding in novel genomes. BMC Bioinformatics 2004; **5**:59.
43. Slater GSC, Birney E. Automated generation of heuristics for biological sequence comparison. BMC Bioinformatics 2005; **6**:31.
44. Grabherr MG, Haas BJ, Yassour M, et al. Trinity: reconstructing a full-length transcriptome without a genome from RNA-Seq data. Nature Biotechnology 2011; **29**:644.
45. Trapnell C, Williams BA, Pertea G, et al. Transcript assembly and quantification by RNA-Seq reveals unannotated transcripts and isoform switching during cell differentiation. Nature Biotechnology 2010; **28**:511.
46. Haas BJ, Salzberg SL, Zhu W, et al. Automated eukaryotic gene structure annotation using EVidenceModeler and the Program to Assemble Spliced Alignments. Genome Biology 2008; **9**:R7.
47. Zdobnov EM, Apweiler R. InterProScan–an integration platform for the signature-recognition methods in InterPro. Bioinformatics 2001; **17**:847-848.
48. Ashburner M, Ball CA, Blake JA, et al. Gene ontology: tool for the unification of biology. Nature Genetics 2000; **25**:25.
49. Kanehisa M, Goto S, Kawashima S, et al. The KEGG resource for deciphering the genome. Nucleic Acids Research 2004; **32**:277-280.
50. Boeckmann B, Bairoch A, Apweiler R, et al. The SWISS-PROT protein knowledgebase and its supplement TrEMBL in 2003. Nucleic Acids Research 2003; **31**:365-370.
51. Calcino AD, Kenny NJ, Gerdol M. Single individual structural variant detection uncovers widespread hemizygosity in molluscs. bioRxiv 2020:298695.
52. Li L, Stoeckert CJ, Roos DS. OrthoMCL: identification of ortholog groups for eukaryotic genomes. Genome Research 2003; **13**:2178-2189.
53. Edgar RC. MUSCLE: multiple sequence alignment with high accuracy and high throughput. Nucleic Acids Research 2004; **32**:1792-1797.
54. Stamatakis A. RAxML-VI-HPC: maximum likelihood-based phylogenetic analyses with thousands of taxa and mixed models. Bioinformatics 2006; **22**:2688-2690.
55. Kumar S, Stecher G, Suleski M, et al. TimeTree: a resource for timelines, timetrees, and divergence times. Mol Biol Evol 2017; **34**:1812-1819.
56. Yang Z. PAML: a program package for phylogenetic analysis by maximum likelihood. Computer Applications in the Biosciences 1997; **13**:555-556.
57. Rambaut A. FigTree, a graphical viewer of phylogenetic trees. 2007:http://tree.bio.ed.ac.uk/software/figtree/.
58. PicardToolkit. Broad Institute, GitHub Repository 2019:http://broadinstitute.github.io/picard/.
59. Mckenna A, Hanna M, Banks E, et al. The Genome Analysis Toolkit: A MapReduce framework for analyzing next-generation DNA sequencing data. Genome Research 2010; **20**:1297-1303.
60. Cingolani P, Platts AE, Wang LL, et al. A program for annotating and predicting the effects of single nucleotide polymorphisms, SnpEff: SNPs in the genome of *Drosophila melanogaster* strain w1118. Fly 2012; **6**:80-92.
61. Liu FY, Li YL, Yu HW, et al. MolluscDB: an integrated functional and evolutionary

genomics database for the hyper-diverse animal phylum Mollusca. Nucleic Acids Res 2020;**49**:D1556.

62. Smyth GK, Ritchie M, Thorne N, et al. LIMMA: linear models for microarray data. In Bioinformatics and Computational Biology Solutions Using R and Bioconductor. Statistics for Biology and Health. 2005.

63. Di G, Xiao X, Tong MH, et al. Proteome of larval metamorphosis induced by epinephrine in the Fujian oyster *Crassostrea angulata*. BMC Genomics 2020; **21**:675.

64. Eisenhofer G, Tian H, Holmes C, et al. Tyrosinase: a developmentally specific major determinant of peripheral dopamine. The FASEB Journal 2003; **17**:1248-1255.

65. Bonar DB, Coon SL, Walch M, et al. Control of oyster settlement and metamorphosis by endogenous and exogenous chemical cues. Bulletin of Marine Science 1990; **46**:484-498.

66. Joyce A, Vogeler S. Molluscan bivalve settlement and metamorphosis: neuroendocrine inducers and morphogenetic responses. Aquaculture 2018; **487**:64-82.

67. Narasimhan VM, Danecek P, Scally A, et al. BCFtools/RoH: a hidden Markov model approach for detecting autozygosity from next-generation sequencing data. Bioinformatics 2016; **32**:1749-1751.

68. Kenny NJ, Mccarthy S, Dudchenko O, et al. The Gene-Rich Genome of the Scallop *Pecten maximus*. GigaScience 2020;**9**:giaa037.

69. Feng DD. The hard-shelled mussel *Mytilus coruscus* - gene models, annotations and related files of the whole genome. Figshare 2020:doi:10.6084/m6089.figshare.10259618.

70. Yang J, Feng D, Liu J, Xu J, Chen K, Li Y et al. Supporting data for “Chromosome-level genome assembly of the hard-shelled mussel *Mytilus coruscus*, a widely distributed species from the temperate areas of East Asia.” GigaScience Database 2021 http://dx.doi.org/10.5524/100874

687 **Table 1 Statistics of whole genome sequencing using Illumina and ONT**

Types	Method	Library size (bp)	Reads number	Clean data (Gb)	length (bp)	coverage (x)
Genome	Illumina	300-350	1,235,384,620	160.6	150	104×
Genome	ONT	20,000	11,108,773	246.8	30,945 (N50)	159×
Genome	Hi-C	\	832,911,978	249.6	150	161×
Transcriptome	Illumina	300-350	787,692,308	102.4	150	\

688

Table 2 Results of contig anchoring on pseudochromosomes using Hi-C data

LG	Length (bp)	Gene Number	Contig N50 length (bp)	Contig Number	Number of Gaps (bp)
LG01	141,585,364	3,535	2,274,693	122	12,100
LG02	144,576,766	3,347	3,700,000	88	8,700
LG03	99,268,963	2,454	1,068,300	196	19,500
LG04	99,542,347	2,554	894,135	225	22,400
LG05	122,084,758	3,159	2,900,000	96	9,500
LG06	102,382,230	2,442	2,078,006	106	10,500
LG07	122,148,919	2,720	3,437,001	91	9,000
LG08	101,363,610	2,456	2,665,365	138	13,700
LG09	90,511,107	2,243	1,458,983	124	12,300
LG10	94,491,177	2,295	1,062,238	172	17,100
LG11	85,619,405	1,927	619,639	249	24,800
LG12	76,129,233	1,754	767,559	180	17,900
LG13	79,962,191	1,837	2,050,444	117	11,600
LG14	63,392,598	1,391	1,000,000	125	12,400
**Total**	**1,423,058,668**	**34,114**	**1,700,000**	**2,029**	**201,500**

Gaps are preset at 100 Ns.

Table 3 General statistics of the predicted protein-coding genes

Gene set		Number	Average transcript length (bp)	Average CDS length (bp)	Average exons per gene	Average exon length (bp)	Average intron length (bp)
De novo	SNAP	52,359	15,377	488	4.8	101	3,894
	GlimmerHMM	196,665	7,017	525	3.3	157	2,776
	Augustus	67,930	8,512	1,036	4.1	250	2,380
Homolog	*B. platifrons*	34,836	10,631	784	3.6	217	3,778
	M. philippinarum	27,088	7,174	643	2.8	227	3,568
Trans.orf/RNAseq		53,578	16,183	966	6.0	275	2,900
Final EVM models		37,478	14,735	1,290	5.9	217	2,727

Table 4 General statistics of gene functional annotation

Type		Number	Percentage (%)
Total		37,478	100.0
Annotated	InterPro	32,821	87.6
	GO	18,497	49.4
	KEGG	7,625	20.3
	Swissprot	16,868	45.0
	NR	31,489	84.0
Annotated		35,471	94.6
Unannotated		2,007	5.4

Figure 1. Sequenced individuals and sampling sites.

Click here to access/download;Figure;Fig1 Sequenced individuals and sampling sites.tif

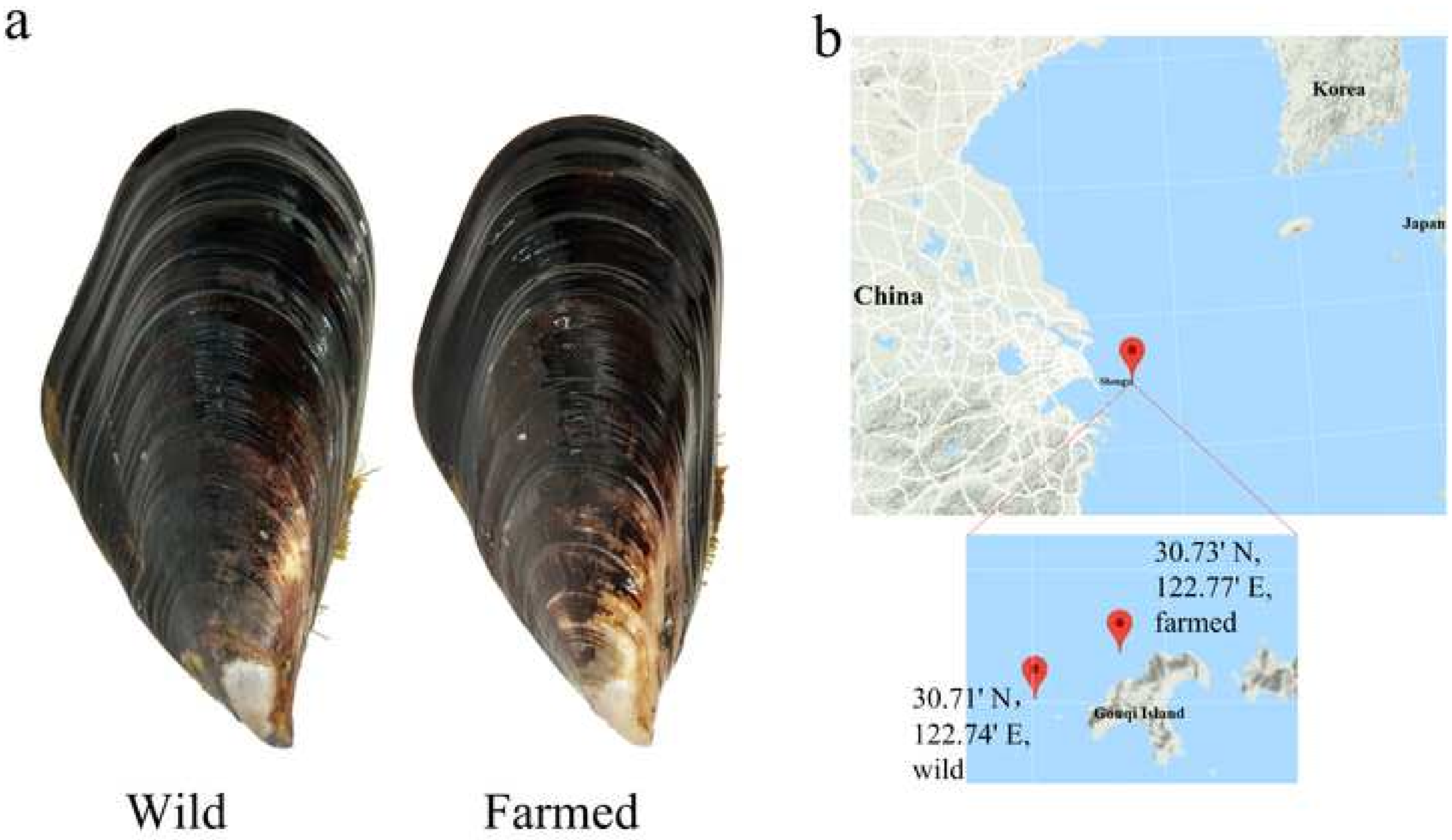

Figure 2. Workflow of genome sequencing and annotation

Click here to access/download;Figure;Figure 2. Workflow of genome sequencing and annotation.png

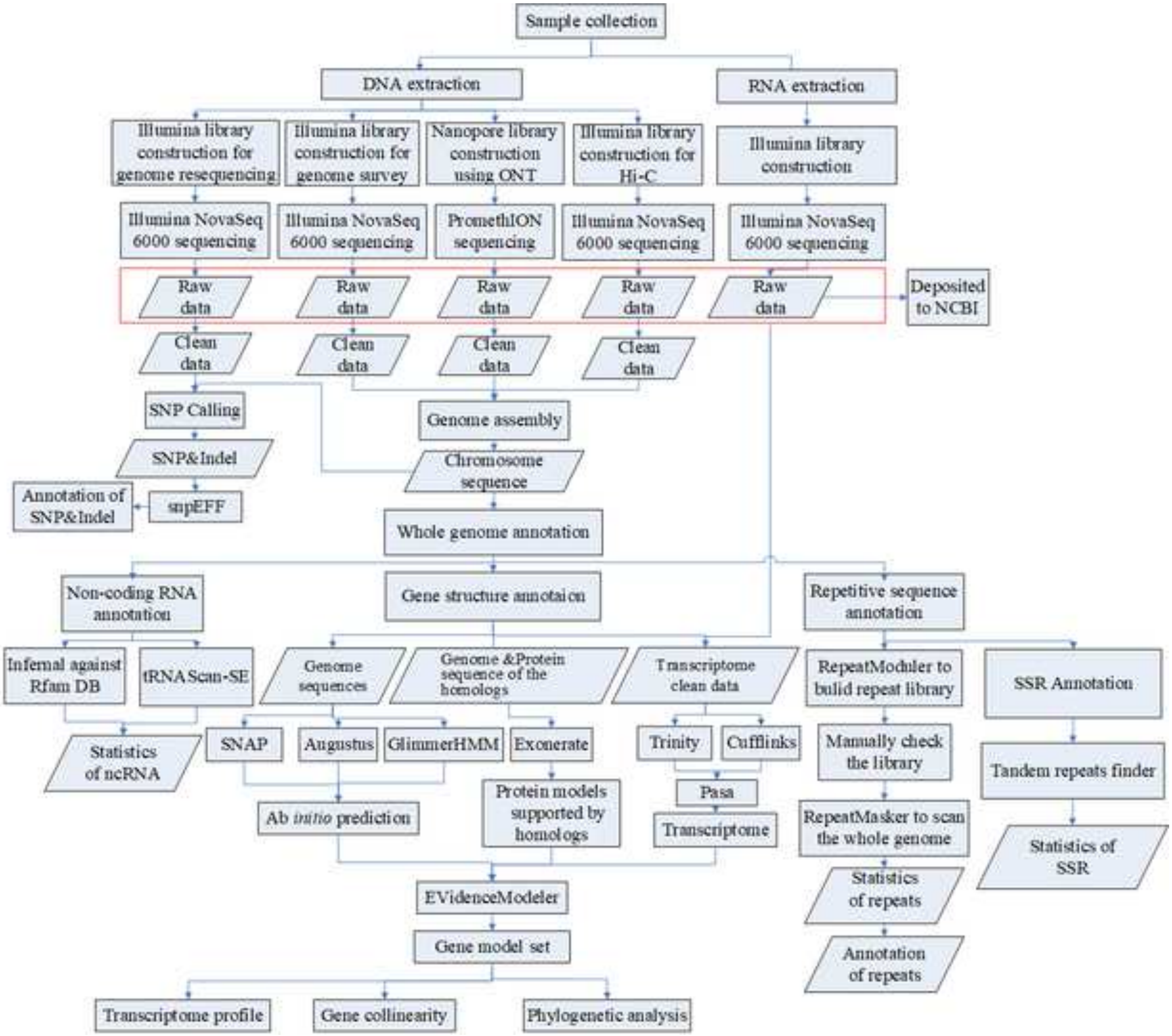

Figure 3. Annotation and evolution

Click here to access/download;Figure;Fig3 genome annotation and evolution.jpg

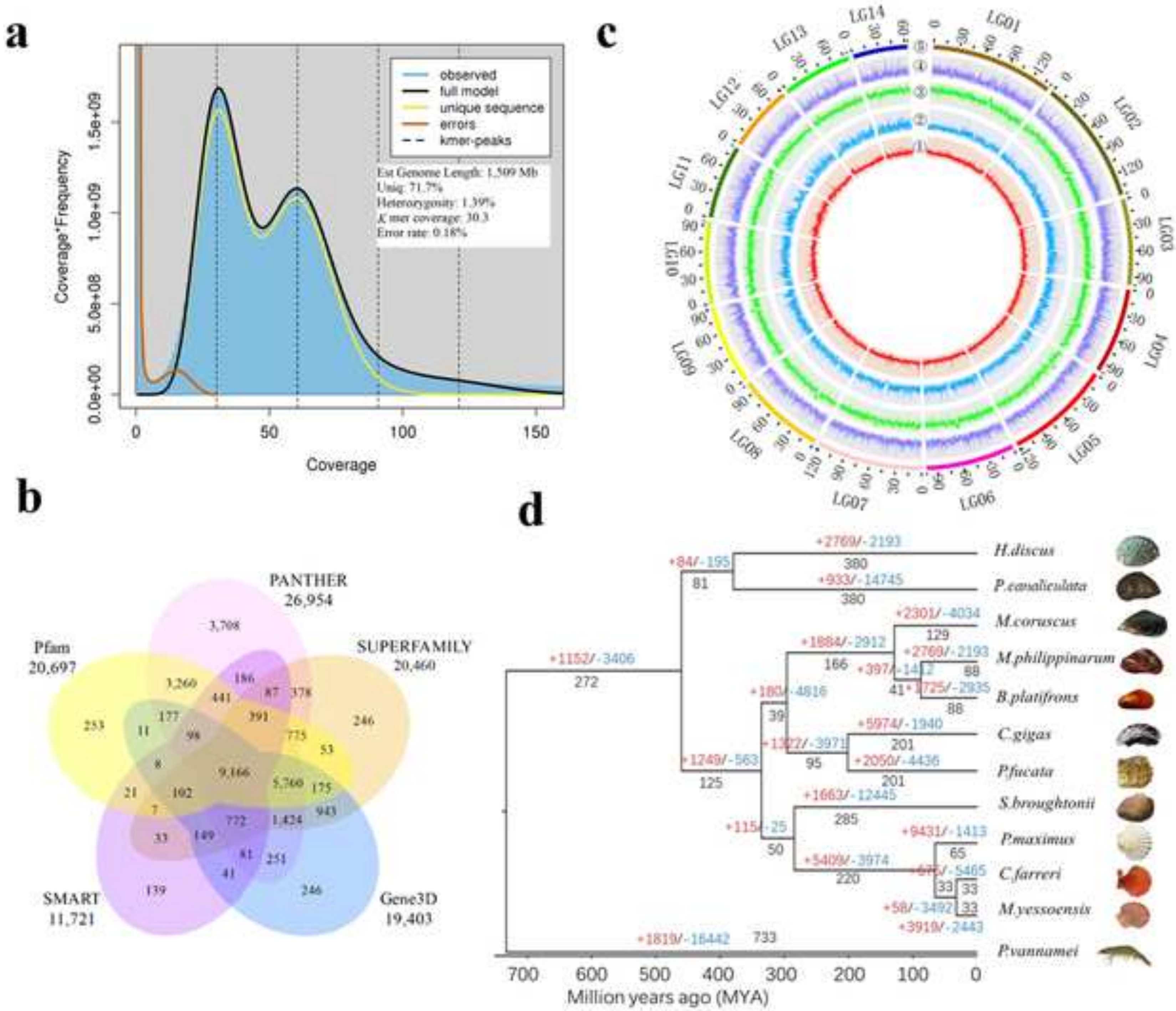

Figure 4. Sequence variations between farmed and wild populations

Click here to access/download;Figure;Fig4 Sequence variations between farmed and wild populations.png

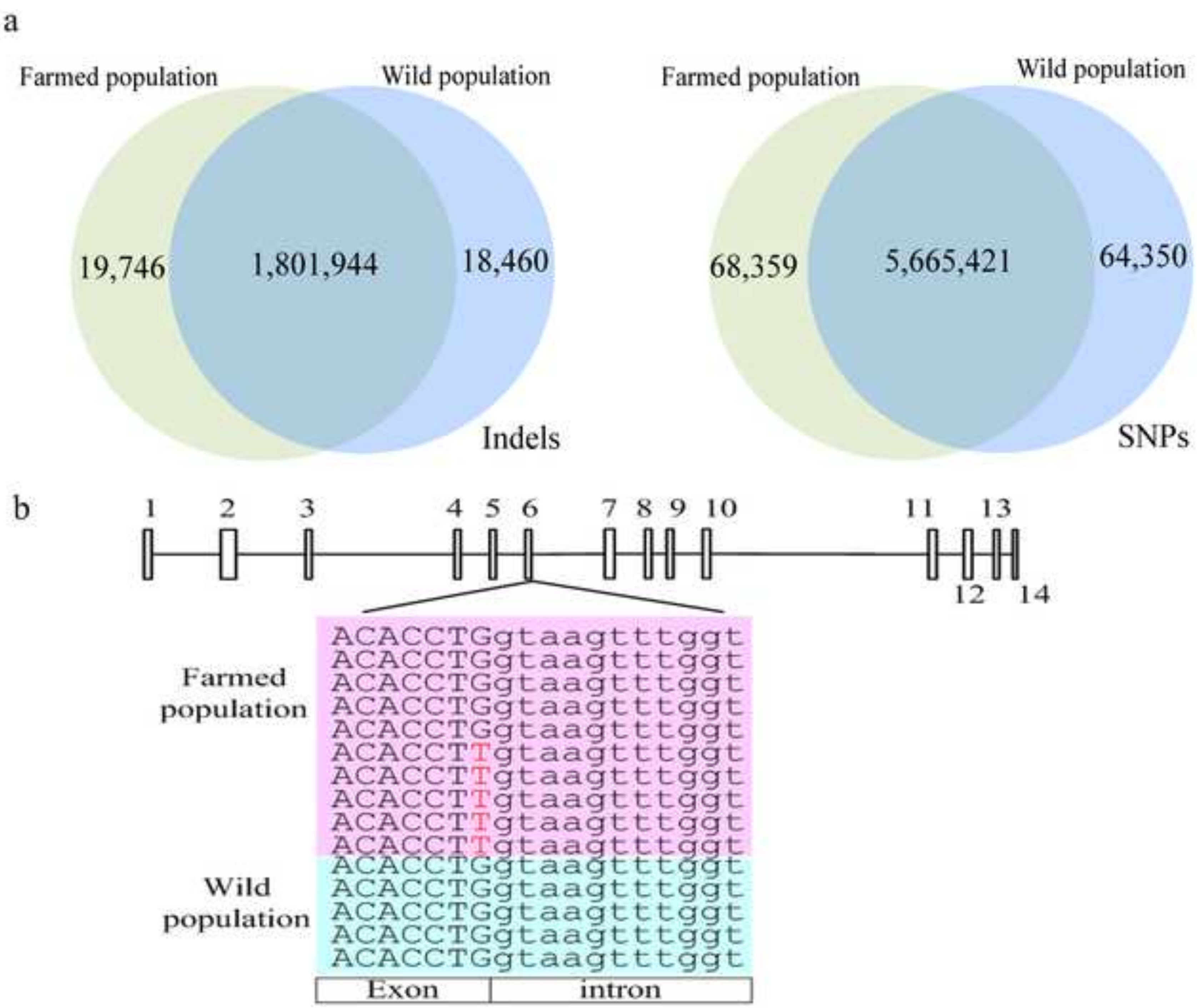

Figure 5. Chromosome synteny.

Click here to access/download;Figure;Fig5 Chromosome synteny20210222.jpg

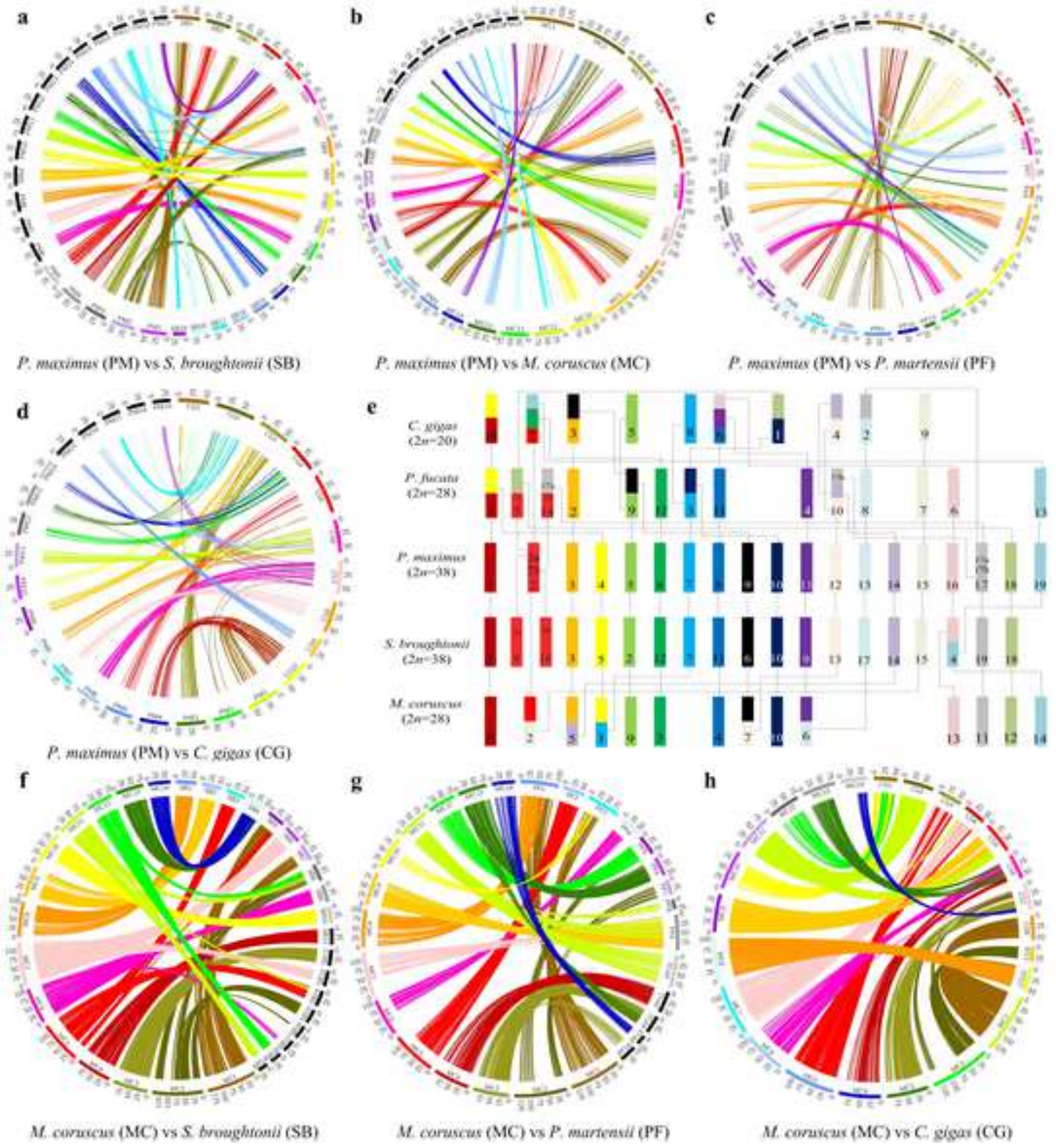

Figure 6. Spatial and temporal expression of genes involved in development and metamorphosis

Click here to access/download;Figure;Fig6 DoapmineSynpatic.png

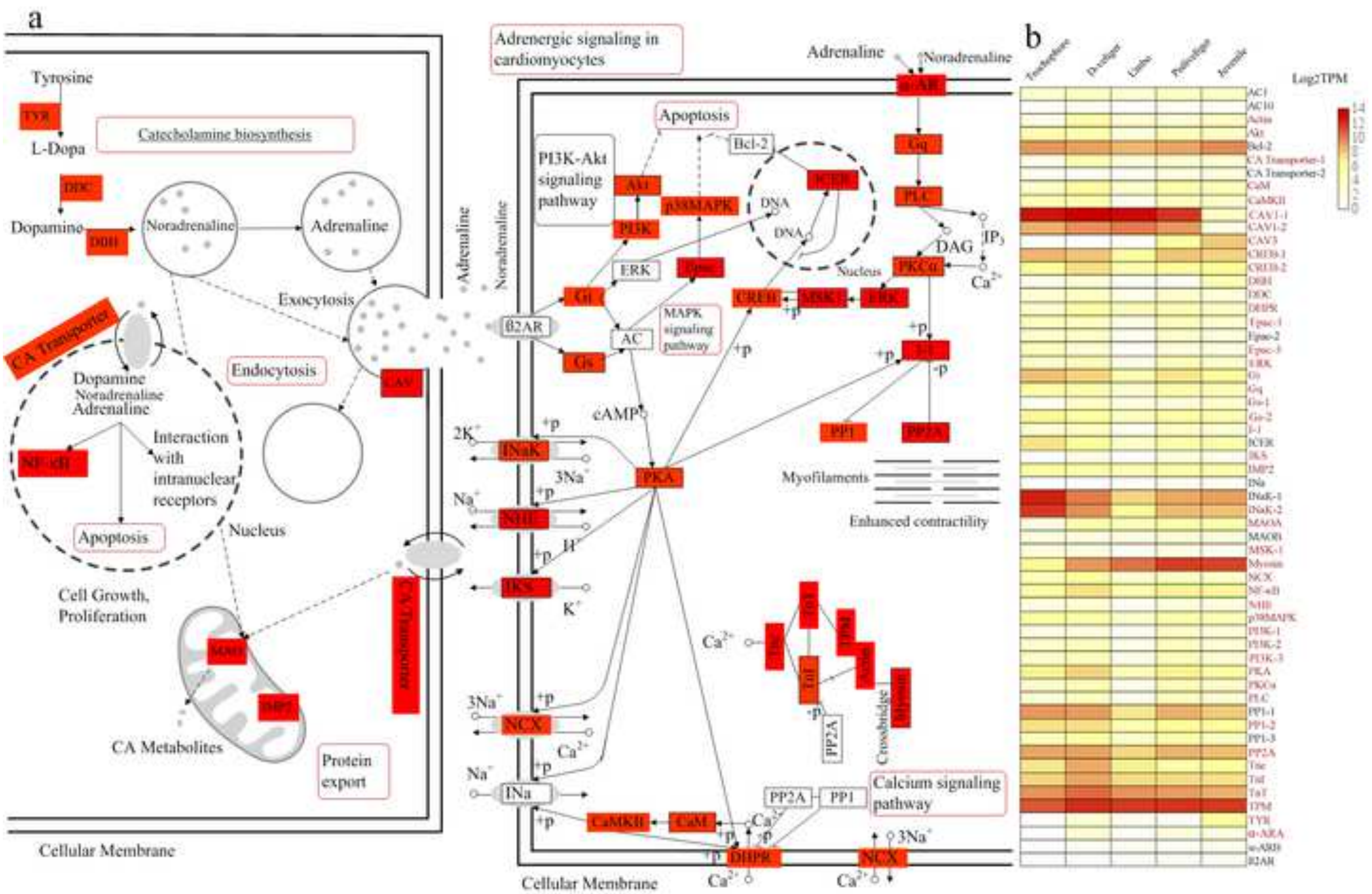

Supplementary Table S1. Repetitive sequences in the hard-shelled mussle genome

Click here to access/download
Supplementary Material
Supplementary Table S1 Repetitive sequences.xls

Supplementary Table S2. Overview of the predicted non-coding RNAs

Click here to access/download

Supplementary Material

Supplementary Table S2 Overview of predicted non-coding RNAs.xls

Supplementary Table S3. Bidirectional BLASTp

Click here to access/download
Supplementary Material
Supplementary Table S3 Bidirectional BLASTp.docx

Supplementary Table S4. Gene expression profiles during five developmental stages

Click here to access/download

Supplementary Material

Supplementary Table S4 The gene expression profile.xlsx

Supplementary Table S5. Genes involved in metamorphosis

Click here to access/download
Supplementary Material
Supplementary Table S5 Metamorphosis.docx

Supplementary Table S6. Information of the motifs with the characteristic of telomeric repeats

Click here to access/download
Supplementary Material
Supplementary Table S6 Telomeric.xlsx

point-by-point response

Click here to access/download
Supplementary Material
GIGA-D-20-00287R2-point-to-point_Responses22.doc

Personal Cover

Click here to access/download;Personal Cover;Cover_letter_Feb22Revision.docx

GIGA-D-20-00287R2

Dear Editor Prof. Hans Zauner,

We would like to appreciate the editors and the reviewers for taking the time to review the manuscript entitled "Chromosome-level genome assembly of the hard-shelled mussel *Mytilus coruscus*, a widely distributed species from the temperate areas of East Asia ". According to the reviewers' comments, the manuscript is revised by improving the experiments and the descriptions. **All of the corresponding alterations made in the revised main text are highlighted in red**. A point-by-point letter is uploaded to address the comments. At the same time, we have performed another round of proof reading to make small corrections elsewhere in the revised main text, none of which affect substance. The related data and codes are now available in the public database.

Main alterations in the revision:

1) Now, we are sure that the main tables are included. And total numbers of gaps are listed in Table 2 of the revision, which describes that each gap is preset as 100 Ns (Line 693-694).

2) We construct the synteny between *M. coruscus* and another four reported chromosome-level genomes in the revision (Fig. 5).

Looking forward to receiving your positive reply.

Best regards.

Jin-Long Yang (jlyang@shou.edu.cn) & Ying Lu (yinglu@shou.edu.cn)

College of Fisheries and Life Science, Shanghai Ocean University, Shanghai, China
