# Supplementary material for: Chromosome-level genome assembly of the hard-shelled mussel Mytilus coruscus, a widely distributed species from the temperate areas of East Asia
Source: Gigascience. 2021 Apr 23;10(4):giab024. doi: 10.1093/gigascience/giab024 (PMC8063583; doi:10.1093/gigascience/giab024)
Supplement: giab024_Supplemental_Files [file giab024_supplemental_files.zip › Supplementary Table S3 Bidirectional BLASTp.docx]

**Supplementary Table S3**. Bidirectional BLASTp between the previously published gene models of the hard-shelled mussel and the predicted gene models in this study.

| Relationship type of gene members in each family | Quantity of gene families (gene numbers in brackets) | |
| --- | --- | --- |
|  | Published draft assemblies (A) | Assemblies in this study (B) |
| One to one | 15,265 (15,265) | 15,265 (15,265) |
| One (A) to many (B) | 281 (281) | 281 (780) |
| Many (A) to one (B) | 3,531 (10,781) | 3,531 (3,531) |
| Many to many | 541 (2,904) | 541 (1,556) |
| A = B | 180 (413) | 180 (413) |
| A > B | 327 (2,369) | 327 (889) |
| A < B | 34 (122) | 34 (254) |
| Unique (only A or B) | 3,569 (12,154) | 538 (1,688) |
