# Supplementary material for: Chromosome-level genome assembly of the hard-shelled mussel Mytilus coruscus, a widely distributed species from the temperate areas of East Asia
Source: Gigascience. 2021 Apr 23;10(4):giab024. doi: 10.1093/gigascience/giab024 (PMC8063583; doi:10.1093/gigascience/giab024)
Supplement: giab024_Supplemental_Files [file giab024_supplemental_files.zip › Supplementary Table S5 Metamorphosis.docx]

**Supplementary Table S5.** Genes involved in the pathways of catecholamine biosynthesis and adrenergic signaling in the cardiomyocytes were reported to affect metamorphosis

| Gene_ID | Gene Symbol | Gene Function | Reference |
| --- | --- | --- | --- |
| LG01.g3505 | α-AR1 | The α-adrenergic receptor (α-AR) has been reported to effect larval metamorphosis in *Crassostrea gigas*, *Mytilus galloprovincialis*, and *Mytilus coruscus*. The α2-adrenergic receptor (Mca2AR) transcript was observed to be present during the entire process of larval development, Mca2AR-knockdown resulted in a substantial reduction in the abundance of the Mca2AR transcript and significantly inhibited the metamorphosis of *M. coruscus* larvae. “Adrenergic signaling in cardiomyocytes” pathway was identified to involve in larval metamorphosis in the Fujian oyster using quantitative proteomics. | (Coon and Bonar, 1987; Di et al., 2020; Liang et al., 2019; Yang et al., 2008; Yang et al., 2011) |
| LG08.g2668 | α-AR1 | The α-adrenergic receptor (α-AR) has been reported to effect larval metamorphosis in *C. gigas*, *M. galloprovincialis*, and *M. coruscus*. The α2-adrenergic receptor (Mca2AR) transcript was observed to be present during the entire process of larval development, Mca2AR-knockdown resulted in a substantial reduction in the abundance of the Mca2AR transcript and significantly inhibited the metamorphosis of *M. coruscus* larvae. “Adrenergic signaling in cardiomyocytes” pathway was identified to involve in larval metamorphosis in the Fujian oyster using quantitative proteomics. | (Coon and Bonar, 1987; Di et al., 2020; Liang et al., 2019; Yang et al., 2008; Yang et al., 2011) |
| LG02.g152 | ß2AR | The ß -adrenergic receptor (ß-AR) activating compounds were reported to effect larval metamorphosis in an oyster, *C. gigas*, a clam, *Meretrix meretrix*, and two mussels, *M. galloprovincialis* and *M. coruscus* | (Coon and Bonar, 1987; Wang et al., 2006; Yang et al., 2008; Yang et al., 2011) |
| LG01.g3223 | p38mapk | The p38 MAPK signaling regulate steps of metamorphosis after cilia loss in the tubeworm. | (Shikuma et al., 2016) |
| LG02.g1658 | AC1 | The adenylate cyclase/cyclic AMP (AC/cAMP) have been reported to mediate metamorphosis of larvae in the mussel. A sharp change in the cyclic AMP level and in the activity of adenylate cyclase enzyme seems to coincide with the moulting event during metamorphosis of moth. | (Hiripi and Rózsa, 1981; Liang et al., 2018) |
| LG05.g1154 | AC10 | The adenylate cyclase/cyclic AMP (AC/cAMP) have been reported to mediate metamorphosis of larvae in the mussel. A sharp change in the cyclic AMP level and in the activity of adenylate cyclase enzyme seems to coincide with the moulting event during metamorphosis of moth. | (Hiripi and Rózsa, 1981; Liang et al., 2018) |
| LG01.g4745 | CAV1-1 | Caveolin is larval-territory gene. The association of caveolin with larva-specific features exists in both protostomes (mollusc and annelid) and deuterostomes (tunicate). The strong expression of Caveolin will be lost during transition from the larval to the sessile juvenile stage. | (Wang et al., 2020) |
| LG01.g2677 | CAV1-2 | Caveolin is larval-territory gene. The association of caveolin with larva-specific features exists in both protostomes (mollusc and annelid) and deuterostomes (tunicate). The strong expression of Caveolin will be lost during transition from the larval to the sessile juvenile stage. | (Wang et al., 2020) |
| LG01.g1246 | CAV3 | Caveolin is larval-territory gene. The association of caveolin with larva-specific features exists in both protostomes (mollusc and annelid) and deuterostomes (tunicate). The strong expression of Caveolin will be lost during transition from the larval to the sessile juvenile stage. | (Wang et al., 2020) |
| LG12.g1248 | CaM | CaM and CaM-dependent myosin light chain kinase are suggested to function during larval settlement of the Barnacle *Balanus amphitrite* | (Chen et al., 2012) |
| LG01.g2240 | Gi | G protein-coupled receptor was widely reported to involve in metamorphosis in Molluscs and mussel. | (Baxter and Morse, 1987; Liang et al., 2018) |
| LG02.g2387 | Gs-1 | G protein-coupled receptor was widely reported to involve in metamorphosis in Molluscs and mussel. | (Baxter and Morse, 1987; Liang et al., 2018) |
| LG06.g1564 | Gs-2 | G protein-coupled receptor was widely reported to involve in metamorphosis in Molluscs and mussel. | (Baxter and Morse, 1987; Liang et al., 2018) |
| LG14.g1367 | DBH | Dopamine beta-hydroxylase (DBH), playing a key role in the synthesis of catecholamines, was reported to regulate metamorphosis of the razor clam Sinonovacula constricta by RNAi and DBH inhibitor. Tyrosinase and other catecholoxidase as the phenoloxidase system are reported to involve in molting and metamorphosis in insects. | (Ashida, 1990; Decker and Tuczek, 2000; Li et al., 2020) |
| LG02.g4109 | DDC | Catecholamines are the most commonly used neurotransmitters in aquaculture to induce metamorphosis of mollusks` larvae. Aromatic-L-amino-acid decarboxylase (DDC) widely reported to involve in the biosynthesis of cathcolamine. DDC was reported to involve in veliger development and potentially earlier development in the Pacific oyster, Crassostrea gigas. | (Ashida, 1990; Decker and Tuczek, 2000; Vogeler et al., 2021) |
| LG13.g2754 | TYR | Catecholamines are the most commonly used neurotransmitters in aquaculture to induce metamorphosis of mollusks` larvae. Tyrosinase (TYR) is widely reported to involve in the biosynthesis of catecholamines. TYR and other catecholoxidase as the phenoloxidase system are reported to involve in molting and metamorphosis in insects. | (Ashida, 1990; Decker and Tuczek, 2000; Joyce and Vogeler, 2018) |
| LG08.g1476 | NF-κB | The finding of NF kappa B being involved in the immune response of the skin during amphibian metamorphosis were proposed to have potential implications for metamorphosis as well. The NF-kappa B signaling pathway is significantly enriched by differentially expressed genes during the larval settlement and metamorphosis of marine peanut worm. | (Cao et al., 2020; Heyland and Moroz, 2006) |
| LG13.g2084 | PI3K-1 | PI3K was reported to be involved in the metamorphosis of the abalone | (Wang et al., 2016) |
| LG13.g1739 | PI3K-2 | PI3K was reported to be involved in the metamorphosis of the abalone | (Wang et al., 2016) |
| LG13.g1738 | PI3K-3 | PI3K was reported to be involved in the metamorphosis of the abalone | (Wang et al., 2016) |

**Reference：**

Ashida M. Biochemistry of the phenoloxidase system in insects : With special reference to its activation, in molting and metamorphosis. Japan Sci Soc Press 1990:237-263.

Baxter G, Morse DE. G protein and diacylglycerol regulate metamorphosis of planktonic molluscan larvae. Proceedings of the National Academy of Sciences 1987; **84**:1867-1870.

Cao F, Zhong R, Yang C, et al. Transcriptomic analysis of differentially expressed genes in the larval settlement and metamorphosis of peanut worm *Sipunculus nudus*. Aquaculture Reports 2020;

Chen Z, Wang H, Matsumura K, et al. Expression of Calmodulin and Myosin Light Chain Kinase during Larval Settlement of the Barnacle *Balanus amphitrite*. PLOS ONE 2012; **7**.

Coon S, Bonar D. Pharmacological evidence that alpha1.-adrenoceptors mediate metamorphosis of the pacific oyster, *Crassostrea gigas*. Neuroscience 1987; **23**:1169-1174.

Decker H, Tuczek F. Tyrosinase/catecholoxidase activity of hemocyanins: structural basis and molecular mechanism. Trends in Biochemical Sciences 2000; **25**:392-397.

Di G, Xiao X, Tong MH, et al. Proteome of larval metamorphosis induced by epinephrine in the Fujian oyster *Crassostrea angulata*. BMC Genomics 2020; **21**:675.

Heyland A, Moroz LL. Signaling mechanisms underlying metamorphic transitions in animals. Integrative and Comparative Biology 2006; **46**:743-759.

Hiripi L, Rózsa KS: Second messengers and monoamine receptors in the regulation of metamorphosis of locusta migratoria migratorioides r. f. In: *Neurotransmitters in Invertebrates.* Edited by Rózsa KS: Pergamon; 1981: 235-253.

Li Z, Niu D, Peng M, et al. Dopamine beta-hydroxylase and its role in regulating the growth and larval metamorphosis in *Sinonovacula constricta*. Gene 2020; **737**:144418.

Liang X, Chen K, Li Y-F, et al. An ɑ2-adrenergic receptor is involved in larval metamorphosis in the mussel, *Mytilus coruscus*. Biofouling 2019; **35**:986-996.

Liang X, Chen YR, Gao W, et al. Effects on larval metamorphosis in the mussel *Mytilus coruscus* of compounds that act on downstream effectors of G-protein-coupled receptors. Journal of the Marine Biological Association of the United Kingdom 2018; **98**:333-339.

Shikuma NJ, Antoshechkin I, Medeiros JM, et al. Stepwise metamorphosis of the tubeworm *Hydroides elegans* is mediated by a bacterial inducer and MAPK signaling. Proceedings of the National Academy of Sciences of the United States of America 2016; **113**:10097-10102.

Wang G, Li N, Zhang L, et al. IGFBP7 is involved in abalone metamorphosis. Aquaculture 2016; **451**:377-384.

Wang G, Liu B, Tang B, et al. Pharmacological and immunocytochemical investigation of the role of catecholamines on larval metamorphosis by β-adrenergic-like receptor in the bivalve *Meretrix meretrix*. Aquaculture 2006; **258**:611-618.

Wang J, Zhang LL, Lian S, et al. Publisher Correction: Evolutionary transcriptomics of metazoan biphasic life cycle supports a single intercalation origin of metazoan larvae. Nat Ecol Evol 2020; **4**:766.

Yang JL, Glenn Satuito C, Bao WY, et al. Induction of metamorphosis of pediveliger larvae of the mussel *Mytilus galloprovincialis* Lamarck, 1819 using neuroactive compounds, KCl, NH4Cl and organic solvents. Biofouling 2008; **24**:461-470.

Yang JL, Li YF, Bao WY, et al. Larval metamorphosis of the mussel *Mytilus galloprovincialis* Lamarck, 1819 in response to neurotransmitter blockers and tetraethylammonium. Biofouling 2011; **27**:193-199.
