# Supplementary material for: Chromosome-level genome assembly of the hard-shelled mussel Mytilus coruscus, a widely distributed species from the temperate areas of East Asia
Source: Gigascience. 2021 Apr 23;10(4):giab024. doi: 10.1093/gigascience/giab024 (PMC8063583; doi:10.1093/gigascience/giab024)
Supplement: giab024_Response_to_Reviewer_Comments_Original_Submission [file giab024_response_to_reviewer_comments_original_submission.pdf]

Author's Response To Reviewer Comments

Close

RESPONSE LETTER
Ying Lu, January 2021
Dear Dr. Hans Zauner
Giga Science

Manuscript GIGA-D-20-00287R1.

Dear Reviewers and Editor, thank you for your time and valuable help in improving this manuscript. Please find below our detailed response letter (answers in blue) addressing in the comments.

Reviewer Comments:
Reviewer #1:

The manuscript by Yang and colleagues reports a high quality genome assembly for the mussel Mytilus coruscus. Although this is not the first genome assembly published for this species, this resource is an improvement compared with the previous version, due to the use of Hi-C libraries and a better management of heterozygous genomic regions. Hence, the contents of this work appear to be appropriate for a data note article. there are however several points that would require some additional information to be added, and bits of text that need to be modified to improve the flow of the text.
Dear reviewer, thank you for your comments and suggestions to improve this manuscript. As requested, we included more information about PAV and the genomic coverage, improved the language by a native English speaker, and made other corrections as suggested.

General comments:
I would suggest the authors to specify the sequencing coverage achieved somewhere in the text (i.e. which coverage was obtained with ONT reads? Which coverage was obtained with Illumina PE? Etc.). This is present in Table 1, but it should be also mentioned in the text.
Thanks for your suggestion, we have specified the sequencing coverage in the text (Line 127, Line 134 and Line 136).

The authors emphasized the high heterozygosity of the genome, pointing out the possible links between SNPs and phenotypic variation. The authors may not be aware of the very recent discoveries that currently indicate that bivalve genomes are characterized by significant hemizygosity and structural variants that affect gene content, resulting in massive gene presence/absence variation. While the authors are not currently required to update this work with a detailed analysis of PAV, I think the text might benefit from some additional points of discussion, especially considering the fact that a congeneric mussel species, M. galloprovincialis, has been shown to be characterized by an astounding level of intraspecific genomic variation (see the preprint by Gerdol et al. 2019, which has recently been accepted for publication and should become available online in the matter of a few weeks on genome Biology).
Also see the preprint by Calcino and colleagues here:
https://www.biorxiv.org/content/10.1101/2020.09.15.298695v1
Thanks for your suggestion. We have checked two related papers to understand the hemizygosity and PAV as described. The papers discovery that bivalve genomes are characterized by significant hemizygosity and structural variants that affect gene content. We cite that reference of the PAV in Line 225-226 and Line 277-279 as “which might be owing to the widespread hemizygosity and massive gene presence/absence variation (PAV) (Gerdol et al. 2020; Calcino et al. 2020)” and “In addition, PAV may play a role in determining phenotypic traits (Gerdol et al. 2020; Calcino et al. 2020), which should be included in the future re-sequencing analyses.”
Reference:
Gerdol M, Moreira R, Cruz F, et al. Massive gene presence-absence variation shapes an open pan-genome in the Mediterranean mussel. Genome Biology 2020; 21:275.
Calcino AD, Kenny NJ, Gerdol M. Single individual structural variant detection uncovers widespread hemizygosity in molluscs. bioRxiv 2020; 298695.

In general, I would recommend the authors to involve a native English-speaking colleague in the revision of the text, as several grammar errors and oddly constructed sentences are present throughout the text.
Thanks for your suggestion. The revised manuscript has been professionally edited by a native English-speaking colleague. The main alternations are highlighted in the revision.

Abstract
1.4: -correct "high-through"; I guess the authors were referring to "high throughput" here.
We replaced "high-through" with "high throughput" (Line 28).

1.5:-Correct "platifron" with "paltifrons"
We revised "platifron" into "paltifrons" (Line 36).

1.6:-"speculating their sharing same origins in evolution" please correct this odd wording.
Sorry for the confusion, we have corrected the sentence as "suggesting that this is shared ancestrally" (Line 38).

List of detailed comments
1.7:-Mussels have been also used as sentinel organisms for biomonitoring, and this information could be added to the list
Thanks for your suggestion, we have added biomonitoring in Line 54.

1.8:-"As with a dozen of marine invertebrates". This is unclear; I guess the authors meant "As several other marine invertebrates"
We rewrote the sentence (Line 61), as follows:
"As many other marine invertebrates, marine mussels also possess a free-swimming larval phase."

1.9:-When talking about the M. galloprovincialis genome assembly, the authors only refer to the paper by Murgarella and colleagues, whereas an improved version has been recently accepted for publication on Genome Biology (this should be probably available online within a few weeks). The text is available as a preprint, see Gerdol et al. https://www.biorxiv.org/content/10.1101/781377v1
Thanks for your notification, we added the reference of an improved genome of M. galloprovincialis by Gerdol et al (Line 80).

Methods
1.10:-This section in particular suffers from the presence of several issues with the quality of the language used, that should be improved.
Thanks for your suggestion, we have revised this section to improve the language by a native English speaker.

1.11:-When talking about the k-mer graph, please refer to the homozygous and heterozygous peaks (instead of "junior peak"
Thanks for your comment. The homozygous and heterozygous peaks are clarified in the revision (Line 152-154). Calculation of the k-mer occurrence is improved using GenomeScope.

1.12:-"very close to the total assemblies (1.57 Gb)". I think it would be worth mentioning that this is also not far from the c-value previously estimated by cytogenetic studies (see Ieyama, H., O. Kameoka, T. Tan, and J. Yamasaki (1994). Chromosomes and nuclear DNA contents of some species of Mytilidae. Venus 53: 327-331)
Thanks for your notification. We added the reference of the genome size estimated by cytogenetic studies (Ieyama et al. 1994) (Line 156).
Reference:
Ieyama H, Kameoka O, Tan T, et al. Chromosomes and nuclear DNA contents of some species in Mytilidae. Venus (Japanese Journal of Malacology) 1994; 53:327-331.

1.13:-"which is much greater than the real size of 1.57 Gb". This is also in line with what has been observed for M. galloprovincialis by Gerdol et al.
Yes. The same observation are added in Line 158-160, as follows:
"This kind of over-estimation for genome size usually occurred to the fragmented assemblies, like the recently published M. galloprovincialis genome, in which considerable heterozygous redundancies seem to be included in the assemblies."

Reference:
Gerdol M, Moreira R, Cruz F, et al. Massive gene presence-absence variation shapes an open pan-genome in the Mediterranean mussel. Genome Biology 2020; 21:275.

1.14:-"The yielded consensus sequences were manually checked by aligning to the GenBank database". This is a clever strategy, but I think it should be explained a bit better here.
Thanks. We revised the sentence as "The yielded consensus sequences were manually checked by aligning to the genes from the GenBank database (nt and nr; released in October 2019) to avoid that sequences of the high-copy genes are masked in following process with RepeatMasker".

1.15:-"which was less than previously-published 42,684 gene models in the draft genome because it introduced over 20% heterozygous redundancies in the assemblies". I agree with this consideration, but in light worth the recent findings about widespread hemizygosity and massive gene presence/absence variation in M. galloprovincialis, the authors might want to update the text with a few additional considerations.
We agree with that widespread hemizygosity and massive gene PAV probably cause the redundancies since it has been identified in M. galloprovincialis, as well as other molluscs (Gerdol et al. 2020; Calcino et al. 2020), as follows (Line 222-226):
"Using a bidirectional BLASTp between the two assemblies, we observed that an considerable heterozygous redundancies (over 20%) were probably included into the previous draft assemblies (Supplementary Table 3), which might be owing to the widespread hemizygosity and massive gene presence/absence variation (PAV) (Gerdol et al. 2020; Calcino et al. 2020) or assembling errors."
Reference:
Gerdol M, Moreira R, Cruz F, et al. Massive gene presence-absence variation shapes an open pan-genome in the Mediterranean mussel. Genome Biology 2020; 21:275.
Calcino AD, Kenny NJ, Gerdol M. Single individual structural variant detection uncovers widespread hemizygosity in molluscs. bioRxiv 2020; 298695.

1.16:-"448 single-copy genes". How were such genes identified? Was BUSCO/OrthoDB used for this?
Sorry for your confusion, we used OrthoDB to find the single-copy gene (Line 237).

1.17:-"Whole genome re-sequencing of farmed and wild individuals". The data provided here are potentially interesting for a preliminary analysis, but the authors should keep in mind (and briefly discuss) the possibility that higher-order structural variants which include gene PAV might have a very important role on phenotypic traits.
Thanks for your notification, we have included the PAV in the discussion in whole genome re-sequencing, as follows:
"In addition, PAV may play a role in determining phenotypic traits (Gerdol et al. 2020; Calcino et al. 2020), which should be included in the future re-sequencing analyses."
Reference:
Gerdol M, Moreira R, Cruz F, et al. Massive gene presence-absence variation shapes an open pan-genome in the Mediterranean mussel. Genome Biology 2020; 21:275.
Calcino AD, Kenny NJ, Gerdol M. Single individual structural variant detection uncovers widespread hemizygosity in molluscs. bioRxiv 2020; 298695.

1.18:-"consistent with their closest phylogenetic relationship in the Bivalvia clade" please add a reference for this.
Thanks. We add the Reference (Liu et al. 2020) for this.
Liu F, Li Y, Yu H, Zhang L, Hu J, Bao Z, Wang S. MolluscDB: an integrated functional and evolutionary genomics database for the hyper-diverse animal phylum Mollusca. Nucleic Acids Res. 2020 Nov 21:gkaa1166. doi: 10.1093/nar/gkaa1166. Epub ahead of print. PMID: 33219684.

1.19:-It would have been more appropriate to use TPM instead of FPKM？？, as this metric allows a more reliable comparison among samples.
Thanks for your suggestion, we use TPM instead of FPKM in transcriptome analysis (Supplementary Table S4, Line 318-320).

1.20:-"indicative of a 91.9% genome completeness when 89.98% of core metazoan orthologs were completely identified in the assemblies." This is somewhat unclear. Does 91.9% indicate present BUSCOs and 89.98% "present and complete"? Adding specific information concerning fragmented BUSCOs and duplicated or missing BUSCOs would help here.

As your suggested, we revised the sentence as "We assayed the genome completeness using Benchmark Universal Single-Copy Orthologs BUSCO v4.1.4 referencing metazoan and molluscan gene sets. In the metazoan dataset, the current assemblies have 89.4% complete (of which 1.0% were duplicated), 1.9% incomplete and 8.7% missing BUSCOs, corresponding to a recovery of 91.3% of the entire BUSCO set. In the molluscan dataset, 85.5% complete (of which 1.3% were duplicated), 0.8% incomplete and 13.7% missing BUSCOs were recorded, corresponding to 86.3% of the entire BUSCO set."

Reviewer #2: Yang et al present the genome assembly of the hard-shelled mussel Mytilus coruscus, alongside gene predictions and analysis of genome content. The work in assembling the genome and predicting genes is technically sound. However, the presentation of this work is inprecise, not yet of publishable standard, and would benefit from careful editing for science and language before re-submission. There are also several scientific points that need to be addressed, to ensure that the claims made in the manuscript are proportionate to the evidence presented. I have noted these below.

Major points to address:
2.1: 1) The authors claim that their genome represents a chromosome-level assembly of the genome of this species. This claim is based on the combination of reasonably long contigs into scaffolds using Lachesis based on linkage. To be able to firmly claim that these represent a "chromosome level assembly" it is necessary to evaluate the degree to which these pseudomolecules are assembled. Table 2 should provide data on the extent of gaps (total Ns) in each chromosome, and in the text, the size distribution of gaps, and information about them, should be noted. Are these, for instance, estimated and set at 100/1000 Ns? or are these a true reflection of the gap size? Is there any evidence of telomeric sequence at each end?
Thanks so much for your suggestions, we list the length (Ns) in the extents of the gaps (Table 2) in the revision. All of the gaps are set at 100 Ns, not the true reflection of the gap size. Total length of the gaps is 201.5 kbps (filled with 201.5 kbp Ns; Table 2; Line 180-181). We detect the characteristic motifs of telomeric sequences in 23 termini of the 13 chromosomes, suggesting the completeness of the assemblies (Supplementary Table S6; Line 359-361).

2.2: 2) There is a stark difference in estimated genome size between the previously published genome for this species and this resource. It would be useful to map the previous (draft) assembly of Li et al to this assembly and determine what percentage of the huge missing fragment (21%) of that assembly is truly missing from this assembly, and why. Does this represent uncollapsed heterozygosity (which would map twice to the same loci, presumably), intraspecific hemizygosity variation, or contamination in the previous genome resource? Or is it perhaps a problem of missing data in the assembly presented here? Any of these answers would be useful for understanding the genome of this species.
This is the good point. When the previous assemblies are aligned to present ones, a total of 141.8 Mb of genome sequences duplicates in the previous version while only 49.7 Mb in this resource (mapping rate of the Illumina reads against our assemblies was over 96.7%), indicating more heterozygous redundancies in the previous drafts. As far as the intraspecific hemizygosity variation reported in the M. galloprovincialis genome and other molluscan genomes (Gerdol et al. 2020; Calcino et al. 2020) is concerned, we do not have the evidence to clarify whether intraspecific hemizygosity variation results in different sizes of the assemblies. However, this reference is cited in the revision to demonstrate that over-estimation of the genome size sometime occurred to the draft assemblies (Line 158-160). In addition, comparative analysis of gene models also suggests considerably heterozygous duplicates in the previously published drafts (see response to 2.4).
Reference:
Gerdol M, Moreira R, Cruz F, et al. Massive gene presence-absence variation shapes an open pan-genome in the Mediterranean mussel. Genome Biology 2020; 21:275.
Calcino AD, Kenny NJ, Gerdol M. Single individual structural variant detection uncovers widespread hemizygosity in molluscs. bioRxiv 2020; 298695.

2.3: 3) Genome size estimation is carried out by a mathematical derivation directly from the highest peak size. This, however, partially excludes from consideration the heterozygous portion of the genome. As the assembly has been polished with Racon and Pilon, heterozygosity could also be underestimated by mapping estimates. It is recommended that alternate genome size estimates are provided. Genomescope (http://qb.cshl.edu/genomescope/genomescope2.0/) is a simple-to-use option that will provide more nuanced information regarding genome size and heterozygosity.
As you suggested, we re-estimated the genome size and heterozygous rate in the revision using Genomescope. The assessment of genome size by K-mer counting using GenomeScope suggested a complete genome size of approximately 1.51 Gb (Fig. 3a) (Line 149-151; Line 154-156). The present

genome had a heterozygous rate of 1.39 %, calculated by GenomeScope (Line 167-168).

2.4: 4) how many of the gene models found in Li et al are present/absent from the final gene set presented here? Were these used in the EVidenceModeler merge step? It is noted that "37,478 final gene models were generated (Table 3), which was less than previously-published 42,684 gene models in the draft genome because it introduced over 20% heterozygous redundancies in the assemblies". Please provide more information on how this was determined, as these extra genes could also represent recent duplicates, which should not be removed from consideration. This could build upon the results of 2) above.
The previous draft genome reported that the protein-coding gene set consists of 42,684 models (Li et al. 2020). However, we find 58,540 genes uploaded in GenBank, which is consistent with the gene numbers in their gff file. We compare the constructed gene families between the previous version and our annotations, using their 58,540 genes and our 37,478 genes. The gene duplicates are identified in the gene clusters of the two assemblies (see the following Table), in which A for the previously published genomes; B for the genome assemblies in this study. Quantity of the A-specific gene clusters that only consist of the genes from the previously published genome is significant higher than the B-specific ones that only consist of the genes from the assemblies in this study. Alignments against the NR database and repeat sequence library exhibits that 12,123 A-specific gene clusters (20.71% of 58,540) are annotated as transposable elements. The genes clustered in the families with more A members is much more than those in the families with more B members. We also find some genes with the same loci, splicing and even intron sequences. All of the information reflected a significant over-estimation in both genome size and quantity of protein-coding genes (Line 222-226).
Supplementary Table S3. Bidirectional BLASTp between the previously published gene models of the hard-shelled mussel and the predicted gene models in this study.
Relationship type of gene members in each family Quantity of gene families (gene numbers in brackets)
Published draft assemblies (A) Assemblies in this study (B)
One to one 15,265 (15,265) 15,265 (15,265)
One (A) to many (B) 281 (281) 281 (780)
Many (A) to one (B) 3,531 (10,781) 3,531 (3,531)
Many to many 541 (2,904) 541 (1,556)
A = B 180 (413) 180 (413)
A > B 327 (2,369) 327 (889)
A < B 34 (122) 34 (254)
Unique (only A or B) 3,569 (12,154) 538 (1,688)

Reference: Li RH, Zhang WJ, Lu JK, et al. The whole-genome sequencing and hybrid assembly of Mytilus coruscus. Frontiers in Genetics 2020; 11:1-6.

2.5: 5) The phylogeny as presented needs further consideration. Was concatenation of genes performed before alignment? (page 11) This could introduce errors at the start and end of each gene as they can artifactually be aligned to non-homologous sequences. This should be checked, and repeated correctly if necessary (with alignment performed gene-by-gene, then concatenating the alignments). -What maximum likelihood model was used? what other settings? how many bootstraps? Please note in text (page 11). -How was divergence time calculated?
Sorry for the confusion. Alignment of one-to-one single copy genes is prior to concatenation of the alignments. The corresponding sentences “448 single-copy genes identified by OrthoDB were aligned and concatenated. The amino acid sequences were first aligned using MUSCLE, which were further concatenated to create one supergene sequence for each species and formed a data matrix” (Line 236-239) are corrected in the revision.
Line240-246: The phylogenetic relationship was constructed using the Maximum-likehood model in RAxML version 8 with the optimal substitution model of PROTGAMMAJTT. Robustness of the maximum-likelihood tree was assessed using the bootstrap method (100 pseudo-replicates). Furthermore, the single-copy orthologs and one reference divergence time on the root node obtained from TimeTree database (http://www.timetree.org) were used to calibrate the divergence dates of other nodes on this phylogenetic tree by MCMCTREE tool in PAML package.

2.6: 6) In the "Whole genome re-sequencing of farmed and wild individuals" section, the assumption that sequence variations are farmed- population-specific (FPS) or wild-population-specific (WPS) is flawed as it is based on a tiny sample (20 individuals) of the enormous diversity of this species. It is not convincing to claim that these variants are unique to either farmed populations or wild populations - they are just observed to be different here due to the limited sampling. The depth of sequencing is also very low per individual (around 2.5x) and SNPs/indels could be missed. This section, and the claims

made from it in the abstract and conclusions, need to be substantially reworked to avoid drawing universal conclusions from what are only initial pilot results.
Thanks for your suggestions. We re-write this section and weaken the claims made from it in the abstract and conclusion since this is just a preliminary try in the genome study. A simple case is added in the revision to illustrate the diversities between farmed and wild populations. We only make a brief speculation that sequence variation might be associated with morphological diversity (Line 276-277).

2.7: 7) The differential expression analysis in larvae is not convincing. Many of the genes cherry-picked for discussion and shown in Fig 6 are expressed in all samples. As only single libraries were sequenced for each larval life stage, claims for differential expression are only very weakly supported. It is good practice to use a minimum of 3 separate samples per condition for DE analysis, and preferentially more. The authors should moderate the strength of the conclusions drawn in the "Transcriptome related to metamorphosis" section considerably, in light of the strength of some of the evidence presented.
Thanks for your suggestion. We have used 3 biological replicates' RNA-Seq data of five developmental stages (SRR13364385、SRR13364374、SRR13364373、SRR13364371、SRR13364370、SRR13364369、SRR13364368、SRR13364367、SRR13364383、SRR13364382、SRR13364381、SRR13364380、SRR13364378、SRR13364377、SRR13364376) to analyze the differential expression with normalized gene expression levels TPM (Line 317-319). We moderated the conclusion by removing the strong claims as "Signal transduction controlling the metamorphosis development seemed to activate during the first two stages, trochophore and D-veliger, although the major morphologic changes represented in the transition from pediveliger to juvenile".

Minor points:

2.8: -The authors are often too strong in their criticism of the earlier genomes for this species and Mytilus. For instance "a low quality draft genome of M. coruscus has been reported" (pg 4). That resource is not as well-contiged, but saying it is low quality is not justified. Perhaps "Draft versions of the genomes of M. coruscus and M. galloprovincialis have been reported". This kind of strong claim should be toned down throughout the manuscript.
We deleted "a low quality" and corrected the sentence as "a draft genome of M. coruscus and an improved genome of M. galloprovincialis have been reported" (Line 79-80).

2.9: - Many of the steps shown in Fig 2 (e.g. read cleaning) are not covered in sufficient detail in the manuscript. Please ensure that the steps required to recapitulate this work are provided.
Thanks. We added the details to describe the steps in Fig 2, as follows:
Line 143-146: The raw reads from Illumina sequencing platform were cleaned using FastQC45 and HTQC46 by the following steps: (a) filtered reads with adapter sequence; (b) filtered PE reads with one reads more than 10% N bases; (c) filtered PE reads with any end has more than 50% inferior quality (≤5) bases.
Line 189-192: The yielded consensus sequences were manually checked by alignment to genes from the GenBank database (nt and nr; released in October 2019) to avoid that sequences of the high-copy genes are masked in following treatment with RepeatMasker.
Line 236-246: Gene clusters were identified among 12 selected genomes … calibrate the divergence dates of other nodes on this phylogenetic tree using the MCMCTREE tool in the PAML package.

2.10: -What settings were used for OrthoMCL?
The settings used for OrthoMCL are a BLASTp value cutoff of 1e−5 and an inflation parameter of 1.5 (Line 236).

2.11: -What settings were used to detect PCR duplicates with Picard?
Thanks for your notification, the duplicate reads were removed with the MarkDuplicates tool of Picard (Line 257-258).

2.12: Fig 3d: caniculata seems to be mis-spelled
Thanks for your notification, we have corrected "canaliculate" into "canaliculata" in Fig 3d.

2.13: Fig 5: Why is P. fucata highlighted? Why not show P. maximus vs Mytilus coruscus? It is the most relevant for this paper. Fig 5a and Fig 5b might be the wrong images?
We illustrate the chromosome synteny of P. maximus vs S. broughtonii, P. maximus vs M. coruscus, P. maximus vs P. fucata, and P. maximus vs C. gigas (Figure 5). We did not highlight P. fucata. Given that the genome of P. maximus was reported to be a slow-evolving genome with many ancestral features,

the P. maximus is selected as a reference to compare with other four chromosome-level bivalves.

Note on language and scientific accuracy:
2.14: Throughout the manuscript there are minor errors in written english, which regularly introduce scientifically inaccurate statements. I have noted some of these below but my list is not complete, and the authors may wish to have their manuscript read over more thoroughly before resubmission. I have not had the time to correct all the errors present in the manuscript.
Thanks for your suggestion. The revised manuscript has been professionally edited by a native English-speaking colleague. The main alternations are highlighted in the revision.

2.15: Throughout: Please refer to the species name or the common name, but not "marine mussel" when you mean Mytilus coruscus - most mussels are marine. Similarly, do not use this to refer to all mussels.
Thanks for your notification, we have referred to the common name in revision (Line 41).

2.16: Title: the authors should consider introducing a comma into their title, breaking it into precise units: e.g. "A chromosome-level genome assembly of the hard-shelled mussel Mytilus coruscus, a widely distributed species from temperate areas of East Asia"
As your suggested, we corrected the title as "Chromosome-level genome assembly of the hard-shelled mussel Mytilus coruscus, a widely distributed species from the temperate areas of East Asia"

Abstract:
2.17: -no "A" in : A chromosome-level genome information
Thanks for your notification, we have deleted "A" in : A chromosome-level genome information (Line 24).

2.18: -high-through - do you mean high-throughput?
Thanks for your notification, we have corrected “high-through” as “high-throughput” (Line 28).

2.19: -" The completeness test exhibits" - I think you mean "comparison to the CEGMA metazoan complement reveals"
Thanks for your notification, we have corrected as "Comparison to the Core Eukaryotic Genes Mapping Approach (CEGMA) metazoan complement revealed" (Line 28).

2.20:-No "The" in "The phylogenetic analysis"
Thanks for your notification, we have revised "The phylogenetic analysis " into " Phylogenetic analysis " (Line 35).

2.21:-"the closest relationship between" - this is not true. I think you mean "phylogenetic analysis shows M. coruscus is the sister taxon to the clade comprised of Modiolus philippinarum and Bathymodiolus platifrons". Note spelling of last species
Thanks for your notification, we have revised the describtion of " the closest relationship between " into " Phylogenetic analysis showed that M. coruscus is a sister taxon to the clade including Modiolus philippinarum and Bathymodiolus platifrons. ", and we have corrected "Bathymodiolus paltifrons " into "Bathymodiolus platifrons " (Line 35-36).

2.22:-No "A", in "A conserved chromosome synteny "
Thanks for your notification, we have deleted "A" in "A conserved chromosome synteny" (Line 36).

2.23:-"speculating their sharing same origins in evolution" do you mean "suggesting that this is shared ancestrally"? Because the former is contentious
Thanks for your notification, we have corrected the sentence as "suggesting that this is shared ancestrally" (Line 38).

2.24:-no on in "studying on"
Thanks for your notification, we have deleted "on" in "studying on" (Line 42).

Context:
2.25:-phylum Mollusca (not Mollusc).
Thanks for your notification, we have corrected "Mollusc" as "Mollusca" (Line 47).

2.26:-"sea mussels". This is an inprecise phrase. Perhaps just use "mussels"

Thanks for your notification, we have revised "sea mussels " into "mussels" (Line 49).

2.27:- " Although their significance" - should read "Although they are significant for biology, ecology and the economy"
Thanks for your notification, we have revised " Although their significance in biology, ecology and economy " into " Although they are significant for biology, ecology and the economy " (Line 56-57).

2.28:- need an "and" before ", settlement mechanism."
Thanks for your notification, we have add an "and" before "settlement mechanism" (Line 60).

2.29:-"As with a dozen of marine invertebrates" - this is a deeply inaccurate statement. Perhaps "As with many marine invertebrates".
Thanks for your notification, we have revised " As with a dozen of marine invertebrates " into " As many marine invertebrates" (Line 61).

2.30:-"modeling of their anatomy " not "modeling of anatomy "
Thanks for your notification, we have revised " modeling of anatomy " into " remodeling of their anatomy " (Line 63).

2.31:-"trigger settlement and metamorphosis is universal in metazoan" - this is not true. Humans, for instance, are metazoans
Thanks for your notification, we have corrected "universal in" as " widespread among " (Line 68).

2.32:- "temperate areas" not "the temperate"
Thanks for your notification, we have revised " the temperate " into " temperate areas " (Line 71).

2.33:-"need adapt..." should read "needs to adapt to the hostile..."
Thanks for your notification, we have revised "need adapt to the hostile" into " needs to adapt to the hostile" (Line 74-75).

2.34:-"Up to date, chromosome level genome" should read "To date, a chromosomal-level genome"
Thanks for your notification, we have revised "Up to date, chromosome level genome" into " To date, no genome of any member of the genus Mytilus " (Line 78).

2.35:-"Lacking whole-genome information" should read "The lack of whole-genome information".
Thanks for your notification, we have revised " Lacking whole-genome information " into " The lack of whole-genome information " (Line 80-81).

2.36:-"The larvaes at five ..." should read "Larvae at five....".
Thanks for your notification, we have revised " The larvaes at five ... " into " Larvae at five.... " (Line 89).
2.36:-"gene expression" not "gene expressions"
Thanks for your notification, we have corrected "gene expressions" into "gene expression" (Line 90).

Methods:
2.38:-"where is the central coast of Chinese mainland" should read "the central coast of the Chinese mainland"
Thanks for your notification, we have revised "where is the central coast of Chinese mainland" into "which is the central coast of the Chinese mainland" (Line 97).

2.39:- "a" needed, A female wild adult with a mature ovary (although these are probably paired but difficult to detect - if paired this would be "with mature ovaries".)
Thanks for your notification, we have added " a " in " mature ovary " (Line 100), which was reported to be a mature ovary in mussel.

2.40:-" for the adductor muscle to isolate high molecular weight genomic DNA for sequencing of reference genome" should read ", with the adductor muscle taken for isolation of high molecular weight genomic DNA, for sequencing of the reference genome".
As your suggested, we have corrected the sentence as " and the adductor muscle was collected to isolate high-molecular-weight genomic DNA for the sequencing of the reference genome " (Line 101-102).

2.41:- no s "The DNAs"
Thanks for your notification, we have revised " The DNAs " into " The DNA" (Line 102).

2.42:-" to be assistant " should read "to assist with"
Thanks for your notification, we have revised " to be assistant " into " to assist with " (Line 101-106).

2.43:-" using SDS extraction method," should read " using the SDS extraction method," and a reference to this protocol should be given.
Thanks for your notification, we have added " the " in " using the SDS extraction method," and provided the reference (Eugene. 2000) (Line 109-110).
Sokolov EP. An improved method for DNA isolation from mucopolysaccharide-rich molluscan tissues, Journal of Molluscan Studies, 2000; 66 (4): 573–575, https://doi.org/10.1093/mollus/66.4.573.

2.44:-"total RNA were extracted" should read " total RNA was extracted "
Thanks for your notification, we have revised " were " into " was " (Line 114).

2.45:-"as well as the larvaes" should read "as well as larvae".
Thanks for your notification, we have corrected "larvaes" as " larvae".

2.46:"to get large segments " should read "to extract large fragments". fragments should be used instead of segments throughout this section.".
Thanks for your notification, we have revised " to get large segments " into " to extract large fragments " (Line 123). And we have corrected " segments " as " fragments ".

2.47:- The high quality library of average 20 kb in length was sequenced on the ONT PromethION platform with corresponding R9 cell and ONT sequencing reagents kit. The genomic DNA was sequenced using the MinION portable DNA sequencer with the 48 hours run script (Oxford Nanopore), which generated a total of 246.8 Gb data" were both the minion and promethion used? please make this clearer.
Sorry for your confusion, we only used PromethION platform and deleted the description of MinION portable DNA sequencer.

2.48:-" were fragmentized" should read " were fragmented"
Thanks for your notification, we have read "were fragmentized " into " was fragmented " (Line 129).

2.49:-novaseq needs a capital
Thanks for your notification, we have corrected " novaseq " as " NovaSeq " (Line 133).

2.50:- "by poly(A)" should read "for poly(A) transcripts". Which protocol was used?
Sorry for your confusion, we described the protocol as " The sample was enriched in mRNA by extracting poly(A) transcripts from total RNA using oligo-d(T) magnetic beads." (Line 138-139).

2.51:-" in 150 bp paired-end model." should read " in 150 bp paired-end mode."
Thanks for your notification, we have revised " in 150 bp paired-end model " into " in 150 bp paired-end mode " (Line 142).

2.52:-"Genome size of the hard-shelled " needs a "The" before
Thanks for your notification, we have revised " Genome size of the hard-shelled " into " The size of the hard-shelled mussel genome" (Line 149).

2.53: -" Average GC content of genome" needs a the before genome.
Thanks for your notification, we have revised " Average GC content of genome " into " an average GC content of genome " (Line 168).

2.54: -"The final assemblies is around 1.57 Gb" should be "The final assembly is around 1.57 Gb"
Sorry for the confusion, we have corrected the grammar (Line 165).

2.55: -"The genome assemblies of hard-shelled mussel" again should be assembly
Thanks for your notification, we have revised " The genome assemblies of hard-shelled mussel " into " The genome assembly of hard-shelled mussel " (Line 172).

2.56: -"with the softwares of Augustus (version 3.1) [38], GlimmerHMM (version 1.2) [39] and SNAP (version 2006-07-28) " should read "with Augustus (version 3.1) [38], GlimmerHMM (version 1.2) [39] and SNAP (version 2006-07-28) software"
Thanks for your notification, we have revised " with the softwares of Augustus (version 3.1) [38], GlimmerHMM (version 1.2) [39] and SNAP (version 2006-07-28) " into " using the Augustus (version 3.1) [38], GlimmerHMM (version 1.2) [39] and SNAP (version 2006-07-28) software " (Line 207-208).

2.57: -"protein sequences of two closed mollusc species" do you mean two closely related mollusc species?
Thanks for your suggestion, we have revised " two closed mollusc species " into "two closely related mollusc species" (Line 209).

2.58: -"Parallelly," should be "In parallel"
Thanks for your notification, we have revised " Parallelly," into "In parallel" (Line 211).

2.59: -"put into a de novo assemble" should be "assembled de novo"
Thanks for your notification, we have revised "put into a de novo assemble " into " assembled de novo " (Line 213).

2.60: -transnfer mis-spelled, Pg 9 (= transfer)
Thanks for your notification, we have corrected "transnfer" as "transfer " (Line 202).

2.61: -"The gene clusters were identified among 12 selected genome" should be "Gene clusters were identified among 12 selected genomes"
Thanks for your notification, we have revised "The gene clusters were identified among 12 selected genome " into " Gene clusters were identified among 12 selected genomes " (Line 229).

2.62: -"reflected the closest relationship between M. coruscus and the clade of M. philippinarum and B. platifrons," This is oddly stated. I think you mean "M. coruscus was found to be the sister taxon to the clade containing M. philippinarum and B. platifrons". Also, how was the divergence time calculated?
Thanks, we corrected the sentence as "M. coruscus is a sister taxon to the clade containing M. philippinarum and B. platifrons" (Line 248-249).

Sorry for the confusion, we revised the sentence into "single-copy orthologs and one reference divergence time on the root node obtained from the TimeTree database were used to calibrate the divergence dates of other nodes on this phylogenetic tree using the MCMCTREE tool in the PAML package" (Line 243-246).

2.63: - s needed, " in farmed and wild sample, respectively" should be " in farmed and wild samples, respectively"
Thanks for your notification, we have revised "in farmed and wild sample, respectively" into " in farmed and wild samples, respectively " (Line 255).

2.64:-"while 5,719,771 and 1,820,404 in wild one" should read "and 5,719,771 and 1,820,404 in wild populations"
Thanks for your notification, we have revised "while 5,719,771 and 1,820,404 in wild one " into " and 5,719,771 and 1,820,404 in wild populations "

2.65:-"The chromosome synteny illustrated that rare large-scale rearrangements between scallop and mussel, but frequent between scallop and oysters" should be rewritten "Chromosome synteny illustrates that large-scale rearrangements are rare between scallop and mussel, but more frequent between scallop and oysters"
Thanks for your notification, we have corrected the sentence as "Chromosome synteny illustrates that large-scale rearrangements are rare between scallop and mussel, but more frequent between scallop and oysters" (Line 292-294).

2.66:-No s "almost all of the chromosomes rearrangements " - should be "almost all of the chromosome rearrangements "
Thanks for your notification, we have revised " almost all of the chromosomes rearrangements " into " almost all of the chromosome rearrangements " (Line 308).

2.67:-"To profile the gene expressions" should be "To profile gene expression"

Thanks for your notification, we have revised " To profile the gene expressions " into " To profile gene expression "

2.68:-"Quality of the assembled genome" should read "The quality of the assembled genome.... "
Thanks for your notification, we have revised "Quality of the assembled genome " into " The quality of the assembled genome " (Line 349).

2.69:-"in genome assemble" should read "in the genome assembly"
Thanks for your notification, we have revised " in genome assemble " into " in the genome assembly " (Line 364-365).

2.70:-"facilitate a wide range of researches in mussel, bivalve, and molluscan." needs another word after molluscan - molluscan biology, maybe?
Sorry for the confusion, we have corrected as " mussels, bivalves, and mollusks " (Line 374).

2.71:-"evolution in bivalve" should be "evolution in bivalves"
Thanks for your notification, we have revised " evolution in bivalve " into " evolution in bivalves " (Line 375).

2.72:-"As one of the best-assembled bivalve genomes" - this is too strong a claim given the evidence presented.
Thanks for your suggestions, we have revised "As one of the best-assembled bivalve genomes" into " As one of the chromosome-level genome assemblies in Bivalve " (Line 376-377).

2.73:Please note there are numerous additional language problems to correct, and this is beyond the scope of my review. I suggest a careful re-reading of the manuscript before resubmission.
Sorry for the confusion, we have re-read and revised the manuscript thoroughly. The revised manuscript has been professionally edited by a native English-speaking colleague.

Reviewer #3: This study presented a high-quality genome of the mussel Mytilus coruscus. Using a mixed strategy to combine Illumina short reads and Nanopore long reads followed by scaffolding with Hi-C, the authors generated a chromosomal-level genome assembly. They further re-sequenced farmed and wild individuals to detect SNP and indel differences among the two populations. The authors then focused on the pathways related to larval settlement and metamorphosis using RNA-seq analysis. Overall, the genome quality looks good, but I have a few questions on how the authors analyzed and interpreted genome and transcriptome data.

Major comments:

1. Although the authors assess the genome completeness with the BUSCO test, a single BUSCO percentage value is not informative when considering the concept of an orthologs finding strategy (i.e. a comparative approach, reference points are needed). To better show the genome completeness, the authors are encouraged to perform the BUSCO test on all close-related available mollusc genomes.
Thanks for your suggestion, we assayed the genome completeness using Benchmark Universal Single-Copy Orthologs BUSCO v4.1.4 referencing metazoan and molluscan gene sets. In the metazoan dataset, the current assemblies have 89.4% complete (of which 1.0% were duplicated), 1.9% incomplete and 8.7% missing BUSCOs, corresponding to a recovery of 91.3% of the entire BUSCO set. In the molluscan dataset, 85.5% complete (of which 1.3% were duplicated), 0.8% incomplete and 13.7% missing BUSCOs were recorded, corresponding to 86.3% of the entire BUSCO set (Line 352-361).
In addition, we performed the BUSCO tests using these close-related available bivalve genomes (see the following table) to show the recovery (Complete + imcomplete) of the entire BUSCO set,
Species Metazoa Mollusca
Pinctada fucata martensii 90.1% 84.0%
Pecten maximus 96.5% 95.9%
Mytilus coruscus 91.3% 86.3%
Mytilus coruscus previous version 94.5% 88.7%
Modiolus philippinarum 90.1% 84.0%
Bathymodiolus platifrons 93.7% 90.1%
Venustaconcha ellipsiformis 74.5% 54.9%

2. Figure 4a: Using Circos to show genome-wide SNPs and indels between farmed and wild populations doesn't seem informative. I don't know what the readers should expect to see from this panel. If there is

no information, then consider removing it from the main figure. Instead, the authors should show a few specific examples, such as the SNP differences at the locus of chitobiase mentioned in the main text. Only listing KEGG or GO terms such as "genetic information processing", "metabolism", and "signaling and cellular processes" is too general and provides no useful information to the readers.
Thanks for your suggestions, we have put the Circos in supplementary Figures and provided the specific example of SNP differences at the locus of chitobiase in the main text and Figure 4b. The speculation of functions have been removed in the revision, because the evidence is absent. We re-write this section and weaken the claims made from it in the abstract and conclusion since this is just a preliminary try in the genome study.

3. Since the genome of the mussel Mytilus coruscus has been previously published, the main point of this paper seems to be their chromosome-level assembly. However, the advantage of having a chromosome-level genome in this manuscript is not apparently demonstrated. And the analysis of Figure 5 is not clear, especially for Figure 5e. The authors are encouraged to pay more attention to this part and present better data to demonstrate the benefit of having a chromosome-level assembly.
Thanks for your suggestions, we re-edit the Figure 5 by adding the subtitiles for the chromosome synteny of P. maximus vs S. broughtonii, P. maximus vs M. coruscus, P. maximus vs P. fucata, and P. maximus vs C. gigas and the dashed lines to indicate the corresponding evolution relationship (Fig. 5e).

4. Figure 6: I understand that the authors tried to use KEGG annotation to make sense of their RNA-seq data, but do mussels have cardiomyocytes? If not, how can a cardiomyocyte pathway be directly applied to a set of mussel genes? For example, actin and myosin are ubiquitous genes as cytoskeleton or component of muscle fibers. What is the rationale to link authors' assumption by just looking at these general gene expressions? Similar to this line, other signaling genes, such as NF-κB and many other protein kinases, also play roles in many different pathways. I do not think that the authors can conclude anything from randomly selecting a set of genes in the cell type that are not existing in the species they analyzed.
Most of the KEGG pathways are constructed by the model animals or plants, not by the mussels. So we focus the pathways that have been reported to be related to metamorphosis in mussel. We analyzed the up-regulated genes during the period from umbo to pediveliger, of which 26 genes are involved in “adrenergic signaling in cardiomyocytes”, “calcium signaling pathway”, “MAPK signaling pathway”, “protein export”, “endocytosis” and “catecholamine biosynthesis” pathways. These pathways are reported to be involved in settlement and metamorphosis [18, 66]. Most of the involved genes are functionally identified to be associated with metamorphosis development (Supplementary Table S5). Selection of these genes are based on their function information, not from a random selection. Most of our observations are consistent with exist study of metamorphosis development. Noticeably, mussels have cardiomyocyte, like most of mollusca species (watts et al, 1981; Kodirov 2011). The recent proteome analysis (Di et al. 2020) and ISH (Yang et al. 2012) identify that the "adrenergic signaling in cardiomyocytes" pathway is functional during metamorphosis of oyster, reflecting its importance in regulation of metamorphosis.
This transcriptome analyses of larva tissues provide a preliminary try to take advantage of current reference genome to investigate the metamorphosis development. Hence, we weaken the speculating claims in the revision, such as discarding the previous hypothesis that signal transduction controlling the metamorphosis development seemed to activate during the first two stages. The instructive suggestion is raised in the end of the section, instead.
Reference:
Watts, J.A., Koch, R.A., Greenberg, M.J. and Pierce, S.K. (1981), Ultrastructure of the heart of the marine mussel, Geukensia demissa. J. Morphol., 170: 301-319.
Kodirov, S. A. (2011). The neuronal control of cardiac functions in Molluscs. Comparative Biochemistry and Physiology Part A: Molecular & Integrative Physiology, 160(2), 102-116.
Di, G., Xiao, X., Tong, M.H. et al. (2020), Proteome of larval metamorphosis induced by epinephrine in the Fujian oyster Crassostrea angulata. BMC Genomics 21, 675.
Yang, B., Qin, J., Shi, B., Han, G., Chen, J., Huang, H., and Ke, C. (2012). Molecular characterization and functional analysis of adrenergic like receptor during larval metamorphosis in Crassostrea angulata. Aquaculture 366-367, 54-61.

5: Furthermore, the heatmap is also not informative. Do these genes differentially expressed at a particular stage? What is the statistical method that the authors use to evaluate differentially expressed genes? With their RNA-seq analysis, the authors expose their weakness in the developmental process of mussels. The whole study is confusing and inconclusive.
A supplementary table corresponding to the heatmap (Fig.6) is added in the revision, which lists the detailed description of gene functions and the related references. Most of the DEGs in the heatmap are

differentially expressed during at least one stage. Quantified gene expression levels are normalized to the TPM values in the revision. This Limma statistical methodologise are suitable to detect differentially expressed genes based on linear models (Smyth et al. 2005). To ensure that the claims are proportionate to the evidence presented, we moderate the conclusion by constructive suggestions instead of the strong claims in the revision

Reference:

Smyth GK, Ritchie M, Thorne N, et al. LIMMA: linear models for microarray data. In Bioinformatics and Computational Biology Solutions Using R and Bioconductor. Statistics for Biology and Health. 2005.

Close
