# Supplementary material for: Chromosome-level genome assembly of the hard-shelled mussel Mytilus coruscus, a widely distributed species from the temperate areas of East Asia
Source: Gigascience. 2021 Apr 23;10(4):giab024. doi: 10.1093/gigascience/giab024 (PMC8063583; doi:10.1093/gigascience/giab024)
Supplement: giab024_Reviewer_1_Report_Original_Submission — Marco Gerdol -- 10/19/2020 Reviewed [file giab024_reviewer_1_report_original_submission.pdf]

Reviewer Report

Title: Chromosome-level genome assembly of the hard-shelled mussel Mytilus coruscus, a widely distributed species from the temperate areas of East Asia

Version: Original Submission **Date:** 10/19/2020

Reviewer name: Marco Gerdol

Reviewer Comments to Author:

The manuscript by Yang and colleagues reports a high quality genome assembly for the mussel Mytilus coruscus. Although this is not the first genome assembly published for this species, this resource is an improvement compared with the previous version, due to the use of Hi-C libraries and a better management of heterozygous genomic regions. Hence, the contents of this work appear to be appropriate for a data note article. there are however several points that would require some additional information to be added, and bits of text that need to be modified to improve the flow of the text.
I will first provide a few general comments. A few detailed notes can be found attached below.
I would suggest the authors to specify the sequencing coverage achieved somewhere in the text (i.e. which coverage was obtained with ONT reads? Which coverage was obtained with Illumina PE? Etc.). This is present in Table 1, but it should be also mentioned in the text.
The authors emphasized the high heterozygosity of the genome, pointing out the possible links between SNPs and phenotypic variation. The authors may not be aware of the very recent discoveries that currently indicate that bivalve genomes are characterized by significant hemizygosity and structural variants that affect gene content, resulting in massive gene presence/absence variation. While the authors are not currently required to update this work with a detailed analysis of PAV, I think the text might benefit from some additional points of discussion, especially considering the fact that a congeneric mussel species, M. galloprovincialis, has been shown to be characterized by an astounding level of intraspecific genomic variation (see the preprint by Gerdol et al. 2019, which has recently been accepted for publication and should become available online in the matter of a few weeks on genome Biology). Also see the preprint by Calcino and colleagues here:
https://www.biorxiv.org/content/10.1101/2020.09.15.298695v1
I general, I would recommend the authors to involve a native English-speaking colleague in the revision of the text, as several grammar errors and oddly constructed sentences are present throughout the text.
Abstract
-correct "high-through"; I guess the authors were referring to "high throughput" here.
-Correct "platifron" with "paltifrons"
-"speculating their sharing same origins in evolution" please correct this odd wording.
List of detailed comments
-Mussels have been also used as sentinel organisms for biomonitorning, and this information could be added to the list
-"As with a dozen of marine invertebrates". This is unclear; I guess the authors meant "As several other marine invertebrates"

-When talking about the M. galloprovincialis genome assembly, the authors only refer to the paper by Murgarella and colleagues, whereas an improved version has been recently accepted for publication on Genome Biology (this should be probably available online within a few weeks). The text is available as a preprint, see Gerdol et al. https://www.biorxiv.org/content/10.1101/781377v1

Methods

-This section in particular suffers from the presence of several issues with the quality of the language used, that should be improved.

-When talking about the k-mer graph, please refer to the homozygous and heterozygous peaks (instead of "junior peak"

-"very close to the total assemblies (1.57 Gb)". I think it would be worth mentioning that this is also not far from the c-value previously estimated by cytogenetic studies (see Ieyama, H., O. Kameoka, T. Tan, and J. Yamasaki (1994). Chromosomes and nuclear DNA contents of some species of Mytilidae. Venus 53: 327-331)

-"which is much greater than the real size of 1.57 Gb". This is also in line with what has been observed for M. galloprovincialis by Gerdol et al.

-"The yielded consensus sequences were manually checked by aligning to the GenBank database". This is a clever strategy, but I think it should be explained a bit better here.

-"which was less than previously-published 42,684 gene models in the draft genome because it introduced over 20% heterozygous redundances in the assemblies". I agree with this consideration, but in light woth the recent findings about widespread hemizygosity and massive gene presence/absence variation in M. galloprovincialis, the authors might want to update the text with a few additional considerations.

-"448 single-copy genes". How were such genes identified? Was BUSCO/OrthoDB used for this?

-"Whole genome re-sequencing of farmed and wild individuals". The data provided here are potentially interesting for a preliminary analysis, but the authors should keep in mind (and briefly discuss) the possibility that higher-order structural variants which include gene PAV might have a very important role on phenotypic traits.

-"consistent with their closest phylogenetic relationship in the Bivalvia clade" please add a reference for this.

-It would have been more appropriate to use TPM instead of FPKM, as this metric allows a more reliable comparison among samples.

-"indicative of a 91.9% genome completeness when 89.98% of core metazoan orthologswere completely identified in the assemblies." This is somewhat unclear. Does 91.9% indicate present BUSCOs and 89.98% "present and complete"? Adding specific information concerning fragmented BUSCOs and duplicated or missing BUSCOs would help here.

Level of Interest

Please indicate how interesting you found the manuscript: Choose an item.

Quality of Written English

Please indicate the quality of language in the manuscript: Choose an item.

Declaration of Competing Interests

Please complete a declaration of competing interests, considering the following questions:

- Have you in the past five years received reimbursements, fees, funding, or salary from an organisation that may in any way gain or lose financially from the publication of this manuscript, either now or in the future?
- Do you hold any stocks or shares in an organisation that may in any way gain or lose financially from the publication of this manuscript, either now or in the future?
- Do you hold or are you currently applying for any patents relating to the content of the manuscript?
- Have you received reimbursements, fees, funding, or salary from an organization that holds or has applied for patents relating to the content of the manuscript?
- Do you have any other financial competing interests?
- Do you have any non-financial competing interests in relation to this paper?

If you can answer no to all of the above, write 'I declare that I have no competing interests' below. If your reply is yes to any, please give details below.

I declare that I have no competing interests

I agree to the open peer review policy of the journal. I understand that my name will be included on my report to the authors and, if the manuscript is accepted for publication, my named report including any attachments I upload will be posted on the website along with the authors' responses. I agree for my report to be made available under an Open Access Creative Commons CC-BY license (http://creativecommons.org/licenses/by/4.0/). I understand that any comments which I do not wish to be included in my named report can be included as confidential comments to the editors, which will not be published.

Choose an item.

To further support our reviewers, we have joined with Publons, where you can gain additional credit to further highlight your hard work (see: https://publons.com/journal/530/gigascience). On publication of this paper, your review will be automatically added to Publons, you can then choose whether or not to claim your Publons credit. I understand this statement.

Yes Choose an item.
