# Supplementary material for: Chromosome-level genome assembly of the hard-shelled mussel Mytilus coruscus, a widely distributed species from the temperate areas of East Asia
Source: Gigascience. 2021 Apr 23;10(4):giab024. doi: 10.1093/gigascience/giab024 (PMC8063583; doi:10.1093/gigascience/giab024)
Supplement: giab024_Reviewer_2_Report_Original_Submission — Nathan Kenny -- 10/24/2020 Reviewed [file giab024_reviewer_2_report_original_submission.pdf]

Reviewer Report

Title: Chromosome-level genome assembly of the hard-shelled mussel Mytilus coruscus, a widely distributed species from the temperate areas of East Asia

Version: Original Submission **Date:** 10/24/2020

Reviewer name: Nathan Kenny

Reviewer Comments to Author:

Yang et al present the genome assembly of the hard-shelled mussel Mytilus coruscus, alongside gene predictions and analysis of genome content. The work in assembling the genome and predicting genes is technically sound. However, the presentation of this work is inprecise, not yet of publishable standard, and would benefit from careful editing for science and language before re-submission. There are also several scientific points that need to be addressed, to ensure that the claims made in the manuscript are proportionate to the evidence presented. I have noted these below.

Major points to address:

1) The authors claim that their genome represents a chromosome-level assembly of the genome of this species. This claim is based on the combination of reasonably long contigs into scaffolds using Lachesis based on linkage. To be able to firmly claim that these represent a "chromosome level assembly" it is necessary to evaluate the degree to which these pseudomolecules are assembled. Table 2 should provide data on the exteent of gaps (total Ns) in each chromosome, and in the text, the size distribution of gaps, and information about them, should be noted. Are these, for instance, estimated and set at 100/1000 Ns? or are these a true reflection of the gap size? Is there any evidence of telomeric sequence at each end?

2) There is a stark difference in estimated genome size between the previously published genome for this species and this resource. It would be useful to map the previous (draft) assembly of Li et al to this assembly and determine what percentage of the huge missing fragment (21%) of that assembly is truly missing from this assembly, and why. Does this represent uncollapsed heterozygosity (which would map twice to the same loci, presumably), intraspecific hemizygosity variation, or contamination in the previous genome resource? Or is it perhaps a problem of missing data in the assembly presented here? Any of these answers would be useful for understanding the genome of this species.

3) Genome size estimation is carried out by a mathematical derivation directly from the highest peak size. This, however, partially excludes from consideration the heterozygous portion of the genome. As the assembly has been polished with Racon and Pilon, heterozygosity could also be underestimated by mapping estimates. It is recommended that alternate genome size estimates are provided. Genomescope (http://qb.cshl.edu/genomescope/genomescope2.0/) is a simple-to-use option that will provide more nuanced information regarding genome size and heterozygosity.

4) how many of the gene models found in Li et al are present/absent from the final gene set presented here? Were these used in the EVidenceModeler merge step? It is noted that "37,478 final gene models were generated (Table 3), which was less than previously-published 42,684 gene models in the draft genome because it introduced over 20% heterozygous redundances in the assemblies". Please provide

more information on how this was determined, as these extra genes could also represent recent duplicates, which should not be removed from consideration. This could build upon the results of 2) above.
5) The phylogeny as presented needs further consideration.
-Was concatenation of genes performed before alignment? (page 11) This could introduce errors at the start and end of each gene as they can artifactually be aligned to non-homologous sequences. This should be checked, and repeated correctly if necessary (with alignment performed gene-by-gene, then concatenating the alignments).
-What maximum likelihood model was used? what other settings? how many bootstraps? Please note in text (page 11).
-How was divergence time calculated?
6) In the "Whole genome re-sequencing of farmed and wild individuals" section, the assumption that sequence variations are farmed- population-specific (FPS) or wild-population-specific (WPS) is flawed as it is based on a tiny sample (20 individuals) of the enormous diversity of this species. It is not convincing to claim that these variants are unique to either farmed populations or wild populations - they are just observed to be different here due to the limited sampling. The depth of sequencing is also very low per individual (around 2.5x) and SNPs/indels could be missed. This section, and the claims made from it in the abstract and conclusions, need to be substantially reworked to avoid drawing universal conclusions from what are only initial pilot results.
7) The differential expression analysis in larvae is not convincing. Many of the genes cherry-picked for discussion and shown in Fig 6 are expressed in all samples. As only single libraries were sequenced for each larval life stage, claims for differential expression are only very weakly supported. It is good practice to use a minimum of 3 separate samples per condition for DE analysis, and preferentially more. The authors should moderate the strength of the conclusions drawn in the "Transcriptome related to metamorphosis" section considerably, in light of the strength of some of the evidence presented.
Minor points:
-The authors are often too strong in their criticism of the earlier genomes for this species and Mytilus. For instance "a low quality draft genome of M. coruscus has been reported" (pg 4). That resource is not as well-contiged, but saying it is low quality is not justified. Perhaps "Draft versions of the genomes of M. coruscus and M. galloprovincialis have been reported". This kind of strong claim should be toned down throughout the manuscript.
- Many of the steps shown in Fig 2 (e.g. read cleaning) are not covered in sufficient detail in the manuscript. Please ensure that the steps required to recapitulate this work are provided.
-What settings were used for OrthoMCL?
-What settings were used to detect PCR duplicates with Picard?
Fig 3d : caniculata seems to be mis-spelled
Fig 5: Why is P. fucata highlighted? Why not show P. maximus vs Mytilus coruscus? It is the most relevant for this paper. Fig 5a and Fig 5b might be the wrong images?
Note on language and scientific accuracy:
Throughout the manuscript there are minor errors in written english, which regularly introduce scientifically inaccurate statements. I have noted some of these below but my list is not complete, and the authors may wish to have their manuscript read over more thoroughly before resubmission. I have

not had the time to correct all the errors present in the manuscript.
Throughout: Please refer to the species name or the common name, but not "marine mussel" when you mean Mytilus coruscus - most mussels are marine. Similarly, do not use this to refer to all mussels
Title: the authors should consider introducing a comma into their title, breaking it into precise units: e.g. "A chromosome-level genome assembly of the hard-shelled mussel Mytilus coruscus, a widely distributed species from temperate areas of East Asia"
Abstract:
-no "A" in : A chromosome-level genome information
-high-through - do you mean high-throughput?
-" The completeness test exhibits" - I think you mean "comparison to the CEGMA metazoan complement reveals"
-No "The" in "The phylogenetic analysis"
-"the closest relationship between" - this is not true. I think you mean "phylogenetic analysis shows M. coruscus is the sister taxon to the clade comprised of Modiolus philippinarum and Bathymodiolus platifrons". Note spelling of last species
-No "A", in "A conserved chromosome synteny "
-"speculating their sharing same origins in evolution" do you mean "suggesting that this is shared ancestrally"? Because the former is contentious
-no on in "studying on"
Context:
-phylum Mollusca (not Mollusc).
-"sea mussels". This is an inprecise phrase. Perhaps just use "mussels"
- " Although their significance" - should read " Although they are significant for biology, ecology and the economy"
- need an "and" before ", settlement mechanism."
-"As with a dozen of marine invertebrates" - this is a deeply inaccurate statement. Perhaps "As with many marine invertebrates".
-"modeling of their anatomy " not "modeling of anatomy "
-"trigger settlement and metamorphosis is universal in metazoan" - this is not true. Humans, for instance, are metazoans
- "temperate areas" not "the temperate"
-"need adapt..." should read "needs to adapt to the hostile..."
-"Up to date, chromosome level genome" should read "To date, a chromosomal-level genome"
-"Lacking whole-genome information" should read "The lack of whole-genome information".
-"The larvaes at five ..." should read "Larvae at five....". "gene expression" not "gene expressions"
Methods:
-"where is the central coast of Chinese mainland" should read "the central coast of the Chinese mainland"
- "a" needed, A female wild adult with a mature ovary (although these are probably paired but difficult to detect - if paired this would be "with mature ovaries".)
-" for the adductor muscle to isolate high molecular weight genomic DNA for sequencing of reference genome" should read ", with the adductor muscle taken for isolation of high molecular weight genomic

DNA, for sequencing of the reference genome".
- no s "The DNAs"
-" to be assistant " should read "to assist with"
-" using SDS extraction method," should read " using the SDS extraction method," and a reference to this protocol should be given.
-"total RNA were extracted" should read "total RNA was extracted"
-"as well as the larvaes" should read "as well as larvae"
"to get large segments " should read "to extract large fragments"
-fragments should be used instead of segments throughout this section.
". The high quality library of average 20 kb in length was sequenced on the ONT PromethION platform with corresponding R9 cell and ONT sequencing reagents kit. The genomic DNA was sequenced using the MinION portable DNA sequencer with the 48 hours run script (Oxford Nanopore), which generated a total of 246.8 Gb data" - were both the minion and promethion used? please make this clearer.
-" were fragmentized" should read " were fragmented"
-novaseq needs a capital
- "by poly(A)" should read "for poly(A) transcripts". Which protocol was used?
-" in 150 bp paired-end model." should read " in 150 bp paired-end mode."
-"Genome size of the hard-shelled " needs a "The" before
-" Average GC content of genome" needs a the before genome.
-"The final assemblies is around 1.57 Gb" should be "The final assembly is around 1.57 Gb"
-"The genome assemblies of hard-shelled mussel" again should be assembly
-"with the softwares of Augustus (version 3.1) [38], GlimmerHMM (version 1.2) [39] and SNAP (version 2006-07-28) " should read "with Augustus (version 3.1) [38], GlimmerHMM (version 1.2) [39] and SNAP (version 2006-07-28) software"
-"protein sequences of two closed mollusc species" do you mean two closely related mollusc species?
-"Parallelly," should be "In parallel"
-"put into a de novo assemble" should be "assembled de novo"
-transnfer mis-spelled, Pg 9 (= transfer)
-"The gene clusters were identified among 12 selected genome" should be "Gene clusters were identified among 12 selected genomes"
-"reflected the closest relationship between M. coruscus and the clade of M. philippinarum and B. platifrons,". This is oddly stated. I think you mean "M. coruscus was found to be the sister taxon to the clade containing M. philippinarum and B. platifrons". Also, how was the divergence time calculated?
- s needed, " in farmed and wild sample, respectively" should be " in farmed and wild samples, respectively"
-"while 5,719,771 and 1,820,404 in wild one" should read "and 5,719,771 and 1,820,404 in wild populations"
-"The chromosome synteny illustrated that rare large-scale rearrangements between scallop and mussel, but frequent between scallop and oysters" should be rewritten "Chromosome synteny illustrates that large-scale rearrangements are rare between scallop and mussel, but more frequent between scallop and oysters"
-No s "almost all of the chromosomes rearrangements " - should be "almost all of the chromosome

rearrangements "
-"To profile the gene expressions" should be "To profile gene expression"
-"Quality of the assembled genome " should read "The quality of the assembled genome.... "
-"in genome assemble" should read "in the genome assembly"
-"facilitate a wide range of researches in mussel, bivalve, and molluscan." needs another word after molluscan - molluscan biology, maybe?
-"evolution in bivalve" should be "evolution in bivalves"
-"As one of the best-assembled bivalve genomes" - this is too strong a claim given the evidence presented.
Please note there are numerous additional language problems to correct, and this is beyond the scope of my review. I suggest a careful re-reading of the manuscript before resubmission.

Level of Interest

Please indicate how interesting you found the manuscript: Choose an item.

Quality of Written English

Please indicate the quality of language in the manuscript: Choose an item.

Declaration of Competing Interests

Please complete a declaration of competing interests, considering the following questions:

- Have you in the past five years received reimbursements, fees, funding, or salary from an organisation that may in any way gain or lose financially from the publication of this manuscript, either now or in the future?
- Do you hold any stocks or shares in an organisation that may in any way gain or lose financially from the publication of this manuscript, either now or in the future?
- Do you hold or are you currently applying for any patents relating to the content of the manuscript?
- Have you received reimbursements, fees, funding, or salary from an organization that holds or has applied for patents relating to the content of the manuscript?
- Do you have any other financial competing interests?
- Do you have any non-financial competing interests in relation to this paper?

If you can answer no to all of the above, write 'I declare that I have no competing interests' below. If your reply is yes to any, please give details below.

I declare that I have no competing interests

I agree to the open peer review policy of the journal. I understand that my name will be included on my report to the authors and, if the manuscript is accepted for publication, my named report including any attachments I upload will be posted on the website along with the authors' responses. I agree for my report to be made available under an Open Access Creative Commons CC-BY license (http://creativecommons.org/licenses/by/4.0/). I understand that any comments which I do not wish to be included in my named report can be included as confidential comments to the editors, which will not be published.

Choose an item.

To further support our reviewers, we have joined with Publons, where you can gain additional credit to further highlight your hard work (see: https://publons.com/journal/530/gigascience). On publication of this paper, your review will be automatically added to Publons, you can then choose whether or not to claim your Publons credit. I understand this statement.

Yes Choose an item.
