# Supplementary material for: Chromosome-level genome assembly of the hard-shelled mussel Mytilus coruscus, a widely distributed species from the temperate areas of East Asia
Source: Gigascience. 2021 Apr 23;10(4):giab024. doi: 10.1093/gigascience/giab024 (PMC8063583; doi:10.1093/gigascience/giab024)
Supplement: giab024_Reviewer_2_Report_Revision_1 — Nathan Kenny -- 1/30/2021 Reviewed [file giab024_reviewer_2_report_revision_1.pdf]

Reviewer Report

Title: Chromosome-level genome assembly of the hard-shelled mussel Mytilus coruscus, a widely distributed species from the temperate areas of East Asia

Version: Revision 1 **Date:** 1/30/2021

Reviewer name: Nathan Kenny

Reviewer Comments to Author:

The response by Yang et al has mostly addressed my concerns. However, I have a few remaining points to address, which should be corrected before acceptance.
1) the main text tables have not been provided, and I am unable to assess their contents. I do not need to see these again provided the editor can confirm their veracity.
in answer to my major point 1) regarding N content as I cannot see if this information is provided in the table, please ensure that it is noted either in the main text or in the table legend that gaps are preset at 100Ns, and may be longer or shorter. It may be useful to give a "total number of gaps" figure in this table.
2) for Figure 5 it is still confusing why M. coruscus is not compared to all the other genomes, rather than doing these comparisons with another species (which has been studied previously). Would you like to show more M. coruscus results?
3) in Fig 6b, please indicate in the figure legend whether these results are the averages of your 3 replicate samples, or individual samples
Minor comments: The manuscript would still benefit from another round of proof reading. I have noted some problems below.
Abstract:
-Linkage misspelled, "14 linage groups"
-carried is the wrong word, and the past tense is incorrect here: "carried 91.9% of core metazoan orthologs." Perhaps "possesses 91.9% of the core metazoan ortholog set"
- "the sister taxon" not "a sister taxon" (also in methods section)
Introduction:
- "due to their high economic" not "due to the high"
-"understanding of the larvae-juvenile" not "understanding of larvae-juvenile"
-"widespread among metazoans" not "widespread among metazoan "
-"Most studies" not "Most of studies"
-"hard-shelled mussel genetic breeding" not "the hard-shelled mussel genetic breeding" (no the)
- I suggest "molluscs" rather than "mollusks" in the last paragraph, to match your usage elsewhere
Methods:
- no "a"s in "at a 57Ã— coverage and the heterozygous peak was at a 28Ã—" - this should read "at 57Ã— coverage and the heterozygous peak was at 28Ã—"
-"usually occurs in fragmented assemblies" not "usually occurs in fragmented assembly",
-"align to one or more of the InterPro " not "have the alignment to one or more of the InterPro"

-"Chromosome-level genomes allow re-sequencing and population genetic studies." not "Chromosome-level genome is important for re-sequencing and population genetic."
-"preliminary assay" not "preliminary try "
-"Benchmark Universal Single-Copy Orthologs (BUSCO) v4.1.4" not "Benchmark Universal Single-Copy Orthologs BUSCO v4.1.4"
Abbreviations"
no "the", TPM: Transcripts per Million;
Figure 3 legend (and possibly elsewhere) please italicise the k in kmer
Figure 4 legend: gene names need italics
Fig 5 legend: "corresponding evolutionary relationship" not "corresponding evolution relationship"
-some bold font needed, Additional Files title page (pg 25)

Level of Interest

Please indicate how interesting you found the manuscript: Choose an item.

Quality of Written English

Please indicate the quality of language in the manuscript: Choose an item.

Declaration of Competing Interests

Please complete a declaration of competing interests, considering the following questions:

- Have you in the past five years received reimbursements, fees, funding, or salary from an organisation that may in any way gain or lose financially from the publication of this manuscript, either now or in the future?
- Do you hold any stocks or shares in an organisation that may in any way gain or lose financially from the publication of this manuscript, either now or in the future?
- Do you hold or are you currently applying for any patents relating to the content of the manuscript?
- Have you received reimbursements, fees, funding, or salary from an organization that holds or has applied for patents relating to the content of the manuscript?
- Do you have any other financial competing interests?
- Do you have any non-financial competing interests in relation to this paper?

If you can answer no to all of the above, write 'I declare that I have no competing interests' below. If your reply is yes to any, please give details below.

I declare that I have no competing interests

I agree to the open peer review policy of the journal. I understand that my name will be included on my report to the authors and, if the manuscript is accepted for publication, my named report including any attachments I upload will be posted on the website along with the authors' responses. I agree for my report to be made available under an Open Access Creative Commons CC-BY license (http://creativecommons.org/licenses/by/4.0/). I understand that any comments which I do not wish to be included in my named report can be included as confidential comments to the editors, which will not be published.

Choose an item.

To further support our reviewers, we have joined with Publons, where you can gain additional credit to further highlight your hard work (see: https://publons.com/journal/530/gigascience). On publication of this paper, your review will be automatically added to Publons, you can then choose whether or not to claim your Publons credit. I understand this statement.

Yes Choose an item.
