# Supplementary material for: Chromosome-level genome assembly of the hard-shelled mussel Mytilus coruscus, a widely distributed species from the temperate areas of East Asia
Source: Gigascience. 2021 Apr 23;10(4):giab024. doi: 10.1093/gigascience/giab024 (PMC8063583; doi:10.1093/gigascience/giab024)
Supplement: giab024_Reviewer_3_Report_Original_Submission — Yi-Jyun Luo -- 10/26/2020 Reviewed [file giab024_reviewer_3_report_original_submission.pdf]

Reviewer Report

Title: Chromosome-level genome assembly of the hard-shelled mussel Mytilus coruscus, a widely distributed species from the temperate areas of East Asia

Version: Original Submission **Date:** 10/26/2020

Reviewer name: Yi-Jyun Luo

Reviewer Comments to Author:

This study presented a high-quality genome of the mussel Mytilus coruscus. Using a mixed strategy to combine Illumina short reads and Nanopore long reads followed by scaffolding with Hi-C, the authors generated a chromosomal-level genome assembly. They further re-sequenced farmed and wild individuals to detect SNP and indel differences among the two populations. The authors then focused on the pathways related to larval settlement and metamorphosis using RNA-seq analysis. Overall, the genome quality looks good, but I have a few questions on how the authors analyzed and interpreted genome and transcriptome data.

Major comments:

1. Although the authors assess the genome completeness with the BUSCO test, a single BUSCO percentage value is not informative when considering the concept of an orthologs finding strategy (i.e. a comparative approach, reference points are needed). To better show the genome completeness, the authors are encouraged to perform the BUSCO test on all close-related available mollusc genomes.
2. Figure 4a: Using Circos to show genome-wide SNPs and indels between farmed and wild populations doesn't seem informative. I don't know what the readers should expect to see from this panel. If there is no information, then consider removing it from the main figure. Instead, the authors should show a few specific examples, such as the SNP differences at the locus of chitobiase mentioned in the main text. Only listing KEGG or GO terms such as "genetic information processing", "metabolism", and "signaling and cellular processes" is too general and provides no useful information to the readers.
3. Since the genome of the mussel Mytilus coruscus has been previously published, the main point of this paper seems to be their chromosome-level assembly. However, the advantage of having a chromosome-level genome in this manuscript is not apparently demonstrated. And the analysis of Figure 5 is not clear, especially for Figure 5e. The authors are encouraged to pay more attention to this part and present better data to demonstrate the benefit of having a chromosome-level assembly.
4. Figure 6: I understand that the authors tried to use KEGG annotation to make sense of their RNA-seq data, but do mussels have cardiomyocytes? If not, how can a cardiomyocyte pathway be directly applied to a set of mussel genes? For example, actin and myosin are ubiquitous genes as cytoskeleton or component of muscle fibers. What is the rationale to link authors' assumption by just looking at these general gene expressions? Similar to this line, other signaling genes, such as NF-ÎºB and many other protein kinases, also play roles in many different pathways. I do not think that the authors can conclude anything from randomly selecting a set of genes in the cell type that are not existing in the species they analyzed.

Furthermore, the heatmap is also not informative. Do these genes differentially expressed at a particular

stage? What is the statistical method that the authors use to evaluate differentially expressed genes? With their RNA-seq analysis, the authors expose their weakness in the developmental process of mussels. The whole study is confusing and inconclusive.

Level of Interest

Please indicate how interesting you found the manuscript: Choose an item.

Quality of Written English

Please indicate the quality of language in the manuscript: Choose an item.

Declaration of Competing Interests

Please complete a declaration of competing interests, considering the following questions:

- Have you in the past five years received reimbursements, fees, funding, or salary from an organisation that may in any way gain or lose financially from the publication of this manuscript, either now or in the future?
- Do you hold any stocks or shares in an organisation that may in any way gain or lose financially from the publication of this manuscript, either now or in the future?
- Do you hold or are you currently applying for any patents relating to the content of the manuscript?
- Have you received reimbursements, fees, funding, or salary from an organization that holds or has applied for patents relating to the content of the manuscript?
- Do you have any other financial competing interests?
- Do you have any non-financial competing interests in relation to this paper?

If you can answer no to all of the above, write 'I declare that I have no competing interests' below. If your reply is yes to any, please give details below.

I declare that I have no competing interests.

I agree to the open peer review policy of the journal. I understand that my name will be included on my report to the authors and, if the manuscript is accepted for publication, my named report including any attachments I upload will be posted on the website along with the authors' responses. I agree for my report to be made available under an Open Access Creative Commons CC-BY license (http://creativecommons.org/licenses/by/4.0/). I understand that any comments which I do not wish to be included in my named report can be included as confidential comments to the editors, which will not be published.

Choose an item.

To further support our reviewers, we have joined with Publons, where you can gain additional credit to further highlight your hard work (see: https://publons.com/journal/530/gigascience). On publication of this paper, your review will be automatically added to Publons, you can then choose whether or not to claim your Publons credit. I understand this statement.

Yes Choose an item.
