# Supplementary material for: Chromosome-level genome assembly of the hard-shelled mussel Mytilus coruscus, a widely distributed species from the temperate areas of East Asia
Source: Gigascience. 2021 Apr 23;10(4):giab024. doi: 10.1093/gigascience/giab024 (PMC8063583; doi:10.1093/gigascience/giab024)
Supplement: giab024_Supplemental_Files [file giab024_supplemental_files.zip › GIGA-D-20-00287R2-point-to-point_Responses22.doc]

GIGA-D-20-00287R2.

Dear Dr. Hans Zauner

Giga Science

Thank you so much for your time and valuable help in improving the manuscript. Please find below our detailed responses addressing the comments (answers in blue).

Reviewer Comments:

**Reviewer** **#2:**

*The response by Yang et al has mostly addressed my concerns. However, I have a few remaining points to address, which should be corrected before acceptance.*

*Major points to address:*

*1) the main text tables have not been provided, and I am unable to assess their contents. I do not need to see these again provided the editor can confirm their veracity.*

*in answer to my major point 1) regarding N content as I cannot see if this information is provided in the table, please ensure that it is noted either in the main text or in the table legend that gaps are preset at 100Ns, and may be longer or shorter. It may be useful to give a "total number of gaps" figure in this table.*

Yes, total numbers of gaps are listed in Table 2 of the revision, which describes that each gap is preset at 100 Ns (Line 675-676).

*2) for Figure 5 it is still confusing why M. coruscus is not compared to all the other genomes, rather than doing these comparisons with another species (which has been studied previously). Would you like to show more M. coruscus results?*

Thanks for your suggestion. We construct the synteny between *M. coruscus* and four reported chromosome-level genomes in the revision (Fig. 5). In the main text (Fig.5), *P. maximus* is selected as a reference to illustrate gene collinearity (P. maximus vs another 4 species, including *M. coruscus*) due to its slow evolution. The synteny between the hard-shelled mussel and another three species (Pacific oyster, blood clam, pearl oyster) is added in the Fig. 5g-h of the revision (mentioned in main text Line 309-312, Line 475-478) as the follows:

In addition, the high gene collinearity between the hard-shelled mussel and another three bivalves of the Pacific oyster, blood clam and pearl oyster also reflected the satisfied quality of the hard-shelled mussel assemblies (Fig. 5f-h).

**Figure 5.** Chromosome synteny. **a**. Alignment of king scallop and blood clam chromosomes. **b**. Alignment of king scallop and hard-shelled mussel chromosomes. **c**. Alignment of king scallop and pearl oyster chromosomes. **d**. Alignment of king scallop and Pacific oyster chromosomes. **e**. Rearrangements between the chromosomes of king scallop and those of four other bivalve species. The king scallop chromosomes are represented by bars of different colors, and synteny and rearrangements in the chromosomes of the four other bivalves are indicated by different blocks, whose colors correspond to those of the reference king scallop chromosomes, the dashed lines indicate the corresponding evolutionary relationship. **f**. Alignment of hard-shelled mussel and blood clam chromosomes. **g**. Alignment of hard-shelled mussel and pearl oyster chromosomes. **h**. Alignment of hard-shelled mussel and Pacific oyster chromosomes. The king scallop linkage groups are labeled as PM 1 to 19, the blood clam chromosomes as SB 1 to 19, the hard-shelled mussel chromosomes as MC 1 to 14, the pearl oyster chromosomes as PF 1 to 14, and the Pacific oyster chromosomes as CG 1 to 10. Scale unit, Mb. **a–d**, **f-h**. The circularized blocks represent the chromosomes of the five bivalves. Aligned homologous genes are connected by ribbons, shown in different colors depending on their chromosome location.

*3) in Fig 6b, please indicate in the figure legend whether these results are the averages of your 3 replicate samples, or individual samples*

Thanks for your suggestion. We describe the information in the legend of the Fig. 6b that these quantification results of gene expression are the averages of three replicate samples. (Line 495-496).

*Minor points:*

*The manuscript would still benefit from another round of proof reading. I have noted some problems below.*

Thanks for your suggestion, we have performed another round of proof reading.

*Abstract:*

*-Linkage misspelled, "14 linage groups"*

Sorry for the typo. The misspelling is corrected in the revision (Line 30).

*-carried is the wrong word, and the past tense is incorrect here: "carried 91.9% of core metazoan orthologs." Perhaps "possesses 91.9% of the core metazoan ortholog set"*

Sorry for the confusion. The sentence is corrected as "the genome possesses 91.9% of the core metazoan ortholog set" (Line 32).

*- "the sister taxon" not "a sister taxon" (also in methods section)*

Sorry for the mistake. We replace "a sister taxon" by "the sister taxon" in both abstract and method sections (Line 34 and Line 247).

*Introduction:*

*- "due to their high economic" not "due to the high"*

Sorry. We have corrected it in the revision. (Line 48).

*-"understanding of the larvae-juvenile" not "understanding of larvae-juvenile"*

Sorry for the mistake. We correct it in the revision (Line 64).

*-"widespread among metazoans" not "widespread among metazoan "*

Sorry for the mistake. We correct it in the revision (Line 68).

*-"Most studies" not "Most of studies"*

Sorry for the mistake. "of " is removed from that phrase (Line 74).

*-"hard-shelled mussel genetic breeding" not "the hard-shelled mussel genetic breeding" (no the)*

Sorry for the mistake. "the " is removed from that sentence in the revision (Line 80).

*- I suggest "molluscs" rather than "mollusks" in the last paragraph, to match your usage elsewhere*

Thanks for your suggestion, "mollusks" is replaced by "molluscs" (Line 88 and Line 375).

*Methods:*

*- no "a"s in "at a 57× coverage and the heterozygous peak was at a 28×" - this should read "at 57× coverage and the heterozygous peak was at 28×"*

Sorry for the mistake. "a" is removed from "at a 57× coverage and the heterozygous peak was at a 28×" in the revision (Line 152-153).

*-"usually occurs in fragmented assemblies" not "usually occurs in fragmented assembly",*

Sorry for the mistake. We correct it in the revision (Line 158).

*-"align to one or more of the InterPro " not "have the alignment to one or more of the InterPro"*

Thanks. The sentence is corrected as "35,471 protein-coding genes (94.6% of the 37,478 gene models) align to one or more of the InterPro " (Line 218).

*-"Chromosome-level genomes allow re-sequencing and population genetic studies." not "Chromosome-level genome is important for re-sequencing and population genetic."*

As your suggestions, the sentence is corrected as "Chromosome-level genomes allow re-sequencing and population genetic studies" (Line 251).

*-"preliminary assay" not "preliminary try "*

Thanks for your suggestions. We use “assay", instead (Line 252).

*-"Benchmark Universal Single-Copy Orthologs (BUSCO) v4.1.4" not "Benchmark Universal Single-Copy Orthologs BUSCO v4.1.4"*

As your suggestion, "Benchmark Universal Single-Copy Orthologs BUSCO v4.1.4" is replaced by "Benchmark Universal Single-Copy Orthologs (BUSCO) v4.1.4" (Line 354).

*Abbreviations"*

*no "the", TPM: Transcripts per Million;*

As your suggestion, "the" is removed (Line 388).

*Figure 3 legend (and possibly elsewhere) please italicise the k in kmer*

As your suggestion, we italicize the k in *k*-mer (Line 148-153, Line 587, Line 593).

*Figure 4 legend: gene names need italics*

Thanks for your suggestion. We italicize the gene names as *chitobiase* (Line 460-461).

*Fig 5 legend: "corresponding evolutionary relationship" not "corresponding evolution relationship"*

Thanks for your suggestion. We correct it in the revision (Line 475).

*-some bold font needed, Additional Files title page (pg 25)*

Thanks for your suggestion. Bold font is used in the revision (Line 513, Line 515).
